# FODMAP intake, gut microbiota, and gastroesophageal reflux disease: a triangulated study of Mendelian randomization and clinical validation

**DOI:** 10.3389/fnut.2026.1847736

**Published:** 2026-08-26

**Authors:** Yuanchun Chen, Shiyun Lu

**Affiliations:** 1 Department of Gastroenterology, Fuzhou University Affiliated Provincial Hospital (Fujian Provincial Hospital), Fuzhou, China; 2 Shengli Clinical Medical College, Fujian Medical University, Fuzhou, China

**Keywords:** FODMAP diet, gastroesophageal reflux disease, gut microbiota, mediation analysis, Mendelian randomization

## Abstract

**Background:**

Dietary factors related to fermentable oligosaccharides, disaccharides, monosaccharides and polyols (FODMAPs) and gut microbiota may influence gastroesophageal reflux disease (GERD), but causal evidence and clinical consistency remain uncertain.

**Objective:**

We tested whether FODMAP-related and GERD-relevant dietary traits influence GERD risk using Mendelian randomization (MR), and whether gut microbiota mediate these associations.

**Methods:**

We integrated UK Biobank dietary GWAS, MiBioGen microbiota GWAS, and FinnGen GERD summary statistics using inverse-variance weighted MR, multivariable MR, Benjamini-Hochberg false discovery rate (BH-FDR) correction, MR-Steiger filtering, and two-step mediation analysis. A 525 matched-pair retrospective case–control cohort provided clinical validation.

**Results:**

After BH-FDR correction, genetically predicted cheese, cereal, oily fish, fresh/dried fruit, and salad/raw vegetables were associated with lower GERD risk (ORs, 0.46–0.77), whereas tea and poultry were associated with higher risk (ORs, 1.28–2.14). Microbiota-wide and mediation signals did not survive FDR correction. The oily fish-*Erysipelatoclostridium* indirect effect was small and statistically imprecise (indirect effect, −0.015; 95% CI, −0.034 to 0.005), corresponding to a mediated proportion of 4.1% (95% CI, −1.2 to 9.5%); therefore, it was interpreted as an exploratory signal rather than evidence of a confirmed microbial pathway. Clinical data directionally supported the main dietary signals: tea, poultry, processed meat, and sweets were associated with higher GERD risk (ORs, 1.04–1.19), while cheese, cereal, fruit, and oily fish were protective (ORs, 0.80–0.94).

**Conclusion:**

Dietary associations with GERD were more robust than microbiota-associated signals. These findings suggest that oily fish-related dietary patterns may be associated with lower GERD risk, while exploratory analyses provide preliminary evidence that gut microbiota may contribute to these associations. Further studies are required to validate these potential biological pathways.

## 1 Introduction

Gastroesophageal reflux disease (GERD) has emerged as a formidable global chronic digestive disorder, characterized by the retrograde flow of gastric contents leading to heartburn, acid regurgitation, and various extra-esophageal manifestations (1). It significantly impairs patients’ quality of life and imposes a substantial medical burden. Epidemiological data indicate that while the prevalence in Western countries has long fluctuated between 10 and 20%, Asia has witnessed a sharp upward trend over the past decade—a phenomenon closely linked to lifestyle Westernization and rising obesity rates (2). Persistent and uncontrolled reflux can progress to reflux esophagitis, Barrett’s esophagus, and even esophageal adenocarcinoma (3). Therefore, identifying modifiable risk factors is of paramount clinical and public health significance for the prevention and long-term management of the disease.

Among various modifiable factors, dietary habits play a pivotal role in shaping GERD risk. Conventional wisdom holds that high-fat foods, caffeine, alcohol, and certain irritants promote reflux by inducing lower esophageal sphincter (LES) relaxation or altering gastric emptying (4). Recently, scientific attention has shifted toward fermentable oligosaccharides, disaccharides, monosaccharides, and polyols (FODMAPs). FODMAPs are poorly absorbed in the small intestine and are subsequently fermented by the gut microbiota in the colon, producing significant amounts of gas. Theoretically, this process may mechanically facilitate reflux by elevating intra-abdominal and intra-gastric pressure (5). The proven efficacy of low-FODMAP diets in alleviating bloating and abdominal pain in irritable bowel syndrome (IBS) suggests a potential biological plausibility for improving GERD symptoms by reducing gastrointestinal flatulence and abdominal pressure (6, 7). Although several small-scale clinical trials and observational studies have reported associations between FODMAP intake and GERD symptoms (8), research in 2024 further explored the impact of FODMAP diets on patients with overlapping GERD and IBS symptoms. Observational evidence suggests that high-FODMAP diets may increase transient lower esophageal sphincter relaxations (TLESRs), the primary pathophysiological mechanism of GERD. However, existing findings remain inconsistent, largely constrained by limited sample sizes and heterogeneous methods for measuring dietary exposure (9).

Beyond the amount of fermentable carbohydrate intake, the broader food matrix may also be relevant to GERD pathophysiology. Dietary patterns rich in fiber, polyphenols, unsaturated fatty acids, and other bioactive compounds can reshape gut microbial ecology and microbial metabolite production, while also influencing host inflammatory tone and epithelial barrier function. Recent evidence on Mediterranean diet foods suggests that natural food matrices, including fruits, vegetables, legumes, whole grains, extra-virgin olive oil, nuts, and fish, can modulate gut microbiota composition and microbial metabolites (10). Although this evidence is not specific to GERD, it provides an important biological context for considering diet-microbiota interactions beyond a simple high- versus low-FODMAP classification.

Natural products and food-derived bioactive substances have also been discussed as potential adjunctive approaches for GERD management. Dietary fiber, plant polyphenols, alginates, omega-3 polyunsaturated fatty acids, and selected probiotic or prebiotic strategies may theoretically affect reflux-related mechanisms through gastric emptying, transient lower esophageal sphincter relaxations, esophageal mucosal protection, oxidative stress, inflammation, and gut microbiota modulation (11). However, the current clinical evidence remains heterogeneous and is insufficient to support broad therapeutic claims. These observations nevertheless strengthen the rationale for evaluating specific dietary components and microbial pathways in GERD using approaches less vulnerable to confounding and reverse causation.

Current observational evidence is insufficient to establish a definitive causal relationship between FODMAPs and GERD, primarily due to two major biases. First, confounding bias: high FODMAP intake often co-occurs with higher BMI, poor overall dietary patterns, or specific lifestyle factors that independently affect GERD risk and are difficult to fully adjust for (12). Second, reverse causality is a concern because GERD patients may passively or actively alter their diets—including reducing or selectively avoiding certain foods—based on the severity of their symptoms, leading to confusion regarding the causal direction (4). Consequently, relying solely on traditional cohort or case–control studies makes it difficult to derive robust causal inferences in the presence of unmeasured confounding and unclear temporal sequences.

Concurrently, the gut microbiota has gained increasing recognition as the core mediator through which FODMAPs exert their physiological effects in GERD pathogenesis (13). The gut microbiota does not merely influence the intestinal–gastric pressure gradient via FODMAP fermentation; rather, alterations in microbial composition (dysbiosis) and metabolic byproducts—such as short-chain fatty acids (SCFAs)—may modulate esophageal motility, LES function, and mucosal barrier integrity via a complex “gut–esophagus axis” involving neuroendocrine and immunological pathways (14, 15). A study underscored that GERD patients exhibit diminished microbial diversity, and specific genera (e.g., a reduction in *Bifidobacterium*) are associated with increased TLESRs (14).

Although existing research has identified distinct gut microbial signatures in GERD patients compared to healthy controls (such as an increased abundance of the Proteobacteria phylum), these observational findings remain unable to address the direction of causality: does dysbiosis predispose individuals to GERD, or does the chronic pathological state of GERD—including the prolonged use of proton pump inhibitors (PPIs)—reshape the microbial ecosystem? (16). To address this, a 2024 Mendelian randomization (MR) study utilized genetic instrumental variables (IVs) to investigate the causal link between gut microbiota and GERD, finding that specific taxa (e.g., *Ruminococcaceae)* may elevate GERD risk through mediating pathways (17).

To circumvent the aforementioned limitations, this study employs the MR framework for causal inference (18). MR leverages genetic variants—specifically single nucleotide polymorphisms (SNPs) associated with exposures and randomly allocated at conception—as IVs. This approach effectively serves as a “natural” randomized controlled trial (RCT), thereby mitigating the inherent biases of confounding and reverse causality (18). In recent years, the application of MR in nutritional and microbiome epidemiology has expanded rapidly, proving to be a robust tool for establishing causal evidence in complex exposure–outcome relationships (19).

Utilizing large-scale genome-wide association study (GWAS) data, the present study represents the first systematic application of two-sample MR to evaluate the potential causal effects of diverse FODMAP components on GERD risk. Furthermore, we investigate whether the gut microbiota plays a mediating or moderating role within this association. Through the rigorous selection of genetic instruments and an array of sensitivity analyses, integrated with external validation from an independent clinical case–control cohort, we aim to provide evidence to inform future dietary evalution. Demonstrating a causal link between specific FODMAP intake and GERD susceptibility would provide a critical foundation for personalized dietary interventions and precision disease prevention strategies.

## 2 Methods

### 2.1 Overview of study design

**Figure 1:**
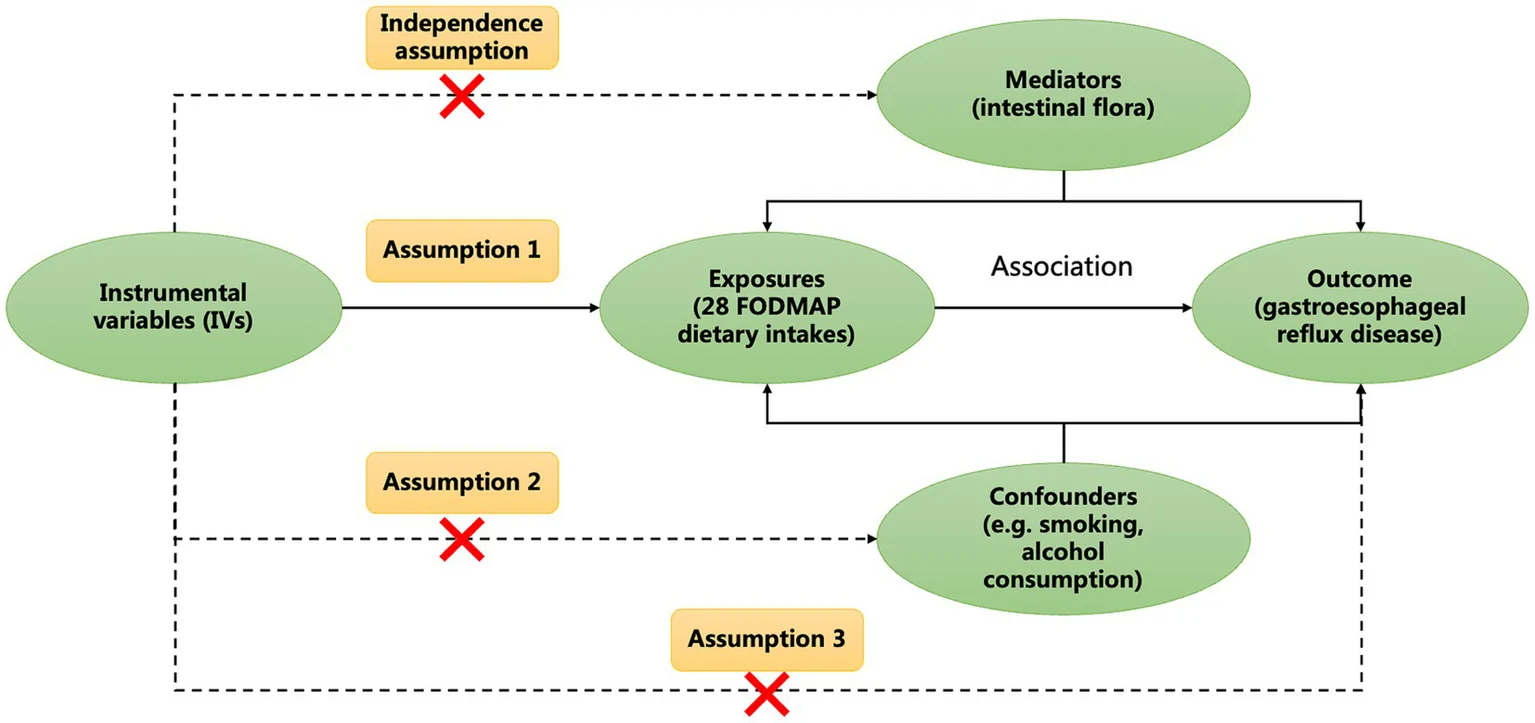
Schematic overview of the Mendelian Randomization (MR) analysis framework used in this study. The flowchart illustrates the sequential steps undertaken: FODMAP-related and GERD-relevant dietary items as exposures, gut microbiota as the candidate mediator, and GERD as the outcome. First, the total effect of dietary items on GERD was assessed. Next, the effect of each gut microbiota on GERD was evaluated, followed by an assessment of the effect of diet on gut microbiota levels. Finally, the proportion of the diet-GERD association mediated by gut microbiota was calculated. This schematic presents the overall study design only; the exploratory mediation path estimates and their 95% CIs are reported in Table 1 and Figure 5.

This study adhered to the Strengthening the Reporting of Observational Studies in Epidemiology using Mendelian Randomization (STROBE-MR) guidelines. We refer to the Supporting Information for the full STROBE-MR checklist. The MR analyses were based on GWAS datasets of predominantly European ancestry to minimize population stratification, whereas the clinical validation cohort was recruited from Fujian Provincial Hospital in China and was used as an external phenotypic validation rather than an ancestry-matched genetic replication. Our investigation relied on three fundamental assumptions of instrumental variable (IV) analysis (Figure 1).

First, the Relevance Assumption requires that genetic variants, specifically SNPs, show a robust and significant association with exposure factors like FODMAP dietary components or gut microbiota.

Second, the Independence Assumption states that selected genetic variants must be independent of known and unknown confounders affecting the relationship between the exposure and the outcome (GERD).

Third, the Exclusion Restriction Assumption dictates that genetic variants influence GERD risk exclusively through the exposure factor. There must be no evidence of horizontal pleiotropy.

### 2.2 MR analysis framework

The MR analysis in this study proceeded through three progressive stages. First, we performed causal association screening. We initially employed univariable MR (UVMR) to evaluate the causal effects of 28 FODMAP-related and GERD-relevant dietary items selected from available UK Biobank dietary GWAS traits on GERD. These items were not intended to represent a quantitative total FODMAP intake score, but rather a set of dietary traits relevant to FODMAP exposure, fermentative potential, and GERD-related dietary patterns. Subsequently, we used multivariable MR (MVMR) to genetically adjust for key confounders, including smoking, alcohol consumption, and BMI. This step isolated the independent biological contribution of dietary factors. Second, we identified microbial mediators. We systematically screened gut microbial taxa that exhibited a robust causal association with GERD. Third, we quantified mediation mechanisms. A two-step MR approach was utilized to construct the mediation model. We calculated the mediation proportion using the product method. This method is defined as the product of the causal effect of the exposure on the mediator (β_exposure-mediator_) and the causal effect of the mediator on GERD (β_mediator-outcome_). This approach allowed us to quantify the contribution of microecological pathways within the diet-disease axis.

### 2.3 Methods

To evaluate whether the MR-derived dietary signals were directionally supported in a real-world clinical setting, we conducted a retrospective case–control study based on electronic medical records (EMR) and food frequency questionnaires (FFQ) at Fujian Provincial Hospital, Affiliated with Fuzhou University. Because this clinical cohort was recruited in China, it was treated as external phenotypic validation rather than an ancestry-matched genetic replication of the European GWAS evidence. This study was approved by the Institutional Ethics Committee (K2026-04-042).

#### 2.3.1 Study population and inclusion criteria

A total of 525 patients with GERD (case group) and 525 baseline-matched individuals (control group) treated at our hospital between 2020 and 2025 were included.

The inclusion criteria for the case group were as follows. (i) Age ≥18 years, with complete medical records and valid FFQ data. (ii) Definitive clinical diagnosis evidenced by: endoscopic findings of reflux esophagitis (Los Angeles (LA) grades A–D), positive 24-h esophageal pH monitoring results, or frequent typical symptoms of reflux /heartburn (at least 2 times per week). (iii) Documented history of acid-suppressant therapy, specifically the continuous use of PPIs or Vonoprazan for ≥8 weeks.

The inclusion criteria for the control group were individuals who visited the hospital during the same period, aged ≥18 years, with no diagnosis of GERD, no history of persistent antacid therapy, and no relevant reflux symptoms.

To eliminate secondary factors that might interfere with the association between diet and symptoms, the following exclusion criteria were applied. (i) History of esophageal or gastric malignancy or major gastrointestinal surgery. (ii) Chronic wasting diseases significantly affecting gastrointestinal function (e.g., severe hepatic or renal insufficiency, inflammatory bowel disease, or chronic pancreatitis). (iii) Pregnancy or lactation. (iv) Major dietary interventions or extreme fluctuations in body weight within the past 6 months. (v) Missing FFQ data or logical inconsistencies (e.g., extreme outliers in total energy intake).

### 2.4 Data sources

#### 2.4.1 Dietary exposure data from UK Biobank

We leveraged summary statistics for 28 dietary exposures from the UK Biobank (UKB) GWAS. This dataset includes genetic association data from approximately 450,000 participants of European ancestry. Because UK Biobank does not provide a direct measure of total FODMAP intake, we defined these exposures as FODMAP-related and GERD-relevant dietary items rather than as a quantitative total FODMAP score. The 28 items were selected based on the availability of GWAS summary statistics, their reported FODMAP content or fermentative potential, their relevance to dietary advice in GERD, and their correspondence to the clinical FFQ items used in our validation cohort. Accordingly, some items represent FODMAP-containing foods or mixed aggregate traits (e.g., fruit, cooked vegetables, salad/raw vegetables, cereal), whereas others were included as low- or negligible-FODMAP comparators or GERD-relevant non-FODMAP dietary exposures. Items such as tea, coffee, alcoholic beverages, meat, and fish should therefore not be interpreted as typical high-FODMAP foods; for example, tea was included as a GERD-relevant low- or negligible-FODMAP beverage exposure rather than as a FODMAP food. Summary information on the GWAS data sources is provided in Supplementary Table S1. A detailed classification and rationale for each dietary item, including FODMAP category, primary FODMAP component where applicable, role in this study, and supporting references, are provided in Supplementary Table S2. Detailed questionnaire items and inquiry lists are provided in Supplementary Table S3. The comprehensive GWAS summary data were retrieved from the IEU OpenGWAS database (available at https://opengwas.io/).

#### 2.4.2 Gut microbiota data from MiBioGen

Genetic variants associated with the gut microbiota were obtained from the MiBioGen consortium. This large-scale meta-analysis integrated 24 cohorts totaling 18,340 individuals, the majority of whom are of European descent (*n* = 13,266). Microbial composition was characterized using 16S rRNA gene sequencing targeting variable regions V1–V2, V3–V4, and V4. Through microbiome quantitative trait loci (mBQTL) mapping, the consortium identified genetic associations across 211 bacterial taxa spanning five biological ranks: phylum, class, order, family, and genus. Detailed datasets are accessible via the MiBioGen portal.^1^

#### 2.4.3 GERD outcome data from FinnGen

The summary statistics for GERD were derived from the FinnGen research project (Release 11 / R11). This dataset comprises 129,080 GERD cases and 473,524 controls, encompassing approximately 2,320,781 SNPs. To minimize bias arising from population stratification, all participants were restricted to those of European ancestry. The dietary exposure GWAS was derived from UK Biobank, whereas GERD summary statistics were obtained from FinnGen; based on the source cohort descriptions, there was no known participant overlap between these datasets, reducing the likelihood of bias from sample overlap in the two-sample MR design.

#### 2.4.4 Ethical considerations

As all data utilized in this study were sourced from de-identified, publicly available summary statistics with prior ethical approval and informed consent from the respective original studies, no additional institutional review board (IRB) approval was required for this investigation.

#### 2.4.5 Data extraction for clinical validation study

To validate the findings derived from the MR analysis, we established a retrospective clinical cohort at Fujian Provincial Hospital, Affiliated with Fuzhou University. We extracted demographic characteristics such as sex, age, height, weight, and calculated BMI, along with clinical diagnoses, medical history, medication history, and diagnostic test results including electronic gastroscopy reports and 24-h pH monitoring data, from the hospital’s Electronic Medical Records. The dietary structure of participants over the past year was evaluated using a validated FFQ that covered major food categories including tea, coffee, poultry, cheese, fresh fruit, salad vegetables, fish, cereals, and dried fruits. Based on self-reported data or face-to-face interviews, food intake frequency and typical portion sizes were converted into standardized weekly intake metrics based on questionnaire-defined reference portions or small reference cups and treated as continuous variables for statistical analysis. The 50 g and 50 mL values were FFQ-specific reference units for semi-quantitative conversion, not national dietary guideline serving sizes or a conventional household cup; therefore, the revised terminology avoids re-scaling the original exposure variables and preserves the clinical regression definitions (20, 21).

### 2.5 Selection of instrumental variables

To ensure the reliability of the causal inferences, we implemented a rigorous multi-step screening procedure to construct the IVs.

#### 2.5.1 Genetic variation extraction thresholds

Dietary Exposures: Given that the heritability of dietary habits is generally modest, we adopted a genome-wide suggestive significance threshold (*p* < 5 × 10^−6^) to identify SNPs associated with the 28 FODMAP-related and GERD-relevant dietary items selected from UK Biobank.

Gut Microbiota: Following the conventions of the MiBioGen consortium and established microbial MR studies, a threshold of *p* < 1 × 10^−5^ was utilized to capture a broader range of genetic variants associated with the abundance of bacterial taxa.

GERD Outcome: As a binary disease trait, we strictly adhered to the genome-wide significance threshold (*p* < 5 × 10^−8^) to identify SNPs associated with GERD risk.

#### 2.5.2 Linkage disequilibrium clumping and independence testing

To ensure the independence of the selected IVs, we performed Linkage Disequilibrium (LD) clumping analysis on the initially identified SNPs. We set a clumping window of 10,000 kb and a threshold of r^2^ < 0.001, retaining only the SNP with the lowest *p*-value within each genomic region. This step ensured that the finalized IVs were mutually independent, thereby minimizing bias introduced by LD.

#### 2.5.3 Data harmonization and allele alignment

Prior to the formal MR analysis, summary statistics for exposures and outcomes underwent rigorous Data Harmonization. We verified the consistency of allele orientations and strictly excluded palindromic SNPs and those with intermediate effector allele frequencies (EAF near 0.5). This precaution was taken to prevent misinterpretation of causal effects due to strand ambiguity.

#### 2.5.4 Assessment of instrumental variable strength

To mitigate the risk of weak instrument bias, we calculated the F-statistic for each SNP using the following formula:

$\;\;F=\frac{R^{2}\times (N-1-k)}{(1-R^{2})\times k}$ ,

Where N represents the sample size of the exposure, and k denotes the number of IVs. The variance explained (R^2^) was calculated as


$$R^{2}=\frac{2\times \beta^{2}\times \mathrm{EAF}\times (1-\mathrm{EAF})}{2\times \beta^{2}\times \mathrm{EAF}\times (1-\mathrm{EAF})+2\times SE^{2}\times N\times \mathrm{EAF}\times (1-\mathrm{EAF})},$$

In these equations, β is the effect size of the allele, SE is the standard error, and EAF is the effect allele frequency. In accordance with statistical conventions, only SNPs with an F-statistic >10 were retained for the final causal inference (22). Detailed genetic instruments for FODMAP-related dietary traits, gut microbiota taxa, and confounders are provided in Supplementary Tables S4–S6.

### 2.6 Statistical analysis

#### 2.6.1 Causal relationship between FODMAP diet and gastroesophageal reflux disease

This study employed a two-sample MR framework, utilizing SNPs as IVs to infer the causal relationship between FODMAP-related and GERD-relevant dietary traits and GERD. The analysis strictly adhered to the three core assumptions of MR: (i) the IVs are robustly associated with the exposure (dietary intake; relevance assumption); (ii) the IVs are independent of any potential confounding factors (independence assumption); and (iii) the IVs influence the outcome solely through the exposure and not via alternative pathways (exclusion restriction assumption, implying no horizontal pleiotropy).

##### Univariable Mendelian randomization analysis

We employed multiple MR methods to evaluate the causal impact of the 28 FODMAP-related and GERD-relevant dietary items on GERD. In the UVMR analysis, the inverse-variance weighted (IVW) method served as the primary approach to assess the causal effect of each dietary trait on GERD, as it provides the greatest statistical power under the premise of no horizontal pleiotropy. To address multiple testing, Benjamini-Hochberg false discovery rate (BH-FDR) correction was applied across the 28 dietary exposures. Associations with FDR-adjusted *p* values < 0.05 were considered FDR-supported dietary findings, whereas associations with nominal p values < 0.05 but FDR-adjusted p values ≥ 0.05 were interpreted as suggestive only. Causal estimates were presented as odds ratios (ORs) with corresponding 95% CIs. To verify the robustness of our findings, four complementary analytical methods were concurrently applied: MR-Egger regression, Weighted Median, Simple Mode, and Weighted Mode. For instance, the Weighted Median method yields valid estimates of the exposure-outcome relationship even when up to 50% of the SNPs are invalid instruments.

##### Multivariable Mendelian randomization analysis

To determine whether the causal effect of FODMAP dietary intake on GERD is robust after accounting for potential lifestyle and metabolic traits, we constructed three separate MVMR models. Specifically, body mass index (BMI), smoking status, and alcohol drinking frequency were individually incorporated into the analysis as additional covariates, rather than including all factors simultaneously. This strategy aimed to evaluate the robustness of the main association with respect to each specific factor. These analyses should be interpreted as three distinct single-covariate MVMR sensitivity models (diet plus BMI, diet plus smoking, or diet plus alcohol), not as a single jointly adjusted model containing all three covariates. The separate-model strategy was used to assess robustness to each covariate individually; therefore, the estimates are not mutually adjusted for BMI, smoking, and alcohol.

##### Data sources

The GWAS summary statistics for BMI, smoking, and alcohol consumption were all derived from large-scale studies of European descent, with sample sizes of *N* = 461,460 (ukb-b-19953), *N* = 462,434 (ukb-b-223), and *N* = 462,346 (ukb-b-5779), respectively.

Instrumental Variable Construction: For each set of MVMR analyses (e.g., diet + BMI), we first independently screened for significant and independent SNPs associated with “diet” and the “specific covariate” (e.g., BMI) utilizing the thresholds of *p* < 5 × 10^−6^, r^2^ < 0.001, and a clumping window of 10,000 kb. Subsequently, the union of the two SNP sets was adopted as the combined genetic instrumental variable set for the model. For each SNP in this union, we extracted its effect estimates ( β ), standard errors (SE), and effect allele frequencies (EAF) across the two exposures (diet and the covariate) and the GERD outcome. Any SNP with missing data in either exposure was excluded.

##### Statistical analysis

The multivariable inverse-variance weighted (MVMR-IVW) method was used to fit the models, estimating the direct causal effect of dietary intake on GERD after adjusting for a specific covariate (BMI, smoking, or alcohol consumption). A significant attenuation in the adjusted causal estimates compared to the UVMR results would suggest that the covariate may act as a confounder or mediator in the association between diet and GERD.

##### Sensitivity analysis and quality control

To ensure the reliability of our causal inferences, we performed comprehensive sensitivity analyses.

##### Horizontal pleiotropy assessment

The intercept of the MR-Egger regression was evaluated to detect directional horizontal pleiotropy (an intercept *p* > 0.05 indicates no significant horizontal pleiotropy). Concurrently, the MR-PRESSO (Pleiotropy Residual Sum and Outlier) global test was applied to identify and remove outliers, followed by a re-estimation of the causal effects.

Heterogeneity test: Cochran’s Q statistic was utilized to detect heterogeneity among the IVs, where a *p* > 0.05 suggests the absence of significant heterogeneity.

##### Causal direction validation

The MR Steiger filtering method was employed to verify the causal direction of each retrieved SNP regarding the exposure and outcome. Only SNPs yielding a “TRUE” result (indicating that the variance explained by the IV is significantly greater for the exposure than for the outcome) were retained, thereby excluding potential reverse causation. We additionally summarized the number of harmonized instruments before filtering, the number removed by MR-Steiger filtering, and the final number retained for each diet-GERD, diet-microbiota, and microbiota-GERD analysis. A summary of MR-Steiger filtering across the diet-GERD, diet-microbiota, and microbiota-GERD analyses is provided in Supplementary Table S16, with pair-level filtering details listed in Supplementary Table S17.

Robustness evaluation: A leave-one-out analysis was conducted by sequentially omitting each SNP to observe its impact on the overall causal estimate, thus ruling out the possibility that the results were driven by any single genetic variant.

##### Data visualization and software implementation

All statistical analyses were performed using R software (version 4.4.1) along with three R packages: “MVMR,” “TwoSampleMR,” and “Mendelian Randomization.” The analytical results were visualized using scatter plots, funnel plots, and forest plots. Following the BH-FDR framework for multiple testing and the STROBE-MR emphasis on transparent reporting of MR analyses, we prespecified separate multiplicity families for dietary MR, microbiota MR, and mediation screening (23, 24). For microbiota MR analyses, BH-FDR correction was applied across the 211 microbial taxa. Microbial associations that did not survive FDR correction were reported as nominal exploratory signals. Except for the significance threshold adjusted for multiple testing, a two-sided *p* < 0.05 was considered statistically significant for all other statistical tests. The predefined multiple-testing families and corresponding BH-FDR correction results are summarized in Supplementary Table S7.

#### 2.6.2 Mendelian randomization mediation analysis (MR-mediation analysis)

To quantitatively evaluate the mediating role of gut microbiota in the relationship between the FODMAP diet and GERD risk, we employed a two-step MR design to construct a mediation model. This analysis decomposed the total effect into a direct effect and an indirect effect.

Step 1, Assessing the causal effect of the FODMAP diet on the abundance of candidate gut microbiota ( $\beta_{a}$ );

Step 2, Evaluating the causal effect of the gut microbiota on GERD risk ( $\beta_{b}$ ).

The indirect effect was estimated as the product of the two-stage causal estimates $\;\beta_{\text{indirect}}=\beta_{a}\times \beta_{b}$ , where $\beta_{a}$ represents the diet-to-mediator association and $\beta_{b}$ represents the mediator-to-GERD association. The proportion mediated was calculated as $\beta_{\text{indirect}}/\beta_{\text{total}}$ , where $\beta_{\text{total}}$ represents the total diet-to-GERD effect. We used the Delta method to calculate 95% CIs for both the indirect effect and the mediated proportion. Because mediation screening involved multiple diet-microbiota pairs, BH-FDR correction was considered across the tested pairs; mediation findings that did not survive FDR correction or had CIs crossing the null were interpreted as hypothesis-generating rather than confirmatory biological pathways.

#### 2.6.3 Analysis of clinical validation cohort

To validate the consistency of our genetic causal inferences within a real-world clinical setting, we conducted an analysis using case–control data obtained from Fujian Provincial Hospital Affiliated to Fuzhou University. All statistical analyses for this validation were performed using SPSS software (version 26.0).

##### 2.6.3.1 Baseline characteristics description

Continuous variables were initially tested for normality. Normally distributed data were presented as mean ± standard deviation ( $\bar{x}$ ±s), and differences between groups were compared using the independent samples t-test. Non-normally distributed data were expressed as median and interquartile range (M(IQR)), with group comparisons performed via the Mann–Whitney U test. Categorical variables were presented as frequencies and percentages, and analyzed using the χ^2^ test.

##### 2.6.3.2 Association analysis

Logistic regression models were constructed with GERD disease status as the dependent variable and dietary intake indicators (assessed by the FFQ) as independent variables. ORs and 95% CIs were calculated to evaluate the associations between distinct dietary factors and the risk of GERD.

##### 2.6.3.3 Confounding bias control

To obtain independent effect estimates of dietary factors on GERD risk, we constructed multivariable logistic regression models. These models adjusted for potential confounding factors, including age, sex, smoking status, alcohol consumption, and BMI, thereby yielding adjusted ORs and 95% CIs.

## 3 Results

### 3.1 Relationship between FODMAP-related diet and GERD

In this study, the IVs utilized for analysis demonstrated genome-wide significance, with all F-statistics exceeding 10, confirming the robustness of these genetic instruments against weak instrument bias. Detailed characteristics of these SNPs are provided in Supplementary Table S4.

**Figure 2:**
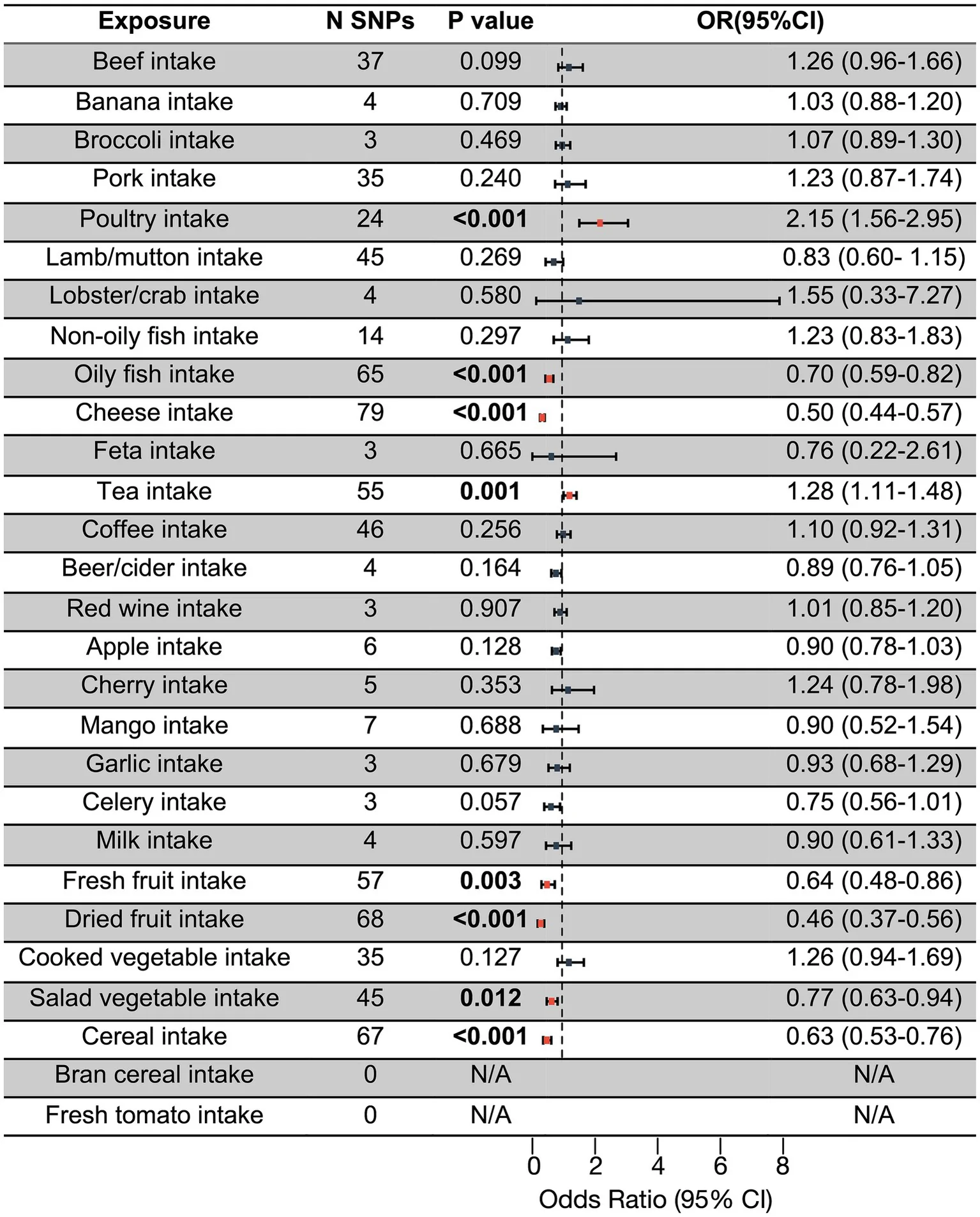
Mendelian randomization estimates for FODMAP-related and GERD-relevant dietary items in relation to GERD risk. Forest plot displaying the MR estimates for each dietary item. For each row, the number of SNP instruments (*n*SNP), the primary estimator (IVW), *p*-value, and the odds ratio (*OR*) with 95% confidence interval (*CI*) per 1-standard deviation increase in genetically predicted intake are shown. *OR* values below 1 indicate a lower risk of GERD. Nominally significant associations (*p* < 0.05) are highlighted. N/A indicates that no eligible SNP instruments remained after the prespecified instrument-selection and harmonization steps; therefore, the IVW *p* value and *OR* could not be estimated for fresh tomato intake or bran cereal intake.

As illustrated in Figure 2, genetically predicted intake of Oily fish (ukb-b-2209), Cheese (ukb-b-1489), Fresh fruit (ukb-b-3881), Dried fruit (ukb-b-16576), Cereal (ukb-b-15926), and Salad/raw vegetables (ukb-b-1996) were associated with a reduced risk of GERD and remained FDR-supported after BH correction across the 28 dietary exposures. Based on the primary IVW method, the estimates were: Oily fish: OR = 0.70 (95% CI: 0.59–0.82, *p* = 1.21 × 10^−5^); Cheese: OR = 0.50 (95% CI, 0.44–0.57, *p* = 1.54 × 10^−25^); Fresh fruit: OR = 0.64 (95% CI, 0.48–0.85, *p* = 0.002); Dried fruit: OR = 0.46 (95% CI, 0.37–0.56, *p* = 1.86 × 10^−13^); Cereal: OR = 0.63 (95% CI, 0.52–0.76, *p* = 1.7 × 10^−6^); Salad/raw vegetables: OR = 0.77 (95% CI, 0.63–0.94, *p* = 0.012). Sensitivity analyses provided further support: the association for oily fish was consistent with the Weighted Median method; cheese intake was supported by both Weighted Median and Weighted Mode; fresh fruit was supported by Weighted Median and Simple Mode; and both dried fruit and cereal intake were validated by Weighted Median, Simple Mode, and Weighted Mode methods. Notably, MR-Egger regression only identified a significant association for dried fruit intake.

Conversely, Tea (ukb-b-6066) and Poultry (ukb-b-8006) intake were associated with an increased risk of GERD and remained FDR-supported after BH correction across the 28 dietary exposures (IVW method: Tea OR = 1.28, 95% CI: 1.10–1.48, *p* = 0.0007; Poultry OR = 2.14, 95% CI: 1.55–2.95, *p* = 2.90 × 10^−6^). The causal estimate for tea was supported by Weighted Median, Simple Mode, and Weighted Mode, while the poultry-GERD association was corroborated by the Weighted Median method. However, MR-Egger regression did not show significant associations for these two factors. No significant causal relationships were observed between the remaining 20 dietary factors and GERD risk (detailed data in Supplementary Table S8; Supplementary Figures 1–78). Alternative MR estimates and MR-PRESSO results for the eight dietary findings are summarized in Supplementary Tables S9, S10.

#### 3.1.1 Multivariable MR adjusting for potential confounders

**Figure 3:**
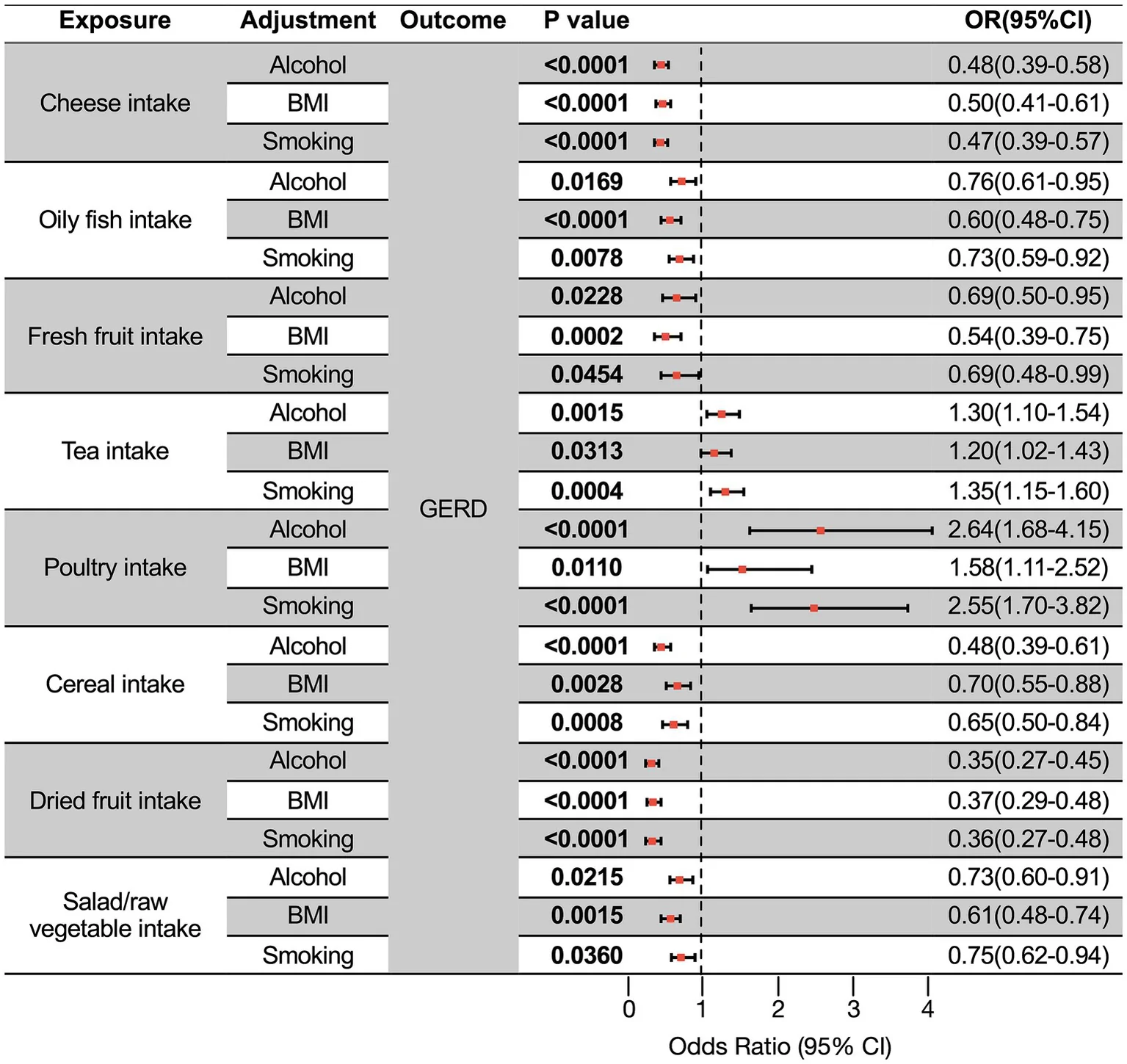
Exploratory multivariable Mendelian randomization (MVMR) sensitivity analyses of FODMAP-related and GERD-relevant dietary items in relation to GERD risk. Forest plot displaying estimates from three separate single-covariate MVMR models, each additionally adjusting for one covariate (diet plus alcohol consumption, diet plus BMI, or diet plus smoking). These analyses represent distinct sensitivity models rather than a single jointly adjusted model containing all covariates simultaneously. This strategy was used to evaluate robustness under alternative adjustment scenarios while avoiding potential model instability caused by limited instrument strength and possible correlations among covariates.

To assess the independent effects of the eight dietary factors identified in the UVMR, we performed MVMR analyses adjusting for BMI, smoking, and alcohol consumption (Figure 3). In the IVW models, the negative associations between GERD risk and the intake of oily fish, cheese, cereal, fresh fruit, dried fruit, and salad/raw vegetables remained statistically significant after adjustment. Similarly, the positive correlations for tea and poultry intake persisted. These results suggest that dietary patterns characterized by higher intake of specific low-FODMAP components (such as oily fish and certain cheeses) may significantly lower the risk of developing GERD.

#### 3.1.2 Sensitivity analysis and heterogeneity

We employed the MR-Egger intercept test and Cochran’s Q test to evaluate horizontal pleiotropy and heterogeneity, respectively. As shown in Supplementary Table S8, the MR-Egger intercept tests revealed no evidence of directional horizontal pleiotropy (*p* > 0.05). However, Cochran’s Q test indicated significant heterogeneity among SNPs in several analyses (P_heterogeneity_ < 0.05), including cheese (*p* = 1.95 × 10^−7^), fresh fruit (*p* = 3.57 × 10^−11^), dried fruit (*p* = 1.69 × 10^−11^), tea (*p* = 5.91 × 10^−9^), and poultry (*p* = 0.0008), among others (Supplementary Table S8). Therefore, although the absence of directional pleiotropy and the leave-one-out results support the stability and direction of the main dietary associations, the IVW estimates should still be interpreted with appropriate caution in the presence of heterogeneity. Leave-one-out sensitivity analysis (Supplementary Figures 79–104) showed that the pooled effect estimates did not fluctuate substantially after sequential exclusion of individual SNPs, suggesting that the main associations were not driven by a single influential variant, while residual heterogeneity or pleiotropy cannot be completely excluded.

### 3.2 Causal relationship between gut microbiota and GERD

In our study, all IVs for the gut microbiota were selected based on a significance threshold of *p* < 1 × 10^−5^, ensuring adherence to the core MR assumptions. The F-statistics for all microbiota-related IVs exceeded 10, indicating a low risk of weak instrument bias.

**Figure 4:**
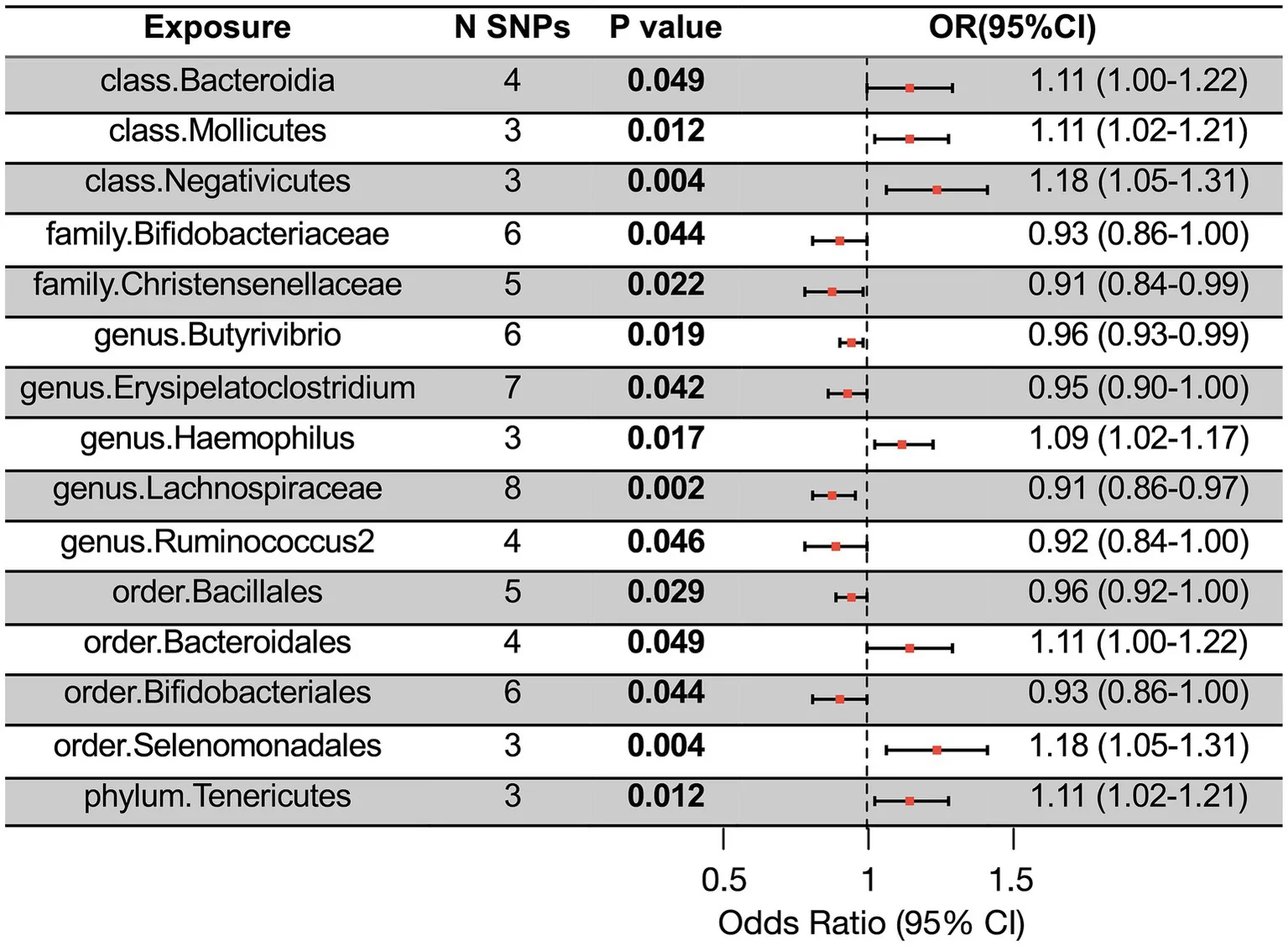
Exploratory Mendelian randomization estimates for the association between gut microbiota and GERD risk. Forest plot displaying the MR estimates for each plotted taxon. *p* values shown in the plot are nominal and uncorrected. None of the associations across the 211 tested taxa survived BH-FDR correction. Accordingly, all plotted associations should be interpreted as exploratory rather than confirmatory causal findings.

The primary IVW analysis results are detailed in Figure 4 and Supplementary Table S11. Among the 211 gut microbiota taxa analyzed, 15 taxa showed nominal associations with genetic susceptibility to GERD at *p* < 0.05; however, none survived BH-FDR correction across all microbial taxa. Therefore, these microbiota results were interpreted as exploratory signals rather than confirmatory causal associations. Eight nominally protective taxa were observed: *Bifidobacteriaceae* and its corresponding order *Bifidobacteriales* shared identical effect estimates (OR = 0.93, 95% CI: 0.86–1.00, *p* = 0.04). Other nominally protective taxa included *Lachnospiraceae UCG004* (OR = 0.91, 95% CI: 0.86–0.97, *p* = 0.002), *Ruminococcus 2* (OR = 0.92, 95% CI: 0.84–0.99, *p* = 0.04), *Erysipelatoclostridium* (OR = 0.95, 95% CI: 0.90–0.99, *p* = 0.04), *Christensenellaceae* (OR = 0.92, 95% CI: 0.84–0.99, *p* = 0.02), *Butyrivibrio* (OR = 0.96, 95% CI: 0.93–0.99, *p* = 0.02), and *Bacillales* (OR = 0.96, 95% CI: 0.92–0.99, *p* = 0.02).

Sensitivity analyses provided supportive but exploratory evidence for several nominal microbiota signals. Specifically, the Weighted Median method further supported the findings for *Bifidobacteriaceae, Bifidobacteriales, LachnospiraceaeUCG004*, and *Erysipelatoclostridium* (all nominal *p* < 0.05). Because these taxa did not survive microbiota-wide FDR correction, they should be interpreted as prioritized candidates for future validation rather than definitive microbial determinants of GERD.

Conversely, seven microbiota taxa showed nominal risk-associated signals for GERD. *Bacteroidia* and its corresponding order *Bacteroidales* showed nominal detrimental effects (OR = 1.11, 95% CI: 1.01–1.22, *p* = 0.049). Other nominal risk-related taxa included *Mollicutes* (OR = 1.11, 95% CI: 1.02–1.21, *p* = 0.01), *Negativicutes* (OR = 1.18, 95% CI: 1.05–1.31, *p* = 0.003), *Haemophilus* (OR = 1.09, 95% CI: 1.02–1.17, *p* = 0.01), *Selenomonadales* (OR = 1.18, 95% CI: 1.05–1.31, *p* = 0.003), and *Tenericutes* (OR = 1.11, 95% CI: 1.02–1.21, *p* = 0.01). Among these, the positive associations of *Mollicutes, Haemophilus*, and *Tenericutes* with GERD risk were further supported by the Weighted Median method, but these findings remained exploratory because they did not survive FDR correction.

No evidence of directional horizontal pleiotropy was detected in the sensitivity analysis (MR-Egger intercept *p* > 0.05, Supplementary Table S11). Although varying degrees of heterogeneity were observed in certain taxa (Cochran’s Q test *p* < 0.05), the leave-one-out analysis demonstrated that the sequential exclusion of any single SNP did not exert a disproportionate influence on the overall causal estimates (Supplementary Figures 150–164). Additionally, scatter plots, funnel plots and forest plots for the 15 nominally associated taxa (Supplementary Figures 105–149) exhibited broadly symmetrical effect distributions across IVs and consistency in the direction of causality. MR-PRESSO results for the 15 nominally associated taxa are summarized in Supplementary Table S12. These robustness checks support the biological plausibility of the nominal signals, but do not override the need for cautious interpretation after FDR correction.

MR-Steiger filtering was re-evaluated using the harmonized SNP-level datasets to improve transparency. In the DIET-GERD analysis, 702 harmonized SNP-exposure records across 27 dietary exposure-GERD pairs supported the diet-to-GERD direction, and no SNPs were removed. In the DIET-GUT analysis, 319,371 harmonized SNP–taxon records across 5,697 diet-microbiota pairs were assessed; 129,813 records (40.6%) did not support the hypothesized diet-to-microbiota direction and were excluded from directionally consistent analyses. These records represent accumulated SNP–exposure–taxon combinations across exposure–taxon pairs rather than 319,371 unique genetic variants. In the GUT-GERD analysis, all 70 harmonized SNP-exposure records across 15 microbiota-GERD associations supported the microbiota-to-GERD direction, and no SNPs were removed.

### 3.3 Mediating effect of gut microbiota in the association between diet and GERD

After conducting two-sample MR analyses between the eight FDR-supported dietary factors and the nominally associated GERD-related microbiota taxa, an exploratory link was observed between genetically predicted oily fish intake (ukb-b-2209) and the abundance of *Erysipelatoclostridium.* Specifically, each one-standard-deviation (SD) increase in genetically predicted oily fish intake was associated with a higher abundance of *Erysipelatoclostridium* in the nominal IVW analysis ( $\beta_{a}$ = 0.277, SE = 0.12, OR = 1.32, 95% CI: 1.03–1.68, *p* = 0.025). Further details of these MR analyses are presented in Supplementary Tables S13, S14, with corresponding scatter plots, funnel plots, and forest plots provided in Supplementary Figures 165–167.

Subsequent analysis showed that the abundance of *Erysipelatoclostridium* was nominally inversely associated with GERD risk (IVW method: $\beta_{b}$ = − 0.054, SE = 0.02, OR = 0.95, 95% CI: 0.90–0.99, *p* = 0.04), suggesting a possible protective signal for this taxon. Because neither the microbiota-wide association nor the mediation screen survived FDR correction, this result was treated as hypothesis-generating (Supplementary Table S15).

**Figure 5:**
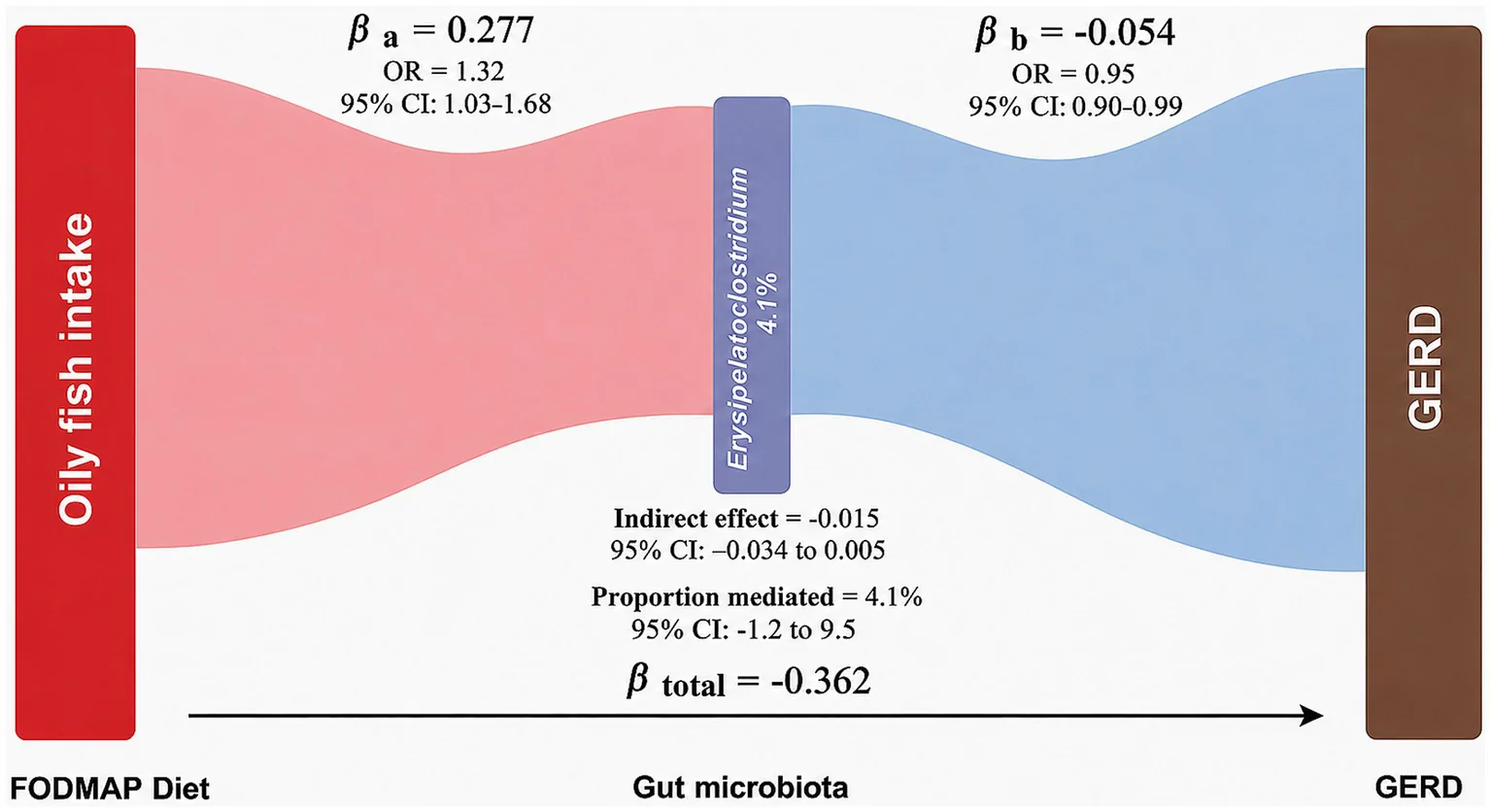
Exploratory mediation analysis of the association between FODMAP-related and GERD-relevant dietary items, gut microbiota, and GERD risk. Path coefficients (*βa* and *βb*) are presented as point estimates with their corresponding 95% confidence intervals shown in the figure. The indirect effect and proportion mediated are exploratory estimates, with 95% confidence intervals provided in the figure and Table 1. Because the confidence intervals for the indirect effect and mediated proportion crossed the null, these findings do not provide evidence for a confirmed mediation pathway.

By constructing a mediation model, we quantitatively decomposed the protective association of oily fish intake into direct and indirect components. The total protective effect of oily fish intake on GERD ( $\beta_{\text{total}}$ ) was −0.362. The indirect effect through *Erysipelatoclostridium* ( $\beta_{\text{indirect}}$ ) was −0.015 (calculated as 0.277 × −0.054; 95% CI, −0.034 to 0.005), accounting for 4.1% of the total effect (95% CI, −1.2 to 9.5%). The direct effect of oily fish intake on GERD ( $\beta_{\text{direct}}$ ) was −0.347, representing 95.9% of the total effect (Table 1; Figure 5). Because the indirect-effect and mediated-proportion CIs crossed the null, and because microbiota-wide and mediation-screening results did not survive FDR correction, this mediation result should be interpreted as a small and statistically imprecise hypothesis-generating signal rather than a confirmed microbial pathway.

**Table 1:** Mediator analysis results of FODMAP diet, gut microbiota and GERD, including Delta-method 95% confidence intervals for the indirect effect and mediated proportion.

FODMAP diet	Gut microbiota	Path a, $\beta_{a}$	Path b, $\beta_{b}$	Total effect $\beta_{\text{total}}$	Direct effect $\beta_{\text{direct}}$	Indirect effect $\beta_{\text{indirect}}$ (95% CI)	Proportion mediated, % (95% CI)	Interpretation
Oily fish	*Erysipelatoclostridium*	0.277	−0.054	−0.362	−0.347	−0.015 (−0.034 to 0.005)	4.1 (−1.2 to 9.5)	Exploratory; 95%CIs include the null value

These results indicate that the protective association of oily fish intake was predominantly attributable to non-microbiota pathways. The *Erysipelatoclostridium*-related indirect-effect estimate was directionally consistent with mediation but imprecise, with confidence intervals compatible with no mediation; it should therefore be interpreted as an exploratory biological clue rather than a dominant or statistically established mechanism (Detailed data in Supplementary Tables S13, S14).

### 3.4 Retrospective case–control study for clinical validation

To validate the MR findings, we conducted a retrospective case–control study including 525 GERD patients (mean age 51.8 ± 13.1 years, 47.2% male) and 525 healthy controls (mean age 50.6 ± 12.7 years, 47% male). No significant differences were observed between the two groups regarding age, sex, smoking status, alcohol consumption, or total energy intake (*p* > 0.05; Table 2).

**Table 2:** Baseline characteristics between GERD and control group.

Variables	GERD	Control	Statistic	*p*-value
Age (years, Mean ± SD)	51.8 ± 13.1	50.6 ± 12.7	t = 1.51	0.134
Male [n (%)]	248 (47.2)	245 (47.0)	*χ*^2^ = 0.68	0.409
BMI (kg/m^2^)	24.8 ± 3.9	24.6 ± 3.5	*t* = 0.86	0.390
Smokers [n (%)]	141 (26.9)	128 (24.3)	*χ^2^ = 0.85*	0.358
Alcohol drinkers [n (%)]	125 (23.8)	138 (27.6)	*χ*^2^ = 1.97	0.161
Total energy intake (kcal/day)	2018 ± 426	1988 ± 401	*t = 1.18*	0.239

Comparative analysis of dietary intake revealed that GERD patients consumed significantly higher amounts of tea, poultry, processed meat, and sweets, while their intake of cereal, cheese, fresh fruit, dried fruit, and oily fish was significantly lower than that of the control group (all *p* < 0.05; Table 3).

**Table 3:** Comparison of dietary intake characteristics between GERD and control group.

Dietary variables	GERD [Median (IQR)]	Control [Median (IQR)]	Z-value	*p*-value
Beer (cups/week)	3.6 (2.2, 5.6)	3.5(2.0, 5.5)	Z = 0.63	0.521
Biscuits/Sweets (servings/week)	4.8 (2.2, 7.0)	4.2 (2.0, 6.9)	Z = 2.71	0.007
Cheese (servings/week)	2.0 (1.0,2.4)	2.4 (1.2, 2.8)	Z = −5.82	<0.001
Coffee (cups/week)	10.2 (3.2, 14.6)	9.6 (4.1, 12.8)	Z = 1.29	0.198
Cooked vegetables (servings/week)	35.6 (20.2, 40.5)	36.2 (22.0, 41.2)	Z = −0.66	0.509
Crustaceans (servings/week)	5.0 (2.0, 6.6)	4.8 (2.2, 7.0)	Z = 0.93	0.351
Dried fruit (servings/week)	0.8 (0.2, 1.0)	0.9 (0.3, 1.0)	Z = −2.73	0.006
Eggs (servings/week)	7.2 (3.2, 9.2)	6.8 (2.8, 8.8)	Z = 1.46	0.144
Fresh fruit (servings/week)	7.8 (4.2, 9.6)	8.6 (4.6, 10.2)	Z = −3.18	0.002
Milk (cups/week)	28.8 (22.5, 35.0)	27.8 (22.2, 34.8)	Z = 1.75	0.081
Non-oily fish (servings/week)	8.5 (4.1, 12.0)	9.0 (4.8, 12.5)	Z = 1.40	0.160
Oily fish (servings/week)	6.0 (3.3, 8.1)	7.1 (3.2, 9.4)	Z = −4.34	<0.001
Pork/Beef/Lamb (servings/week)	15.8 (8.2, 20.6)	16.5 (10.5, 22.2)	Z = −1.60	0.204
Poultry (servings/week)	6.5 (4.5, 7.2)	5.2 (3.0, 6.8)	Z = 8.83	<0.001
Processed meat (servings/week)	2.3 (1.2, 2.8)	1.8 (1.0, 2.4)	Z = 7.28	<0.001
Red wine (cups/week)	2.2 (1.2, 2.4)	2.4 (1.0, 2.6)	Z = −1.55	0.121
Rice (servings/week)	34.5 (25.1, 40.2)	35.6 (23.5,38.5)	Z = −1.60	0.110
Salad vegetables (servings/week)	3.3 (1.5, 3.8)	2.8 (1.0, 3.5)	Z = 1.90	0.057
Tea (cups/week)	8.8 (3.4, 11.8)	7.9 (3.1, 11.1)	Z = 2.40	0.016
Wheat flour (servings/week)	6.8 (3.5, 9.8)	8.2 (4.1, 10.3)	Z = −4.90	<0.001

1 serving was defined as 50 g. 1 cup was defined as 50 mL.

Univariate logistic regression analysis demonstrated that higher intake of tea (OR = 1.05, 95% CI: 1.03–1.07), poultry (OR = 1.10, 95% CI: 1.04–1.16), processed meat (OR = 1.24, 95% CI: 1.05–1.43), and sweets (OR = 1.08, 95% CI: 1.04–1.12) was associated with an increased risk of GERD. Conversely, intake of cereal (OR = 0.92, 95% CI: 0.88–0.96), cheese (OR = 0.79, 95% CI: 0.72–0.87), fresh fruit (OR = 0.80, 95% CI: 0.68–0.92), dried fruit (OR = 0.76, 95% CI: 0.65–0.87), and oily fish (OR = 0.78, 95% CI: 0.65–0.91) was significantly associated with a reduced risk. After adjusting for potential confounders including age, sex, BMI, smoking, and alcohol history in multivariable models, these associations remained statistically significant (Table 4), maintaining high consistency with our Mendelian randomization findings.

**Table 4:** Univariable and multivariable logistic regression analyses of the associations between dietary intake factors and GERD.

Dietary Variables	Univariate		Multivariate	
	OR (95% CI)	*p*-value	OR (95% CI)	*p*-value
Beef/Pork/Lamb (per 1 serving/week)	1.08 (0.98, 1.18)	0.091	1.06 (0.96, 1.16)	0.127
Beer (per 1 cup/week)	0.89 (0.76, 1.02)	0.072	0.92 (0.80, 1.04)	0.081
Biscuits/sweets (per 1 serving/week)	1.08 (1.04, 1.12)	0.003	1.06 (1.02, 1.10)	0.011
Cheese (per 1 serving/week)	0.79 (0.72, 0.87)	<0.001	0.84 (0.76, 0.93)	<0.001
Coffee (per 1 serving/week)	1.11 (0.95, 1.16)	0.175	1.03 (0.89, 1.17)	0.211
Cooked vegetables (per 1 serving/week)	1.22 (0.92, 1.52)	0.125	1.18 (0.96,1.40)	0.167
Crustaceans (per 1 serving/week)	1.21 (0.88, 1.54)	0.228	1.18 (0.85, 1.51)	0.324
Dried fruit (per 1 serving/week)	0.76 (0.65, 0.87)	<0.001	0.80 (0.70, 0.90)	<0.001
Egg (per 1 serving/week)	1.03 (0.95, 1.11)	0.121	1.01 (0.90,1,12)	0.252
Fresh fruit (per 1 serving/week)	0.80 (0.68, 0.92)	0.002	0.88 (0.78, 0.98)	0.012
Milk (per 1 cup/week)	1.12 (0.98, 1.26)	0.095	1.08 (0.92, 1.24)	0.128
Non-oily fish (per 1 serving/week)	1.15 (0.98, 1.32)	0.132	1.12 (0.88, 1.36)	0.238
Oily fish (per 1 serving/week)	0.78 (0.65, 0.91)	<0.001	0.85 (0.72, 0.98)	0.008
Poultry (per 1 serving/week)	1.10 (1.04, 1.16)	<0.001	1.06 (1.03, 1.09)	<0.001
Processed meat (per 1 serving/week)	1.24 (1.05, 1.43)	<0.001	1.19 (1.02, 1,36)	<0.001
Red wine (per 1 cup/week)	1.01 (0.92, 1.10)	0.256	0.98 (0.88, 1.08)	0.332
Rice (per 1 serving/week)	0.96 (0.90, 1.02)	0.072	0.95 (0.88,1.02)	0.098
Salad vegetables (per 1 serving/week)	0.88 (0.74, 1.02)	0.058	0.84 (0.67, 1.01)	0.061
Tea (per 1 serving/week)	1.05 (1.03, 1.07)	<0.001	1.04 (1.02, 1.06)	<0.001
Wheat (per 1 serving/week)	0.92 (0.88, 0.96)	<0.001	0.94 (0.90, 0.98)	0.015

For salad vegetables, the clinical estimate was directionally concordant with the protective MR estimate but did not reach statistical significance after multivariable adjustment; thus, it was interpreted as a directionally consistent but statistically nonsignificant clinical signal rather than as a confirmed clinical association.

### 3.5 Conclusion

For the first time, this study systematically investigated the causal relationships between FODMAP-related dietary patterns, gut microbiota, and GERD using a robust two-step, two-sample MR framework. By integrating evidence from large-scale genome-wide association study (GWAS) data with a retrospective case–control study (*n* = 525 cases vs. *N* = 525 controls), our findings established the following key conclusions:

Causal Dietary Links: Eight dietary habits were causally associated with GERD risk. Specifically, higher intake of fresh fruit, dried fruit, salad/raw vegetables, cheese, cereal, and oily fish exerted significant protective effects, whereas increased consumption of tea and poultry appeared to elevate GERD susceptibility.

Microbial Identification: Through two-step MR analysis, we identified 15 gut microbiota taxa that were nominally associated with GERD risk. However, these associations did not survive BH-FDR correction across 211 taxa and should therefore be considered exploratory candidate signals rather than confirmed microbial causal determinants.

Mediating Mechanisms: Mediation analysis suggested a small exploratory indirect effect linking oily fish intake, Erysipelatoclostridium, and GERD. This pathway accounted for approximately 4.1% of the total effect, but its 95% CI crossed the null, indicating uncertainty around the mediated proportion. Thus, the majority of the protective association was attributed to direct pathways, and the mediation signal should be viewed as hypothesis-generating.

Collectively, this study provides integrated genetic and clinical evidence supporting the relevance of specific dietary components and gut microbial taxa in GERD pathogenesis, while emphasizing that microbiota-mediated effects, particularly the *Erysipelatoclostridium* signal, remain exploratory and require further validation before being considered clinical targets.

## 4 Discussion

The etiology of GERD is multifactorial, with dietary patterns increasingly recognized as modifiable contributors. Our MR study provides FDR-supported genetic evidence for selected FODMAP-related dietary intakes in GERD risk, whereas the gut microbiota and mediation findings should be interpreted as nominal exploratory signals after correction for multiple testing. Importantly, the oily fish-*Erysipelatoclostridium*-GERD finding should be viewed as a small and statistically imprecise hypothesis-generating mediation signal, with a 95% CI crossing the null, rather than as confirmation of a major biological pathway.

### 4.1 FODMAP diet and specific foods: a multidimensional perspective beyond simple mechanisms

Although the pathophysiological cascade of “FODMAP fermentation → gas production → increased intra-abdominal pressure → transient lower esophageal sphincter relaxations (TLESRs)” provides a compelling explanation for GERD symptoms, particularly in patients with comorbid functional bowel disorders (6, 9), relying on it as the sole driver for GERD may be oversimplified. Our two-sample MR analysis reveals a more nuanced landscape: certain foods that are neither high-FODMAP nor strictly low-FODMAP (e.g., fresh fruits and whole cereals) exhibited significant protective effects against GERD. This finding challenges the traditional paradigm of predicting GERD risk solely based on carbohydrate fermentation potential, suggesting that the “food matrix” effect may transcend individual nutrient components. We hypothesize that these protective associations stem from the synergistic actions of dietary fiber, phytochemicals, and antioxidants. For instance, soluble fiber may not only reduce gas production by modulating gut microbiota but also directly strengthen the anti-reflux barrier by increasing the resting pressure of the lower esophageal sphincter (LES) (11). Simultaneously, the anti-inflammatory properties of polyphenols, on the other hand, may desensitize the esophageal mucosa to refluxate (25).

Notably, differences between MR estimates and clinical observations should be interpreted in light of the distinct estimands and exposure measurements. For salad/raw vegetables, the MR estimate reflects genetically proxied long-term intake tendency in UK Biobank, whereas the clinical estimate was based on retrospective FFQ data over the preceding year and was directionally concordant but statistically nonsignificant. This partial discrepancy may reflect limited clinical power, recall and portion-size error, symptom-driven dietary modification, and cultural differences in vegetable preparation; in Chinese dietary settings, vegetables are commonly consumed cooked or stir-fried, and FFQ-defined salad/raw vegetable intake may not map directly onto the UK Biobank salad/raw vegetable trait. Patients with reflux symptoms may also avoid cold, raw, or high-fiber vegetables because of bloating, throat irritation, or perceived symptom triggering, introducing reverse causality. Consistent with GERD guideline recommendations, these foods should therefore be considered within an individualized trigger-food framework rather than interpreted as universally harmful or protective (26–28).

The tea finding should be interpreted within the MR estimand. The estimate reflects genetically predicted long-term tea intake, not acute caffeine exposure, methylxanthine dose, beverage temperature, or gastric distension. Thus, the positive tea-GERD association is better framed as a long-term beverage-pattern signal. Potential explanations include tea type and concentration, preparation method, drinking temperature, additives, correlated diet and lifestyle, and interindividual caffeine metabolism. Methylxanthine-related lower esophageal sphincter relaxation remains plausible, but this MR result alone cannot establish it as the mechanism. This cautious interpretation is supported by mixed observational evidence, prospective beverage-symptom data, recent MR evidence on tea and non-malignant digestive diseases, and American College of Gastroenterology guidance favoring individualized trigger-food avoidance rather than universal restriction of tea or caffeinated beverages (26, 29–31).

Furthermore, processed meats and sweets were identified as risk factors in the case–control study, but were not supported by the MR analysis. We propose that this discrepancy stems from “exogenous processing” rather than long-term biological causality. Specifically, high-temperature processing (e.g., grilling, frying) generates thermal reaction products such as polycyclic aromatic hydrocarbons (PAHs) and heterocyclic amines (HCAs), which may directly irritate the esophageal mucosa or trigger systemic inflammation (32). Additionally, high concentrations of nitrites and phosphates in processed meats, along with artificial sweeteners and emulsifiers in sweets, may induce reflux by compromising gut barrier function or relaxing the LES (33, 34). These risk factors are often inextricably linked with specific lifestyle patterns (e.g., fast-food culture). Because MR captures genetically determined long-term intake tendencies rather than short-term physiological effects driven by processing methods or additives, our findings highlight the critical importance of distinguishing the “food matrix” from actual “dietary exposure” in causal inference.

### 4.2 Exploratory microbiota-related mechanisms in GERD: from metabolites to physiological dysfunction

The “Gut-Esophagus Axis” hypothesis posits that dysbiosis of the gut microbiota can remotely regulate esophageal motor function and visceral hypersensitivity, thereby contributing to GERD development, through influencing neuroendocrine signals, immune modulation, and metabolic products. In recent years, an increasing number of clinical studies utilizing high-throughput sequencing technologies have revealed significant differences in the composition and function of oral, esophageal, and gut microbiota between GERD patients and healthy individuals (35, 36). For instance, multiple studies have reported an increased proportion of Gram-negative bacteria in the esophagus or feces of GERD patients, particularly the abnormal enrichment of Streptococcus, Prevotella, and Neisseria genera (37).

Our MR analysis identified 15 microbial taxa with nominal associations with GERD, offering exploratory evidence relevant to the gut-esophagus axis rather than confirmatory causal proof. Specifically, *Bifidobacteriaceae*, *Ruminococcus2*, *LachnospiraceaeUCG004*, and *Butyrivibrio* exhibited nominal protective signals, aligning with recent paradigms of mucosal barrier integrity (13).

Mechanistically, Ruminococcus2 and *Butyrivibrio* are biologically plausible butyrate-producing commensals (38). Butyrate not only reinforces the physical barrier by upregulating tight junction proteins (e.g., ZO − 1 and Occludin) to prevent endotoxemia, thereby reducing systemic levels of TNF − *α* and IL − 1β (39), but also modulates gastrointestinal motility via vagal pathways to decrease the frequency of transient lower esophageal sphincter relaxations (TLESRs). Furthermore, the protective role of *Bifidobacteriaceae* likely stems from its metabolic flexibility; research indicates it can provide substrates for other butyrate-producers through “cross-feeding” interactions, collectively sustaining a low-inflammatory milieu and desensitizing the esophageal mucosa to acid insult (40, 41). These mechanisms remain biologically plausible explanations rather than confirmed GERD mechanisms in the present MR analysis.

Conversely, *Bacteroidia, Mollicutes, Negativicutes, Haemophilus, Selenomonadales,* and *Tenericutes* showed nominal risk-associated signals for GERD rather than established pathogenic-driver effects. The lipopolysaccharide (LPS)-mediated immune activation represents a primary pathway. While *Bacteroidia* can be symbiotic, its overgrowth—alongside other Gram-negative taxa like *Mollicutes* and *Negativicutes*—leads to elevated LPS loads. This excess LPS activates Toll-like receptors (TLR2 and TLR4) on esophageal epithelial cells, triggering the NF − κB signaling pathway and a subsequent “storm” of pro-inflammatory cytokines (e.g., IL − 6, IL − 8, and TNF − α), which compromises mucosal defense (42–44).

Importantly, LPS has been shown to upregulate inducible nitric oxide synthase (iNOS), leading to increased nitric oxide production that promotes inappropriate LES relaxation, thus exacerbating reflux. Additionally, metabolites from taxa such as *Haemophilus* and *Selenomonadales*, particularly hydrogen sulfide (H_2_S), can directly inhibit mitochondrial respiration and butyrate oxidation in epithelial cells. This results in an “epithelial energy crisis” and severe barrier dysfunction (45). These metabolic perturbations may further induce visceral hypersensitivity by suppressing intestinal hormones like GLP − 1 or exerting genotoxicity, providing testable hypotheses for GERD pathogenesis rather than confirmed causal drivers in this study (46, 47).

### 4.3 Anti-inflammatory drive and homeostatic restoration: multidimensional mechanisms of oily fish and *Erysipelatoclostridium* in GERD

Our MR and mediation analysis suggested that the protective association between oily fish and GERD was driven predominantly by direct pathways, with only a small exploratory contribution from microbial mediation. Specifically, the point estimate suggested that *Erysipelatoclostridium* accounted for approximately 4.1% of the total oily fish-GERD association ( $\beta_{\text{indirect}}$ = − 0.015), but the 95% CIs for both the indirect effect (−0.034 to 0.005) and mediated proportion (−1.2 to 9.5%) crossed the null. This pattern indicates a biologically plausible but statistically imprecise indirect signal rather than a pivotal microbial link within the diet-microbiota-reflux pathway.

#### 4.3.1 Microbial dimension: *Erysipelatoclostridium* as a sentinel of homeostasis

The association of *Erysipelatoclostridium* with GERD protection should be interpreted cautiously. Evidence from other inflammatory or functional gastrointestinal contexts suggests that this genus may track with microbial homeostasis, but direct functional evidence in GERD is lacking (48, 49). Belonging to the *Erysipelotrichaceae* family within the Firmicutes phylum, *Erysipelatoclostridium* has been linked to short-chain fatty acid (SCFA)-related pathways that may influence mucosal barrier function, immune regulation, and gut-brain-microbiota signaling. Clinical interventions, such as those involving STW 5-II, have been associated with *Erysipelatoclostridium* enrichment while suppressing pathogenic *Enterobacteriaceae* (50). However, given the modest mediated proportion, the confidence interval crossing the null, and the limited host genetic heritability of gut microbial abundance, our finding should be interpreted as a hypothesis-generating signal. Future experimental and longitudinal studies are required to determine whether changes in this genus have a direct functional role in GERD-related mucosal defense or motility.

#### 4.3.2 Molecular perspective: direct defense and active resolution mechanisms of fish oil

The direct effects of fish oil—accounting for the majority of the total effect—exhibit more immediate and robust molecular actions. The primary mechanism involves the “defensive clearance” mediated by the Nrf2 pathway. Upon acid-induced oxidative stress in the esophageal epithelium, n − 3 PUFAs activate Nrf2 as a “molecular switch” to clear reactive oxygen species (ROS) and block NLRP3 inflammasome-mediated pyroptosis. This specific inhibition of the NLRP3/Caspase−1 axis constitutes the critical final defense against the collapse of the esophageal epithelial barrier (51).

Concurrently, fish oil facilitates “active repair” via n-3PUFA-derived metabolites (such as Resolvins and Protectins). Unlike passive anti-inflammation, n − 3 PUFA-derived SPMs actively initiate mucosal resolution and healing (52). This sequential process—spanning from oxidative stress defense and pyroptosis inhibition to active inflammation resolution—explains the potent independent protective capacity of oily fish against GERD. Collectively, oily fish establishes a comprehensive defense system by integrating intracellular direct repair with the restoration of intestinal microbial homeostasis.

### 4.4 Triangulating evidence: robustness, limitations, and causal boundaries

The core value of this study lies in the construction of a methodological triangle, integrating “MR-based causal inference” with “observational epidemiological validation.” By leveraging genetic variants—randomly assigned during gametogenesis—as IVs, our analysis not only circumvents common confounders (such as lifestyle and socioeconomic status) and reverse causality bias (53), but also cross-validates genetic evidence with real-world clinical phenotypic data through the “diet–microbiota–GERD” mediation framework. This dual-validation strategy enhances the biological plausibility of the causal chain and confirms the independent association of dietary factors within complex real-world environments, effectively bridging the gap where genetic studies often lack clinical granularity.

Despite this rigorous design, the results must be interpreted in light of inherent limitations. Foremost is the persistent challenge of “horizontal pleiotropy” in MR analysis—the risk that genetic variants may influence GERD via pathways independent of the target exposure, such as BMI or systemic inflammation. While we implemented multiple sensitivity analyses, including MR − Egger and MR − PRESSO, to screen for such effects (54, 55), the possibility of residual pleiotropy remains a concern within the polygenic architecture of complex diseases.

Furthermore, constraints in data characteristics directly influence the precision of our effect estimates. Given that the host heritability of microbial abundance is generally low, the proportion of variance explained by current GWAS remains limited (56–58). This potentially introduces weak-instrument bias and reduces the statistical power of microbiome MR mediation analyses. In addition, after BH-FDR correction across 211 microbial taxa, the microbiota associations in this study did not remain statistically significant. Such FDR-nonsignificant but nominally consistent signals can still be useful for prioritizing biological hypotheses in exploratory MR research, especially when supported by sensitivity analyses and clinical plausibility, but they should not be presented as definitive causal evidence (59). In the same vein, the 4.1% *Erysipelatoclostridium*-mediated proportion had a 95% CI that crossed the null and should therefore be interpreted as a small, imprecise exploratory signal rather than evidence of a dominant biological pathway.

A major limitation is the high MR-Steiger exclusion rate in the diet-to-microbiota step: 129,813 of 319,371 harmonized SNP–taxon instrument records (40.6%) did not support the assumed diet-to-microbiota direction. This substantial attrition indicates that many candidate instrument records showed insufficient evidence for the hypothesized directionality, potentially because the estimated variance explained was greater for the microbial trait than for the dietary exposure, or because of differences in measurement precision, phenotype definition, and GWAS characteristics. Because this step is critical to the mediation framework, the high exclusion rate limits confidence in the robustness and directional interpretation of the proposed pathway and highlights sensitivity to exposure measurement error, differences in GWAS populations, and conditional instrument strength. Therefore, the surviving oily fish–*Erysipelatoclostridium* association should be considered an exploratory finding requiring replication using improved dietary and microbiome instruments and complementary directionality assessments.

Simultaneously, measurement errors inherent in FFQ-based dietary assessments, coupled with the retrospective nature of clinical data, may introduce residual confounding (60). The FFQ assessed usual intake over the previous year and may therefore be affected by long-term recall bias, portion-size estimation error, and limited food-list granularity. Although the FFQ used in this cohort had been evaluated against repeated 24-h dietary recalls in a Fujian population, no FODMAP-specific recovery biomarker was available to objectively validate fermentable carbohydrate intake at the individual level (61). Looking ahead, future explorations should transcend mere sample size expansion. Instead, emphasis should be placed on incorporating objective biomarkers (e.g., metabolomics) and integrating metagenomic data to capture “invisible” but functionally critical metabolic pathways that possess low heritability. Only through such deep multi-omic integration can we more accurately delineate the full landscape of dietary-microbial interventions in the prevention and management of GERD. Future validation should incorporate prospective food records, repeated 24-h recalls, image-assisted portion-size estimation, FODMAP composition databases, and objective metabolomic or metagenomic biomarkers.

Another important limitation concerns population generalizability. The genetic evidence was derived mainly from European ancestry GWAS datasets, whereas the clinical validation cohort consisted of patients and controls from Fujian, China. Although this design allowed us to triangulate genetic causal inference with an independent real-world phenotype, ancestry differences may limit the direct transferability of the MR effect estimates to Chinese populations. Future studies using large-scale East Asian dietary and GERD GWAS resources will be required to confirm whether these genetic associations are consistent across ancestries. Phenotypic agreement in the Fujian cohort cannot establish the validity or transportability of European-derived genetic instruments in East Asian populations, because linkage-disequilibrium patterns, allele frequencies, dietary exposures, and microbiome composition differ across ancestries. This represents a structural limitation of the triangulation design that cannot be fully addressed by defining the clinical cohort as phenotypic validation. Moreover, the clinical cohort was drawn from a single institution in Fujian and evaluated within a single-center research framework, limiting geographic, dietary, referral-pattern, and procedural representativeness within China and increasing the possibility of site-specific selection or measurement effects.

Overall, the microbiota-related and mediation findings should be interpreted as hypothesis-generating evidence rather than definitive evidence of a biological mechanism. In particular, the oily fish–*Erysipelatoclostridium*–GERD association represents a small and statistically imprecise exploratory signal that requires independent replication before any causal interpretation or clinical implication can be considered.

## 5 Conclusion and future perspectives

By integrating MR causal inference with clinical epidemiological validation, this study provides an integrated assessment of the relationships between dietary factors, gut microbiota, and GERD. Our core findings suggest that while selected gut taxa may be involved in GERD susceptibility, the protective association of oily fish represents a dietary association with GERD risk that is not fully explained by the exploratory microbiota-related findings. The *Erysipelatoclostridium* signal identifies a possible but statistically imprecise microbiota-related hypothesis for future study, and should not be considered a validated clinical target at this stage. These findings highlight the complexity of diet–microbiota–GERD relationships and support a multidimensional, stratified approach to future dietary research.

Building upon these insights, future research should move from correlation validation toward deeper biological characterization and clinical translation. A primary objective is to use multi-omic integration, longitudinal cohorts, and experimental models to investigate whether candidate taxa such as *Erysipelatoclostridium* are biologically relevant to GERD-related processes, including esophageal mucosal barrier regulation and motility, while recognizing the current statistical power constraints of microbial IVs. Clinical translation should proceed cautiously through stratified randomized controlled trials (RCTs) based on host phenotype, diet, and baseline microbial signatures. Such studies will be essential before microbiota-targeted or food-derived interventions can be translated into clinically actionable precision nutrition prescriptions for GERD.

## Data Availability

The original contributions presented in the study are included in the article/Supplementary material, further inquiries can be directed to the corresponding author.

## Footnotes

1
https://mibiogen.gcc.rug.nl/
