# Supplementary material for: FODMAP intake, gut microbiota, and gastroesophageal reflux disease: a triangulated study of Mendelian randomization and clinical validation
Source: Front Nutr. 2026 Aug 26;13:1847736. doi: 10.3389/fnut.2026.1847736 (PMC13553803; doi:10.3389/fnut.2026.1847736)
Supplement: Supplementary file 1 [file Data_Sheet_1.pdf]

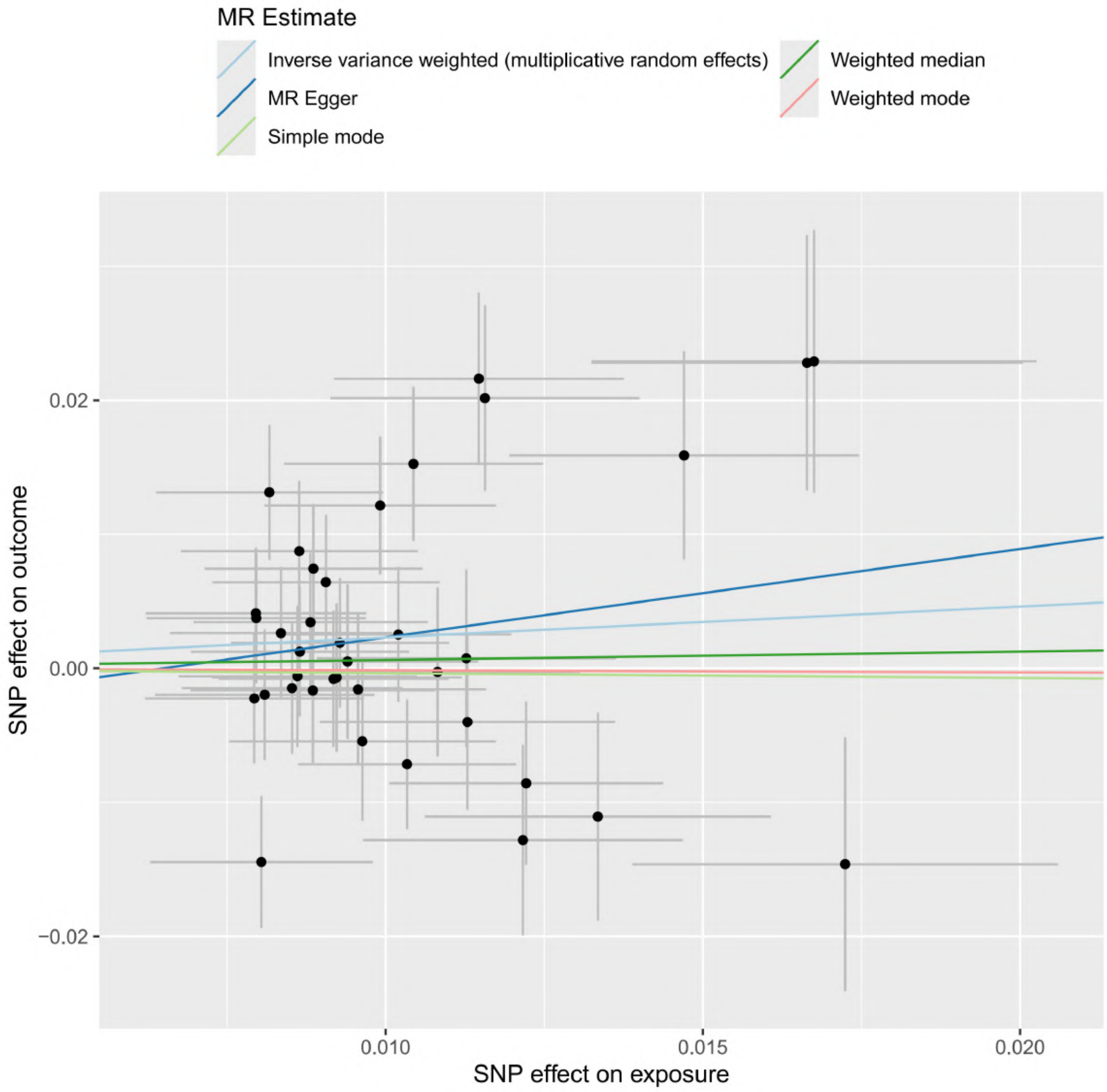

Supplementary Fig. 1 Scatter plot of the causal effect of beef intake on GERD

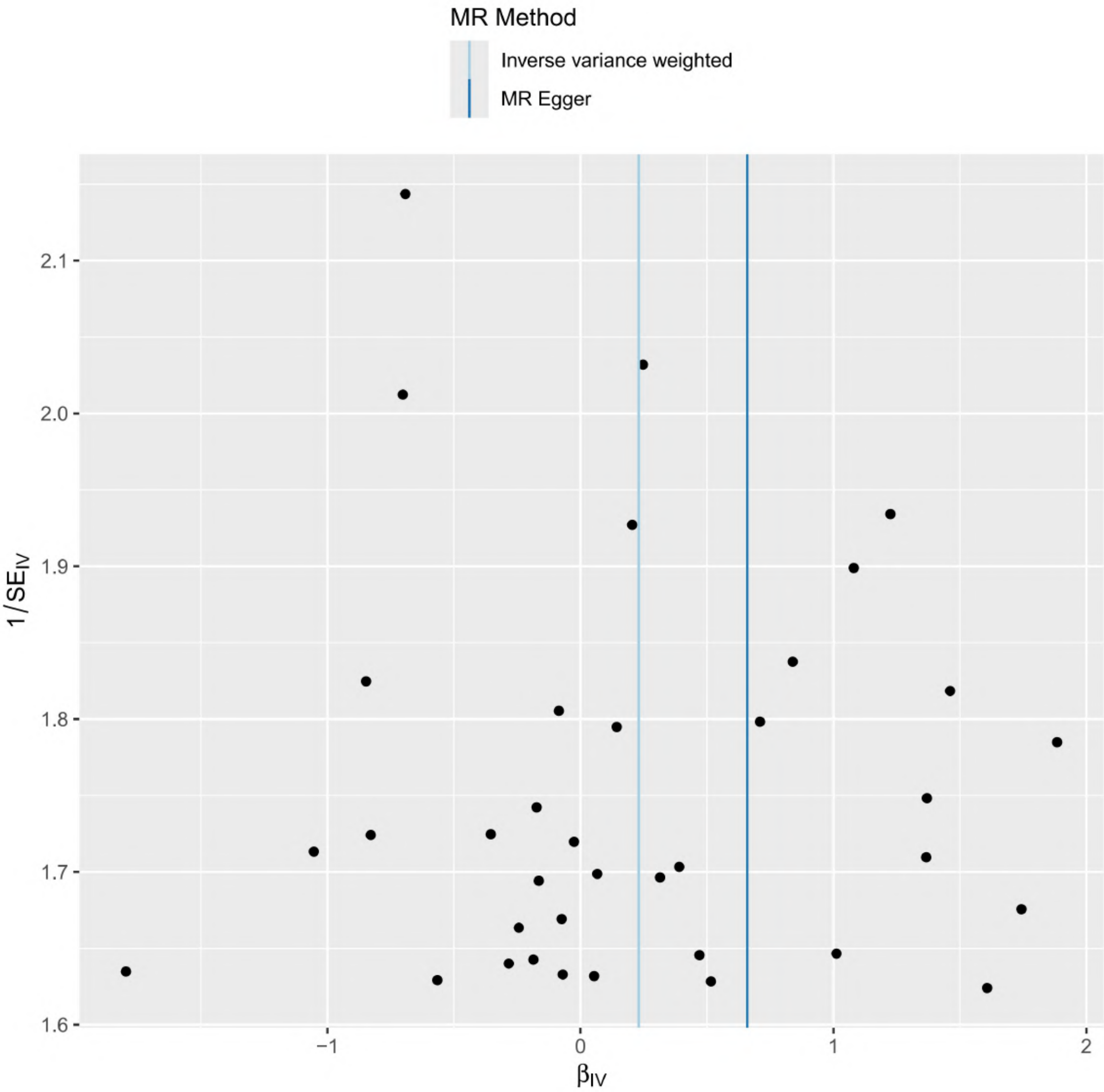

Supplementary Fig. 2 Funnel plot of the causal effect of beef intake on GERD

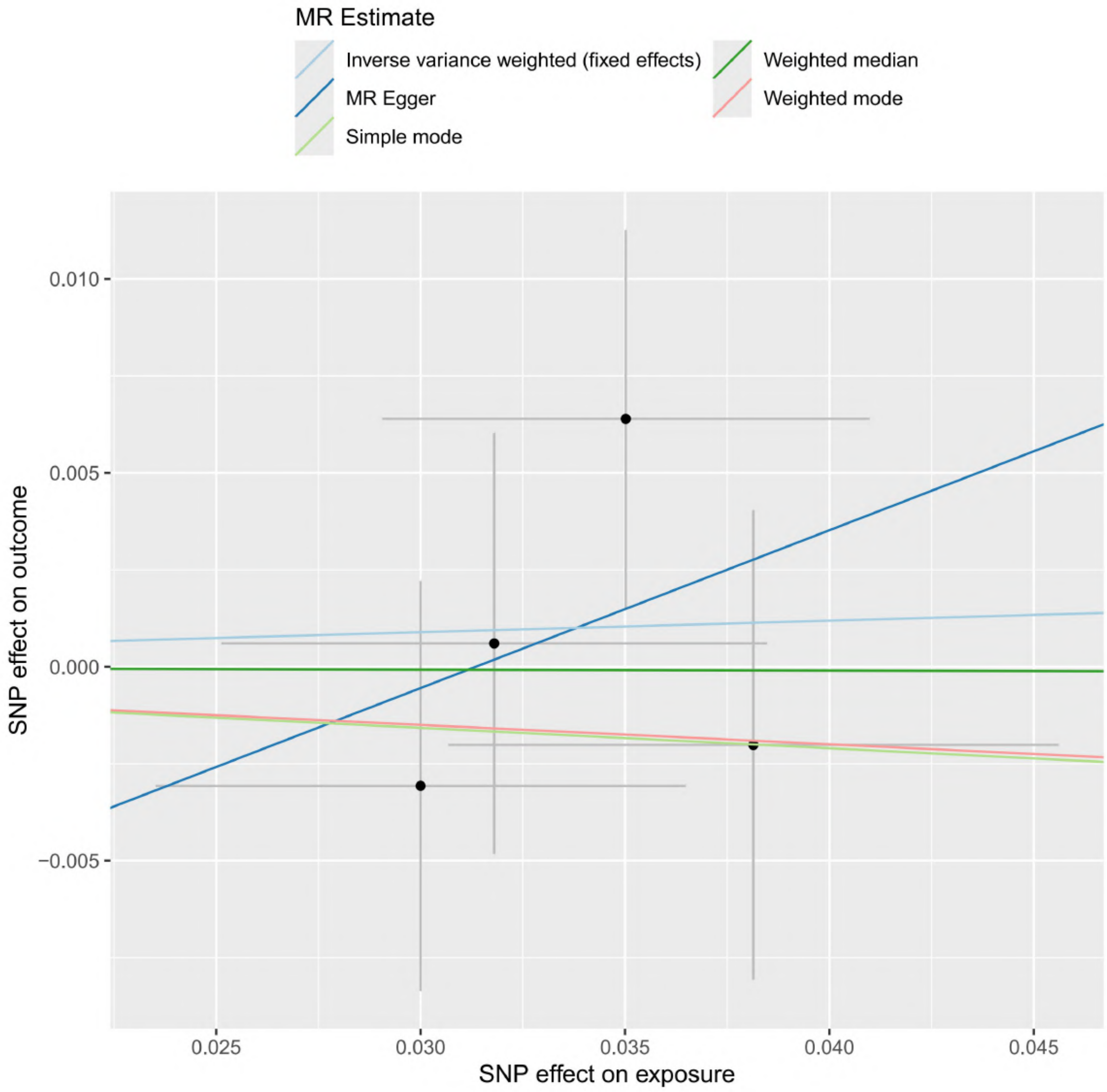

Supplementary Fig. 3 Scatter plot of the causal effect of banana intake on GERD

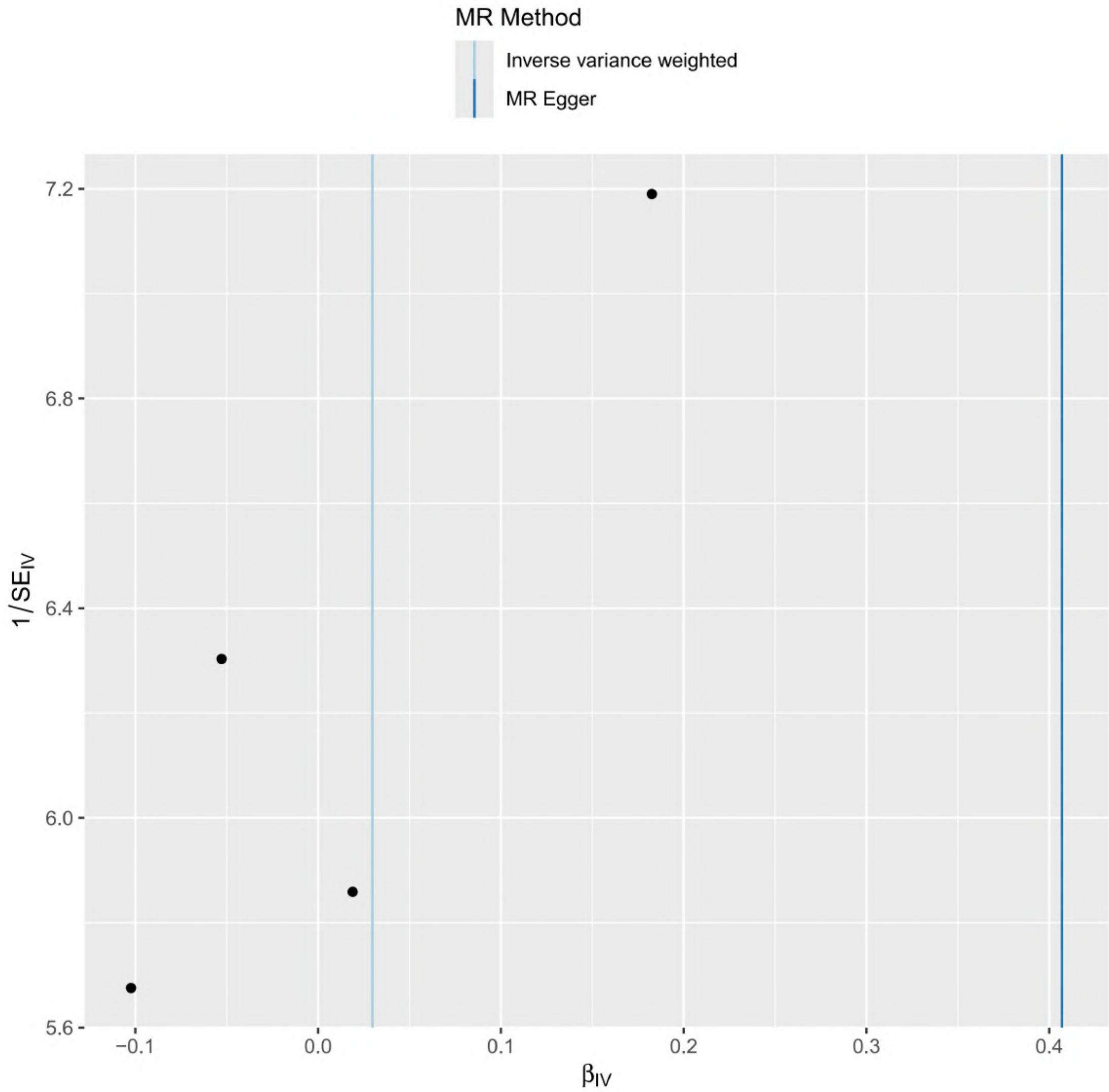

Supplementary Fig. 4 Funnel plot of the causal effect of banana intake on GERD

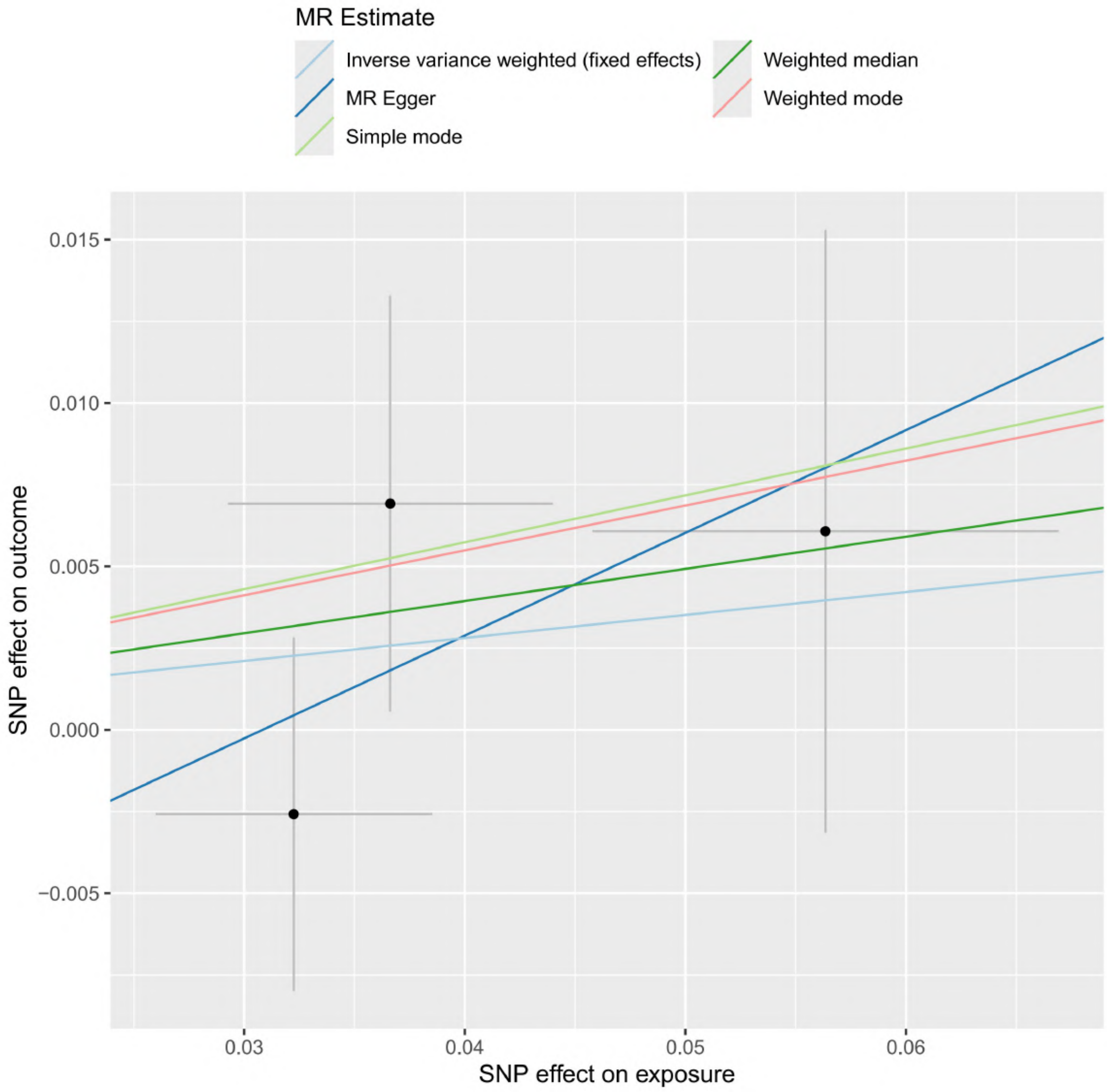

Supplementary Fig. 5 Scatter plot of the causal effect of Broccoli intake on GERD

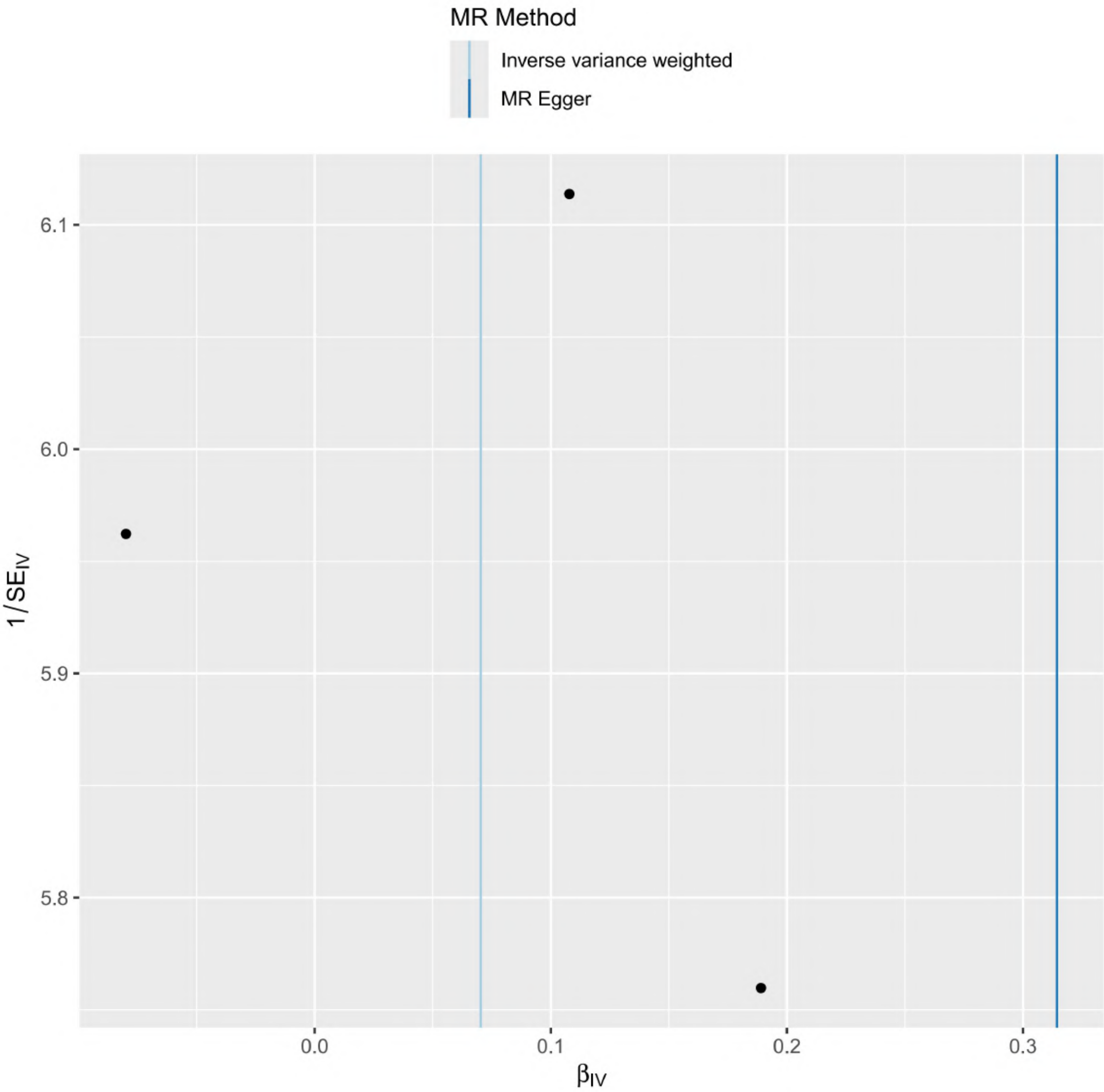

Supplementary Fig. 6 Funnel plot of the causal effect of Broccoli intake on GERD

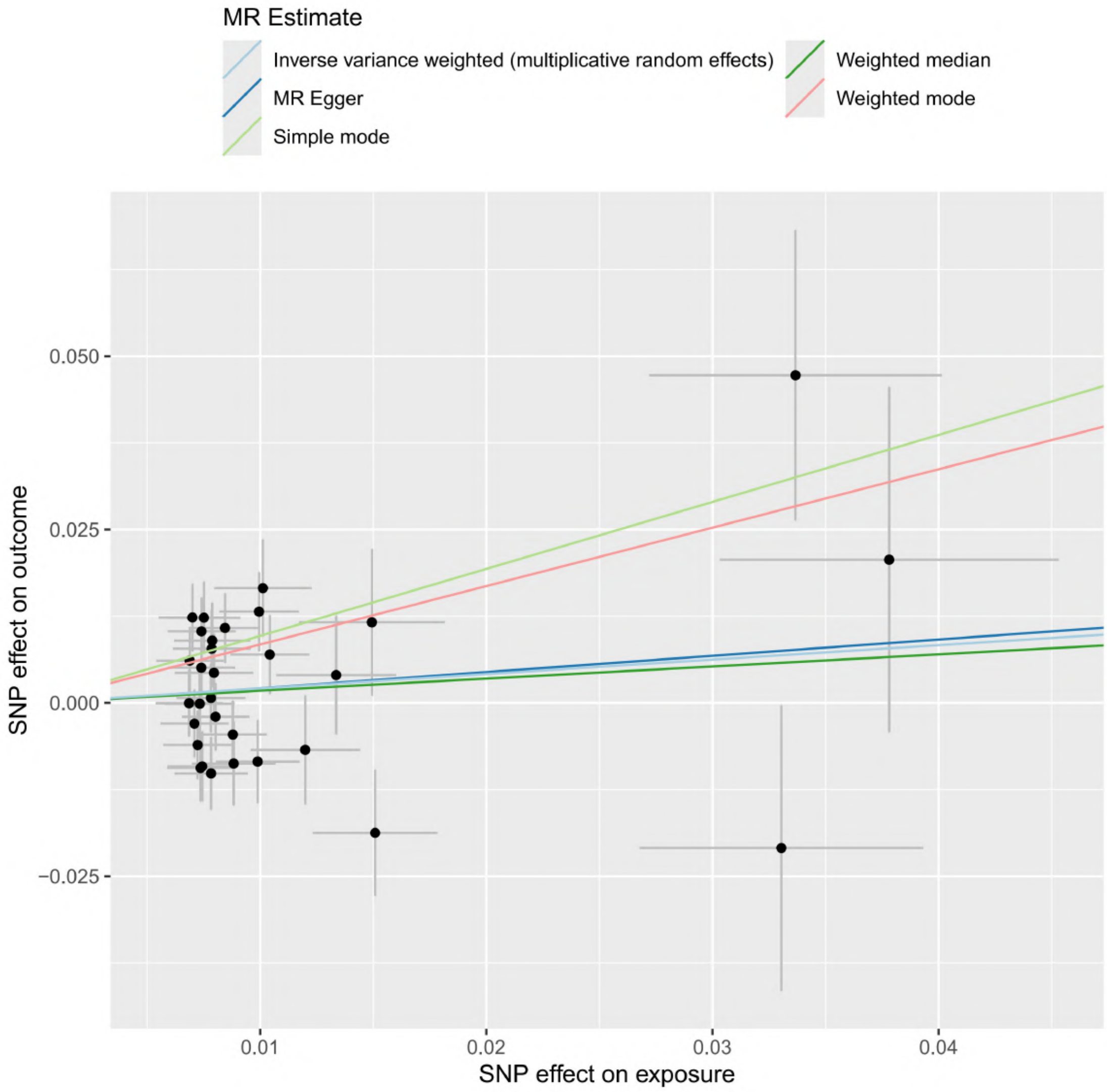

Supplementary Fig. 7 Scatter plot of the causal effect of pork intake on GERD

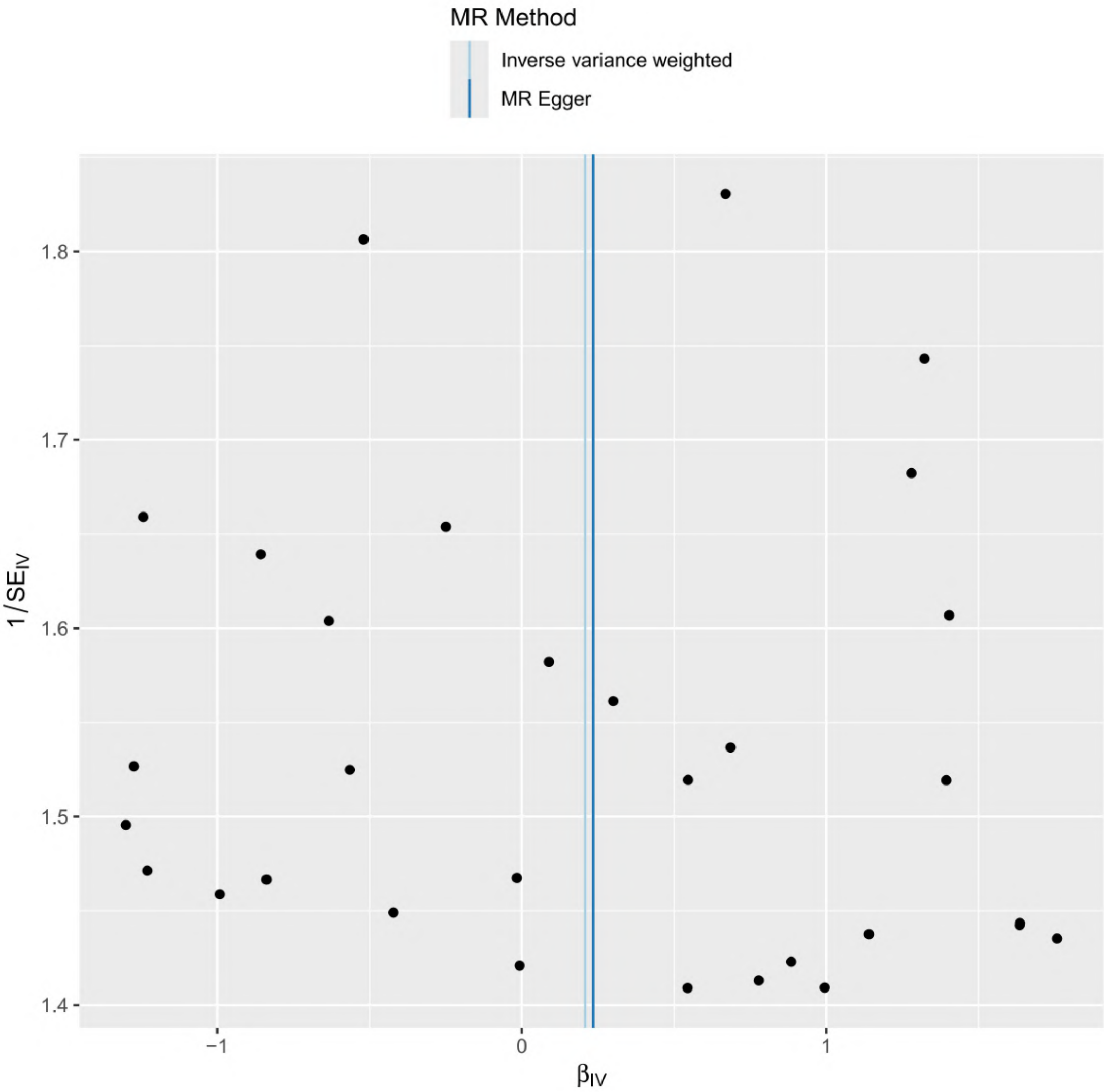

Supplementary Fig. 8 Funnel plot of the causal effect of pork intake on GERD

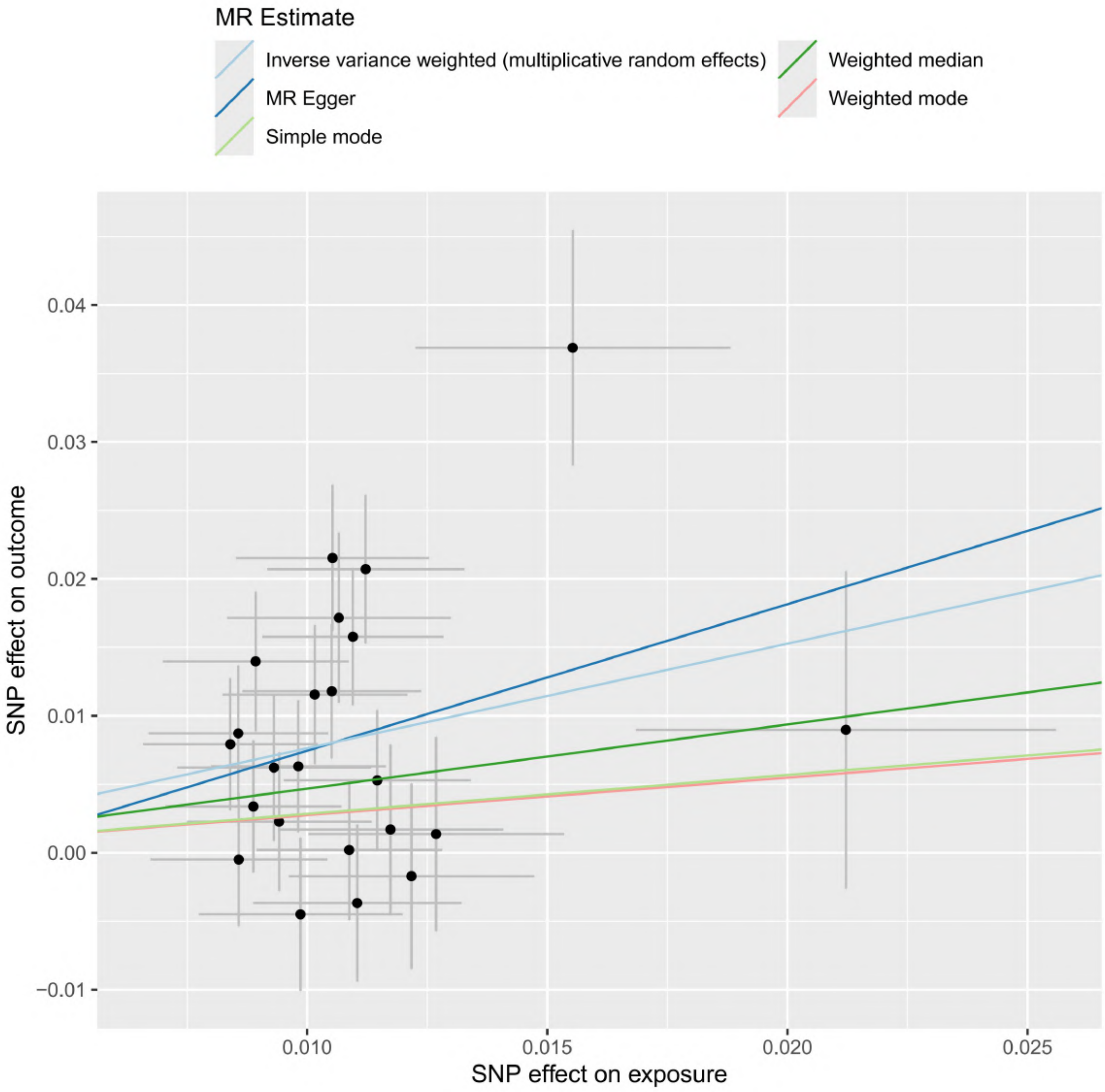

Supplementary Fig. 9 Scatter plot of the causal effect of poultry intake on GERD

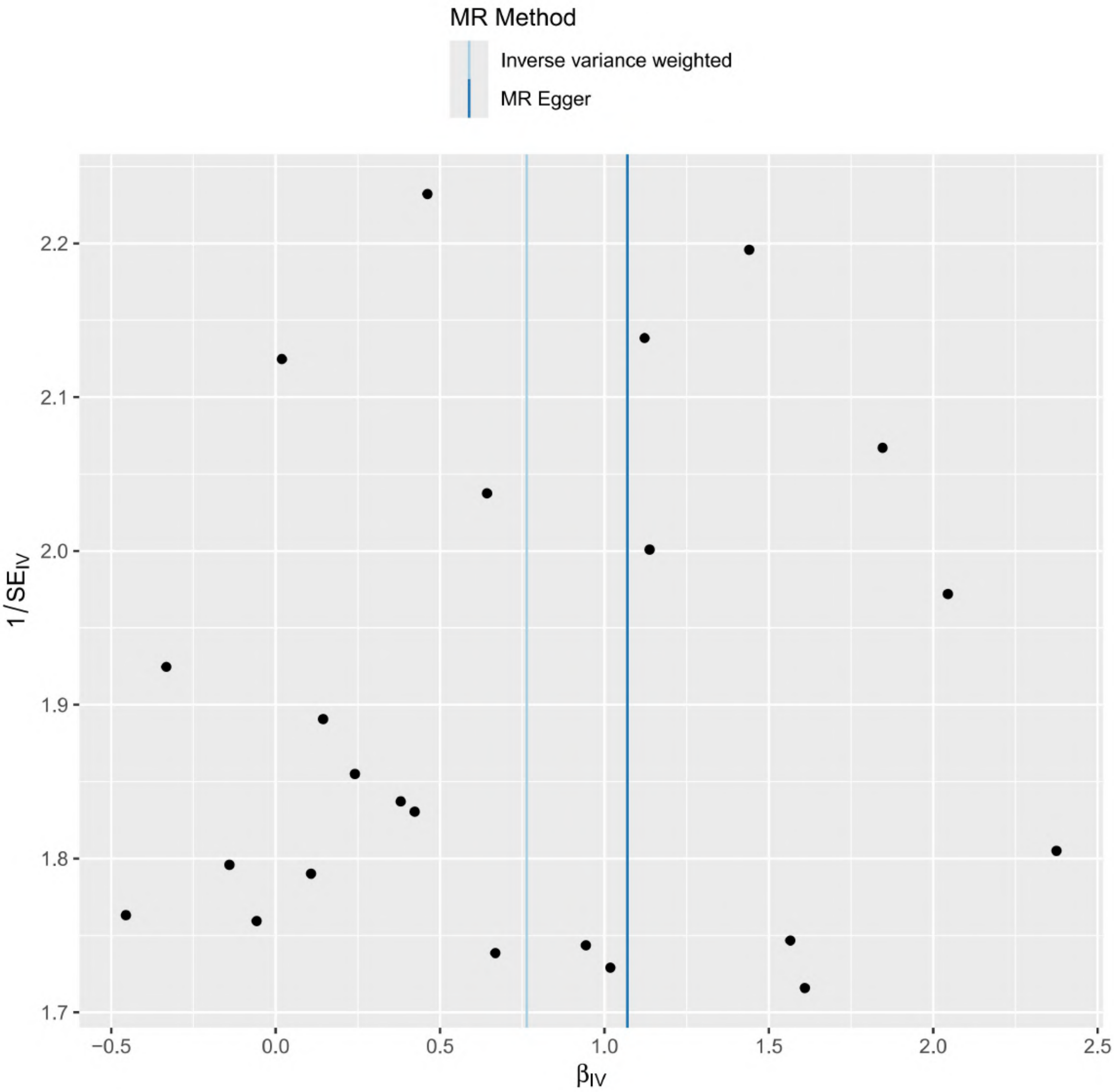

Supplementary Fig. 10 Funnel plot of the causal effect of poultry intake on GERD

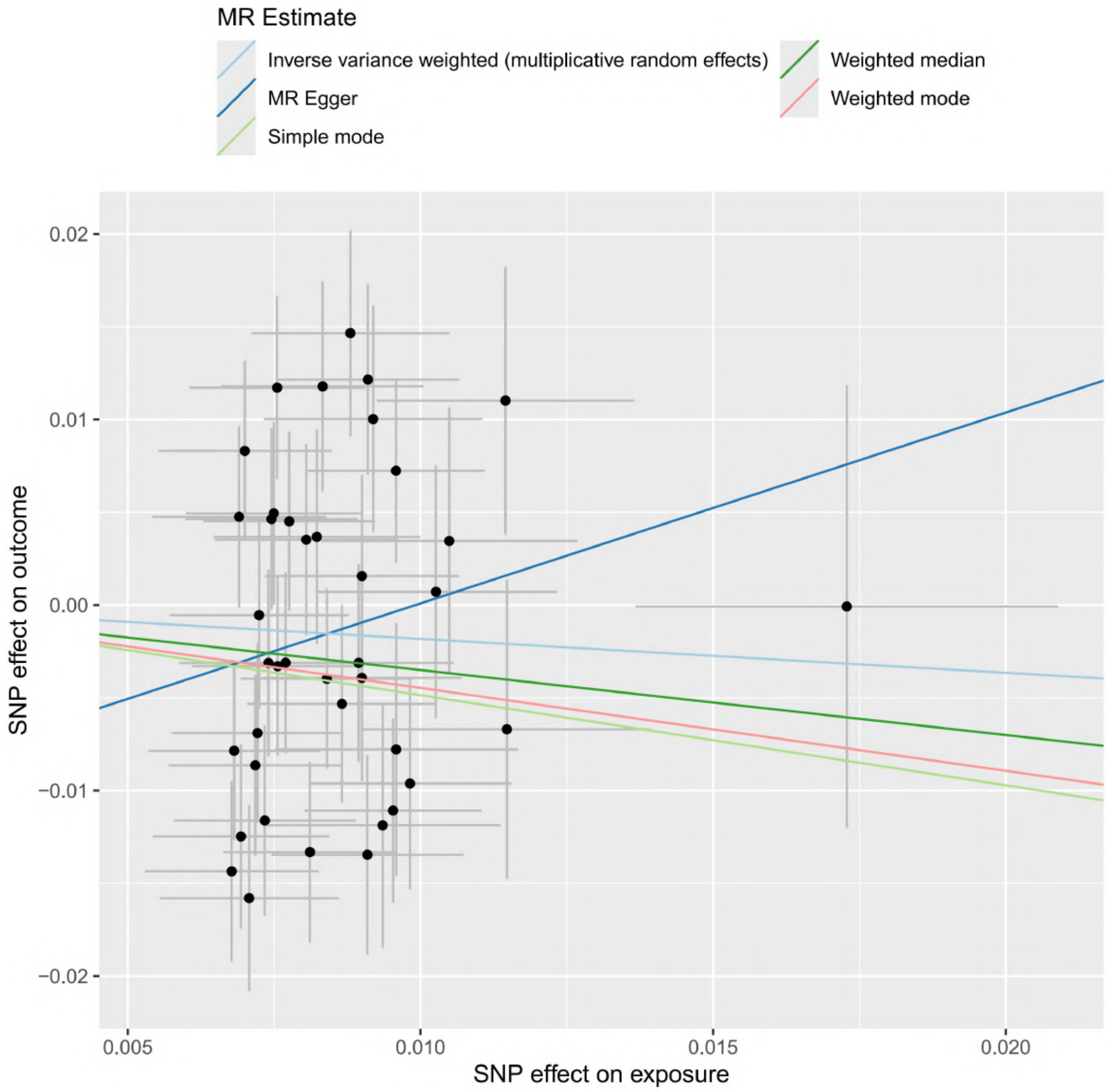

Supplementary Fig. 11 Scatter plot of the causal effect of Lamb/mutton intake on GERD

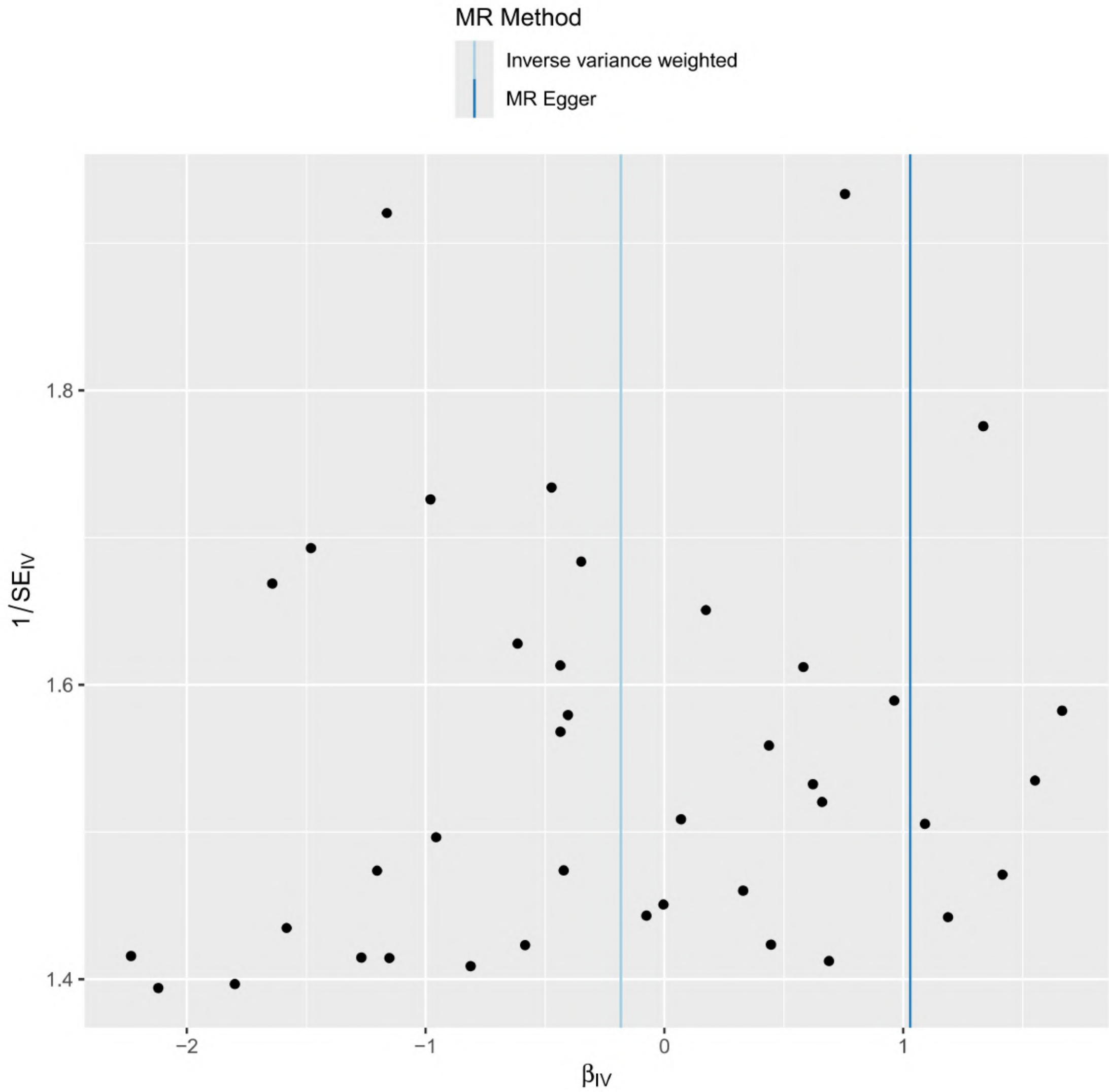

Supplementary Fig. 12 Funnel plot of the causal effect of Lamb/mutton intake on GERD

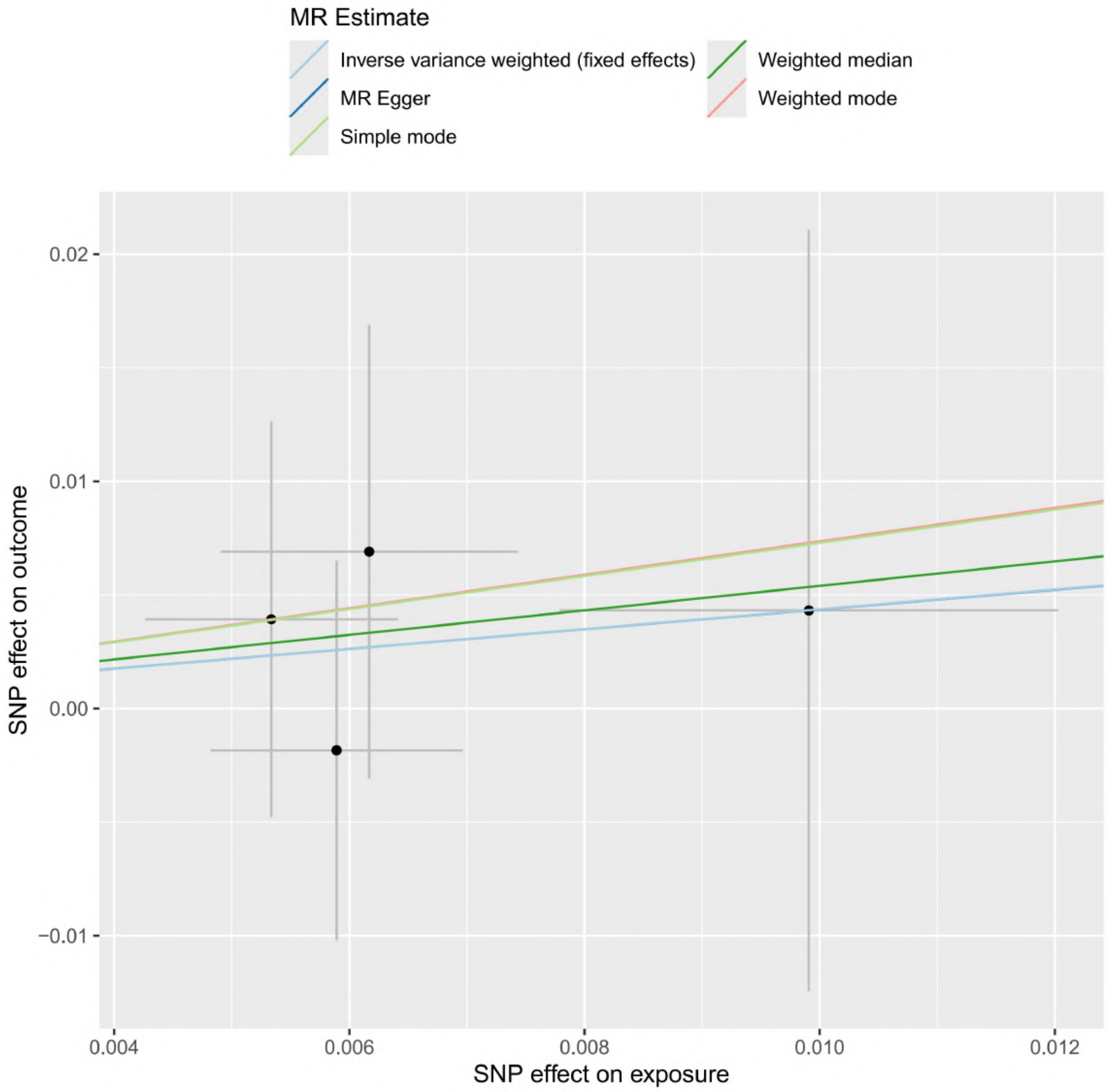

Supplementary Fig. 13 Scatter plot of the causal effect of Lobster/crab intake on GERD

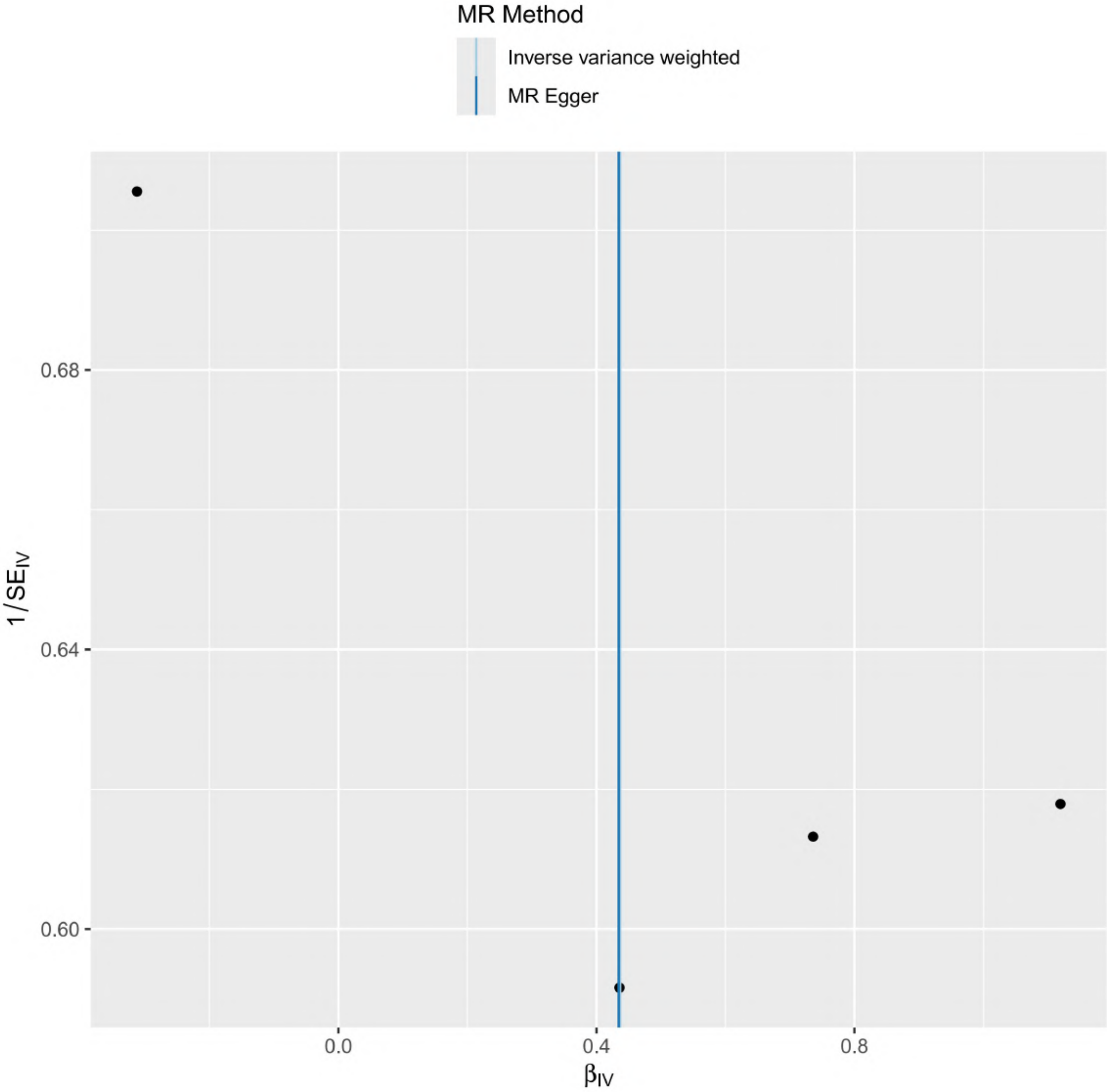

Supplementary Fig. 14 Funnel plot of the causal effect of Lobster/crab intake on GERD

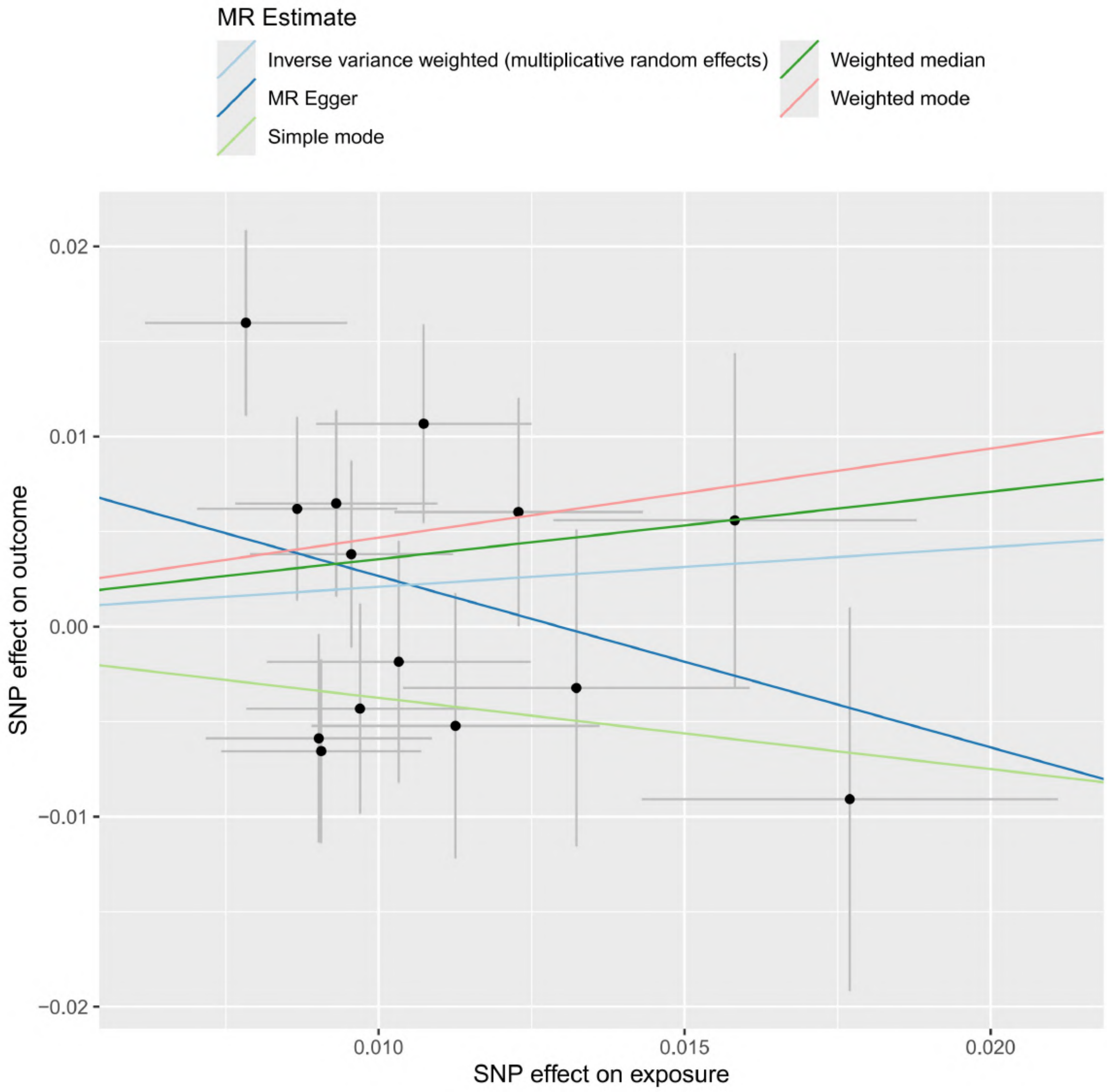

Supplementary Fig. 15 Scatter plot of the causal effect of Non-oily fish intake on GERD

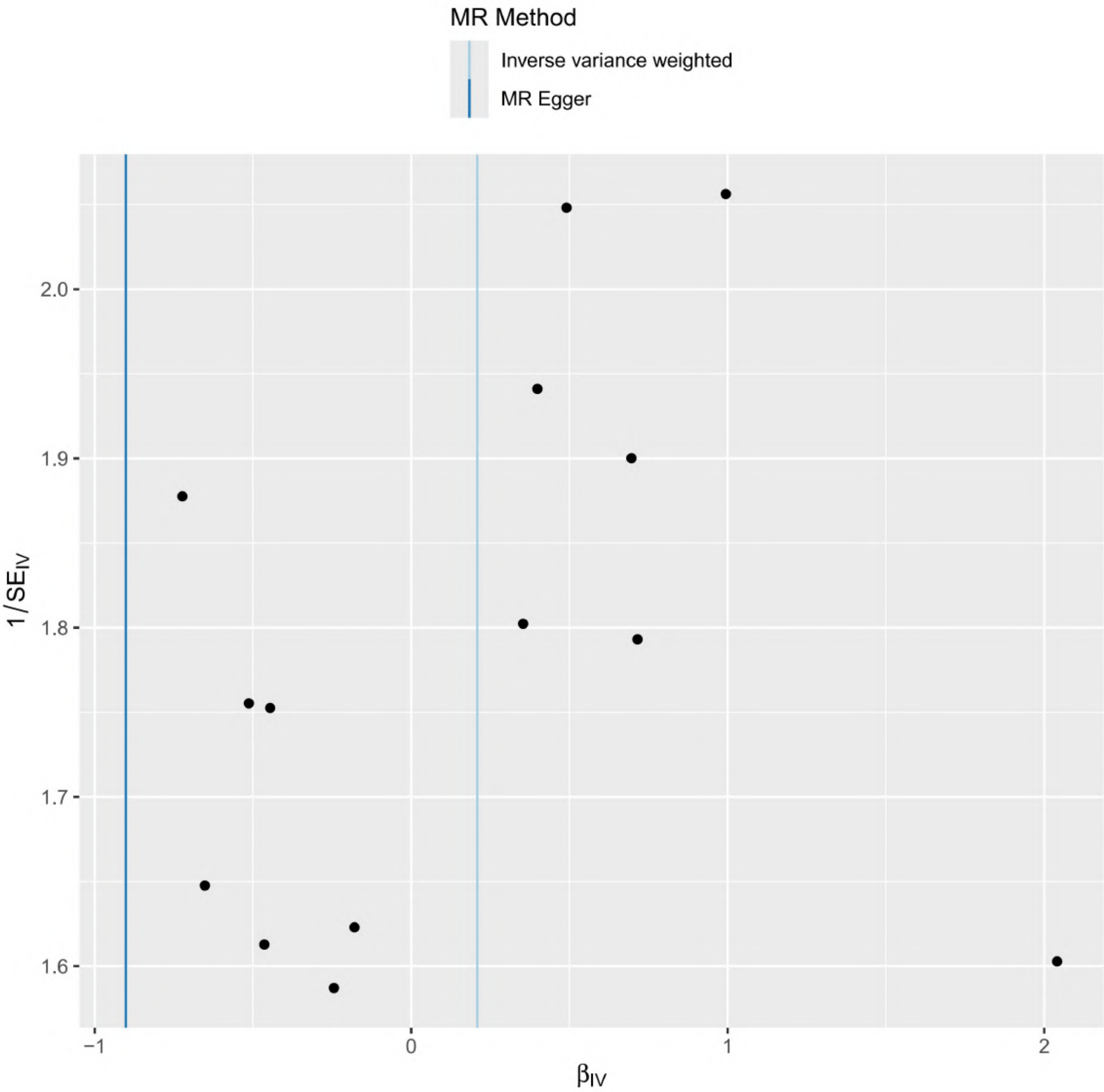

Supplementary Fig. 16 Funnel plot of the causal effect of Non-oily fish intake on GERD

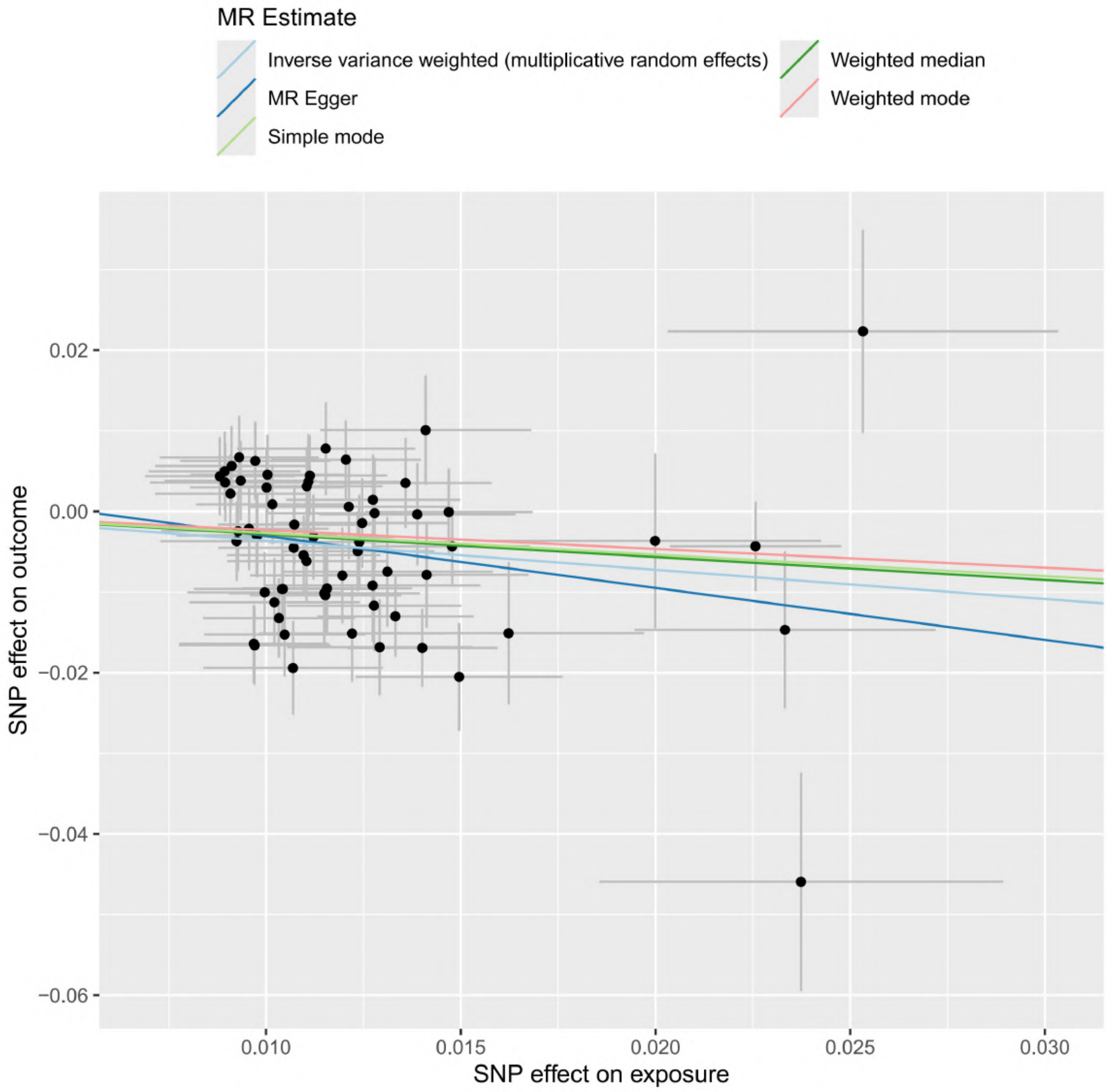

Supplementary Fig. 17 Scatter plot of the causal effect of Oily fish intake on GERD

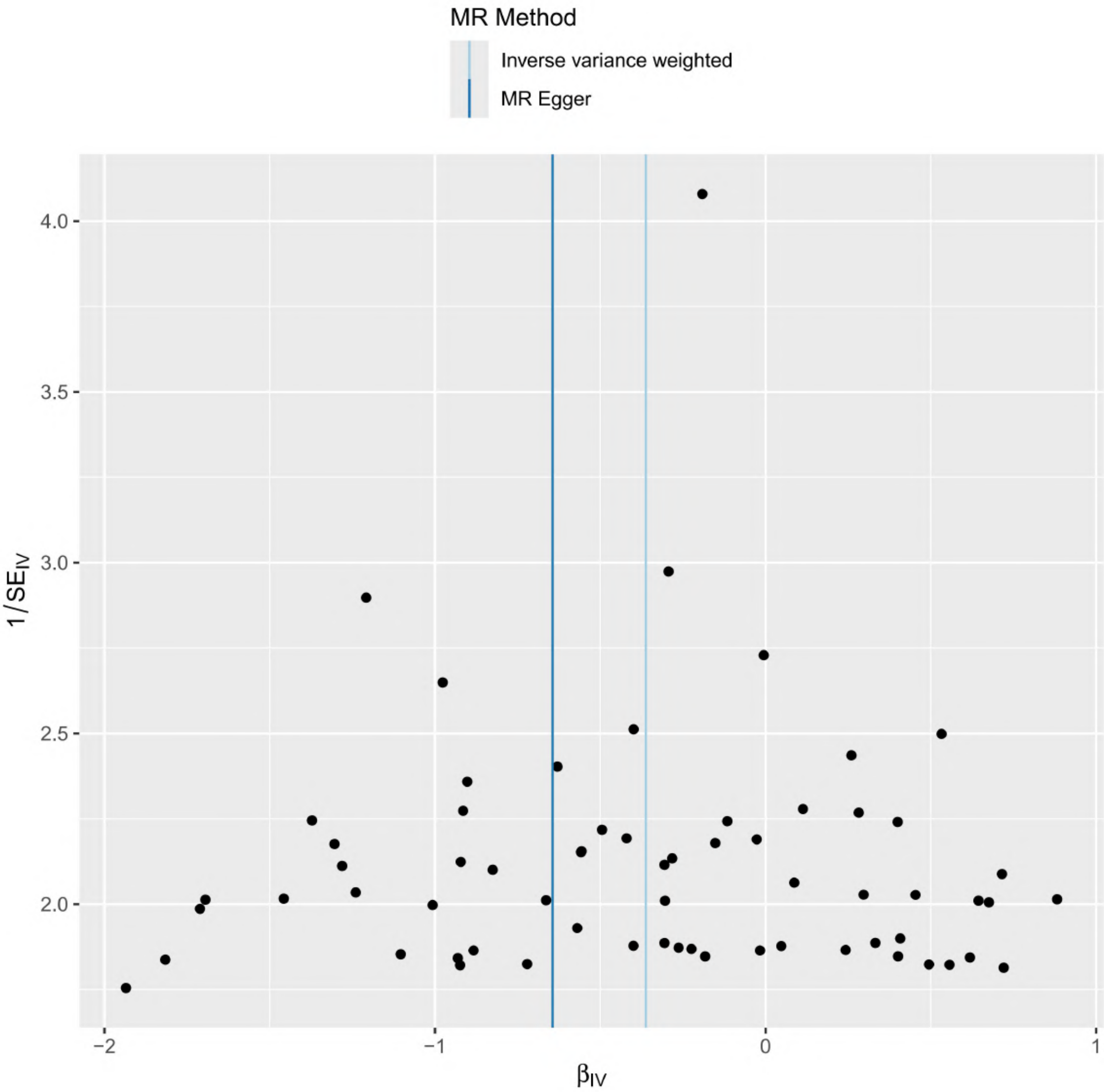

Supplementary Fig. 18 Funnel plot of the causal effect of Oily fish intake on GERD

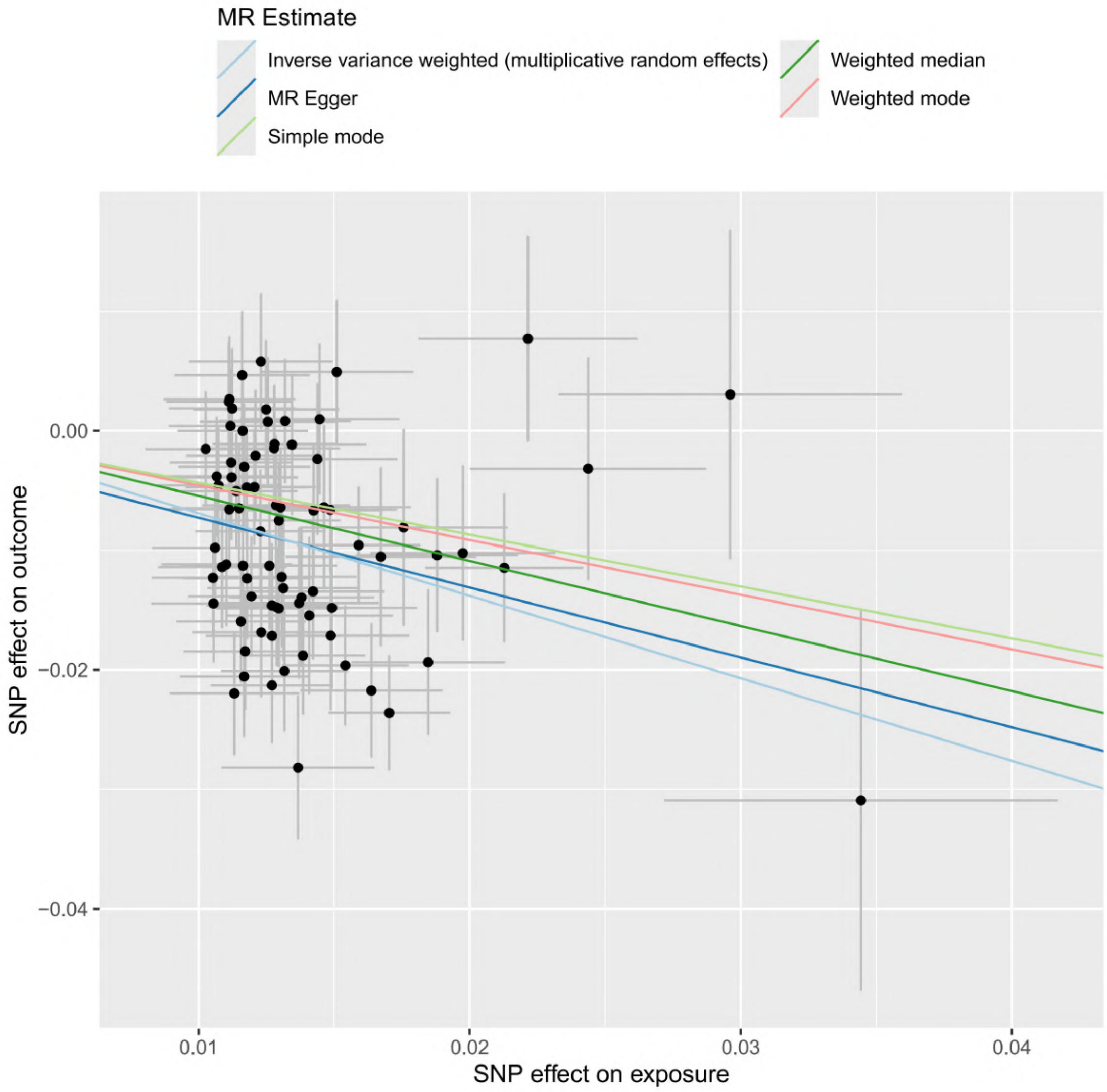

Supplementary Fig. 19 Scatter plot of the causal effect of cheese intake on GERD

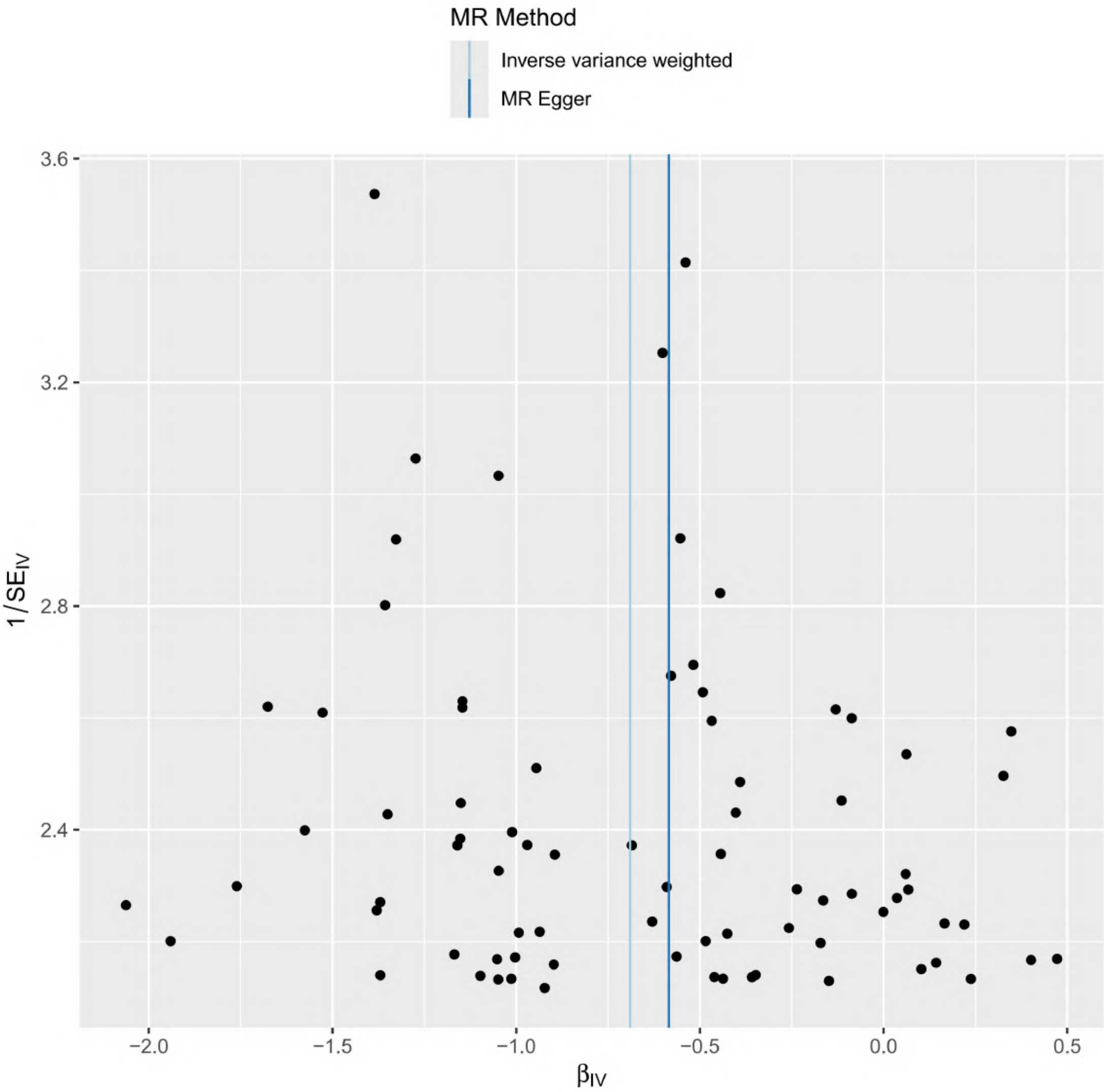

Supplementary Fig. 20 Funnel plot of the causal effect of cheese intake on GERD

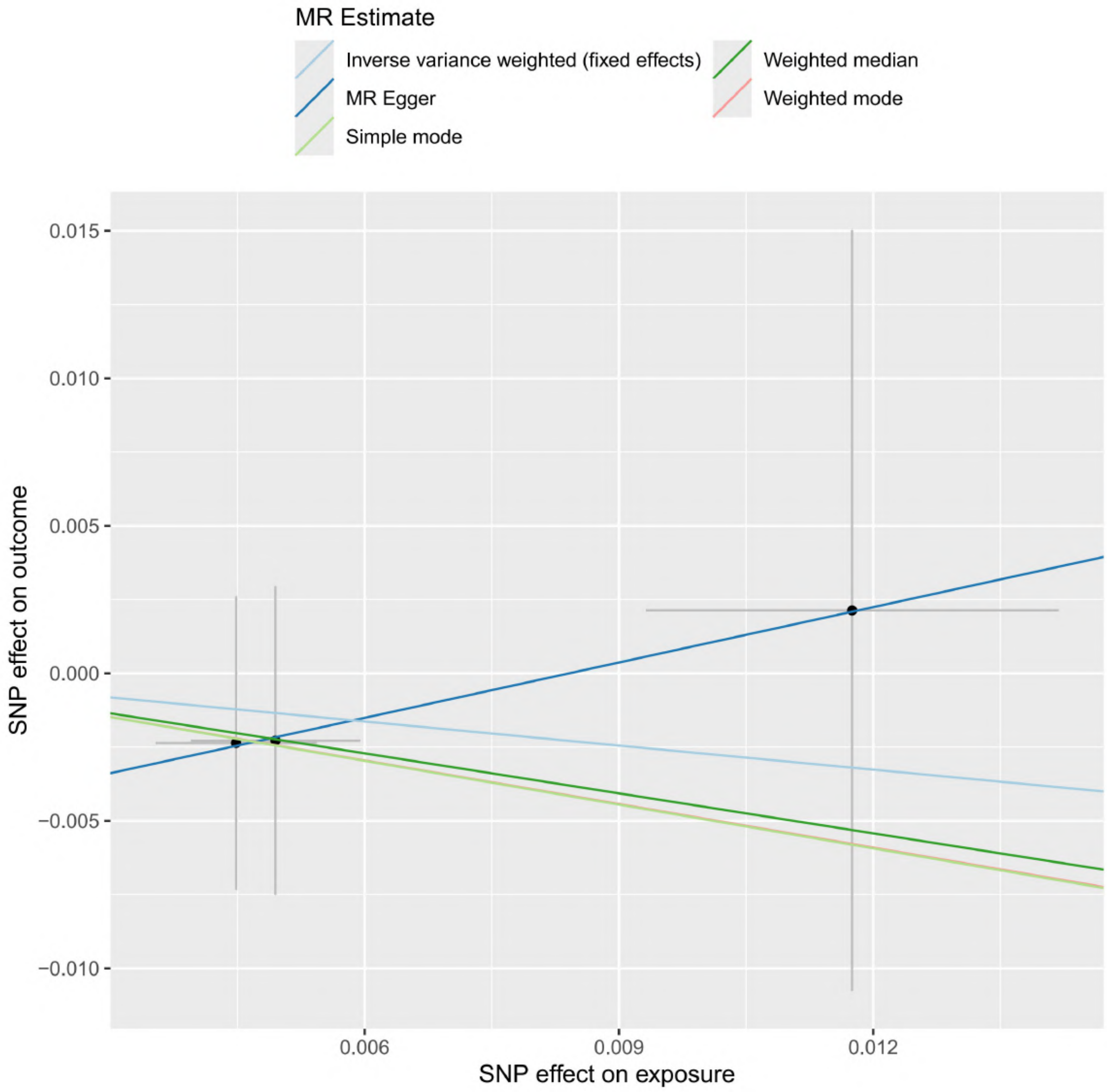

Supplementary Fig. 21 Scatter plot of the causal effect of feta intake on GERD

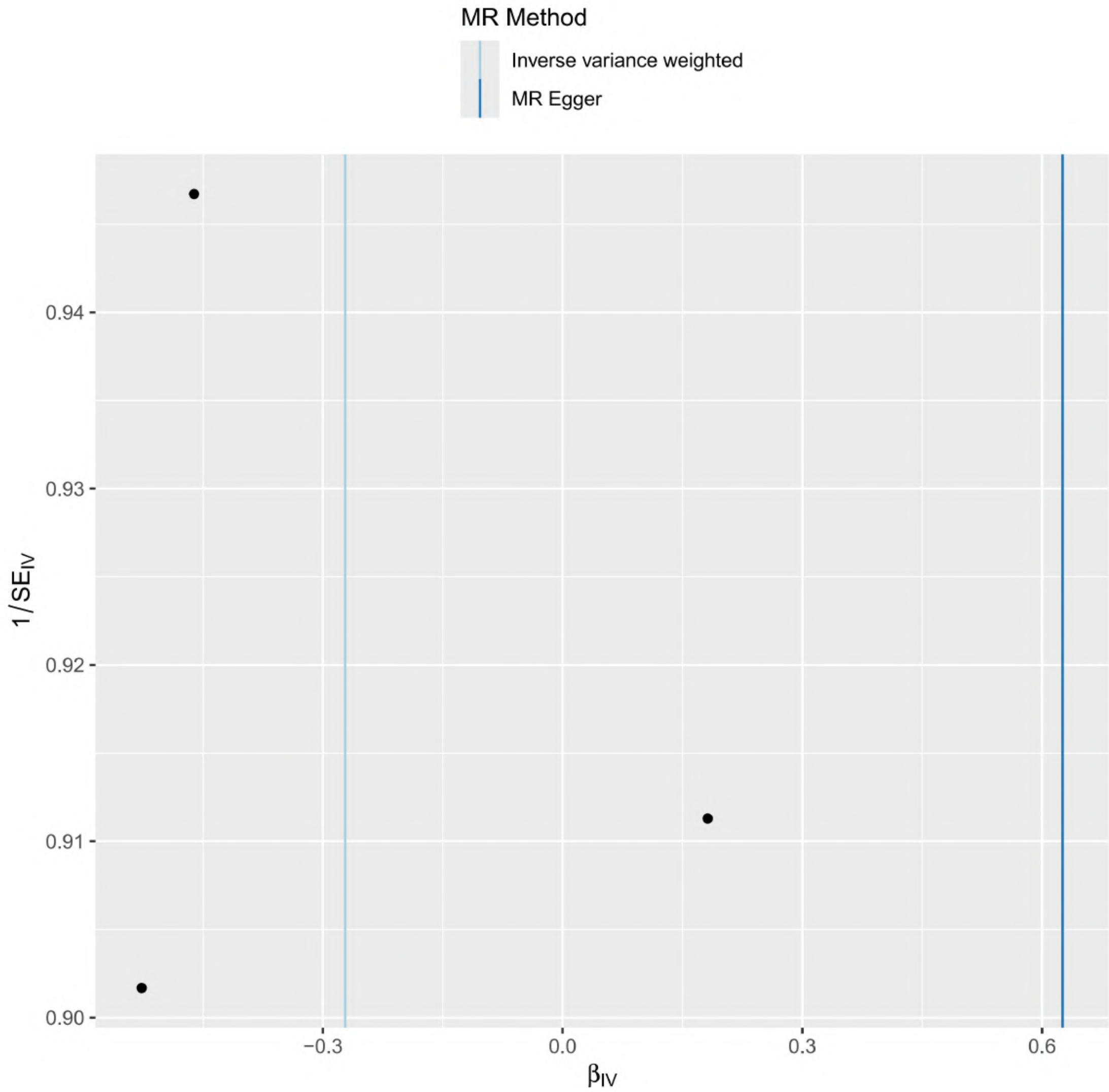

Supplementary Fig. 22 Funnel plot of the causal effect of feta intake on GERD

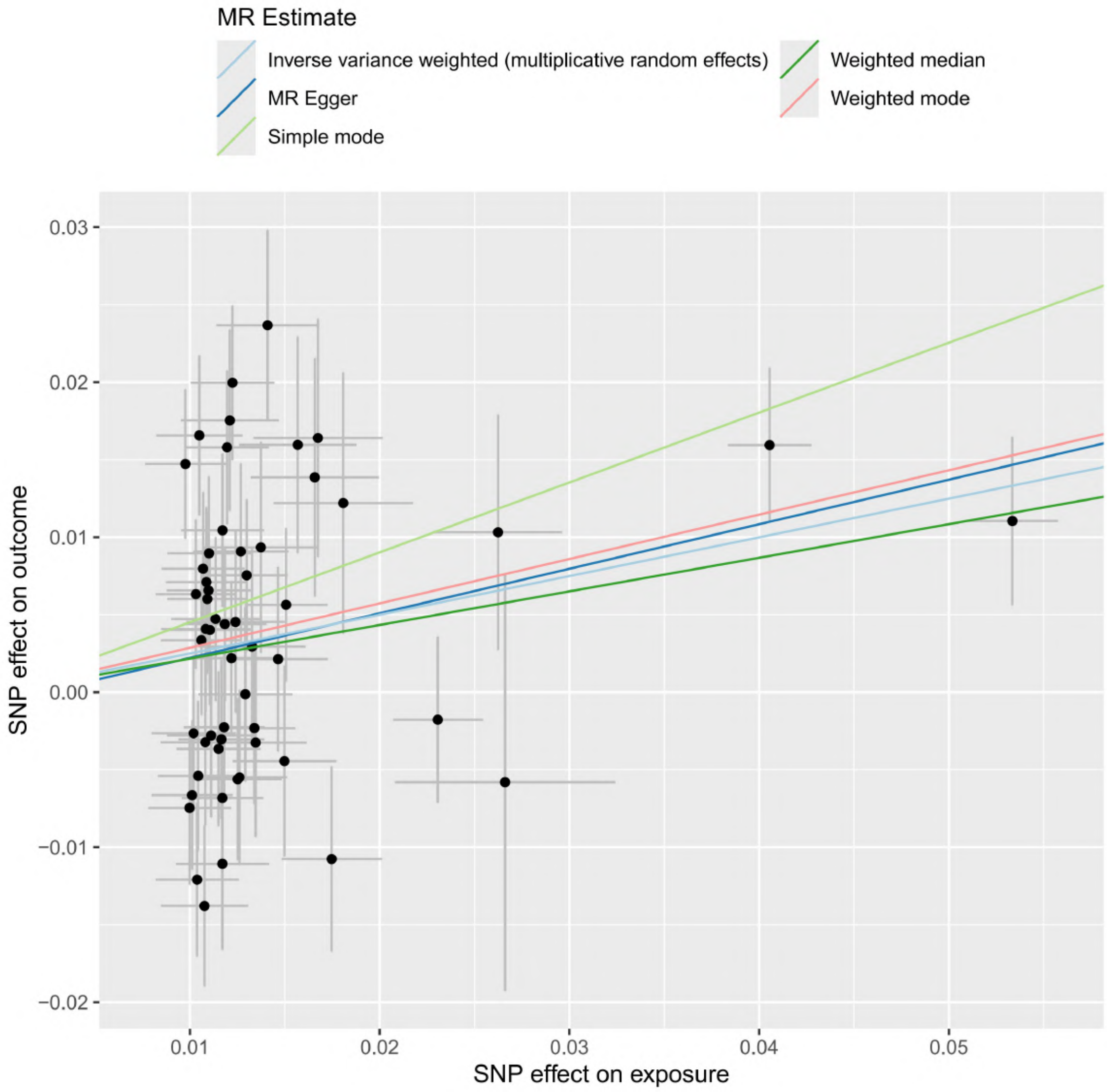

Supplementary Fig. 23 Scatter plot of the causal effect of tea intake on GERD

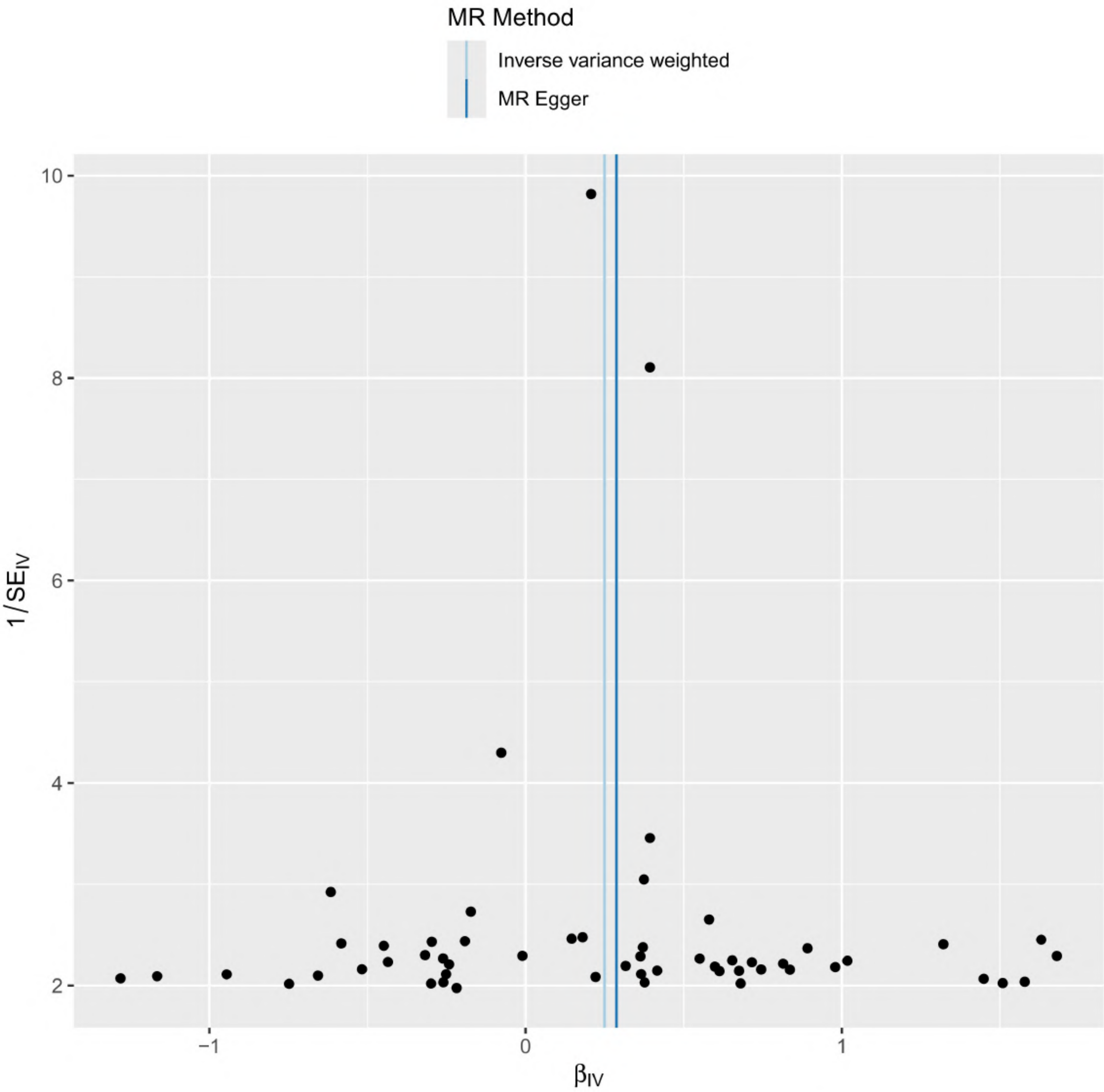

Supplementary Fig. 24 Funnel plot of the causal effect of tea intake on GERD

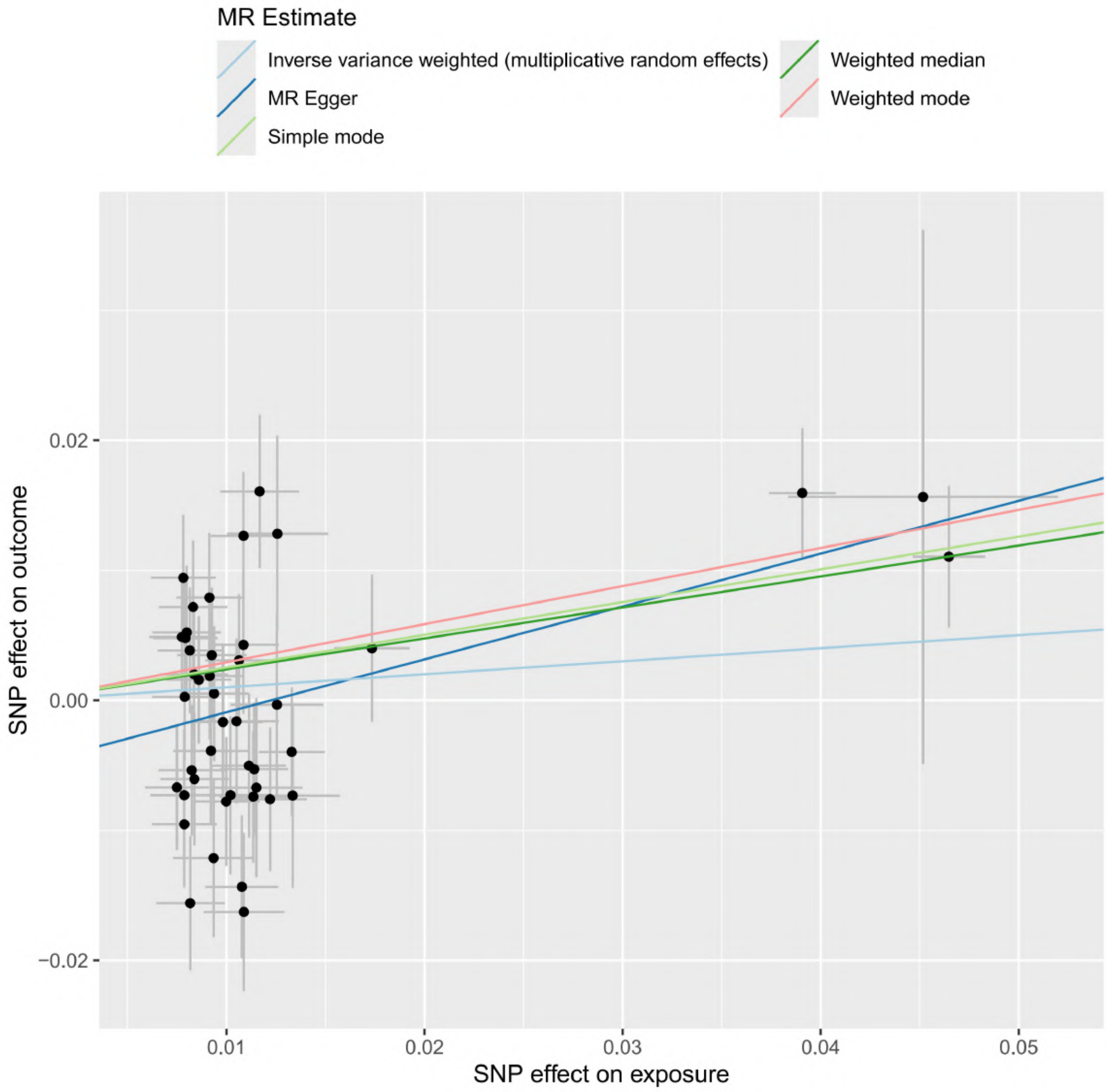

Supplementary Fig. 25 Scatter plot of the causal effect of coffee intake on GERD

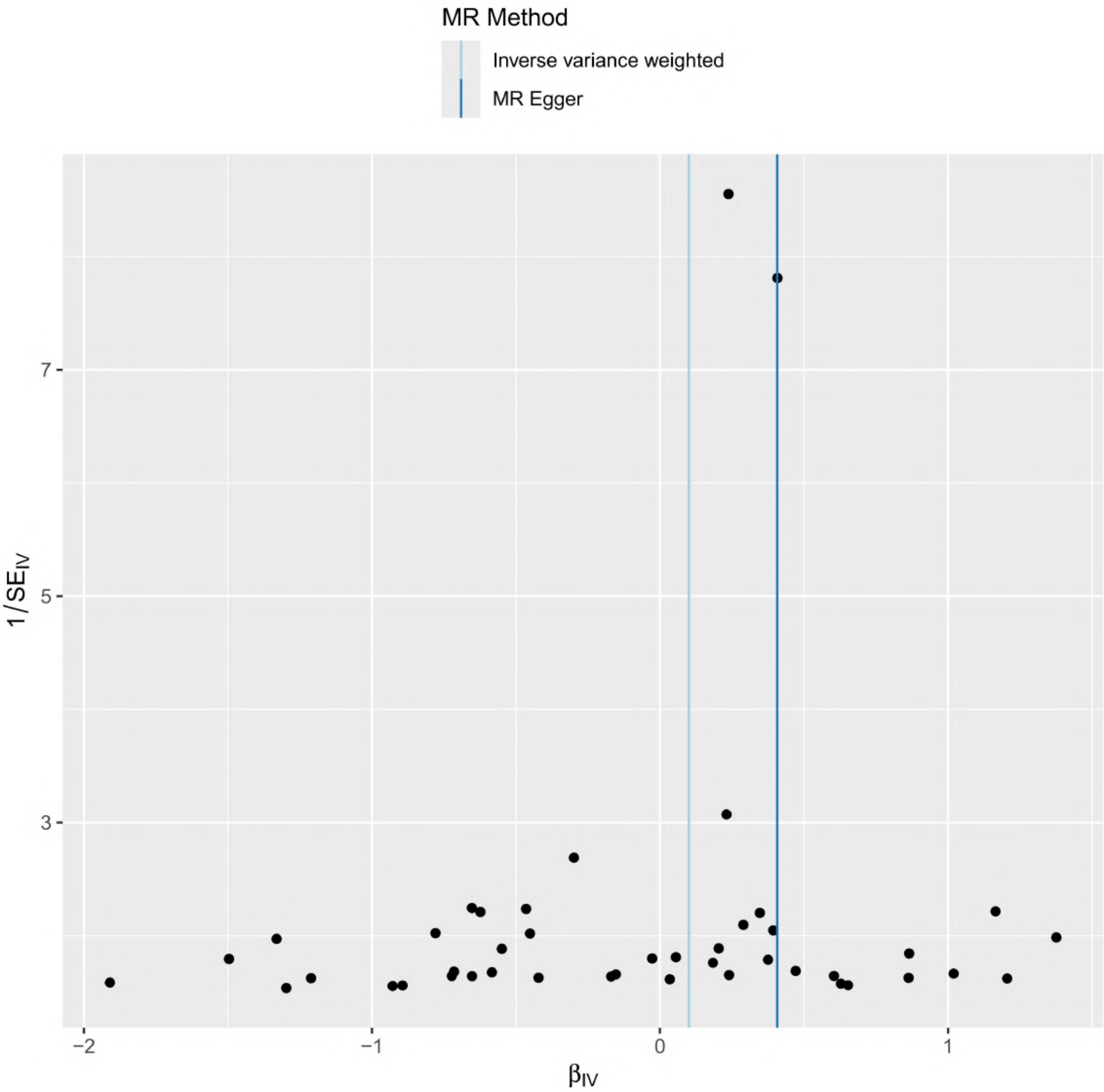

Supplementary Fig. 26 Funnel plot of the causal effect of coffee intake on GERD

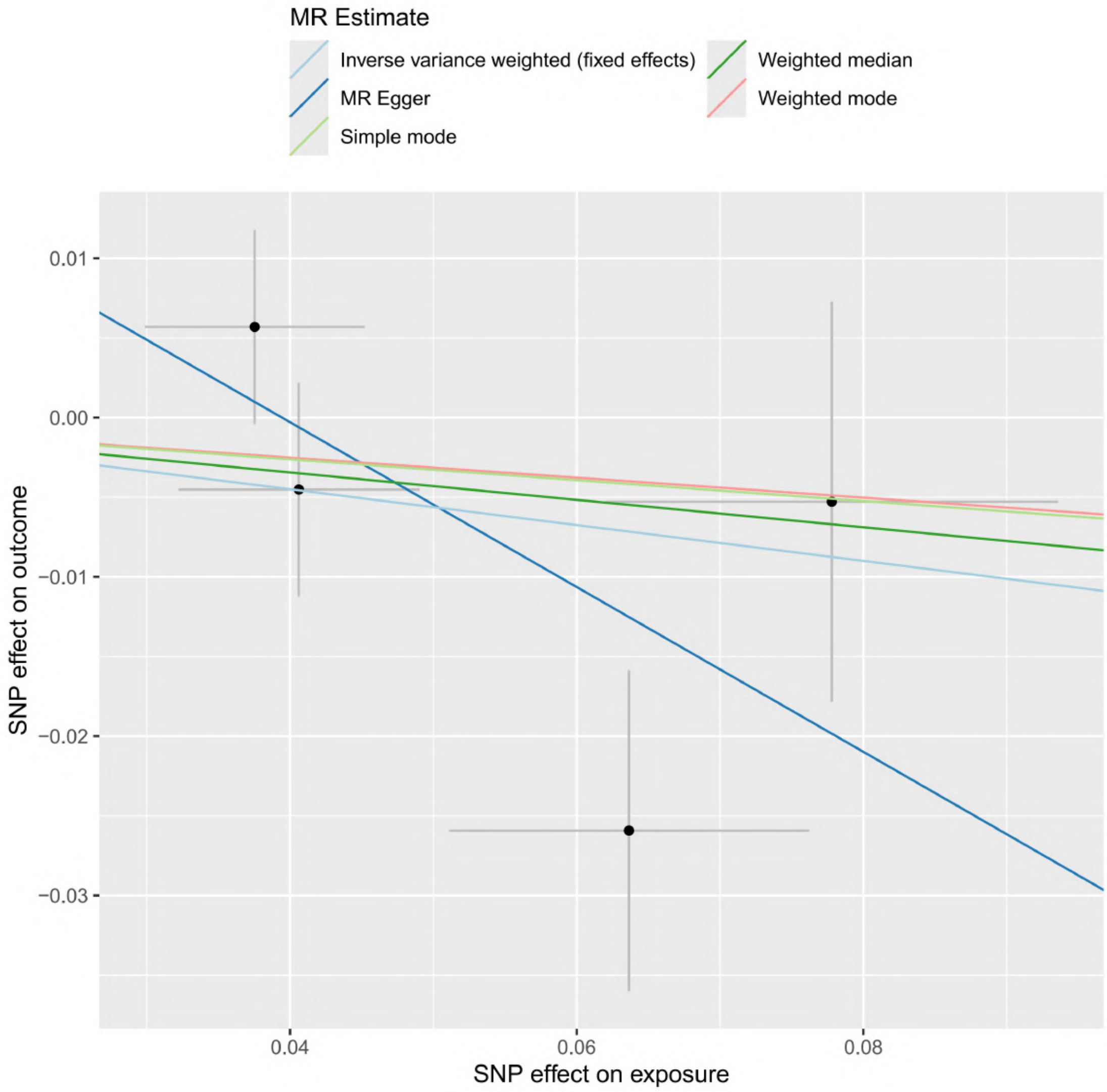

Supplementary Fig. 27 Scatter plot of the causal effect of beer/cider intake on GERD

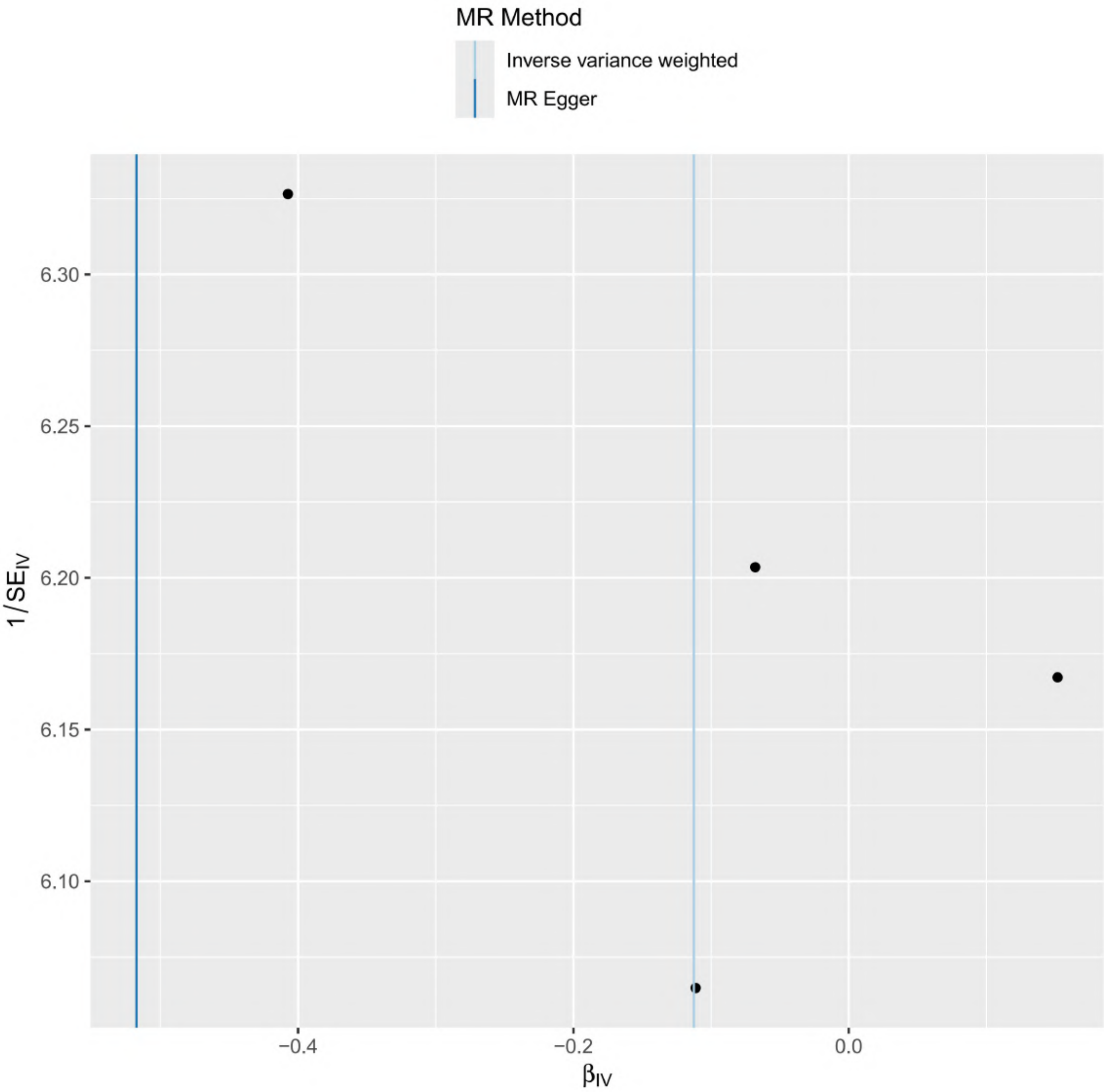

Supplementary Fig. 28 Funnel plot of the causal effect of beer/cider intake on GERD

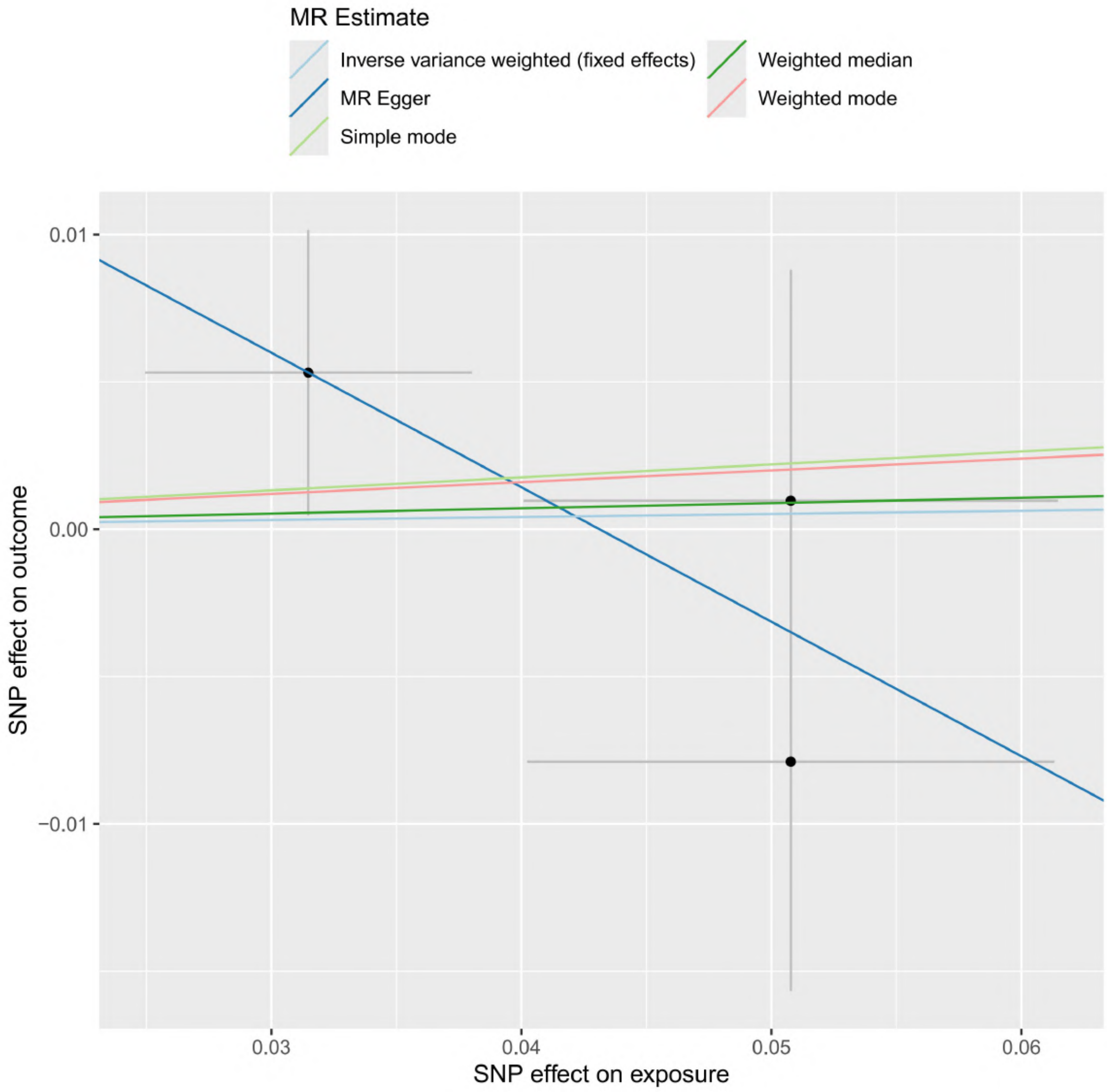

Supplementary Fig. 29 Scatter plot of the causal effect of red wine intake on GERD

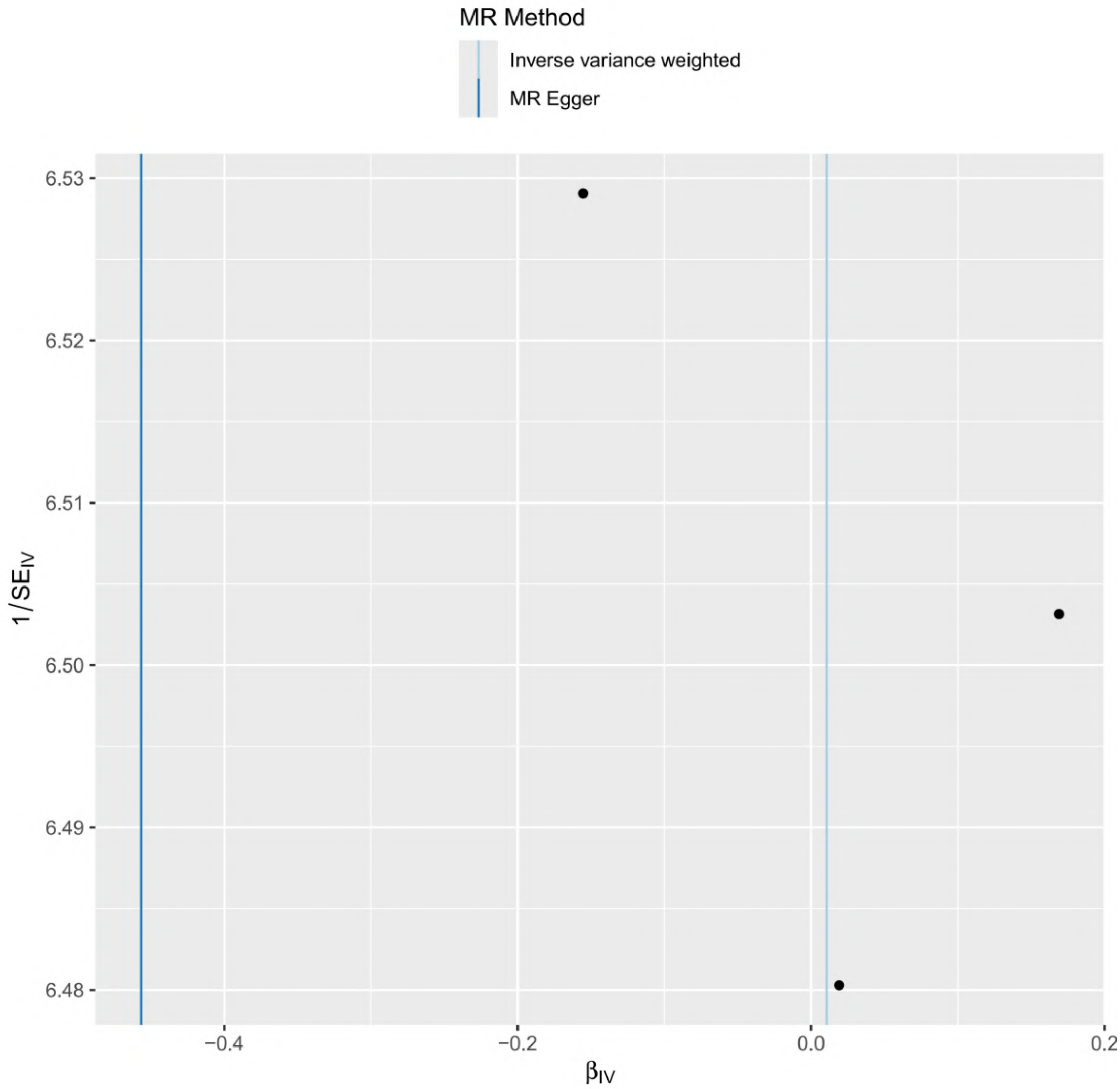

Supplementary Fig. 30 Funnel plot of the causal effect of red wine intake on GERD

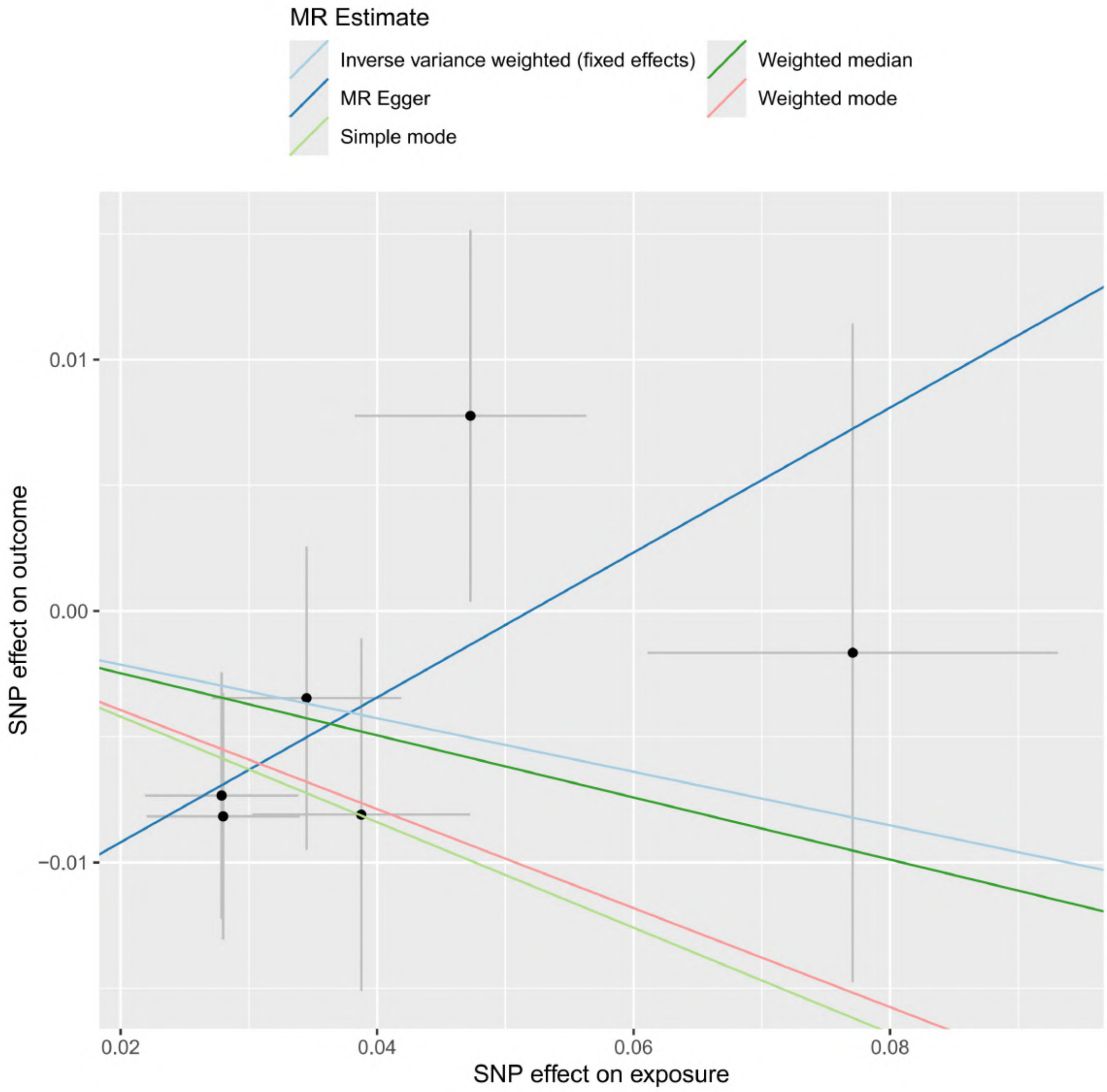

Supplementary Fig. 31 Scatter plot of the causal effect of apple intake on GERD

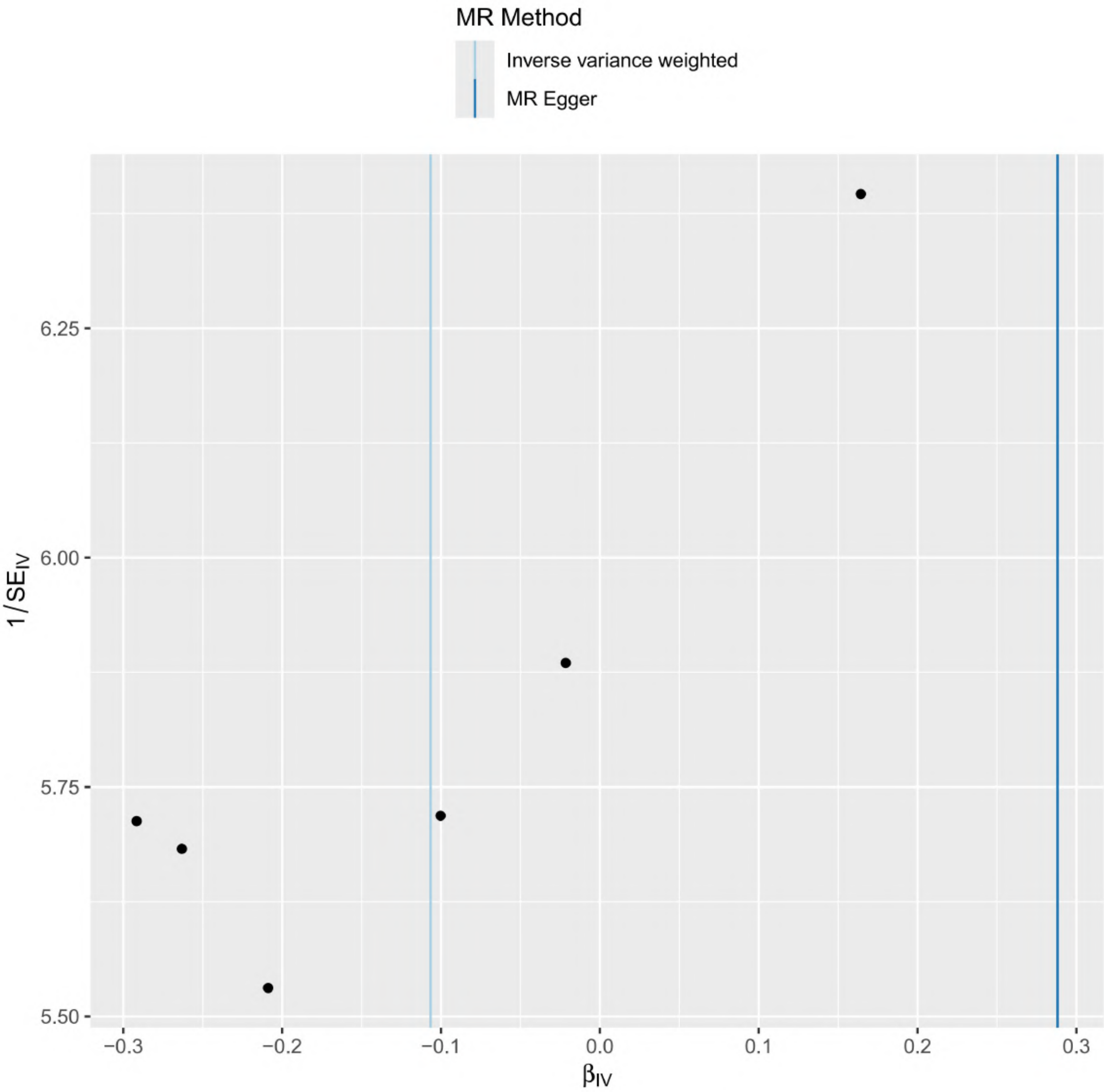

Supplementary Fig. 32 Funnel plot of the causal effect of apple intake on GERD

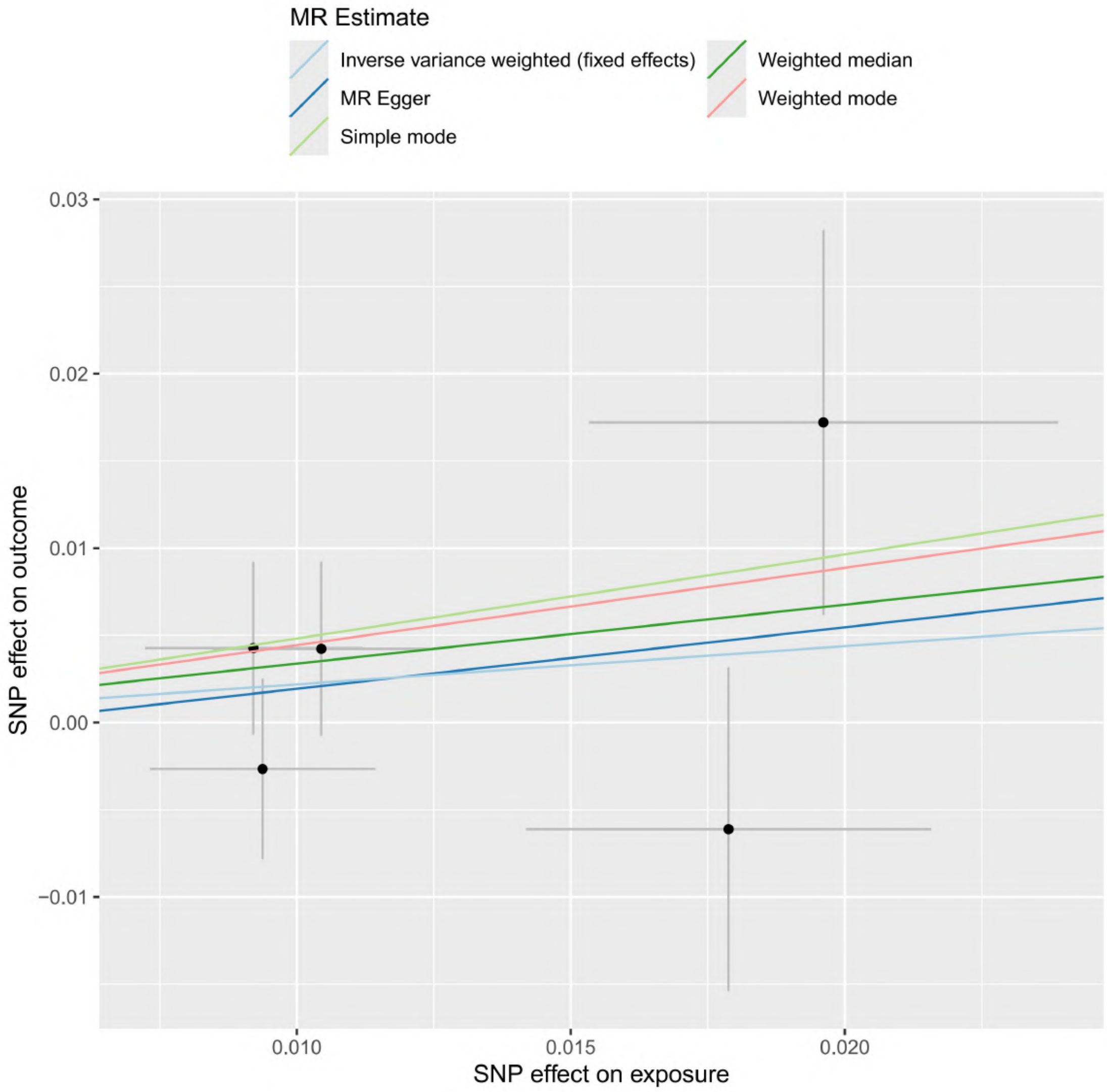

Supplementary Fig. 33 Scatter plot of the causal effect of cherry intake on GERD

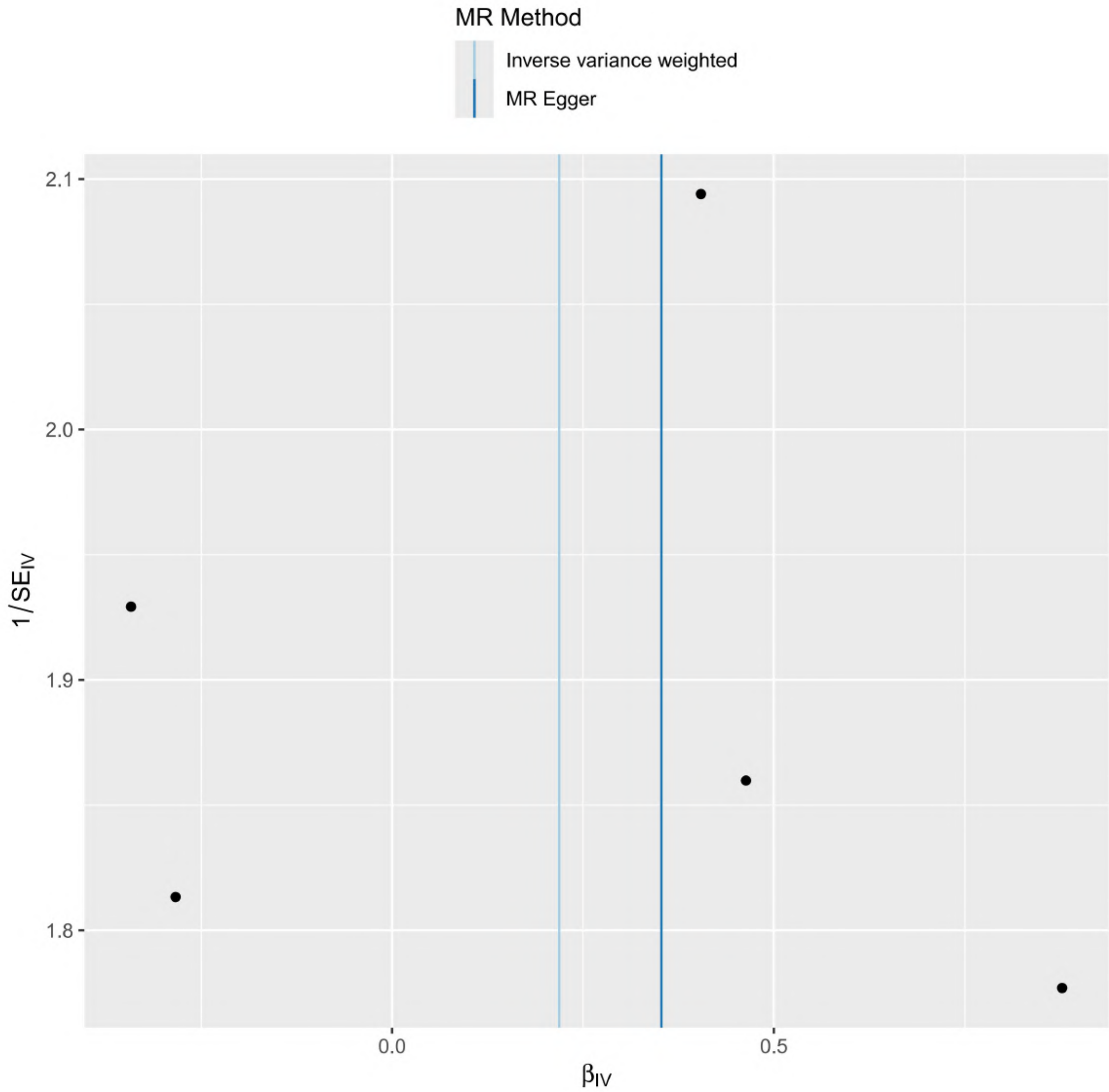

Supplementary Fig. 34 Funnel plot of the causal effect of cherry intake on GERD

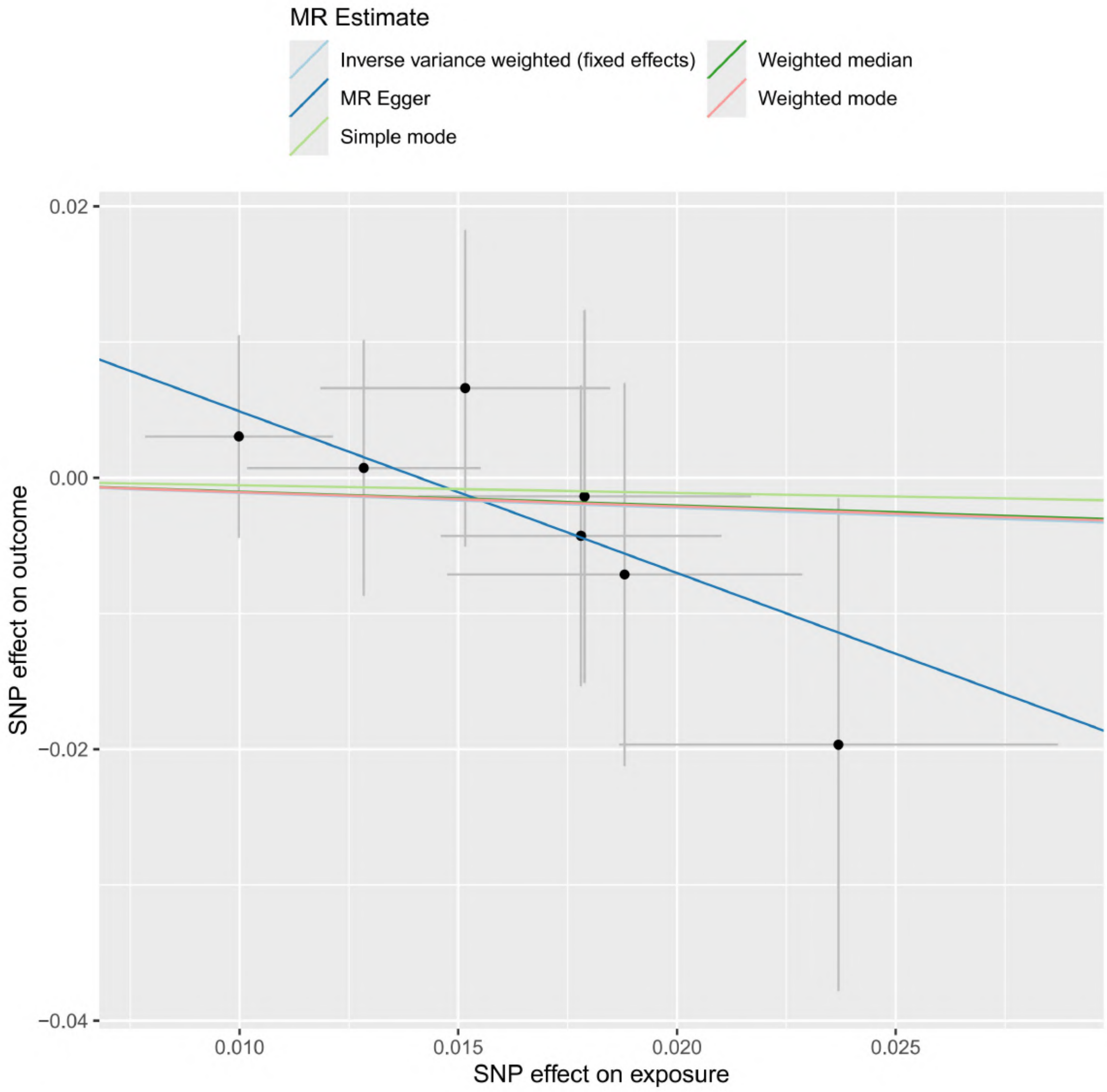

Supplementary Fig. 35 Scatter plot of the causal effect of mango intake on GERD

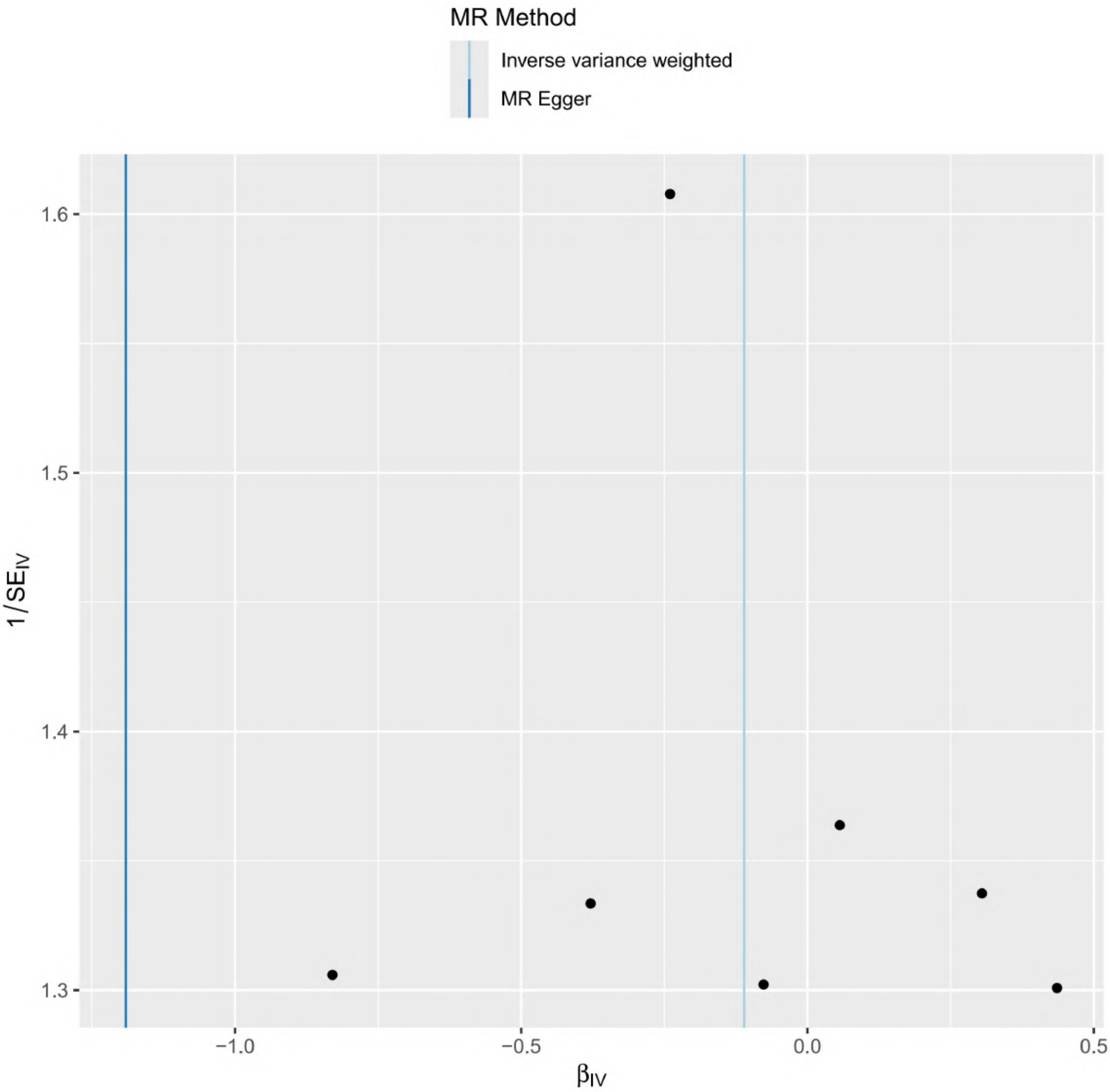

Supplementary Fig. 36 Funnel plot of the causal effect of mango intake on GERD

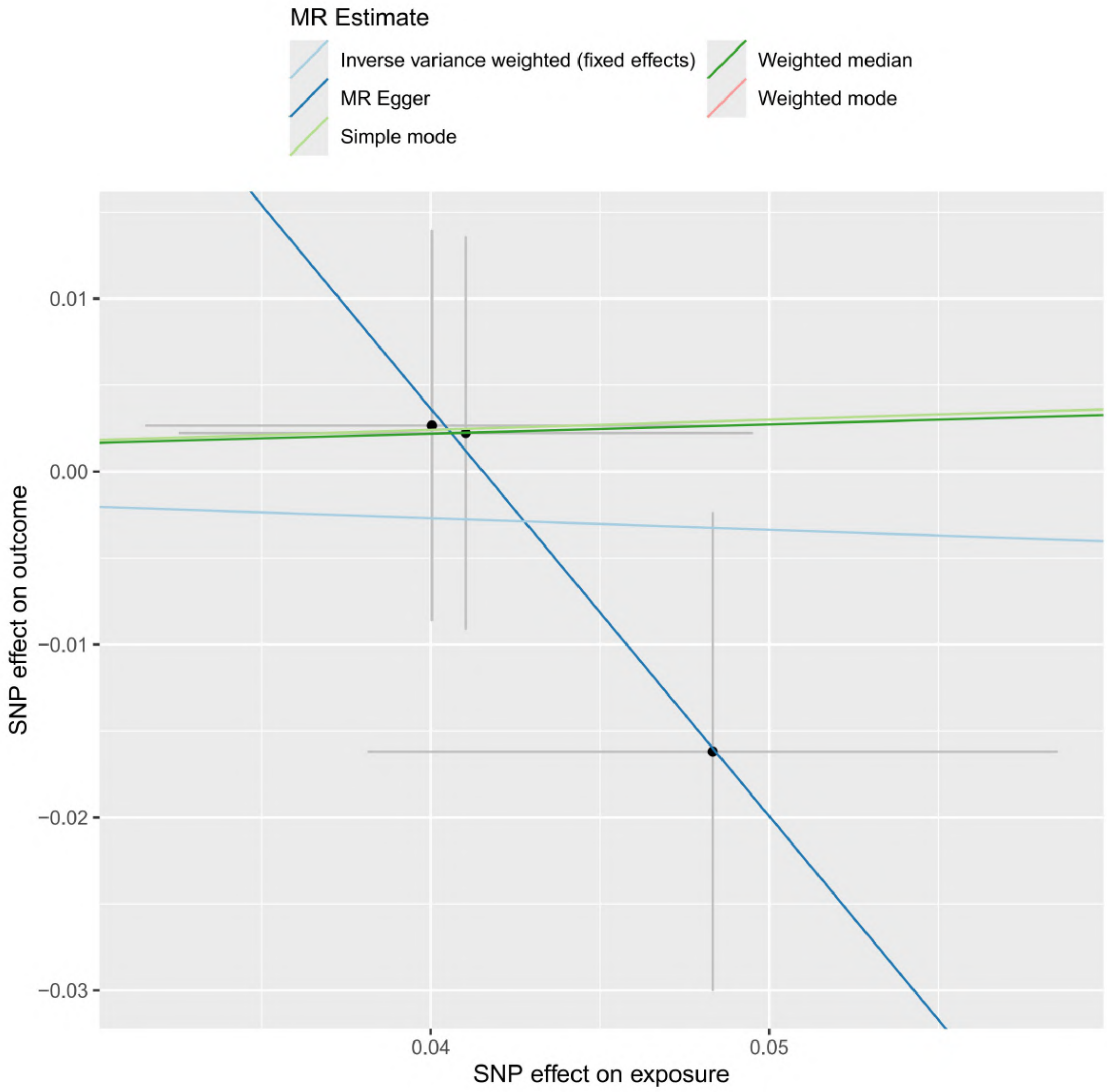

Supplementary Fig. 37 Scatter plot of the causal effect of garlic intake on GERD

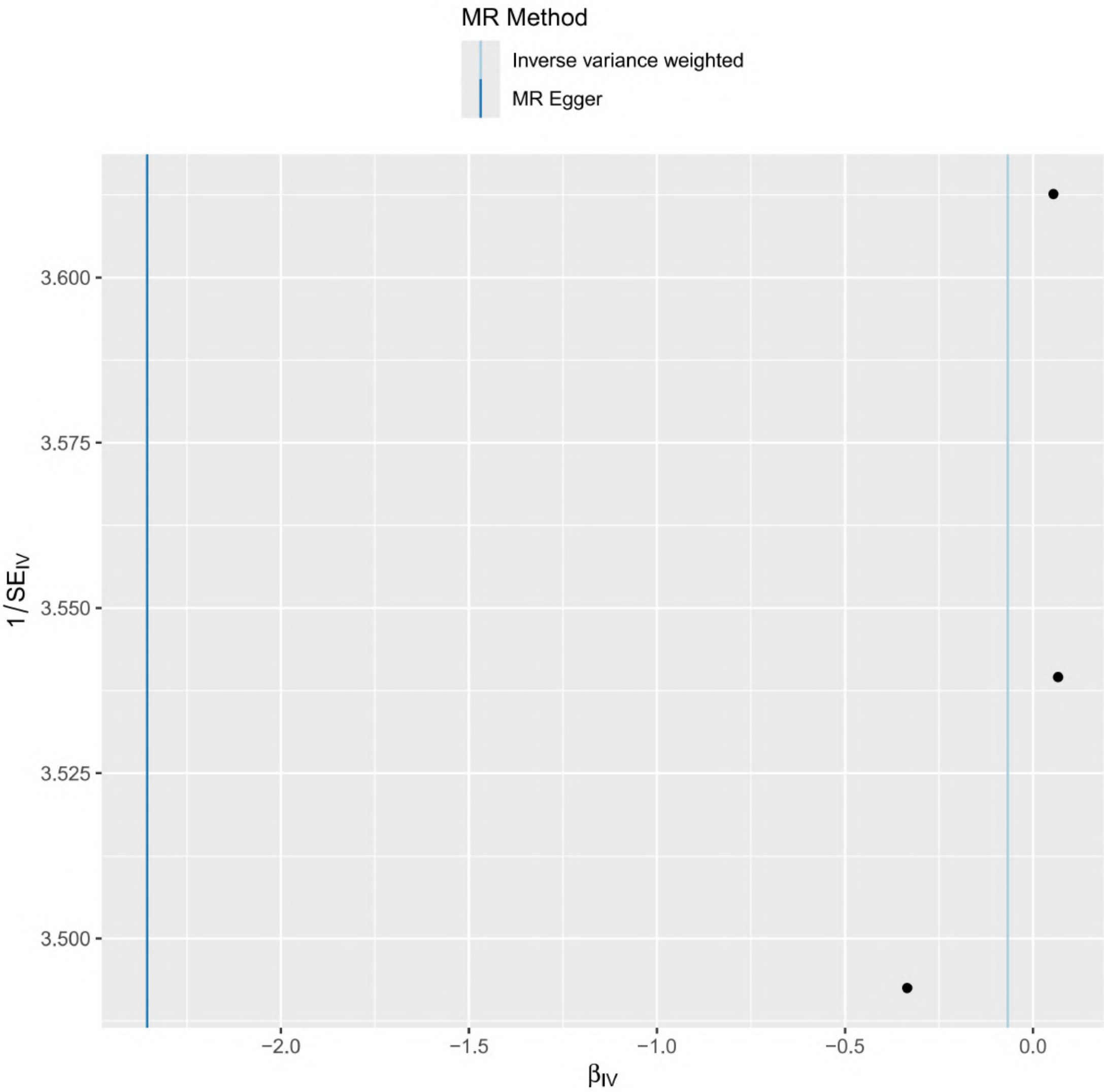

Supplementary Fig. 38 Funnel plot of the causal effect of garlic intake on GERD

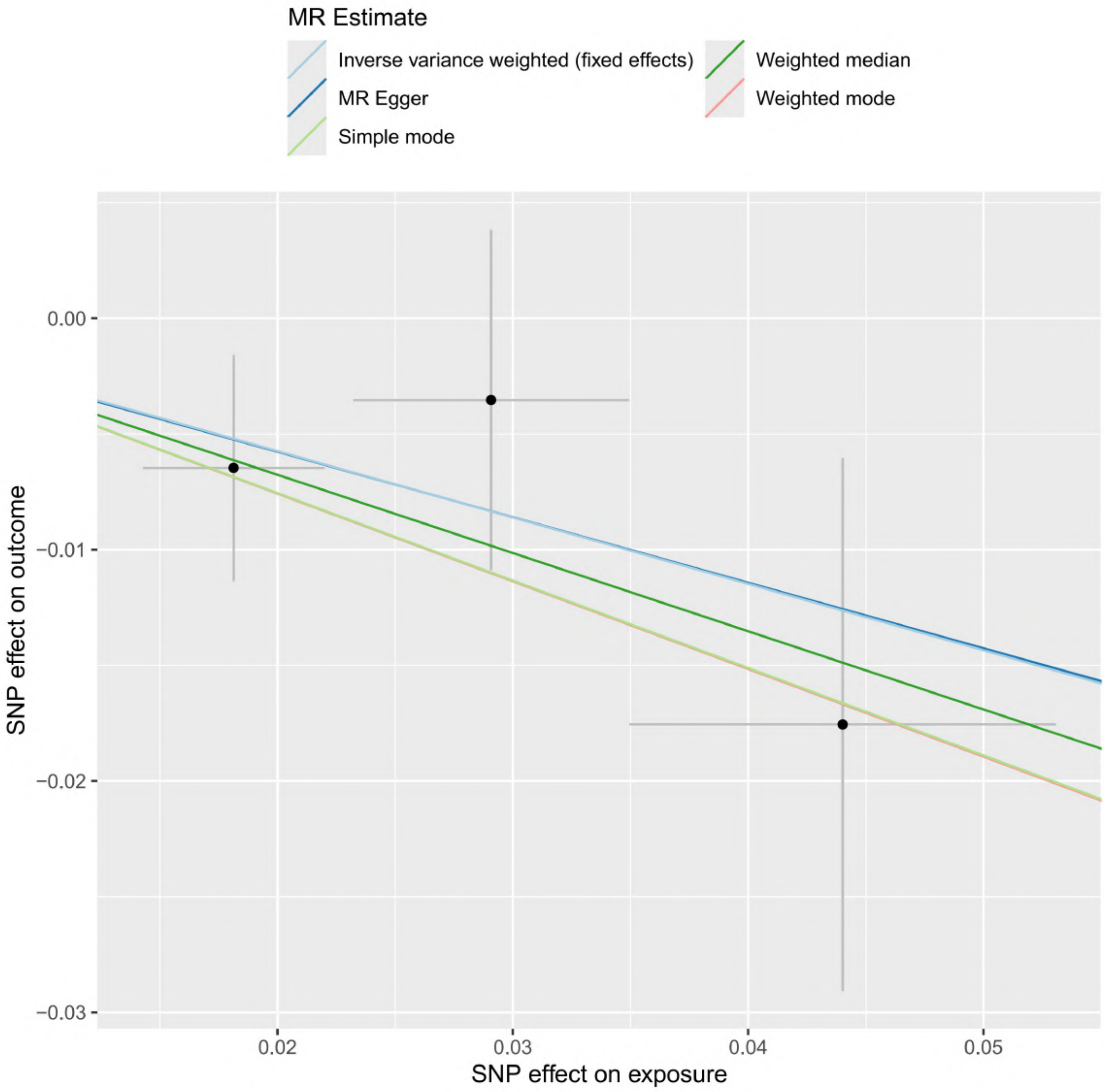

Supplementary Fig. 39 Scatter plot of the causal effect of celery intake on GERD

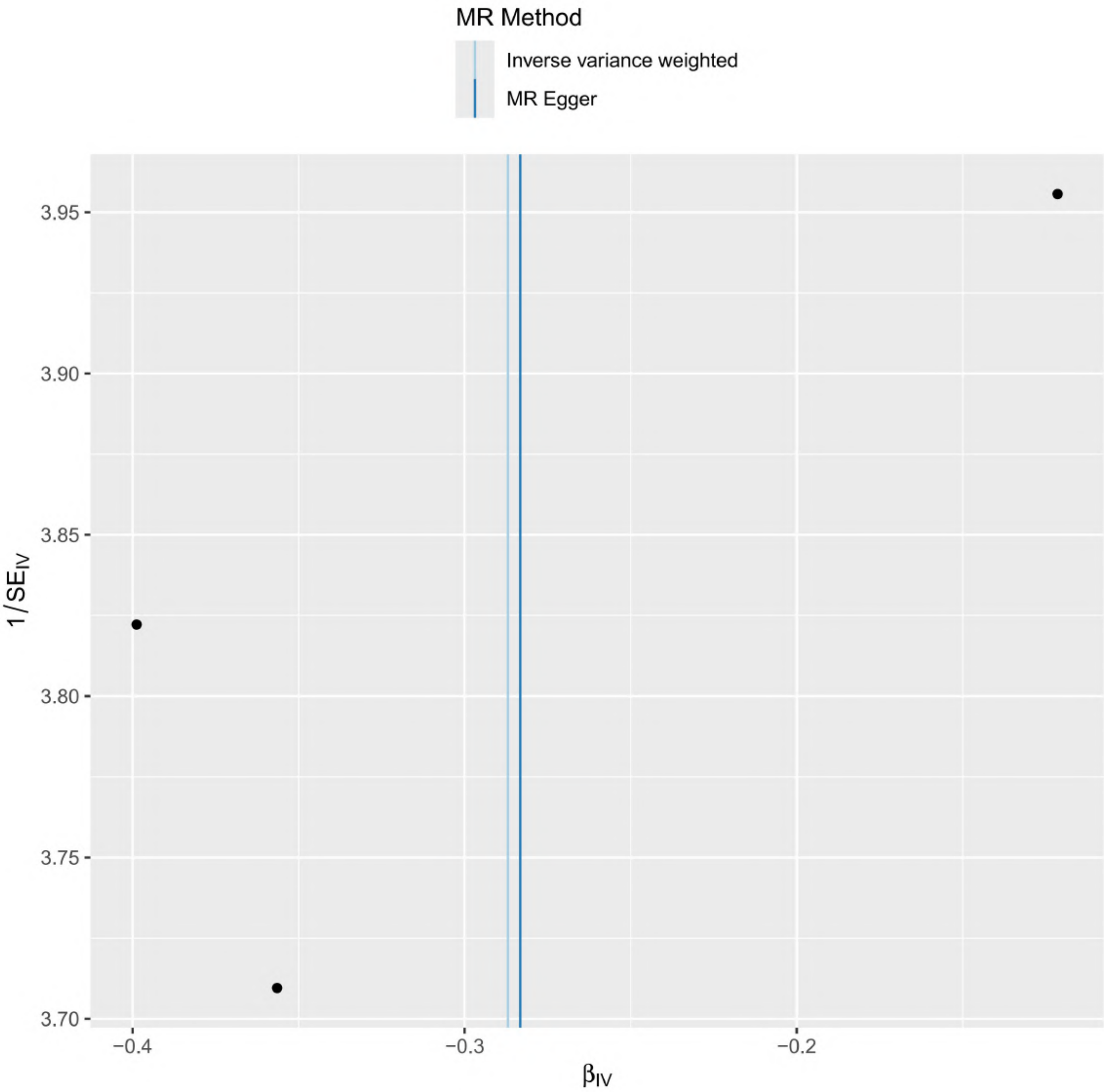

Supplementary Fig. 40 Funnel plot of the causal effect of celery intake on GERD

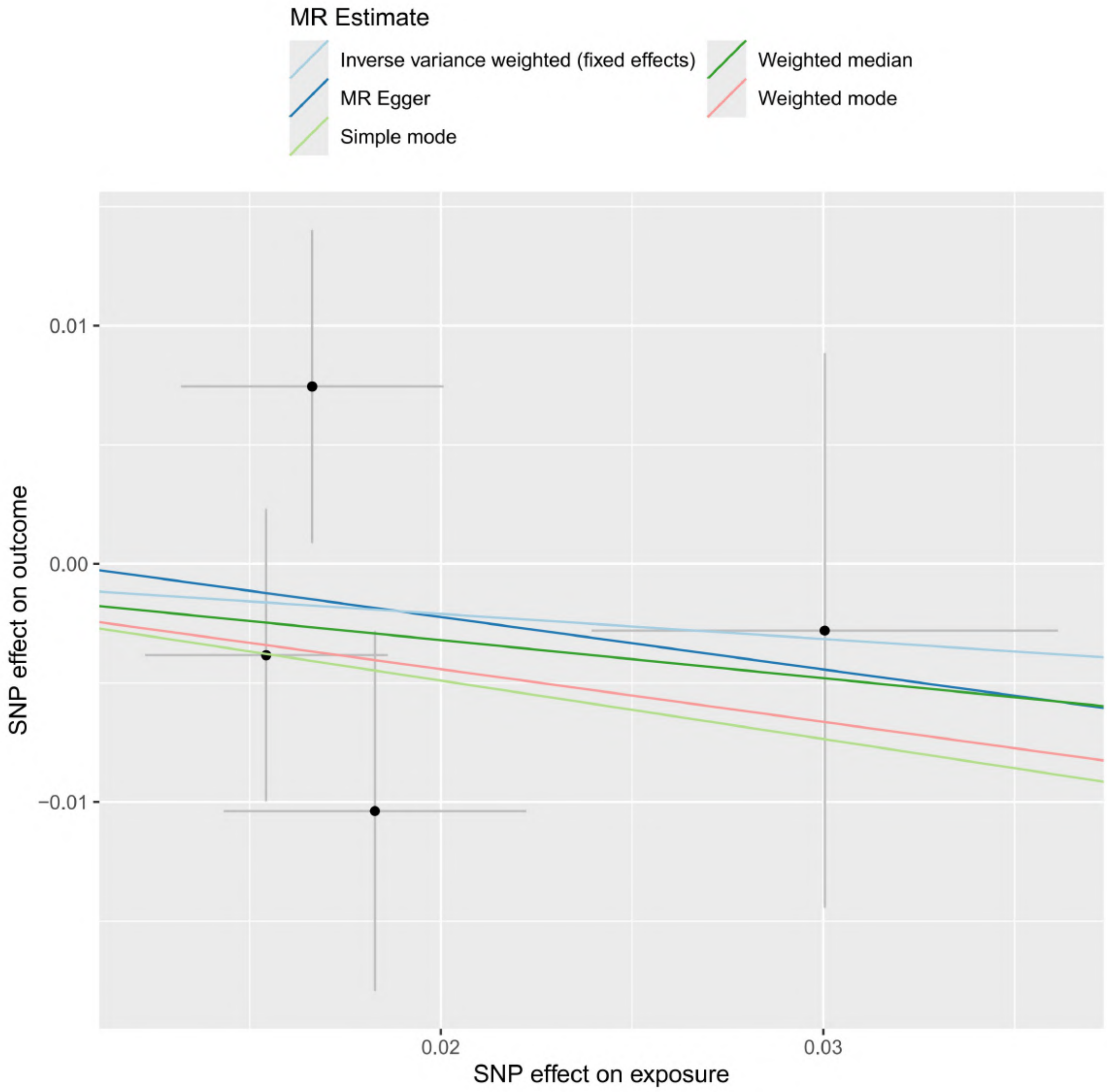

Supplementary Fig. 41 Scatter plot of the causal effect of milk intake on GERD

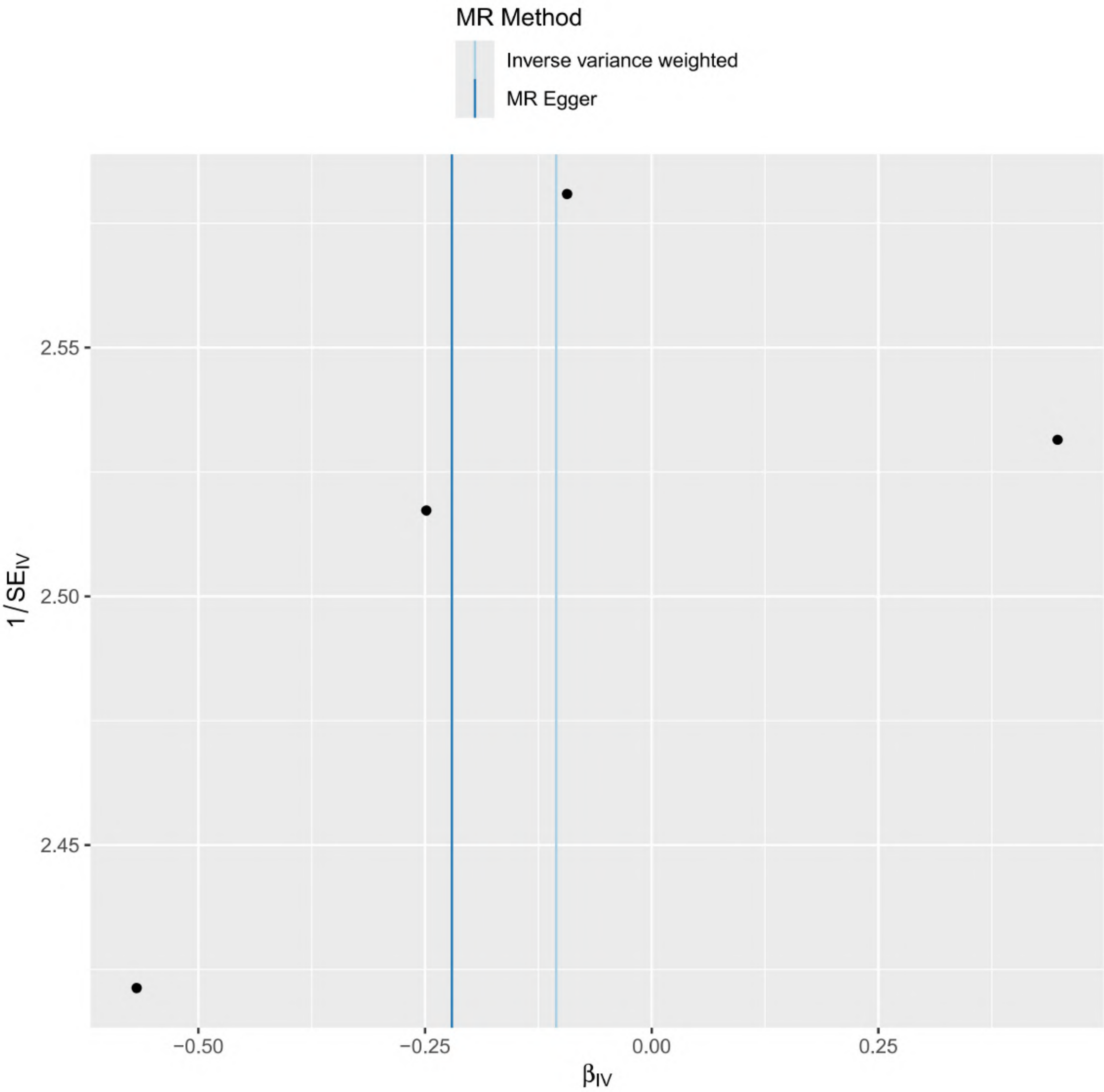

Supplementary Fig. 42 Funnel plot of the causal effect of milk intake on GERD

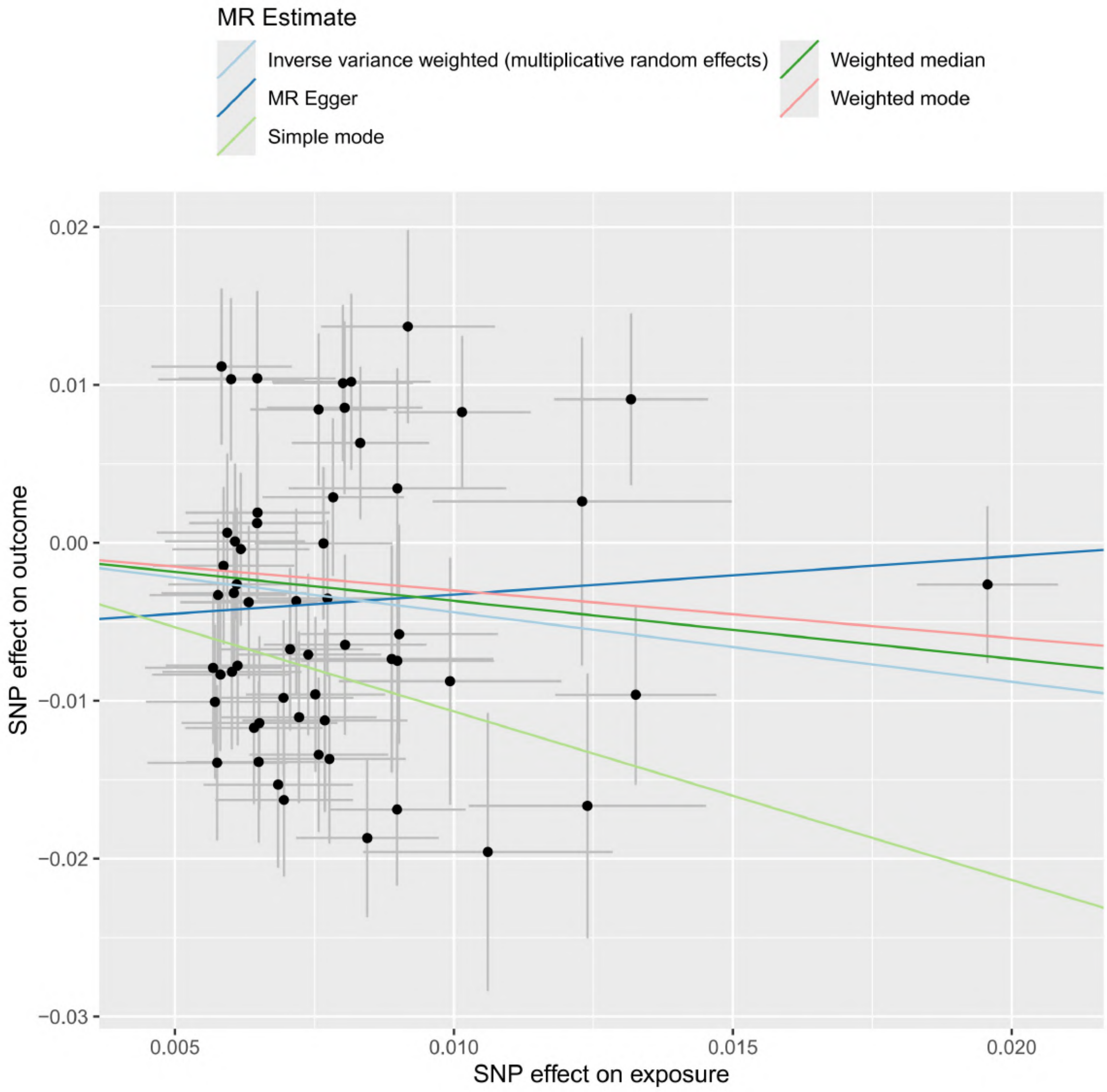

Supplementary Fig. 43 Scatter plot of the causal effect of fresh fruit intake on GERD

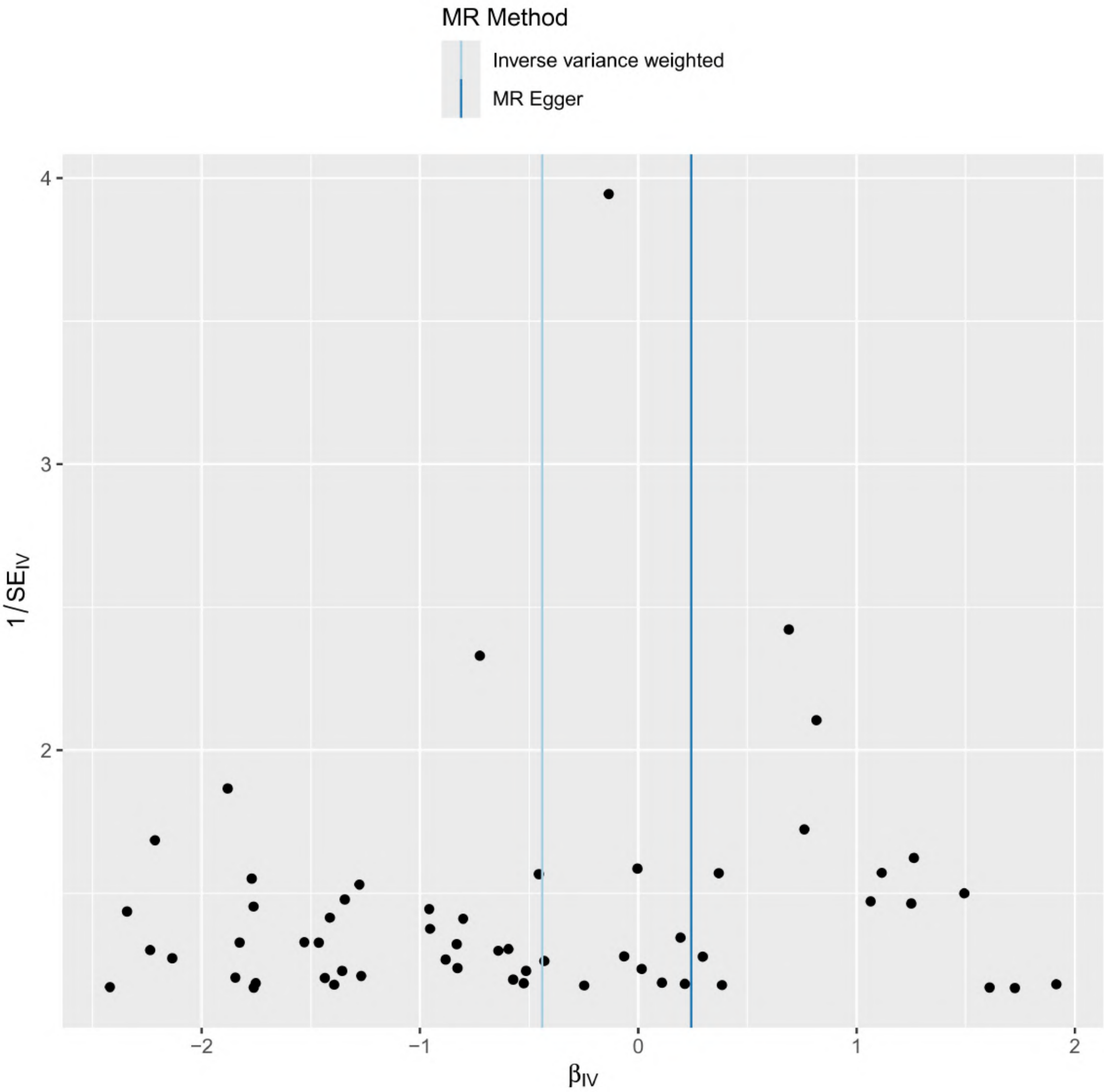

Supplementary Fig. 44 Funnel plot of the causal effect of fresh fruit intake on GERD

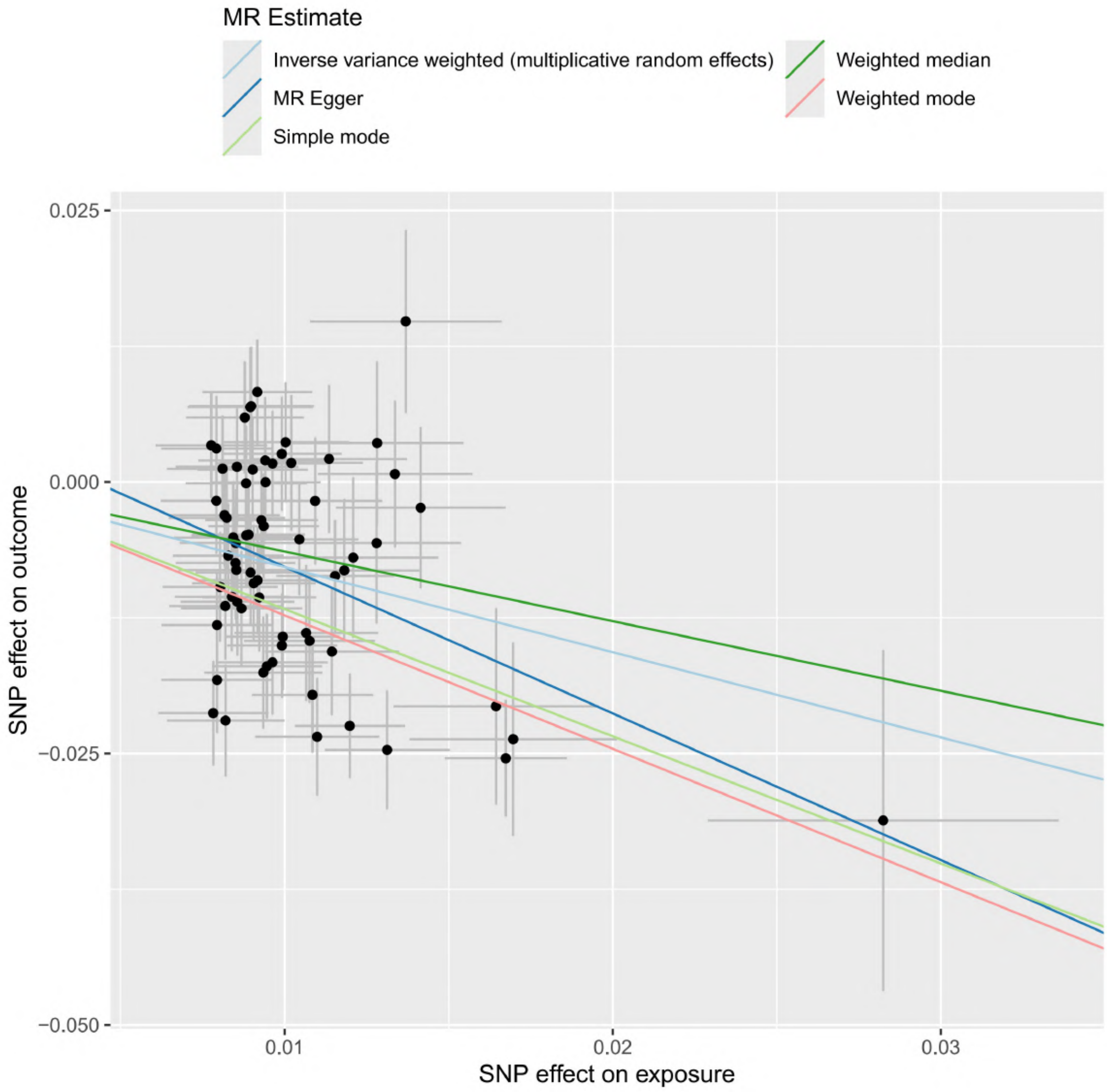

Supplementary Fig. 45 Scatter plot of the causal effect of dried fruit intake on GERD

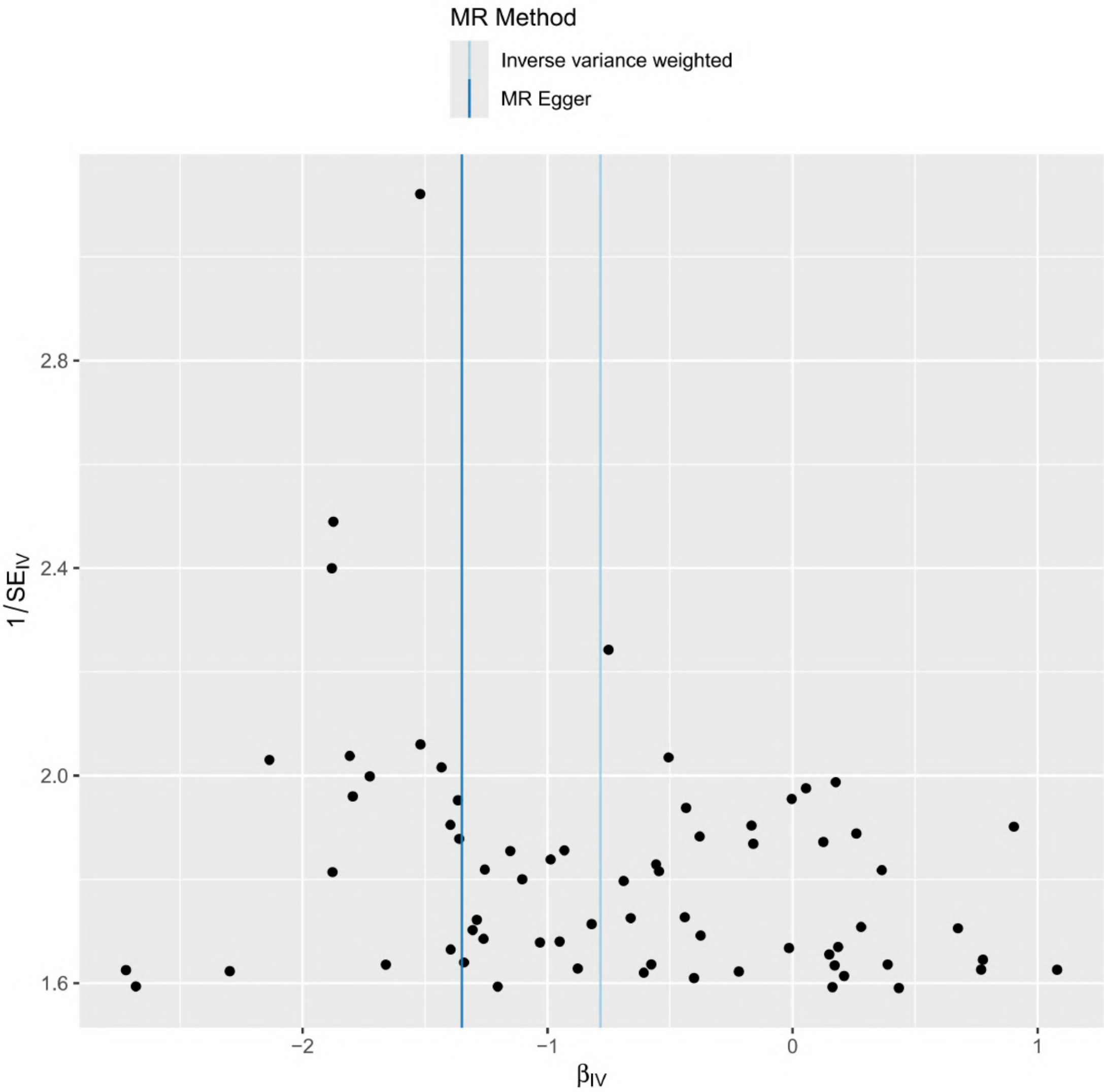

Supplementary Fig. 46 Funnel plot of the causal effect of dried fruit intake on GERD

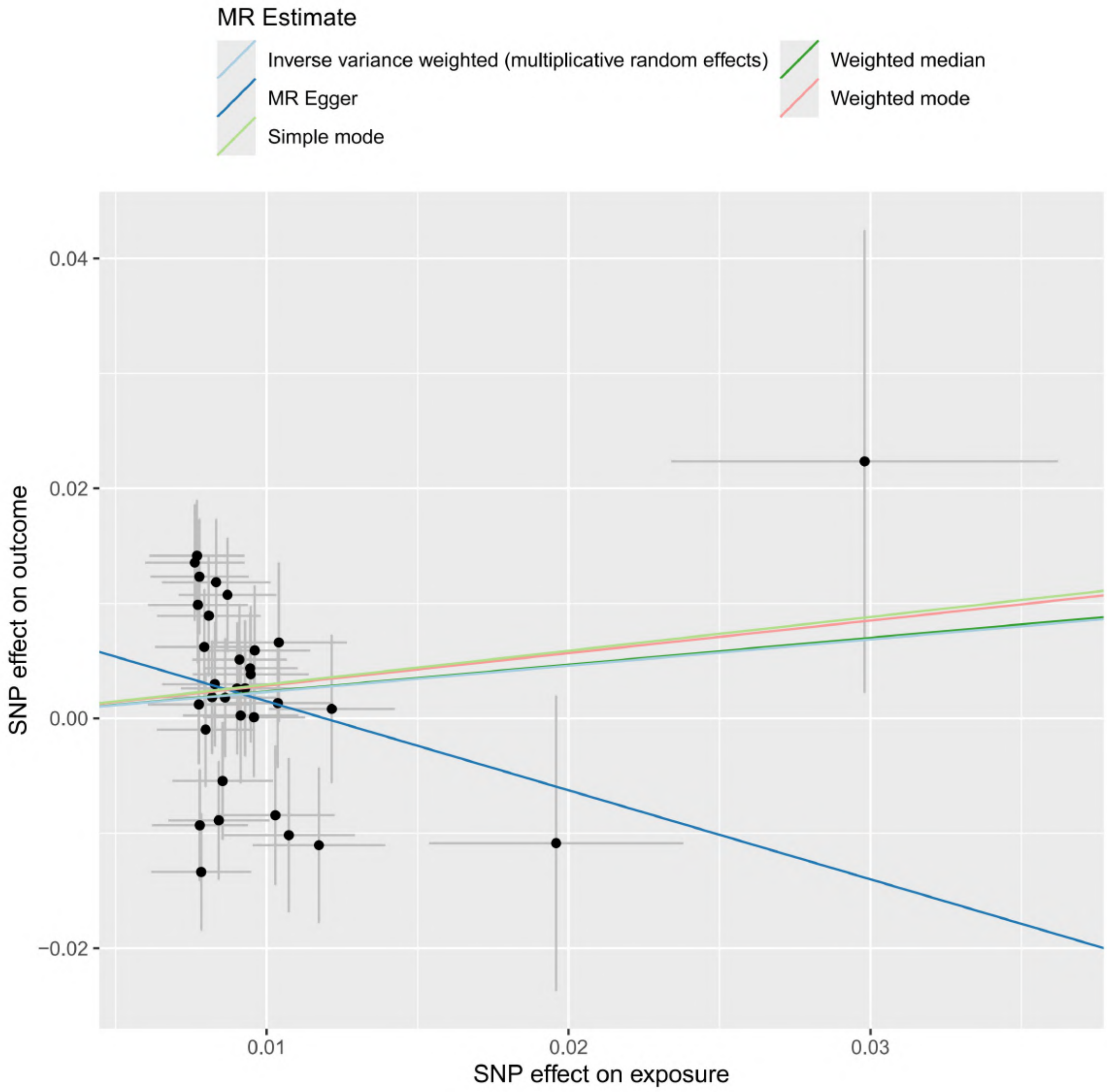

Supplementary Fig. 47 Scatter plot of the causal effect of Cooked vegetable intake on GERD

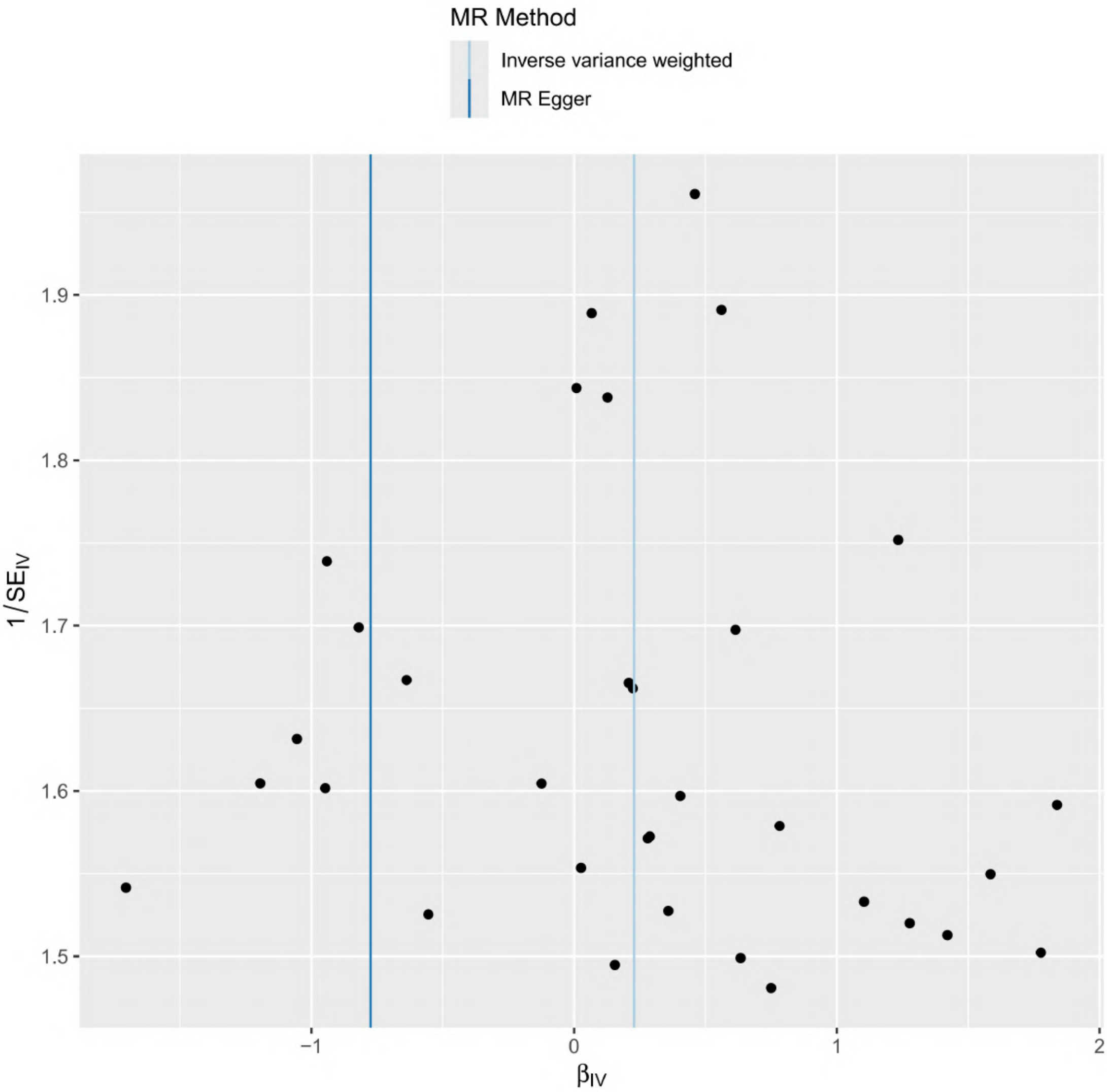

Supplementary Fig. 48 Funnel plot of the causal effect of Cooked vegetable intake on GERD

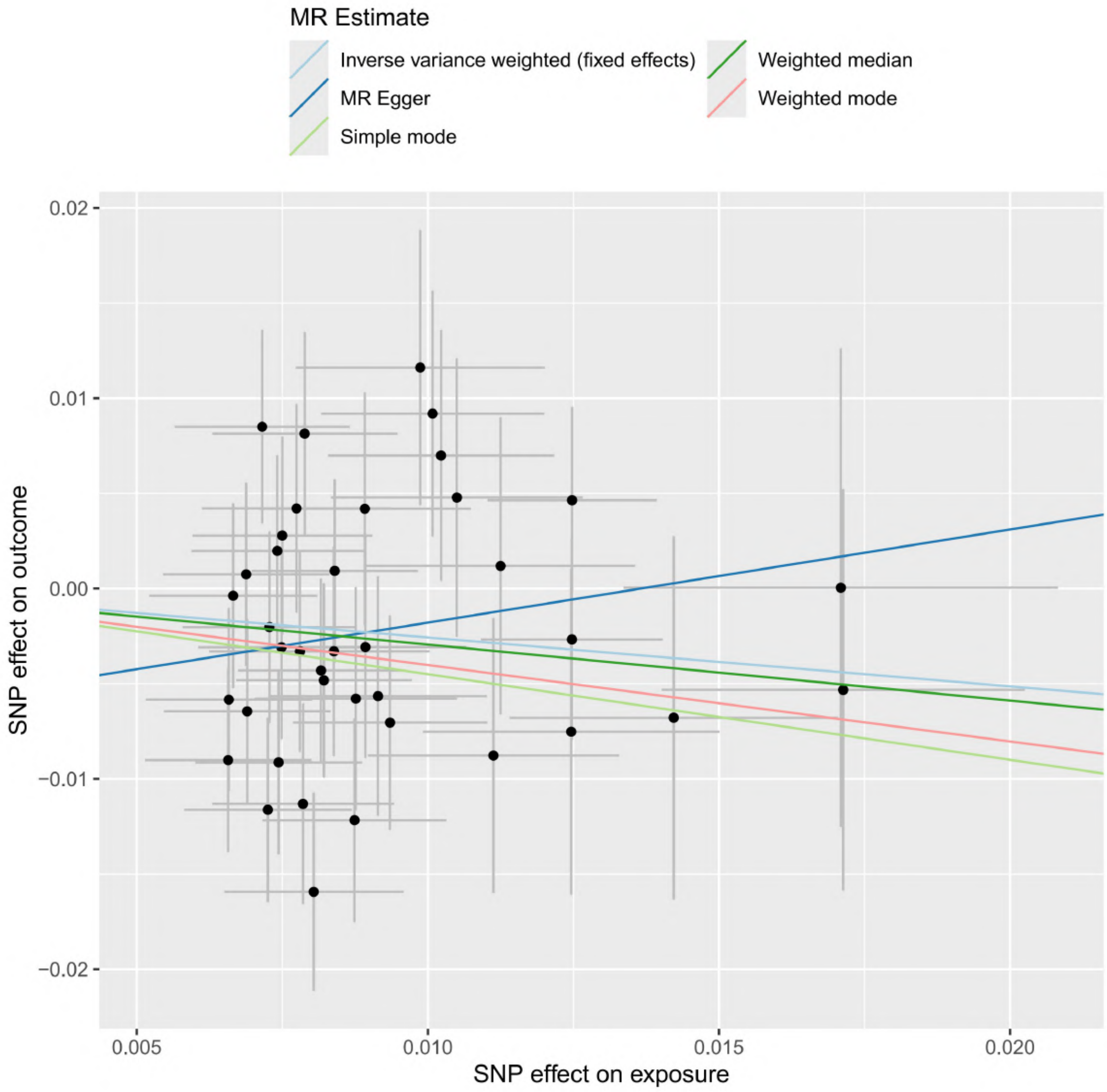

Supplementary Fig. 49 Scatter plot of the causal effect of Salad / raw vegetable intake on GERD

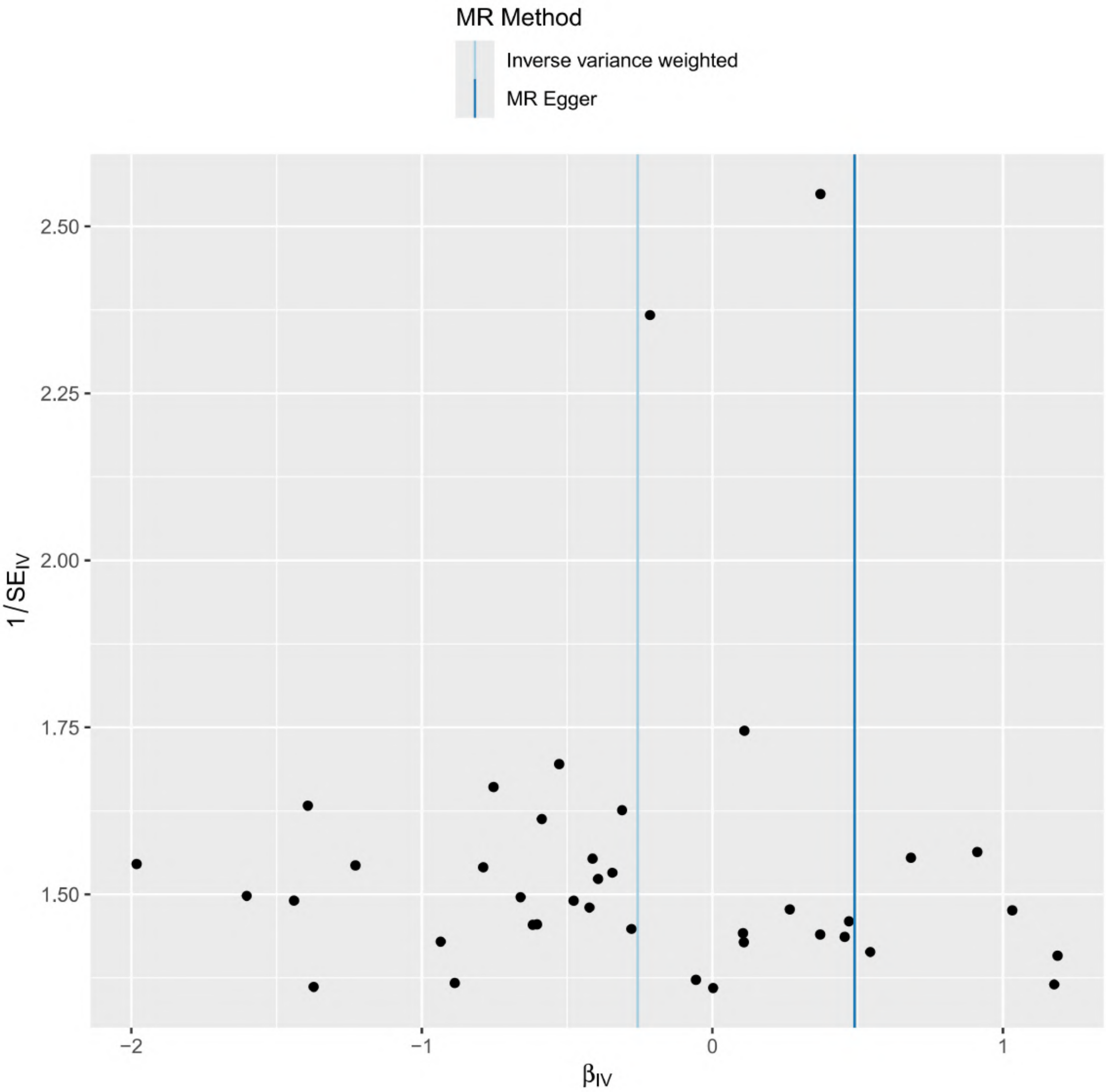

Supplementary Fig. 50 Funnel plot of the causal effect of Salad / raw vegetable intake on GERD

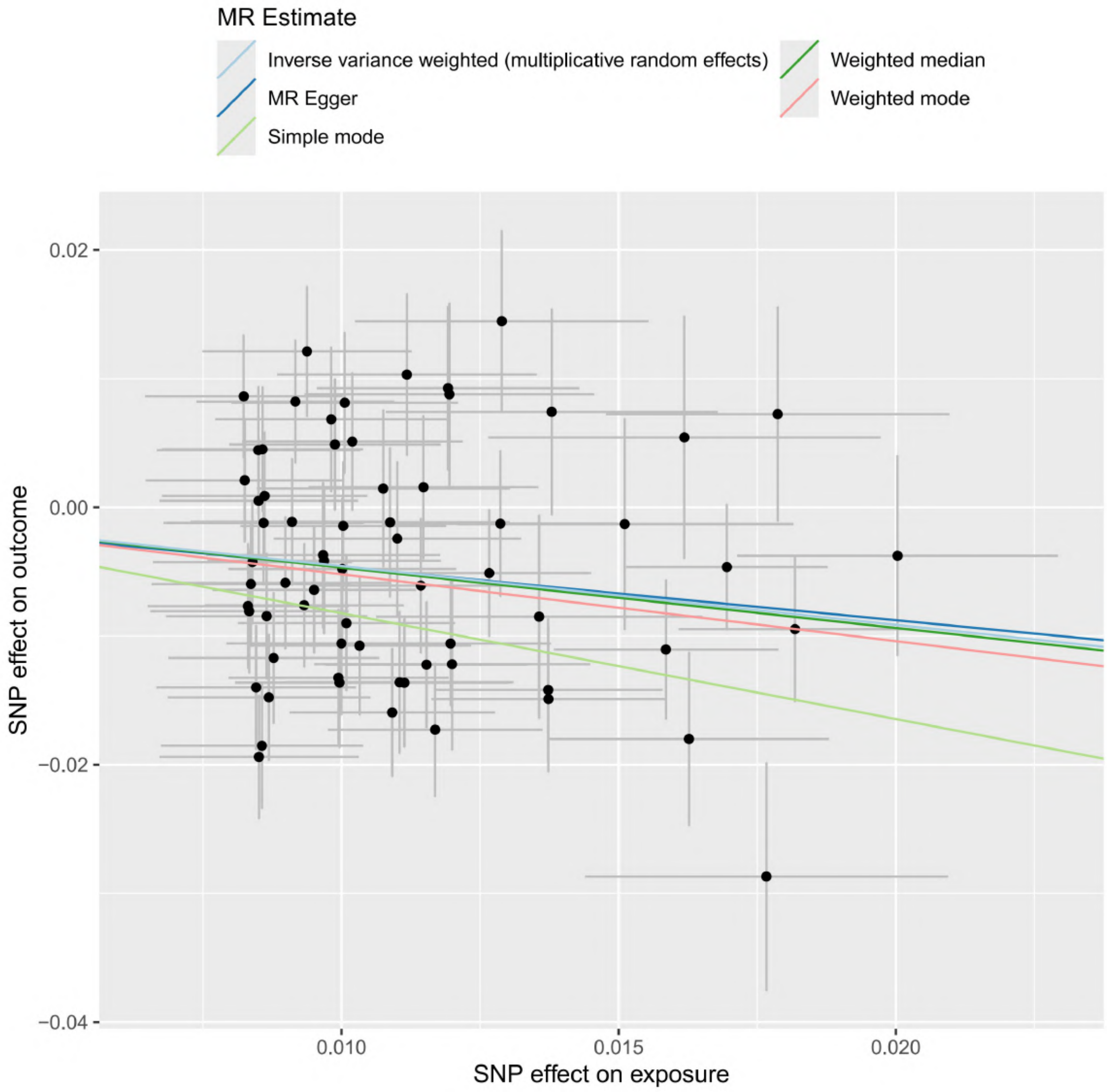

Supplementary Fig. 51 Scatter plot of the causal effect of Cereal intake on GERD

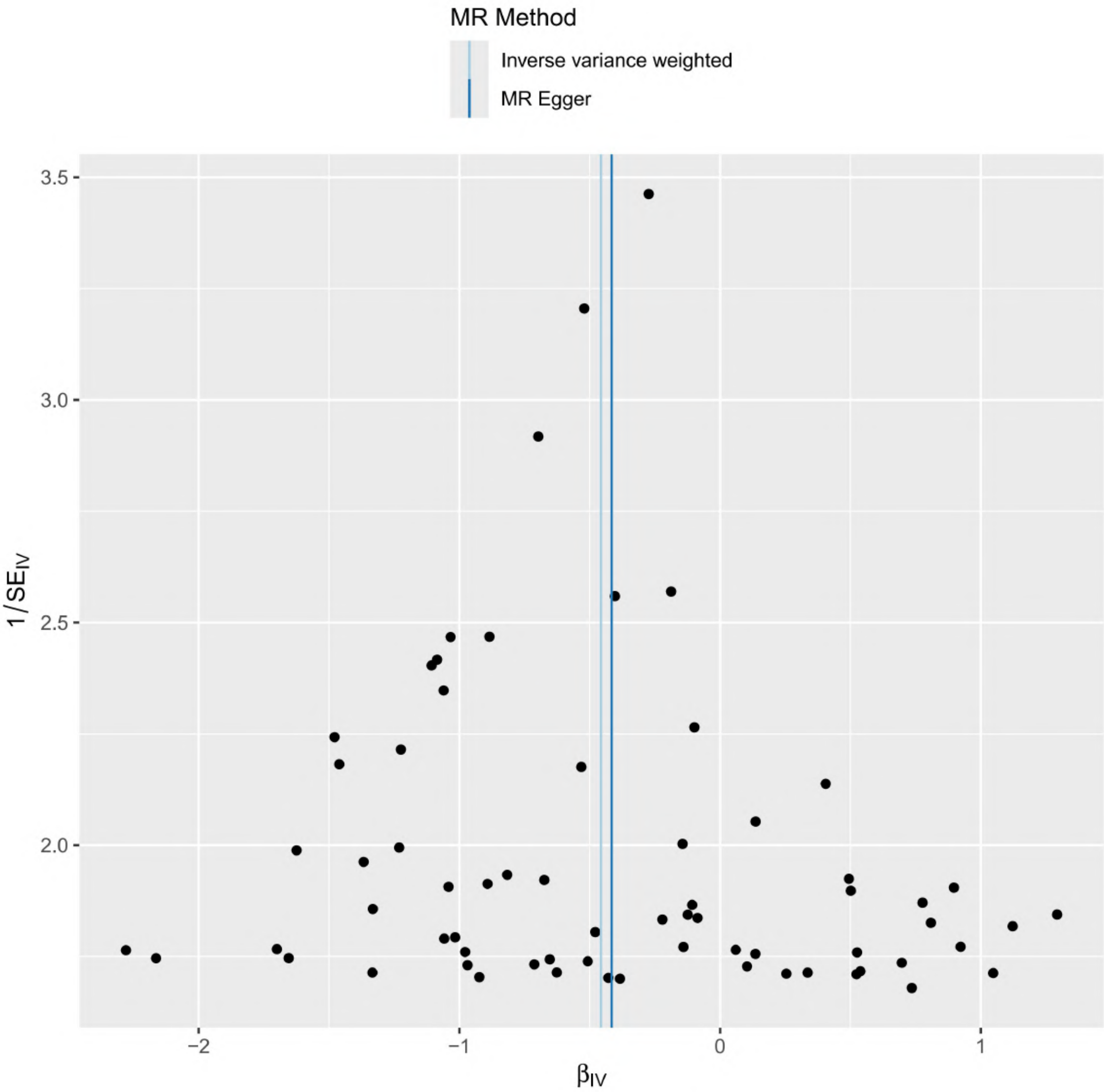

Supplementary Fig. 52 Funnel plot of the causal effect of Cereal intake on GERD

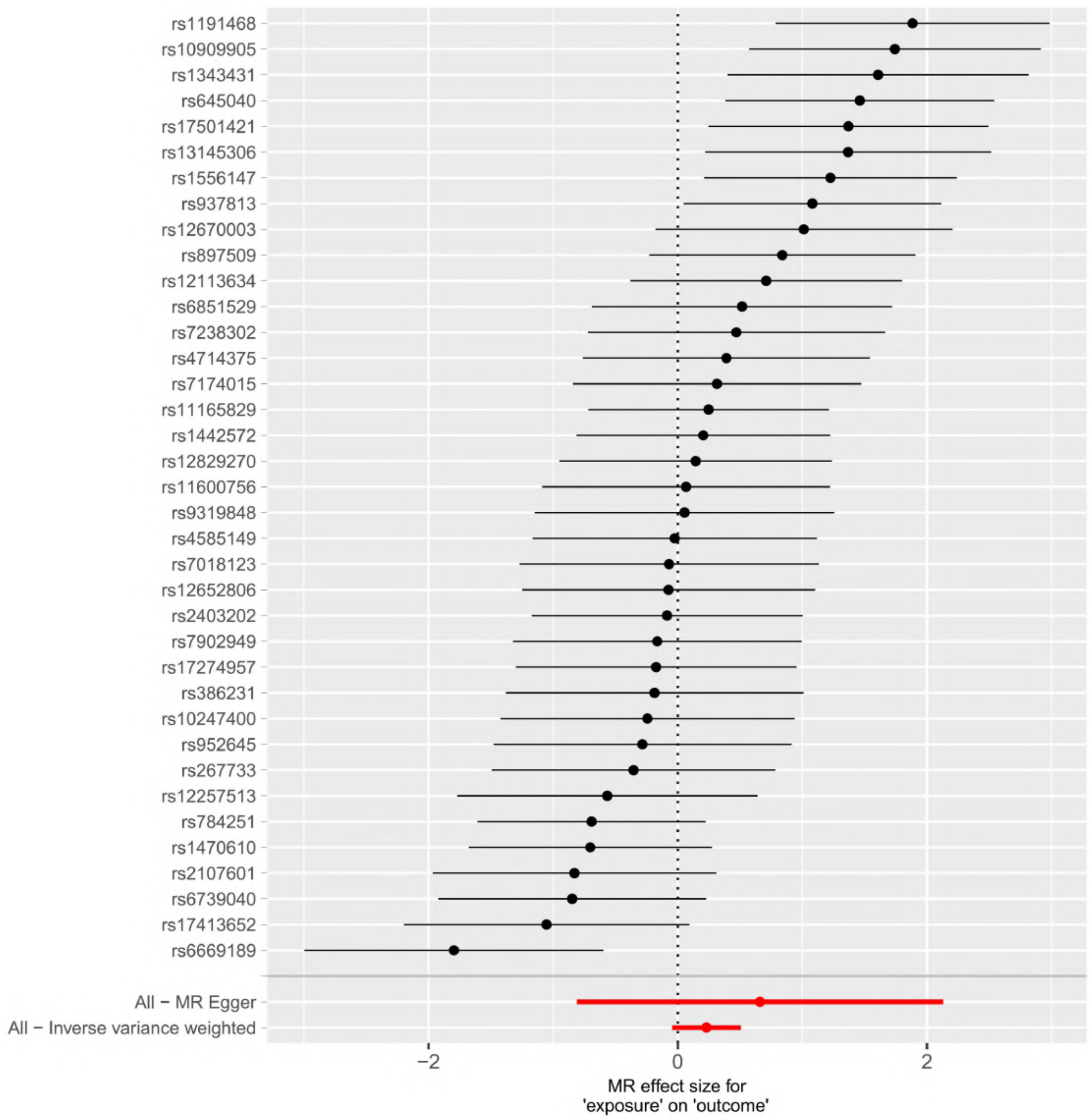

Supplementary Fig. 53 Forest plot of the causal effect of beef intake on GERD

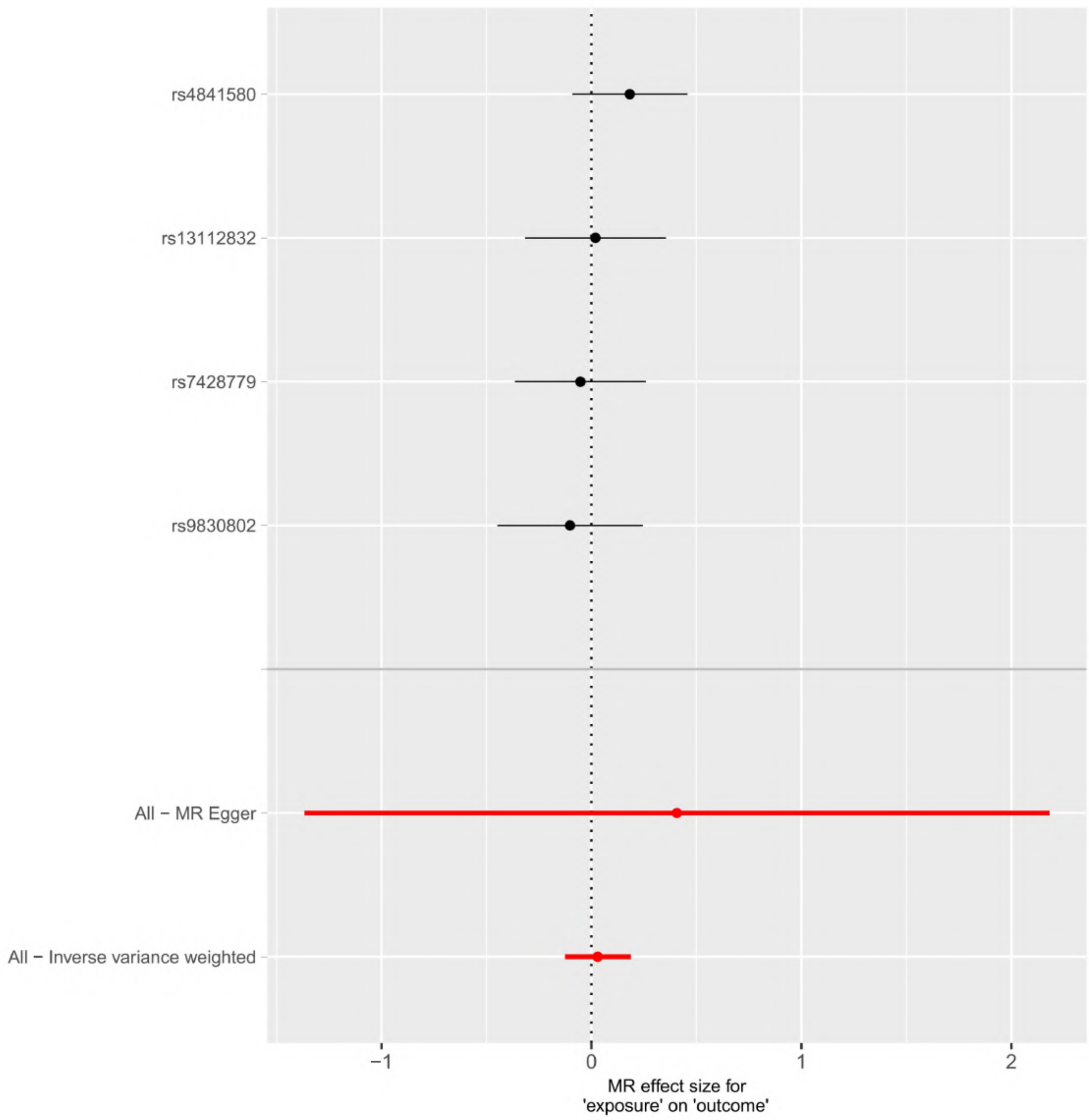

Supplementary Fig. 54 Forest plot of the causal effect of banana intake on GERD

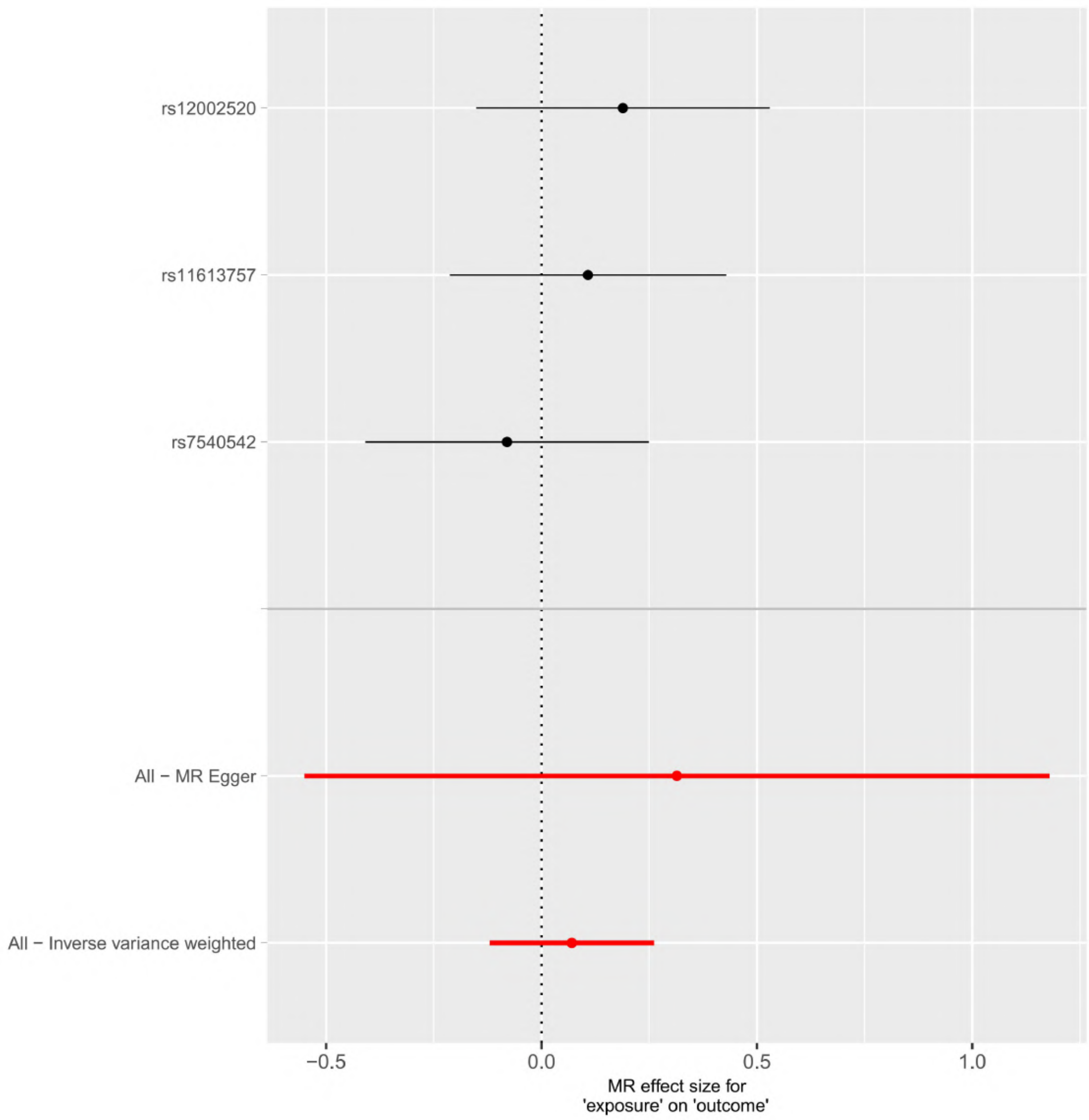

Supplementary Fig. 55 Forest plot of the causal effect of broccoli intake on GERD

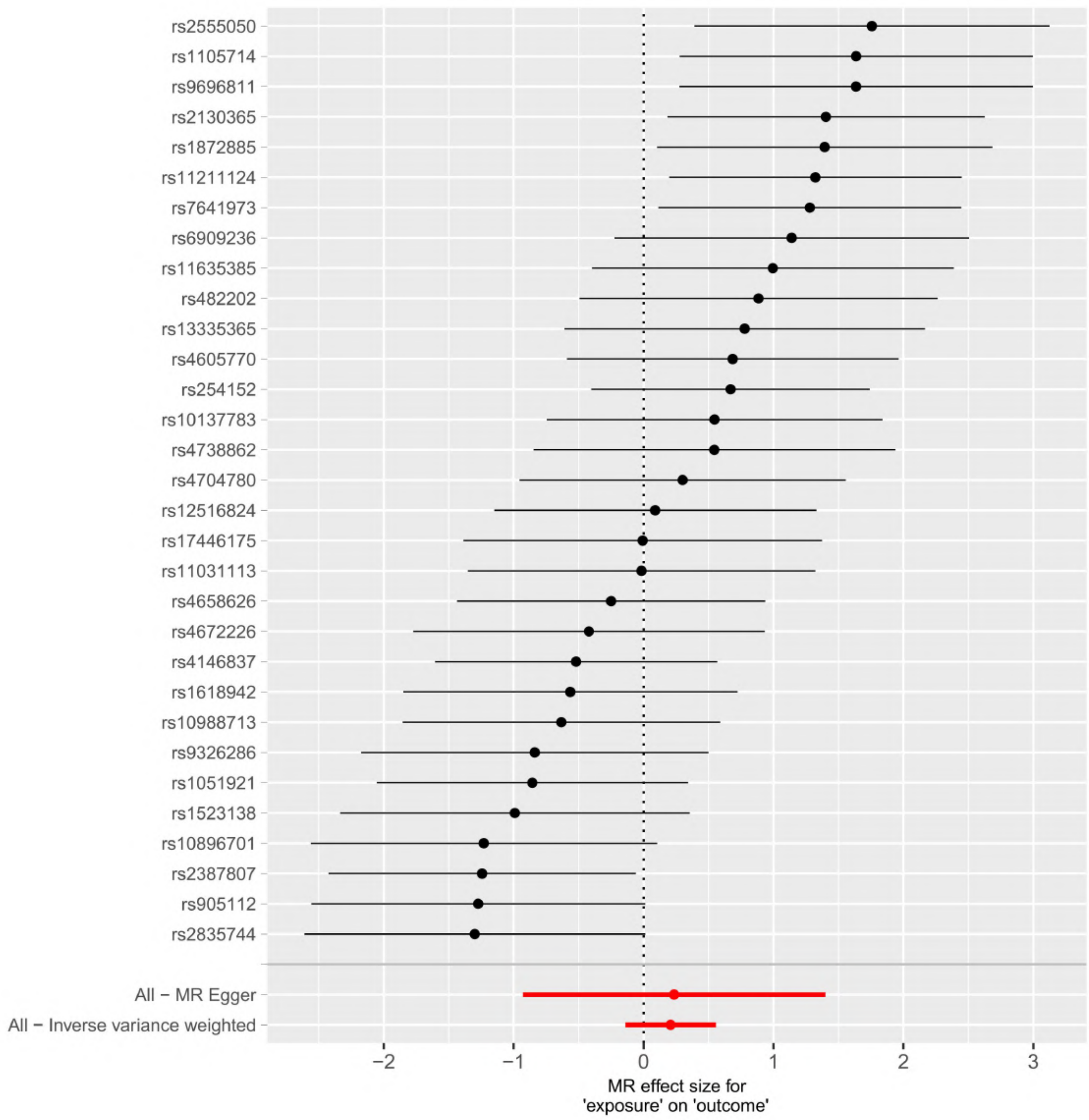

Supplementary Fig. 56 Forest plot of the causal effect of pork intake on GERD

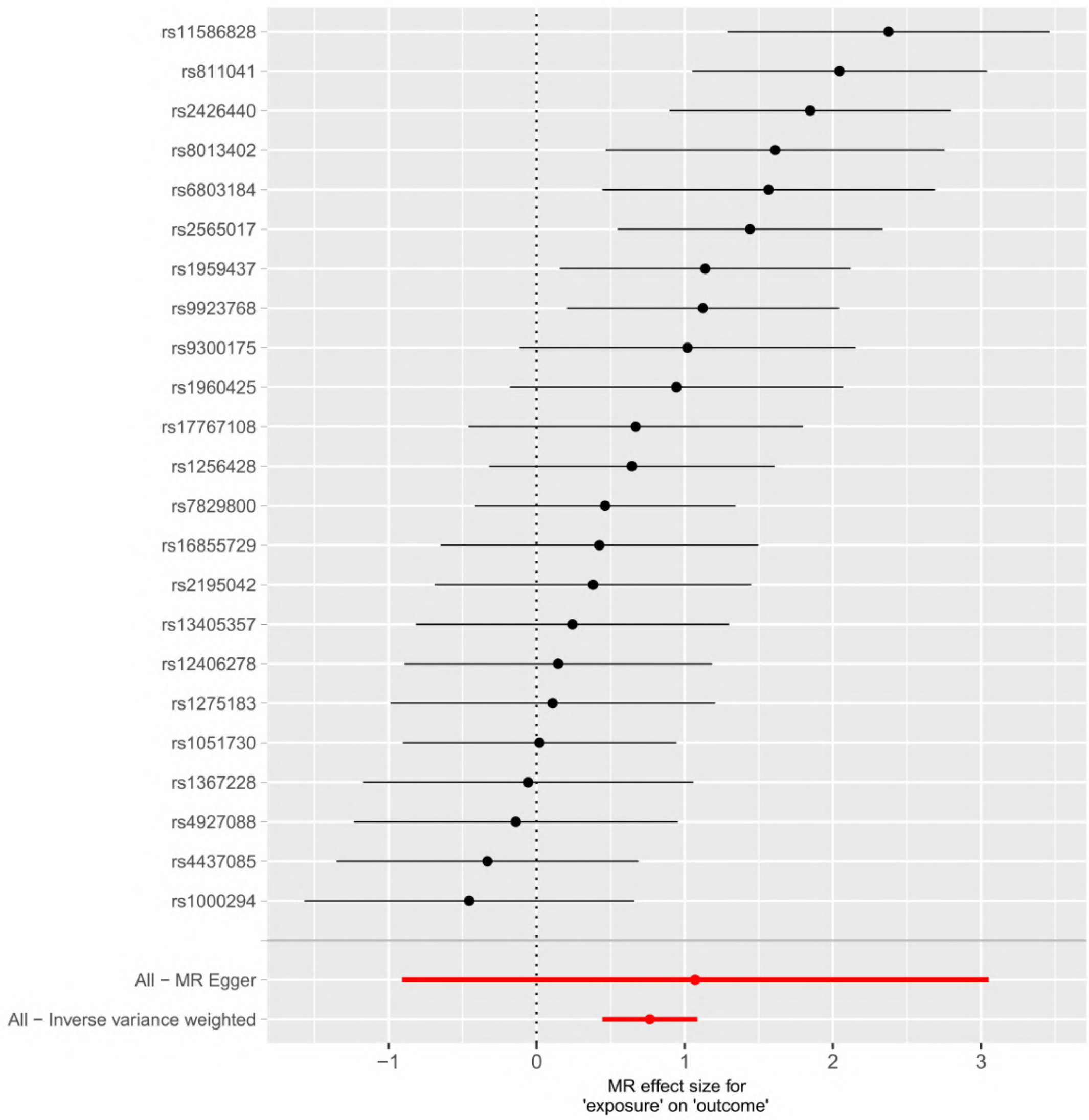

Supplementary Fig. 57 Forest plot of the causal effect of pourtry intake on GERD

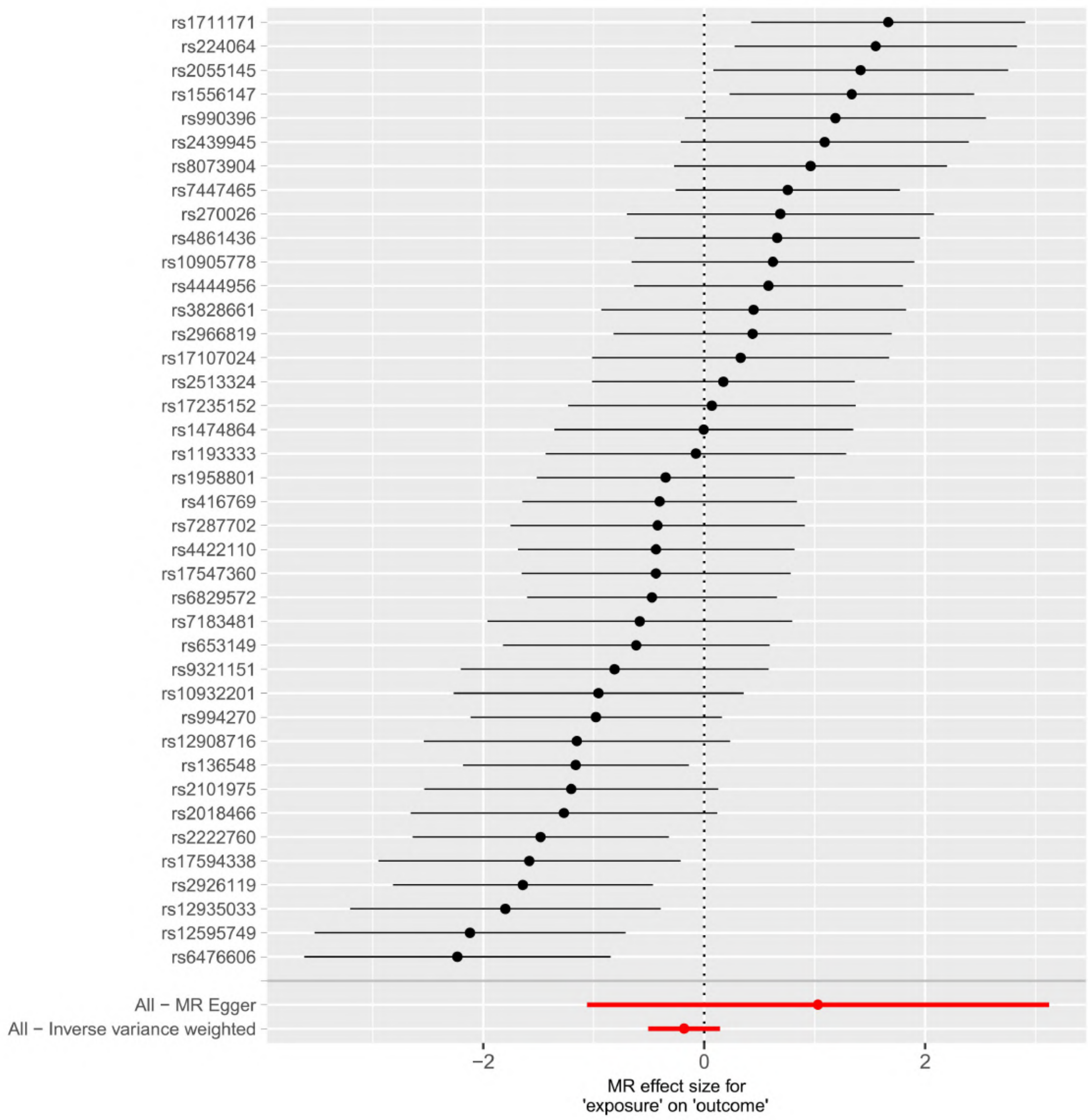

Supplementary Fig. 58 Forest plot of the causal effect of Lamb/mutton intake on GERD

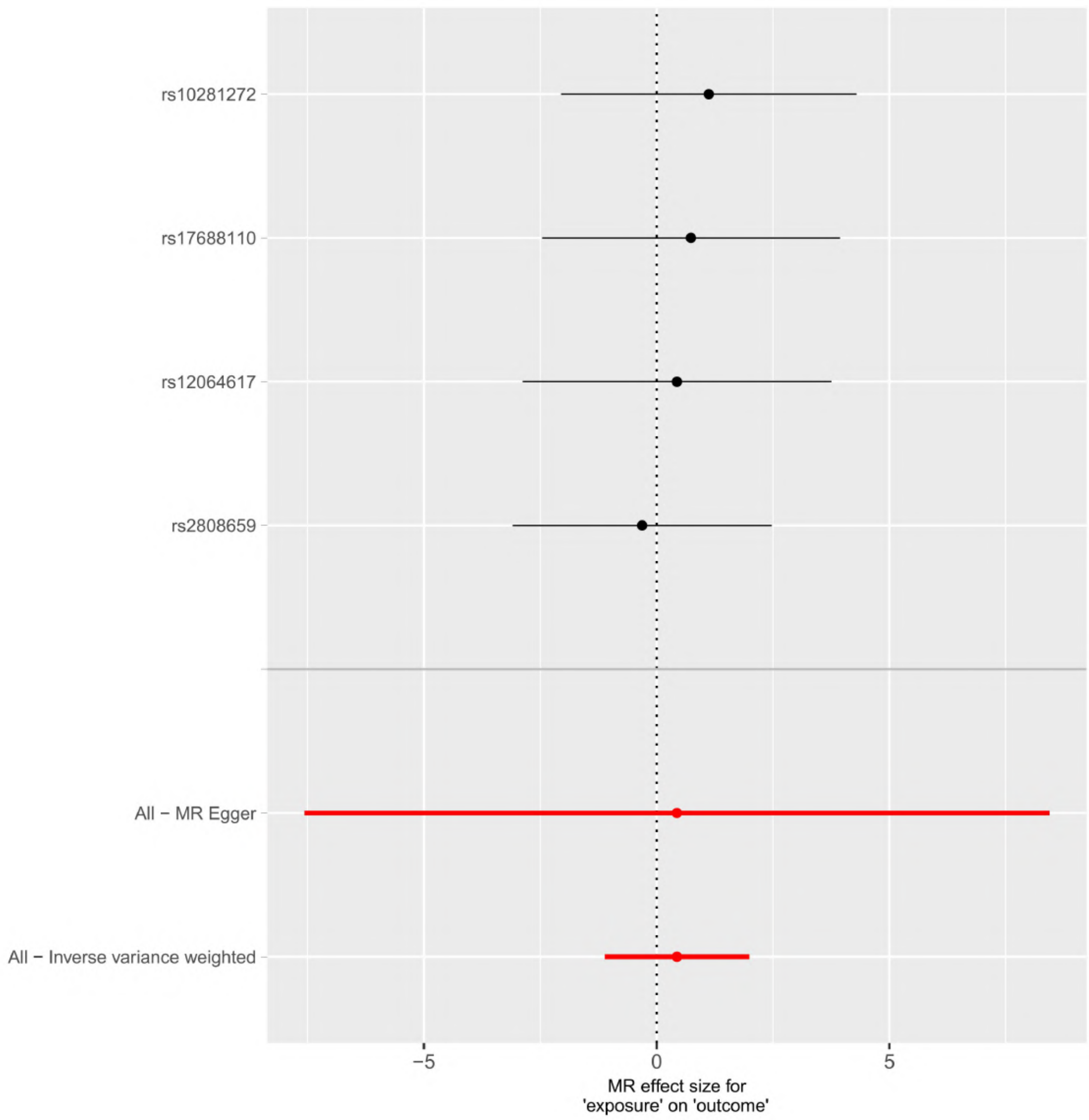

Supplementary Fig. 59 Forest plot of the causal effect of Lobster/crab intake on GERD

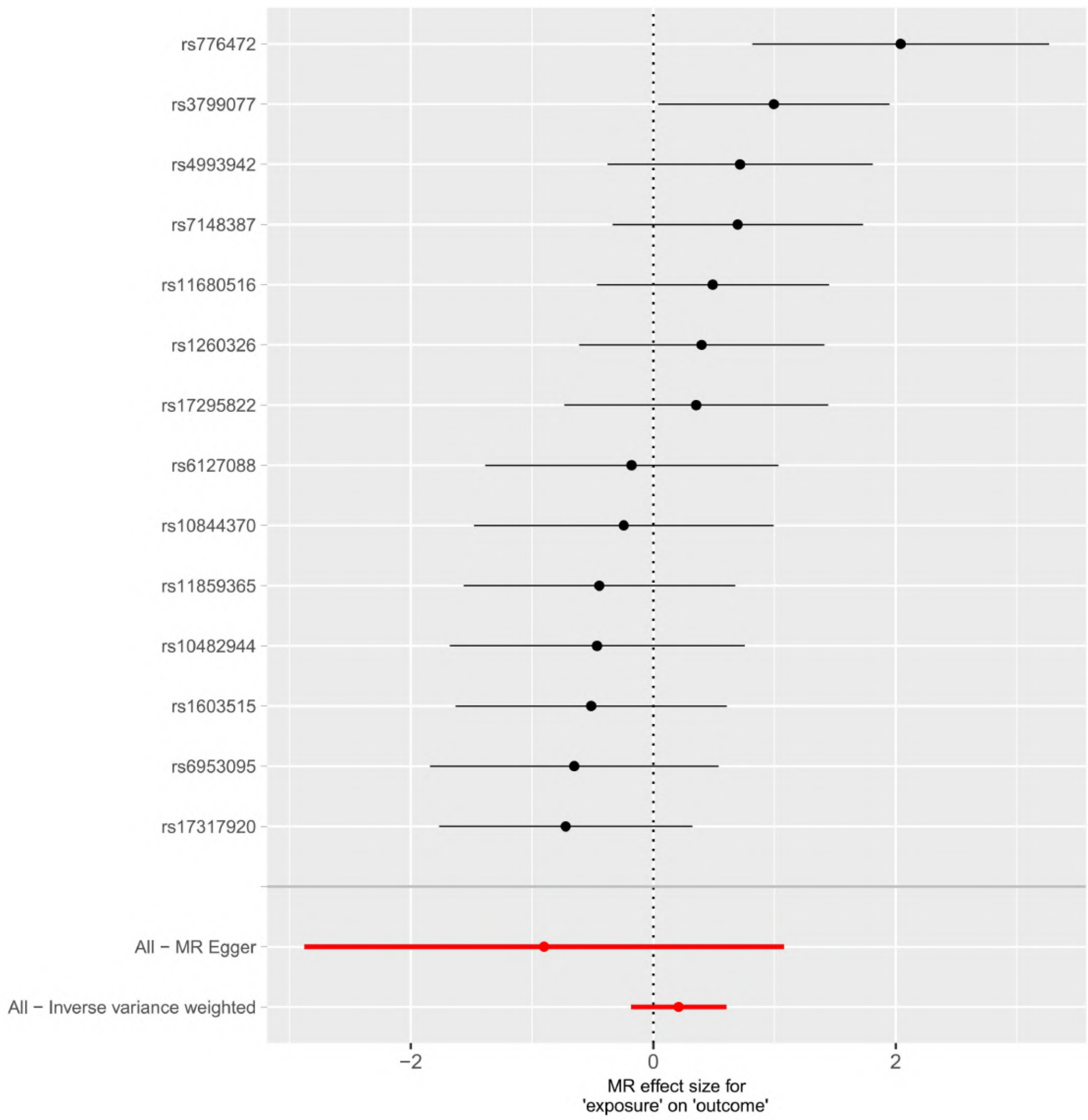

Supplementary Fig. 60 Forest plot of the causal effect of Non-oily fish intake on GERD

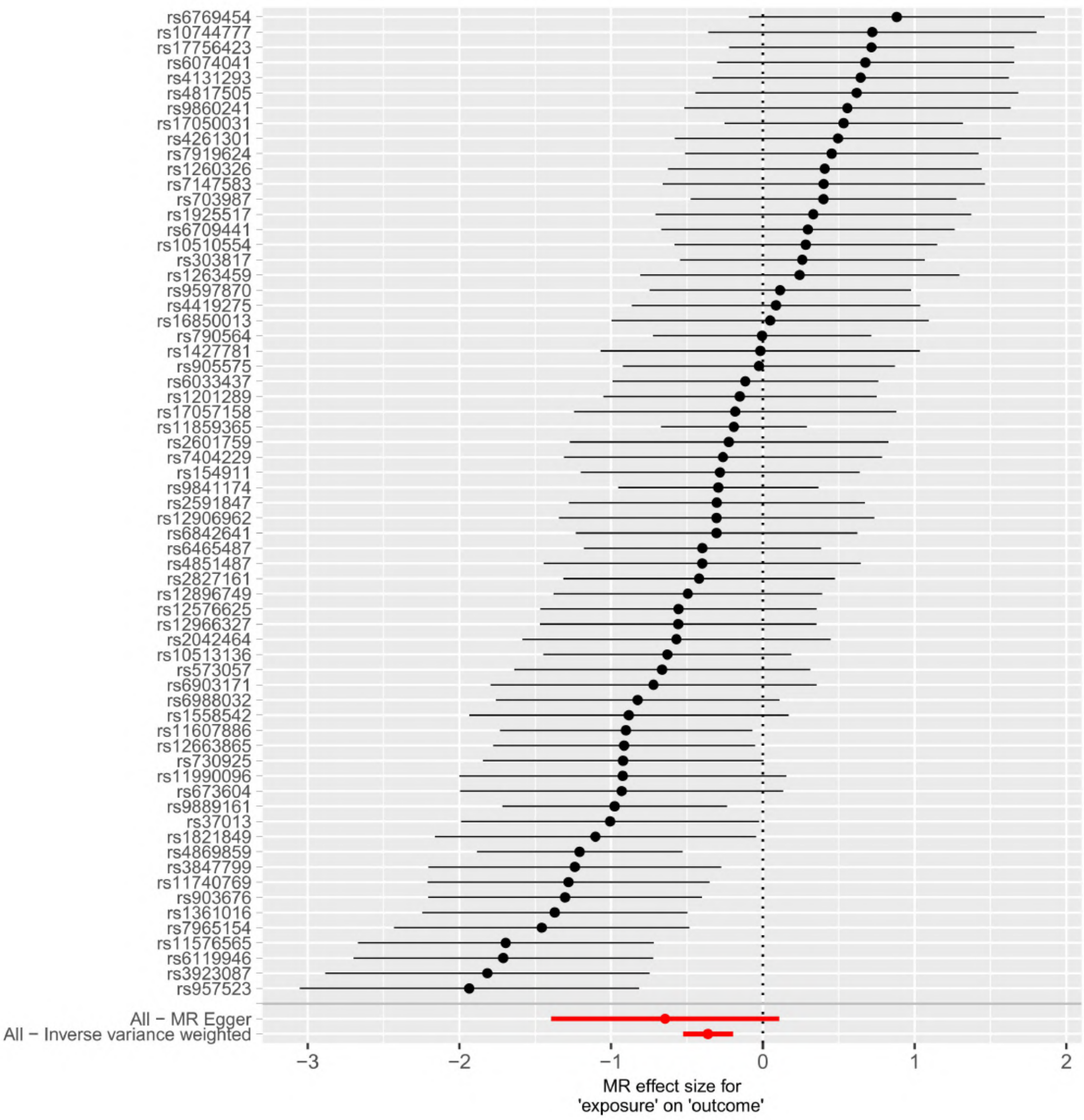

Supplementary Fig. 61 Forest plot of the causal effect of oily fish intake on GERD

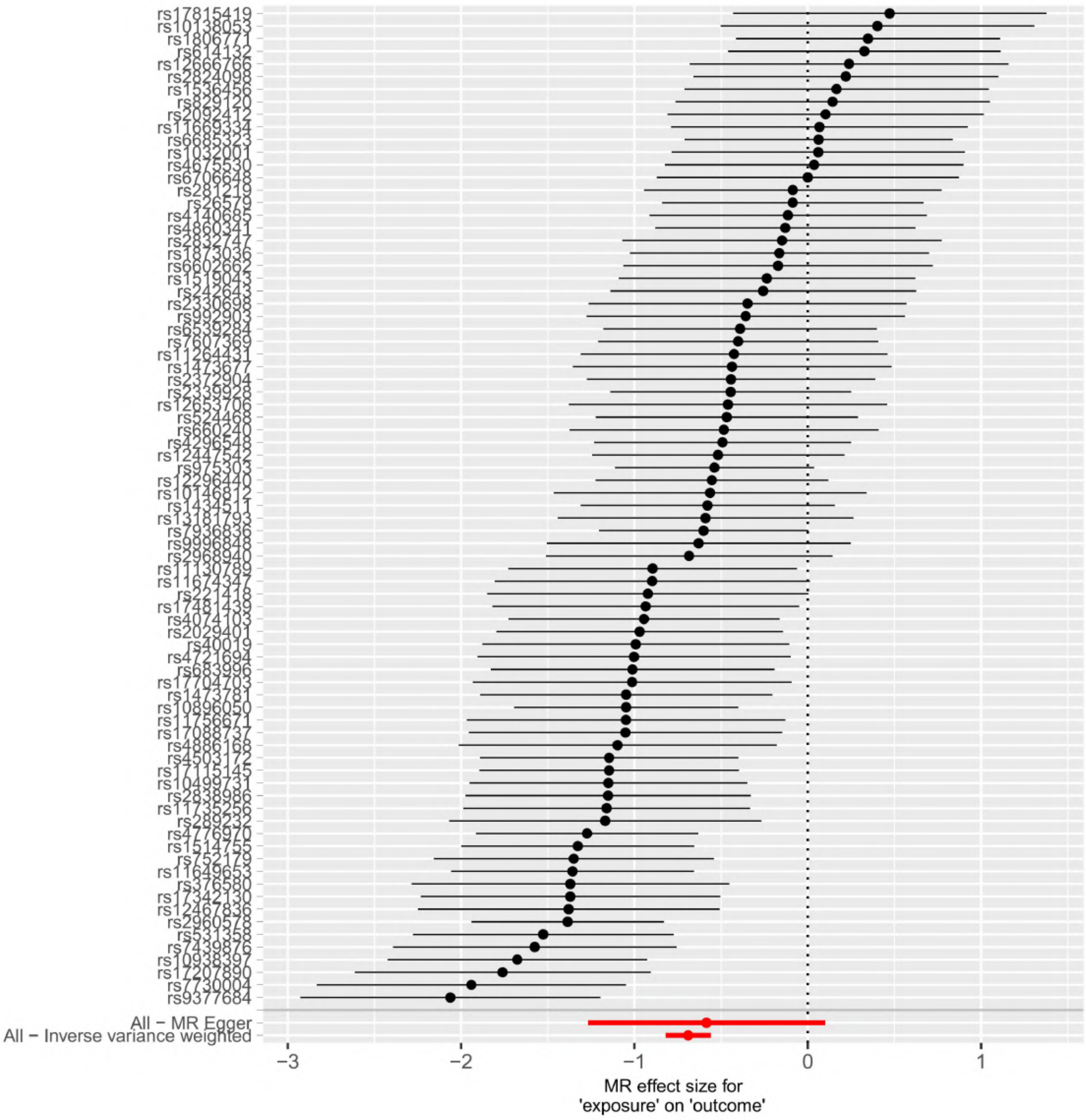

Supplementary Fig. 62 Forest plot of the causal effect of cheese intake on GERD

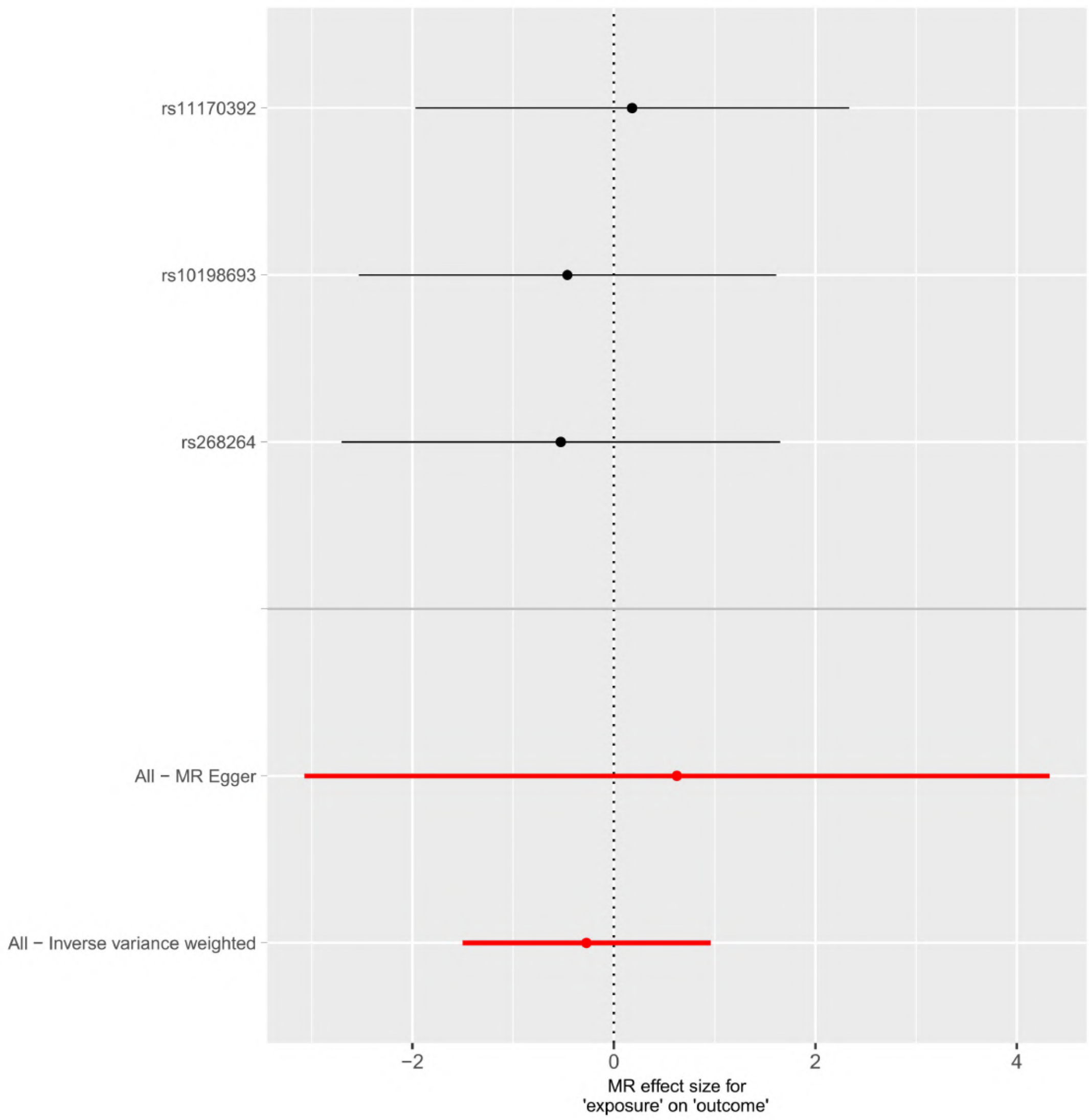

Supplementary Fig. 63 Forest plot of the causal effect of feta intake on GERD

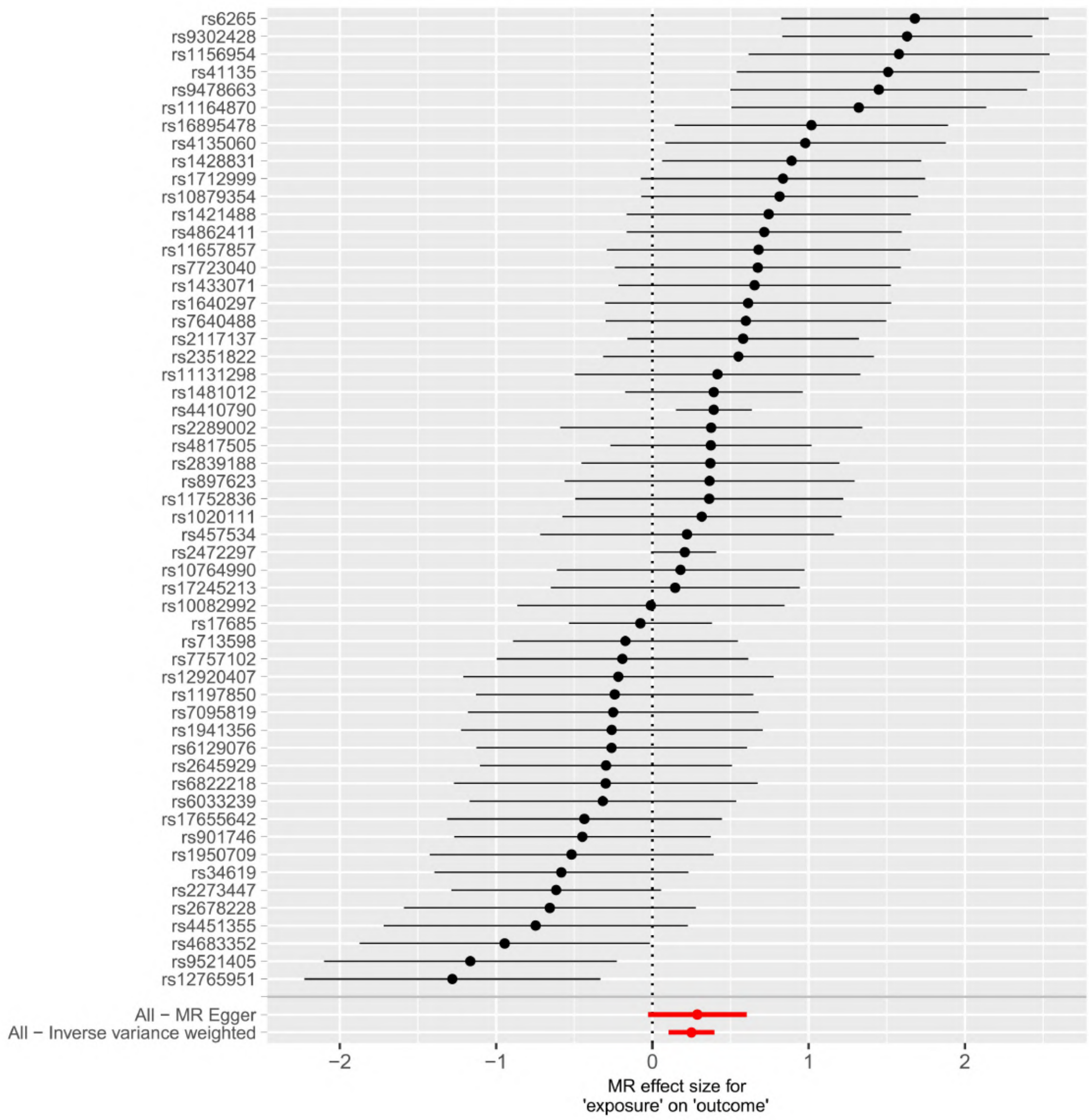

Supplementary Fig. 64 Forest plot of the causal effect of tea intake on GERD

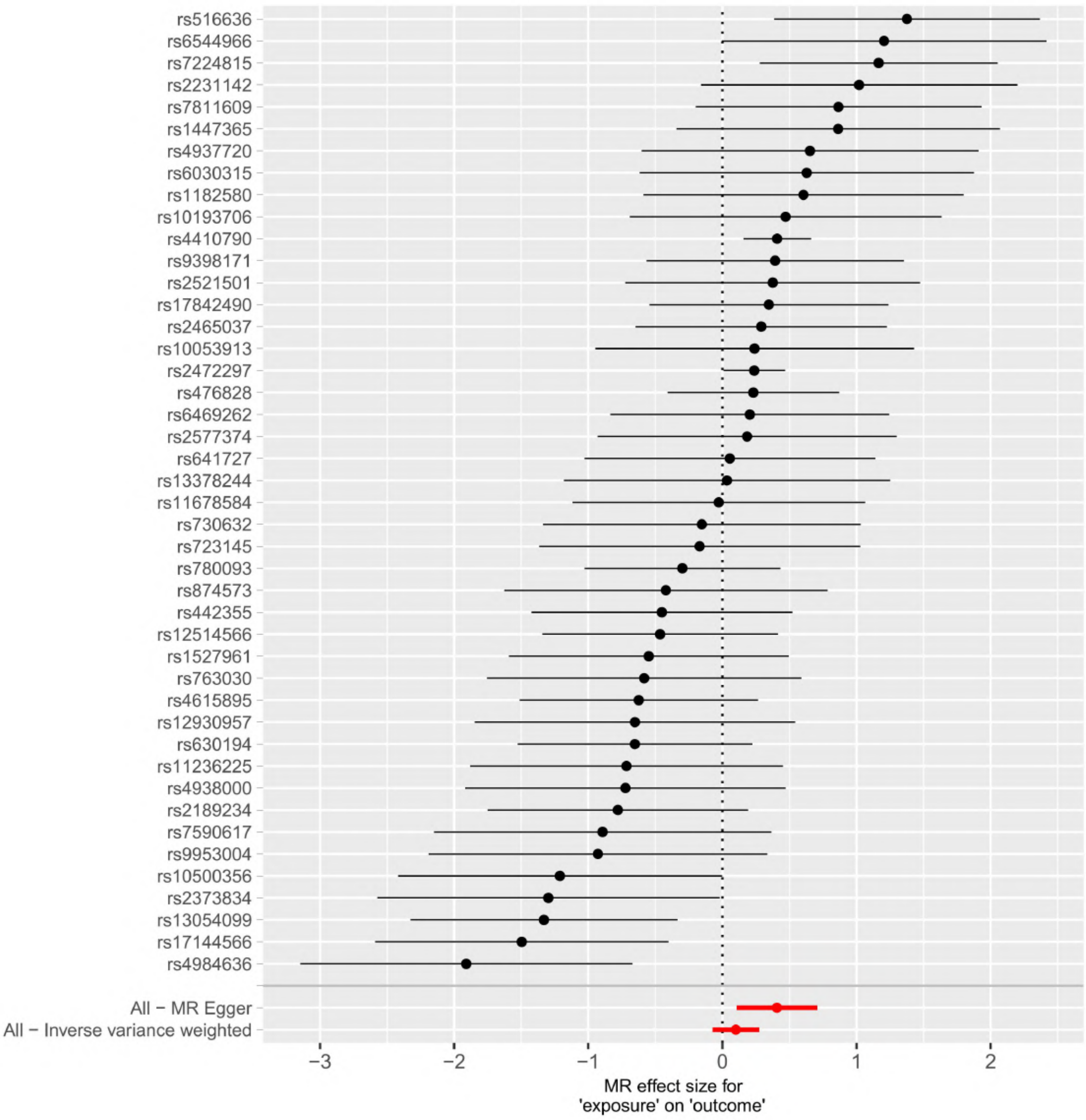

Supplementary Fig. 65 Forest plot of the causal effect of coffee intake on GERD

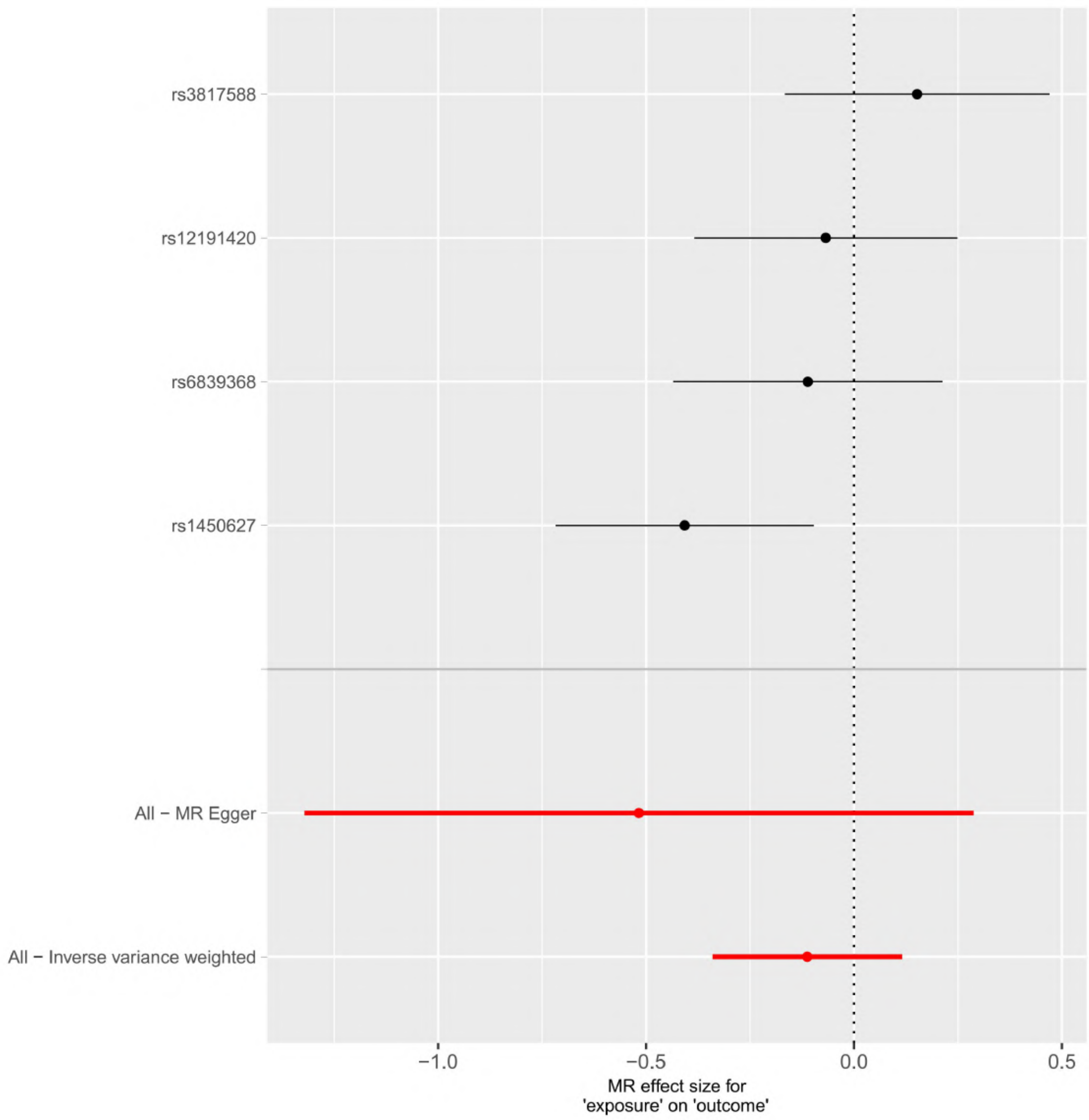

Supplementary Fig. 66 Forest plot of the causal effect of beer/cider intake on GERD

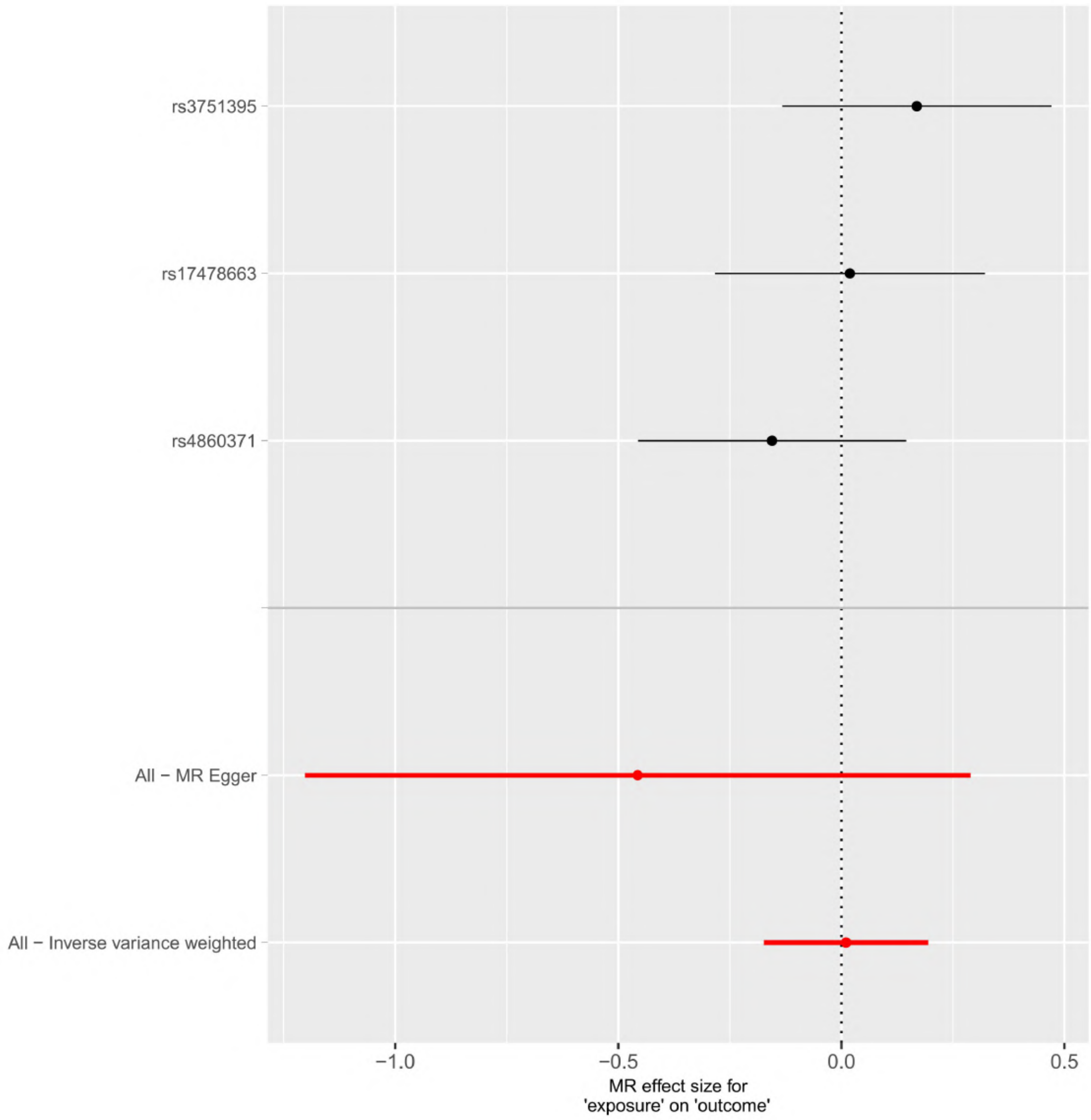

Supplementary Fig. 67 Forest plot of the causal effect of red wine intake on GERD

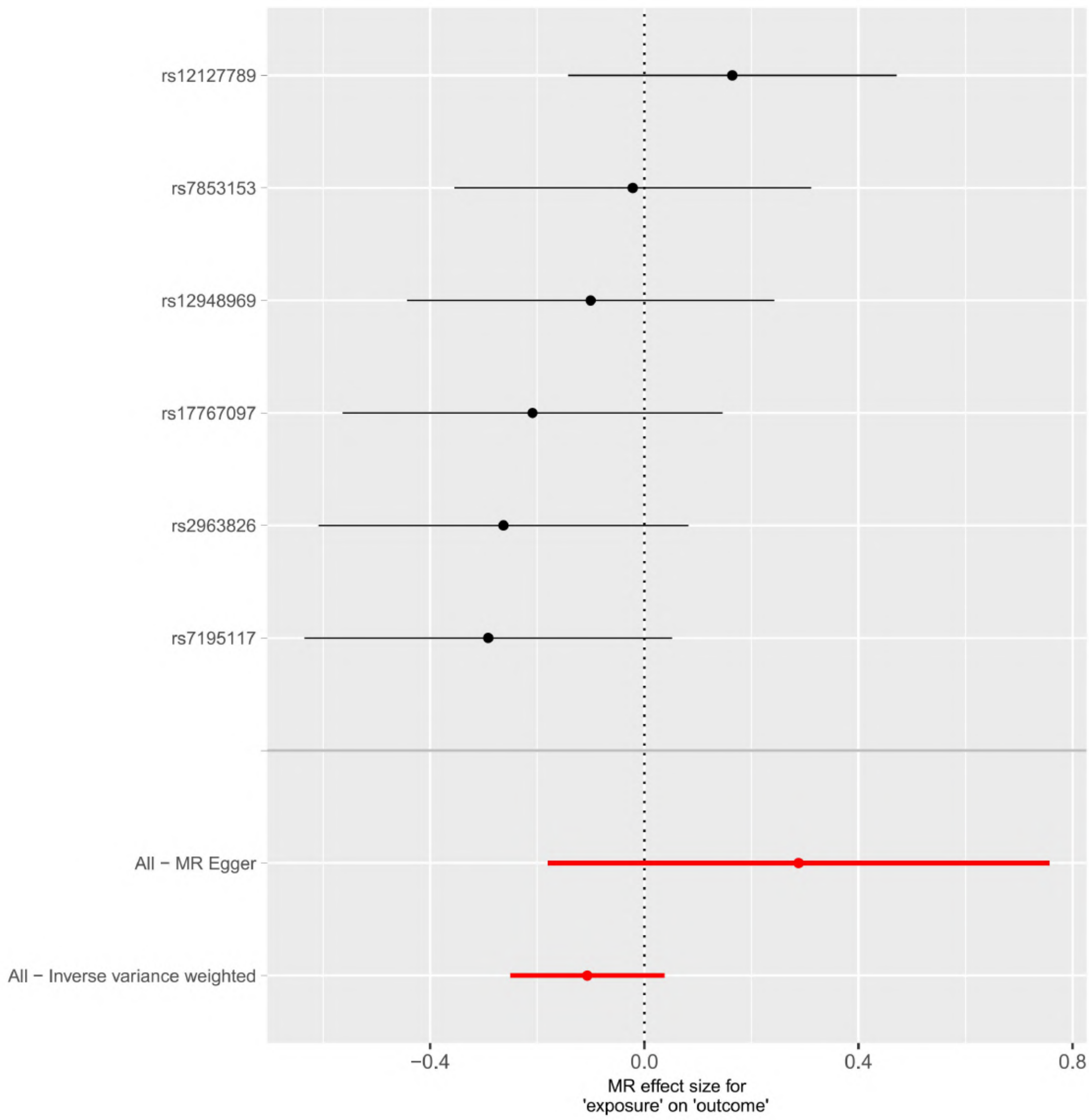

Supplementary Fig. 68 Forest plot of the causal effect of apple intake on GERD

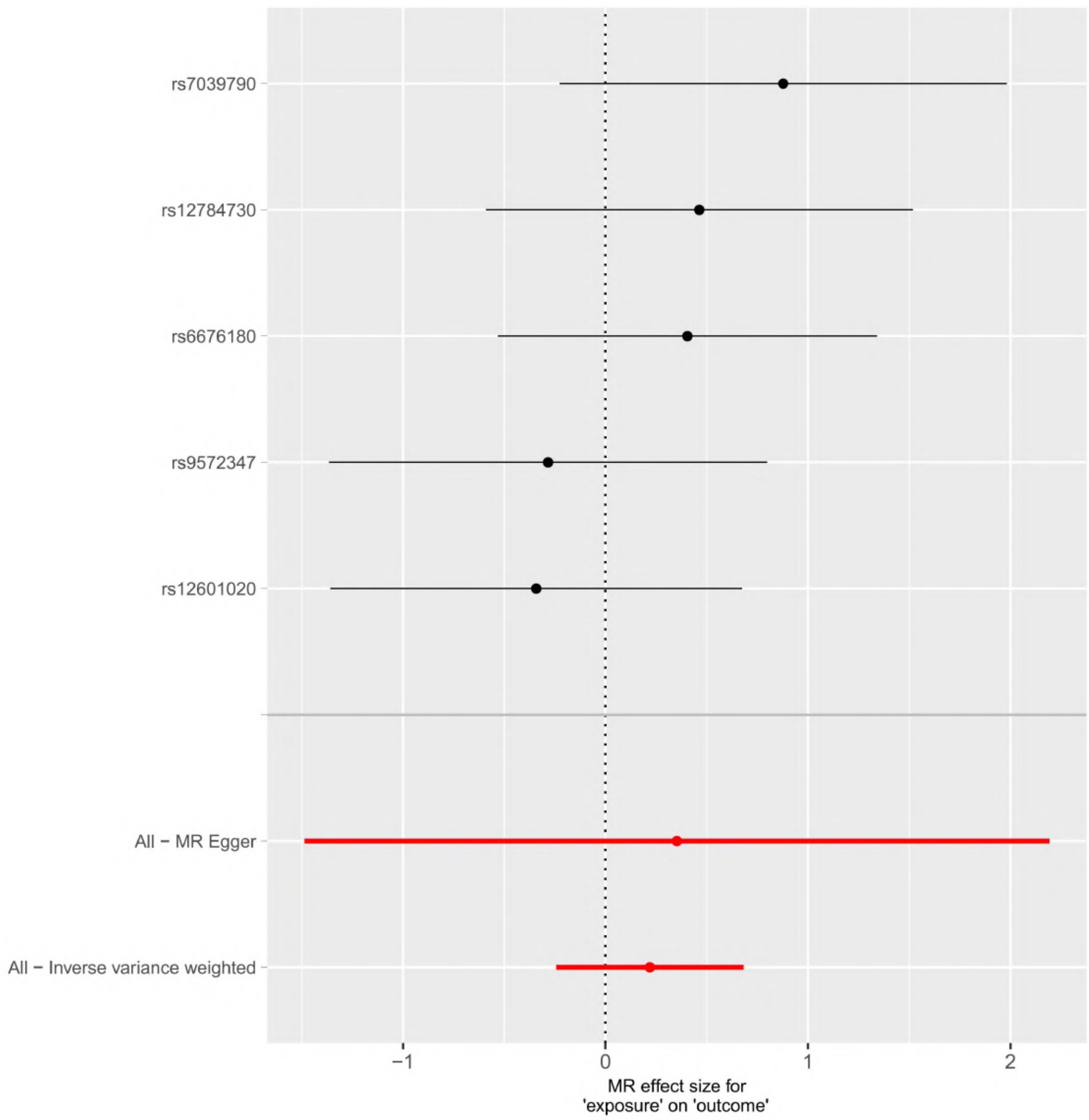

Supplementary Fig. 69 Forest plot of the causal effect of cheery intake on GERD

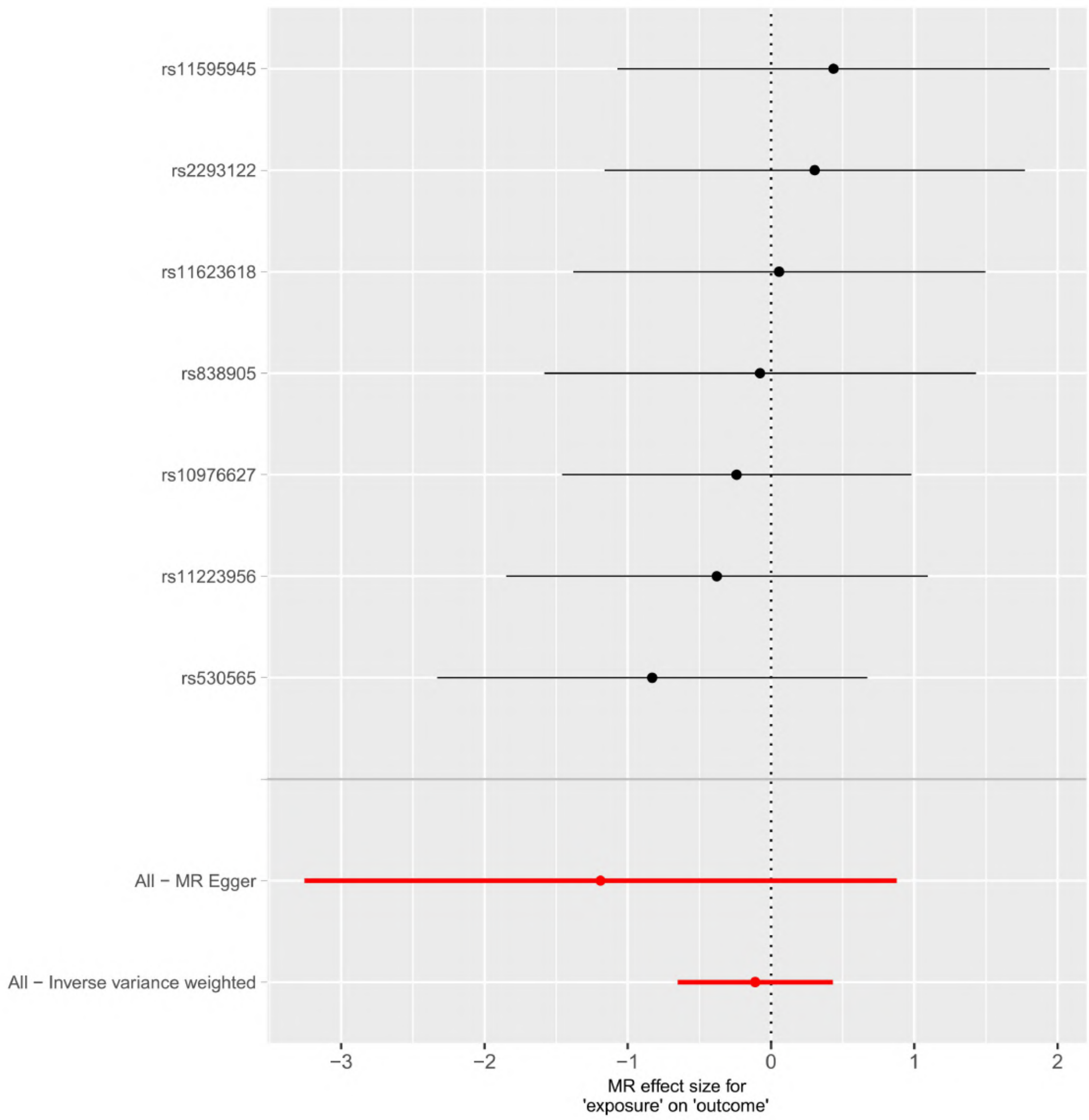

Supplementary Fig. 70 Forest plot of the causal effect of mango intake on GERD

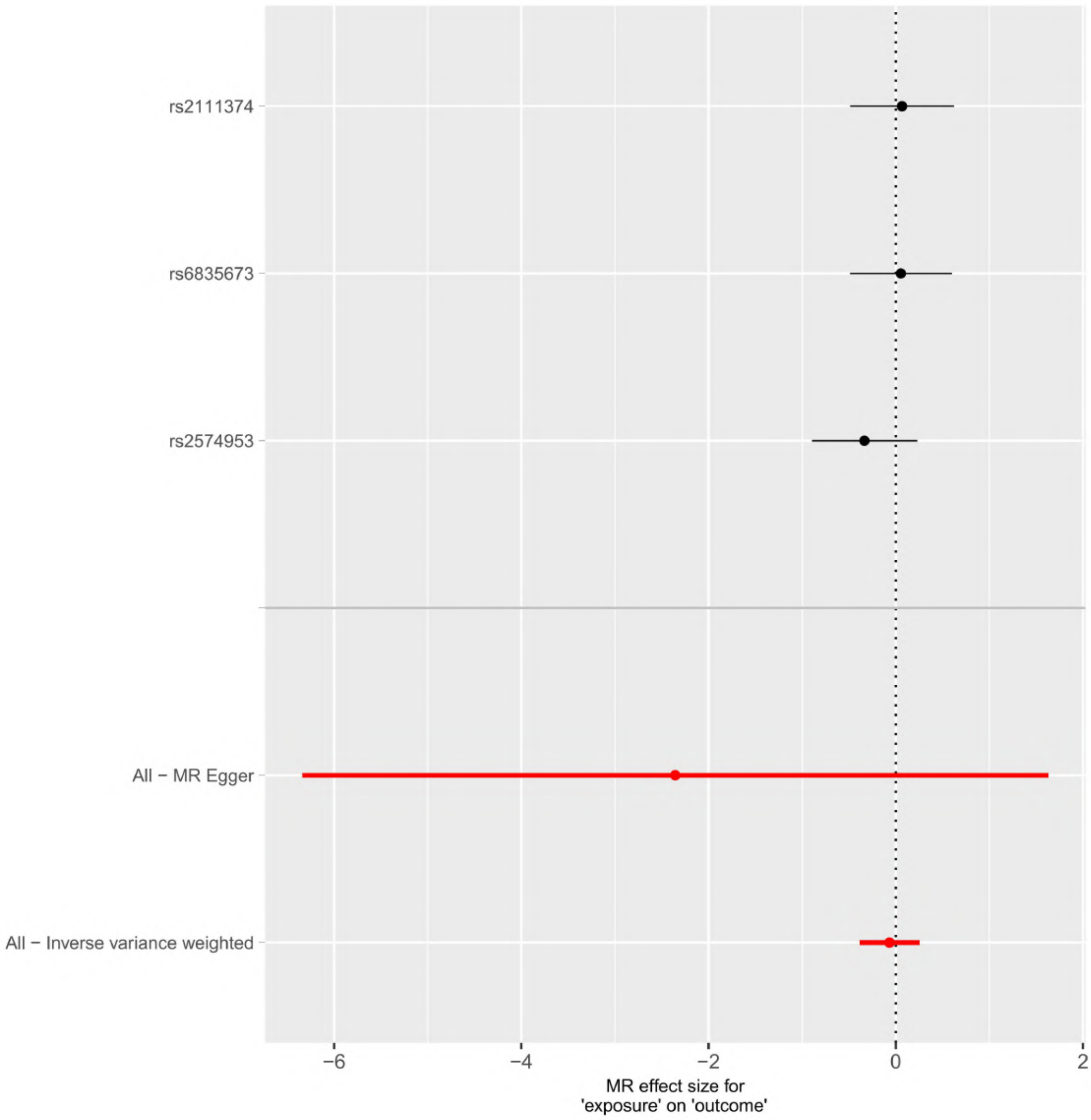

Supplementary Fig. 71 Forest plot of the causal effect of garlic intake on GERD

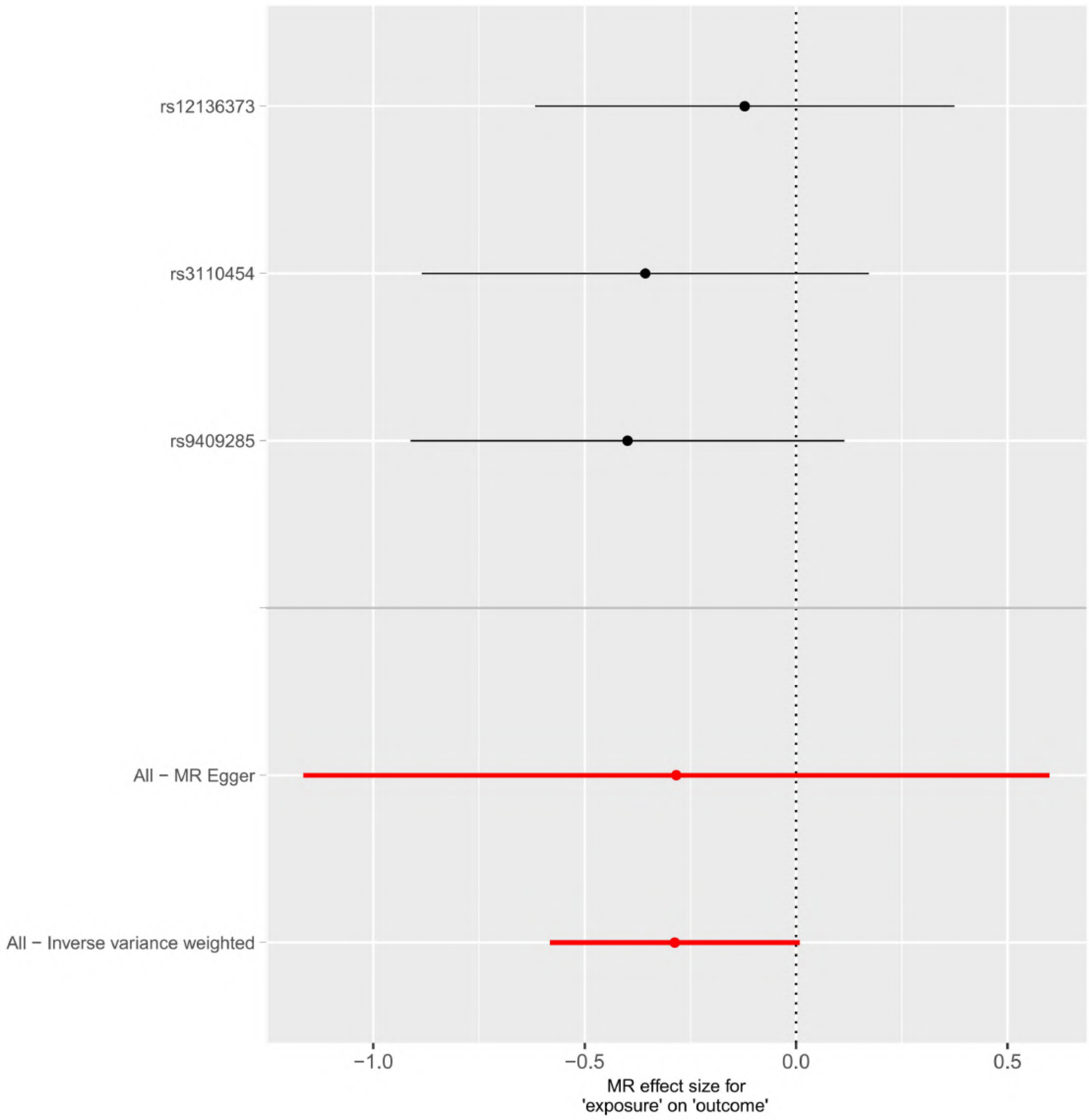

Supplementary Fig. 72 Forest plot of the causal effect of celery intake on GERD

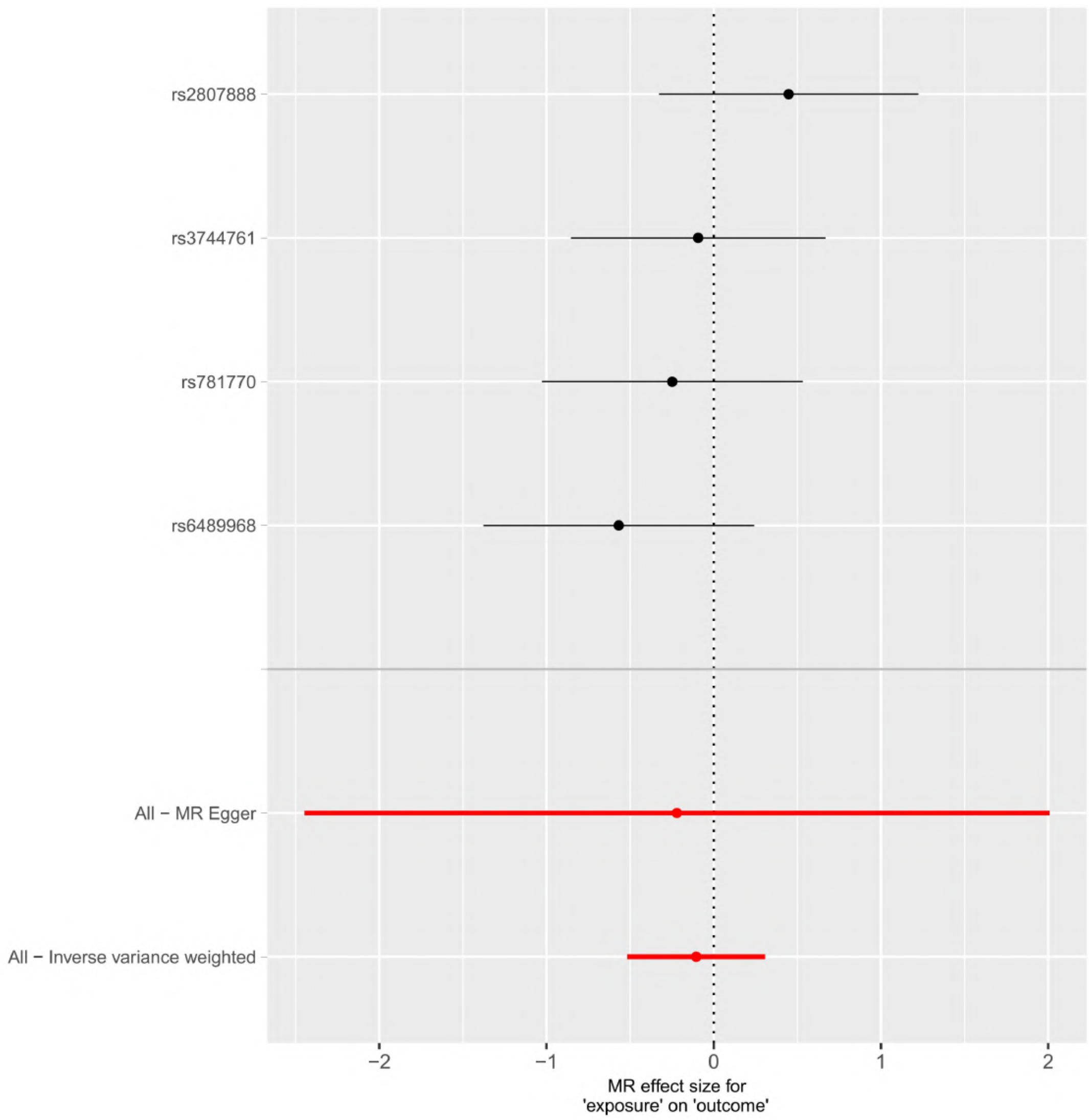

Supplementary Fig. 73 Forest plot of the causal effect of milk intake on GERD

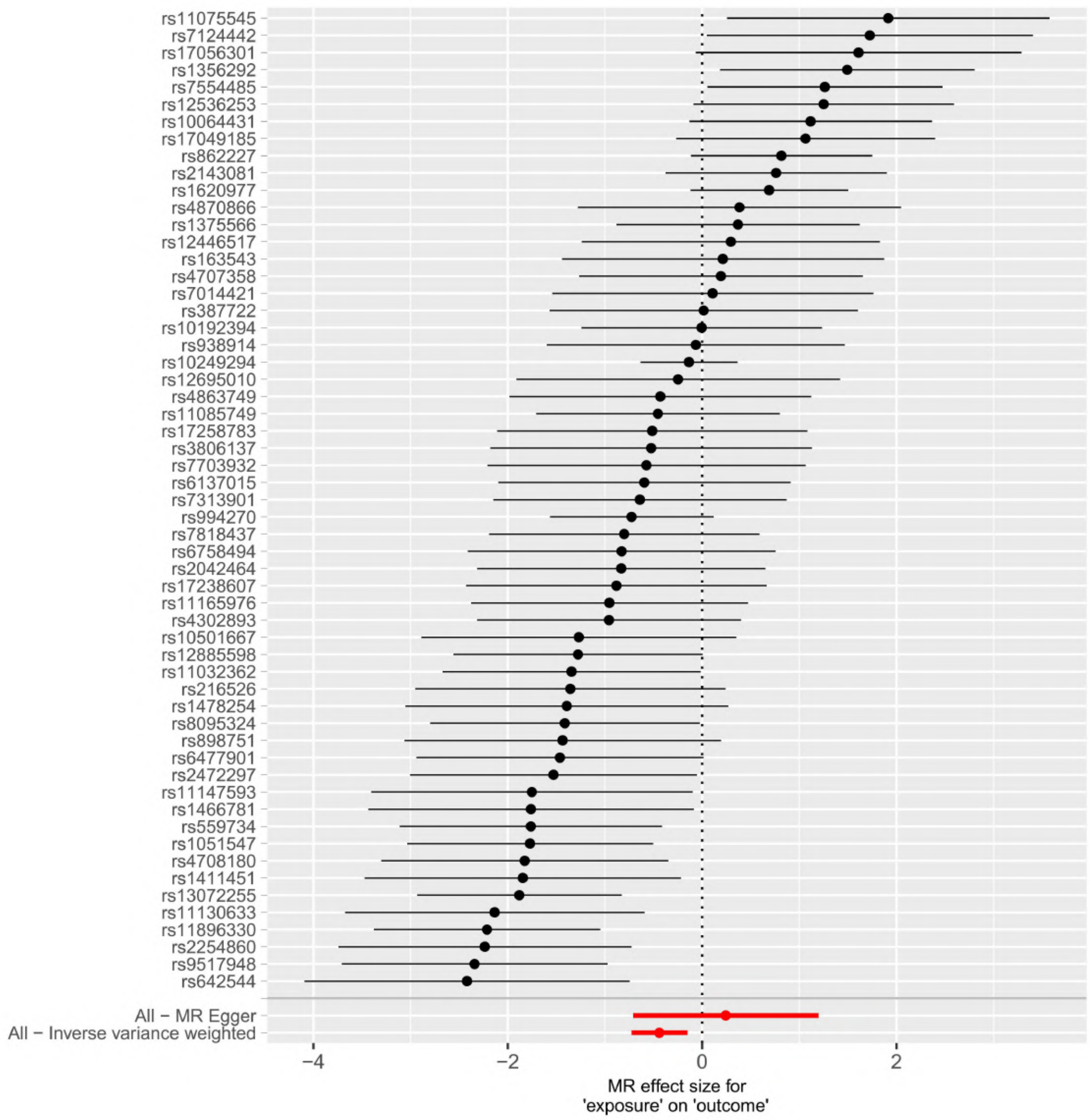

Supplementary Fig. 74 Forest plot of the causal effect of fresh fruit intake on GERD

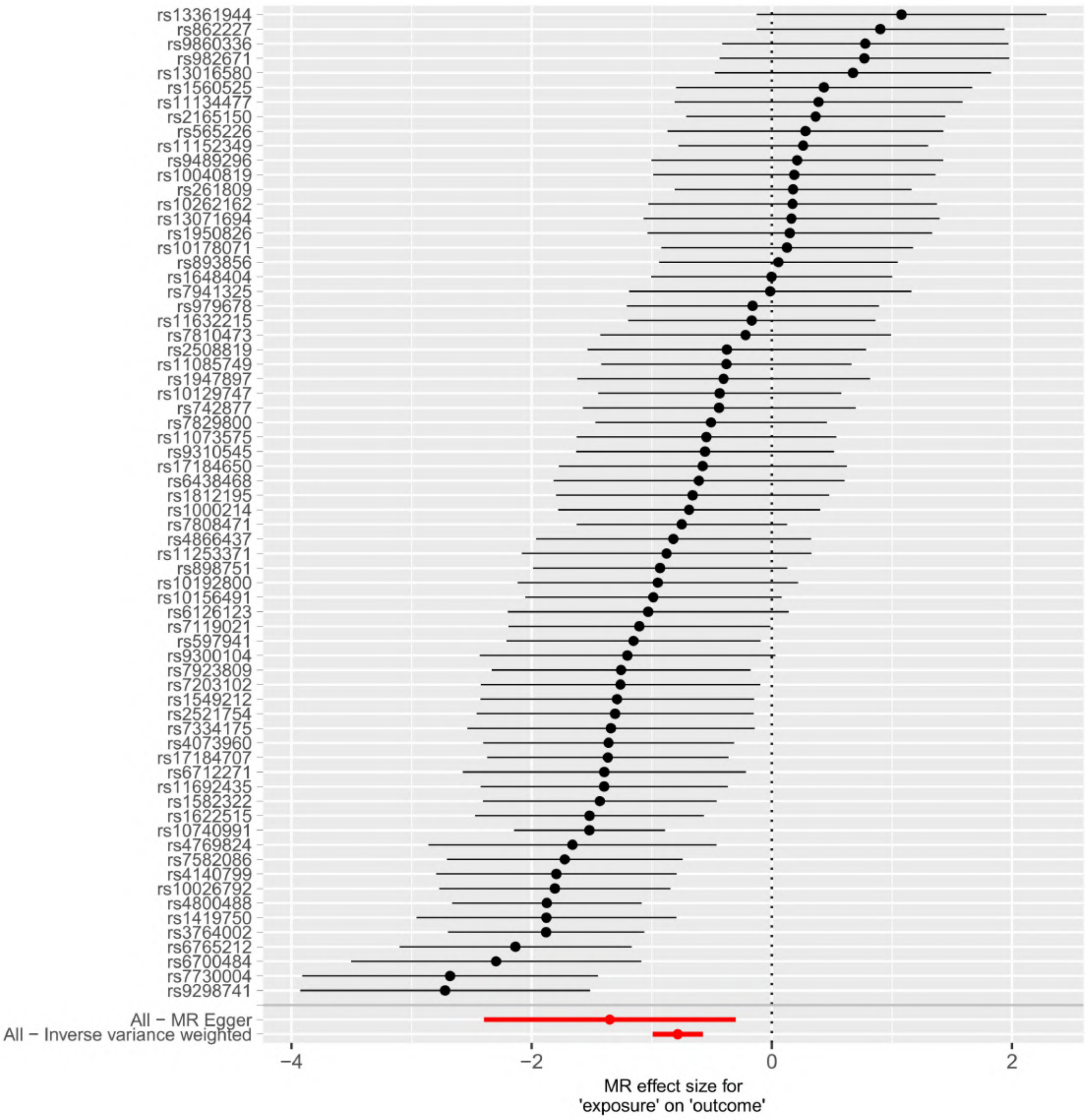

Supplementary Fig. 75 Forest plot of the causal effect of dried fruit intake on GERD

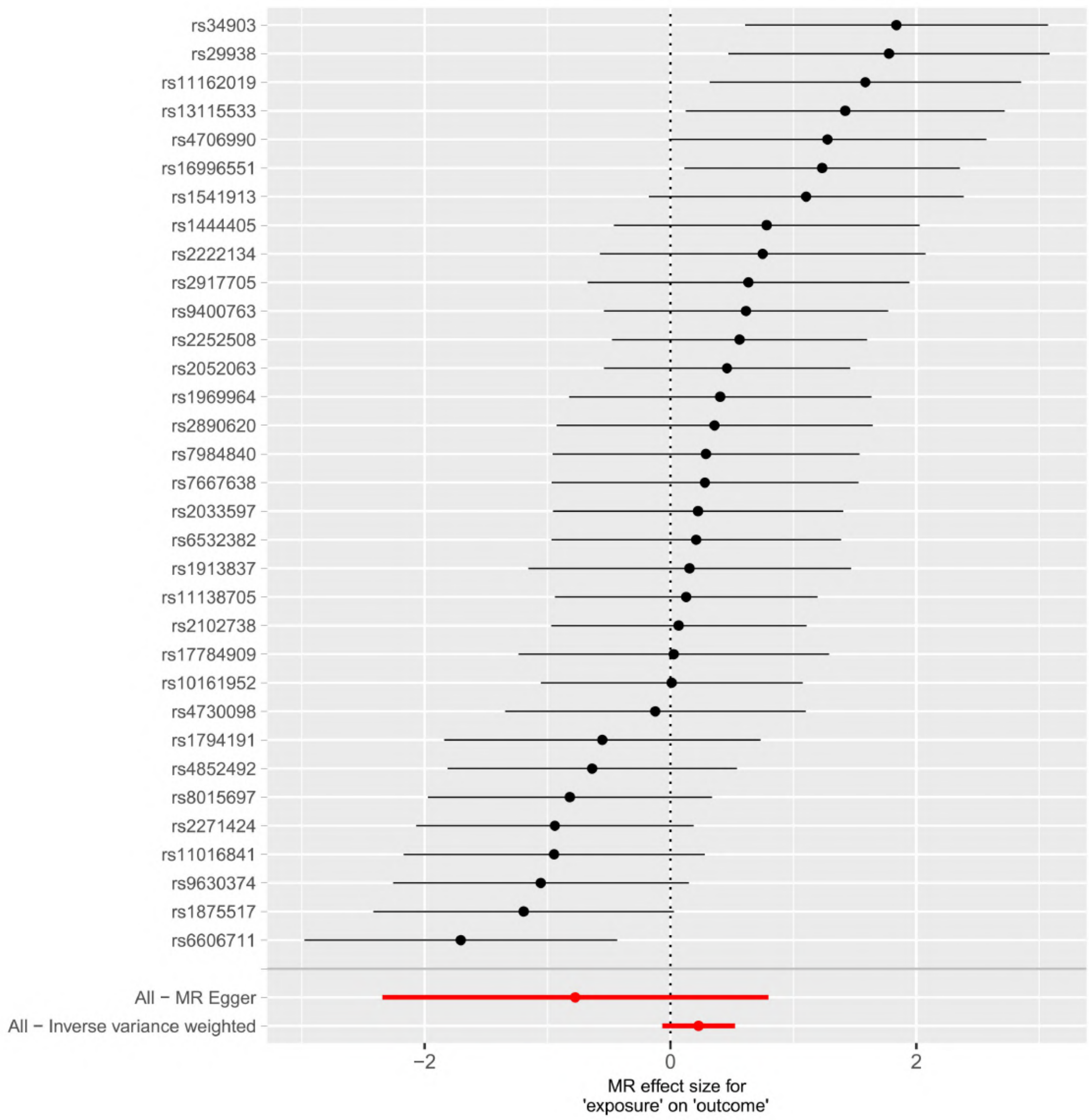

Supplementary Fig. 76 Forest plot of the causal effect of cooked vegetable intake on GERD

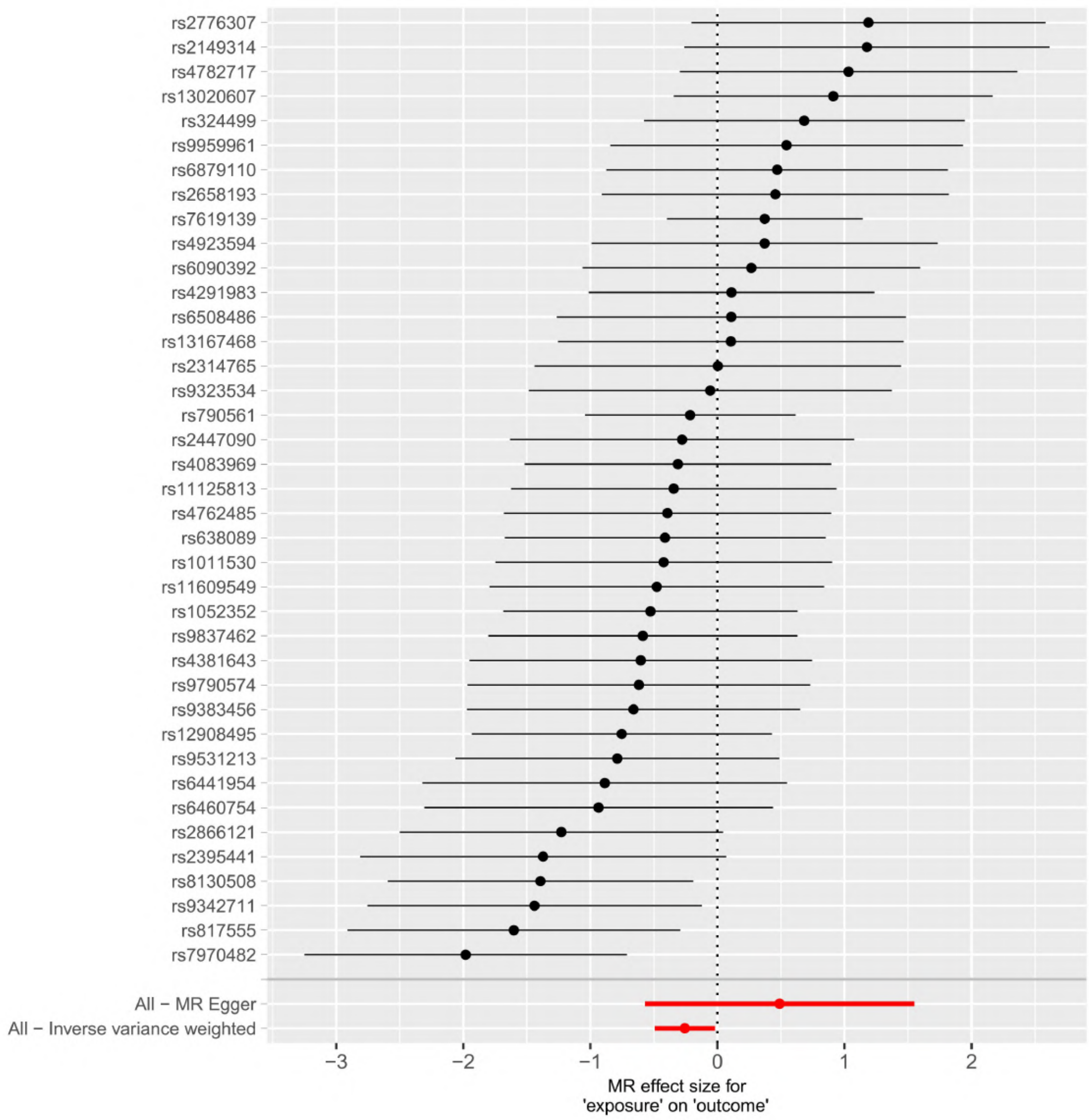

Supplementary Fig. 77 Forest plot of the causal effect of salad/raw vegetable intake on GERD

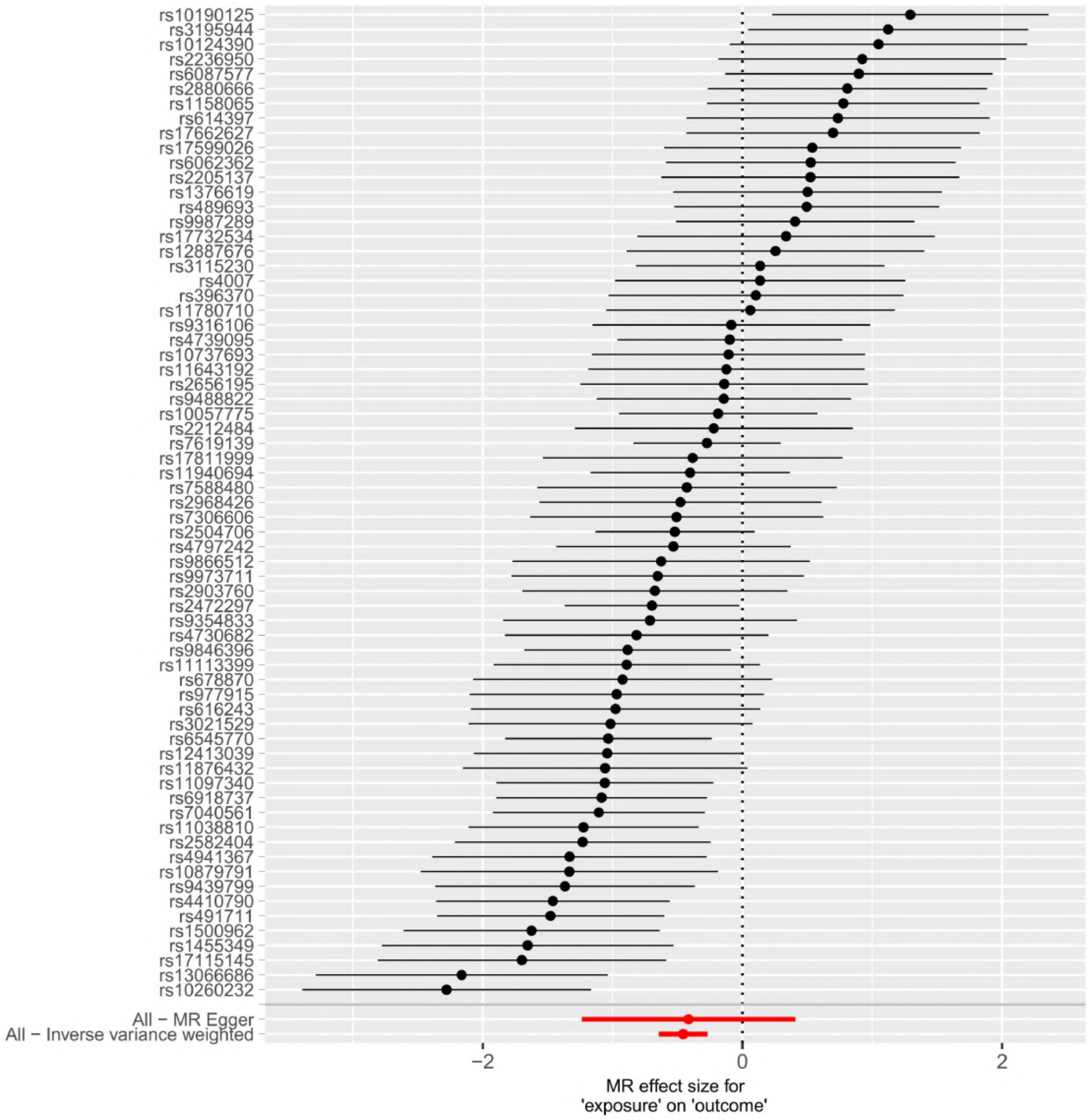

Supplementary Fig. 78 Forest plot of the causal effect of cereal intake on GERD

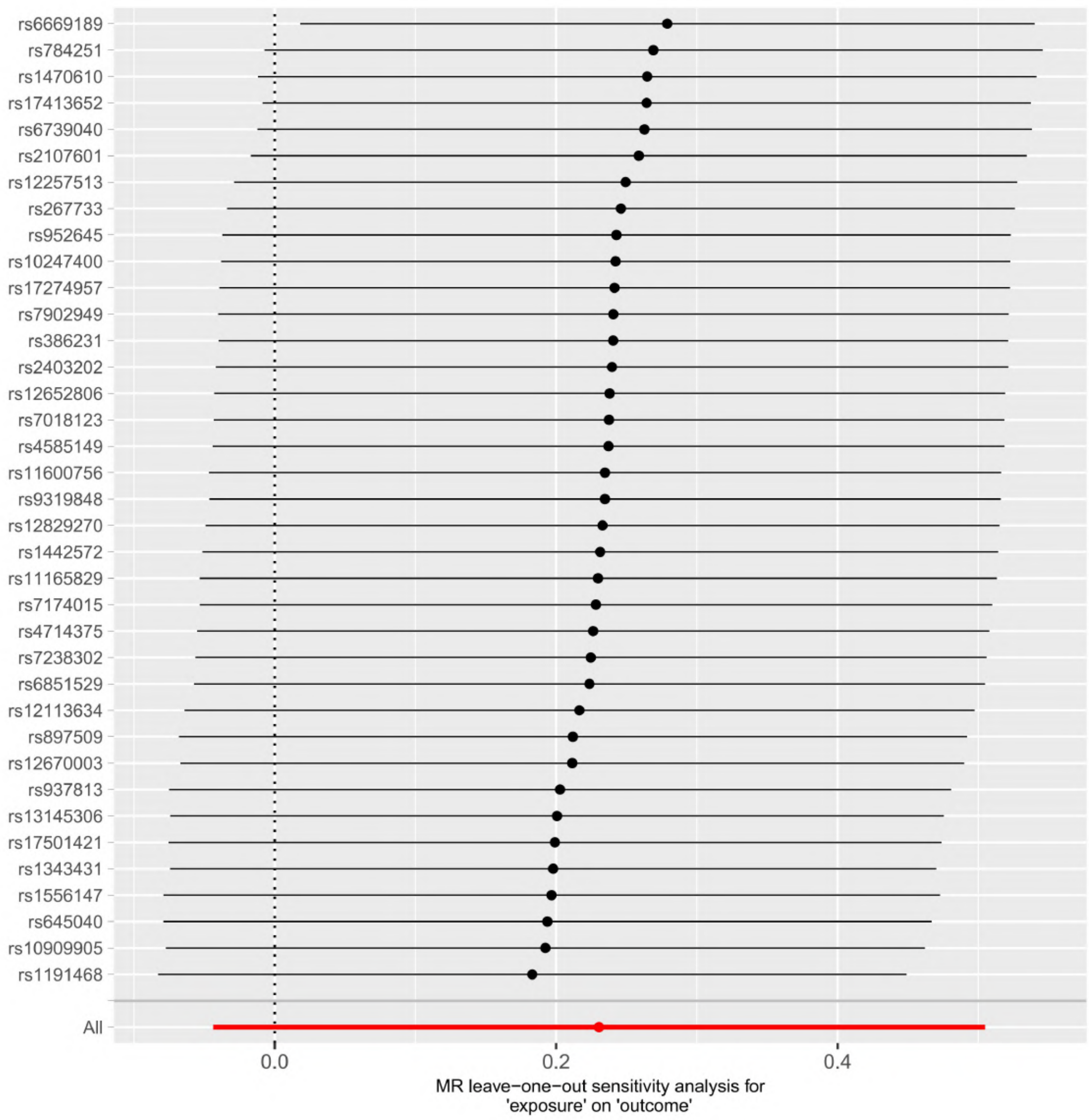

Supplementary Fig. 79 Leave-one-out inverse-variance weighted mendelian randomization analysis of beef intake on GERD

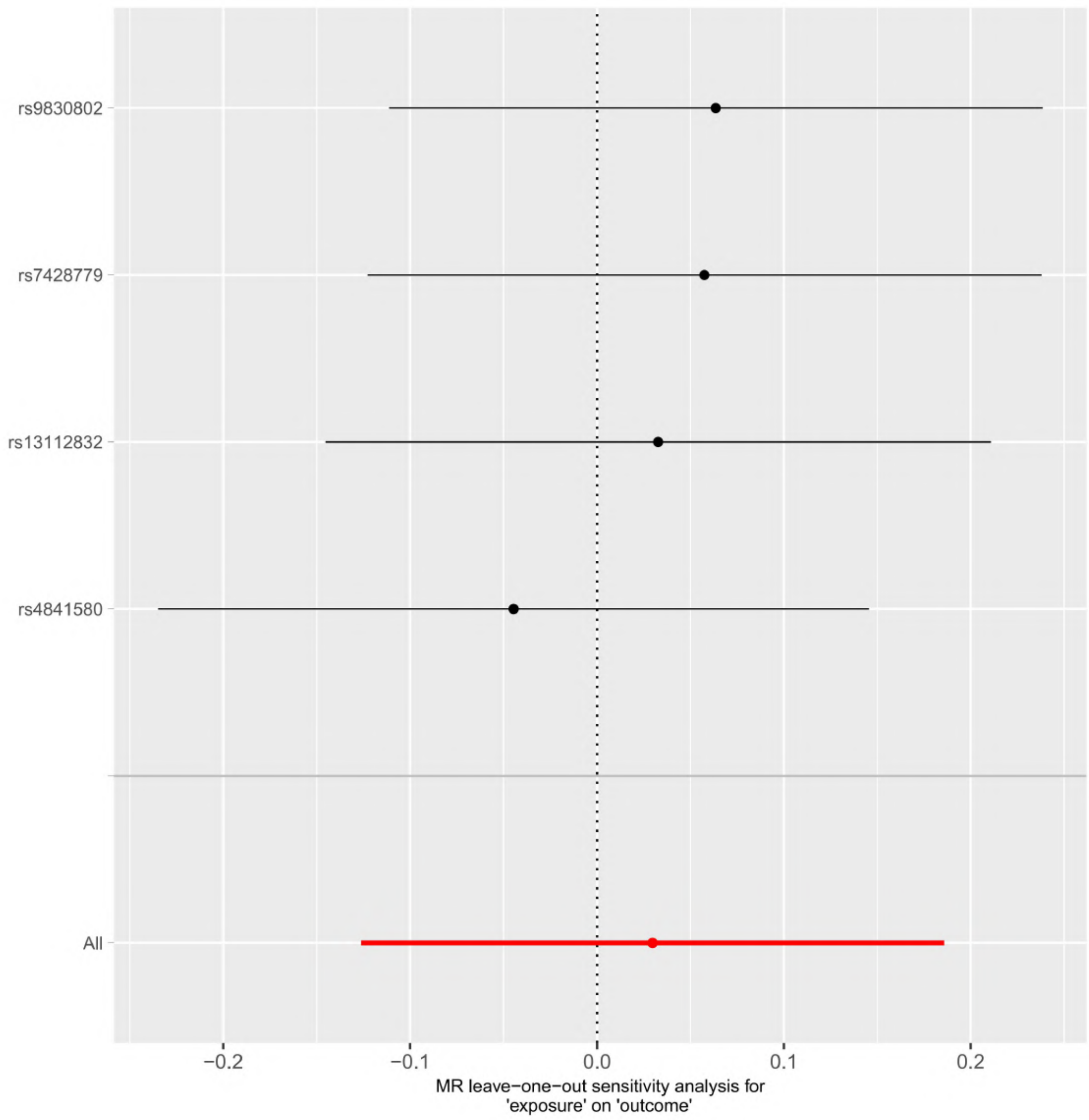

Supplementary Fig. 80 Leave-one-out inverse-variance weighted mendelian randomization analysis of banana intake on GERD

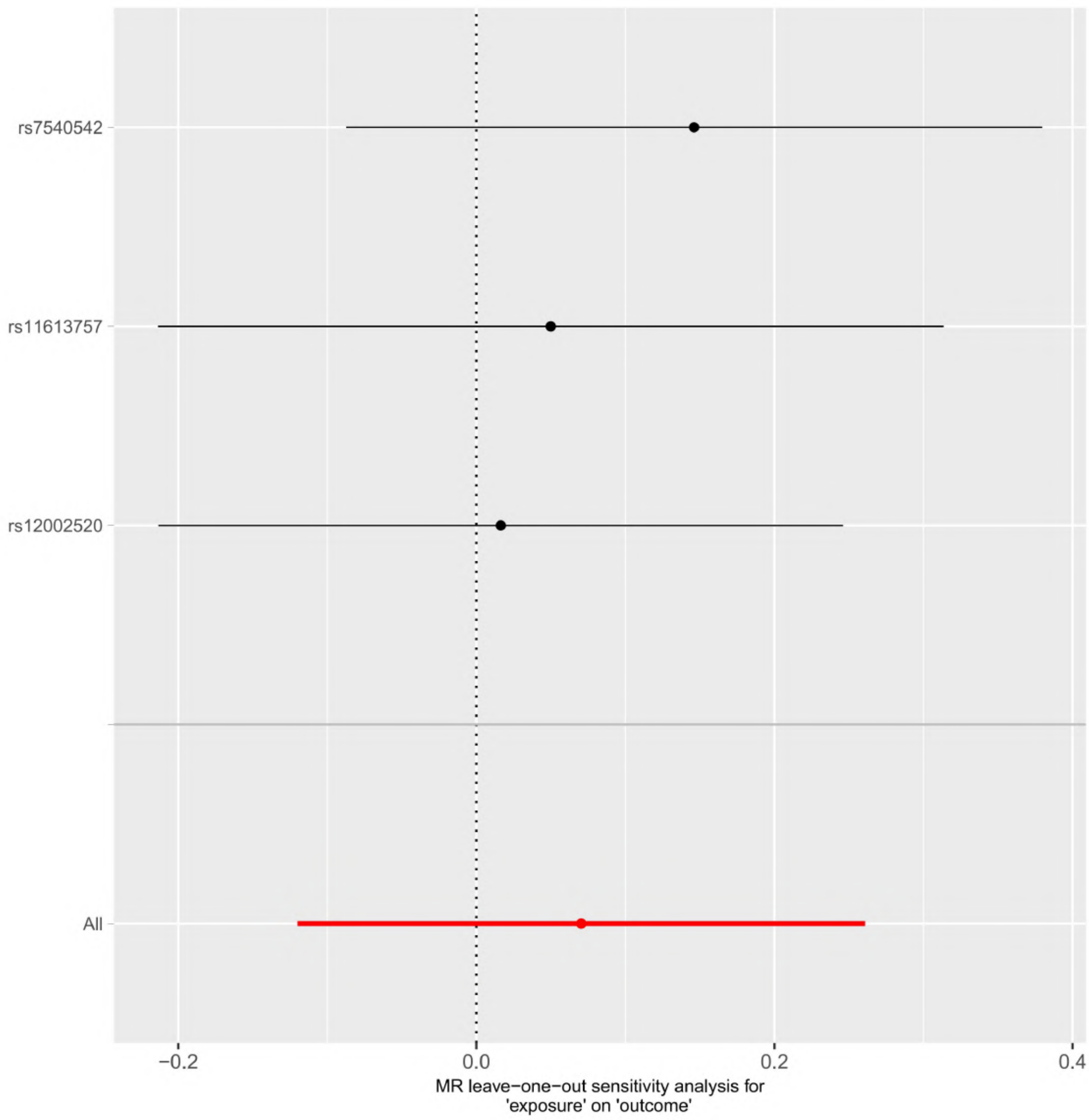

Supplementary Fig. 81 Leave-one-out inverse-variance weighted mendelian randomization analysis of broccoli intake on GERD

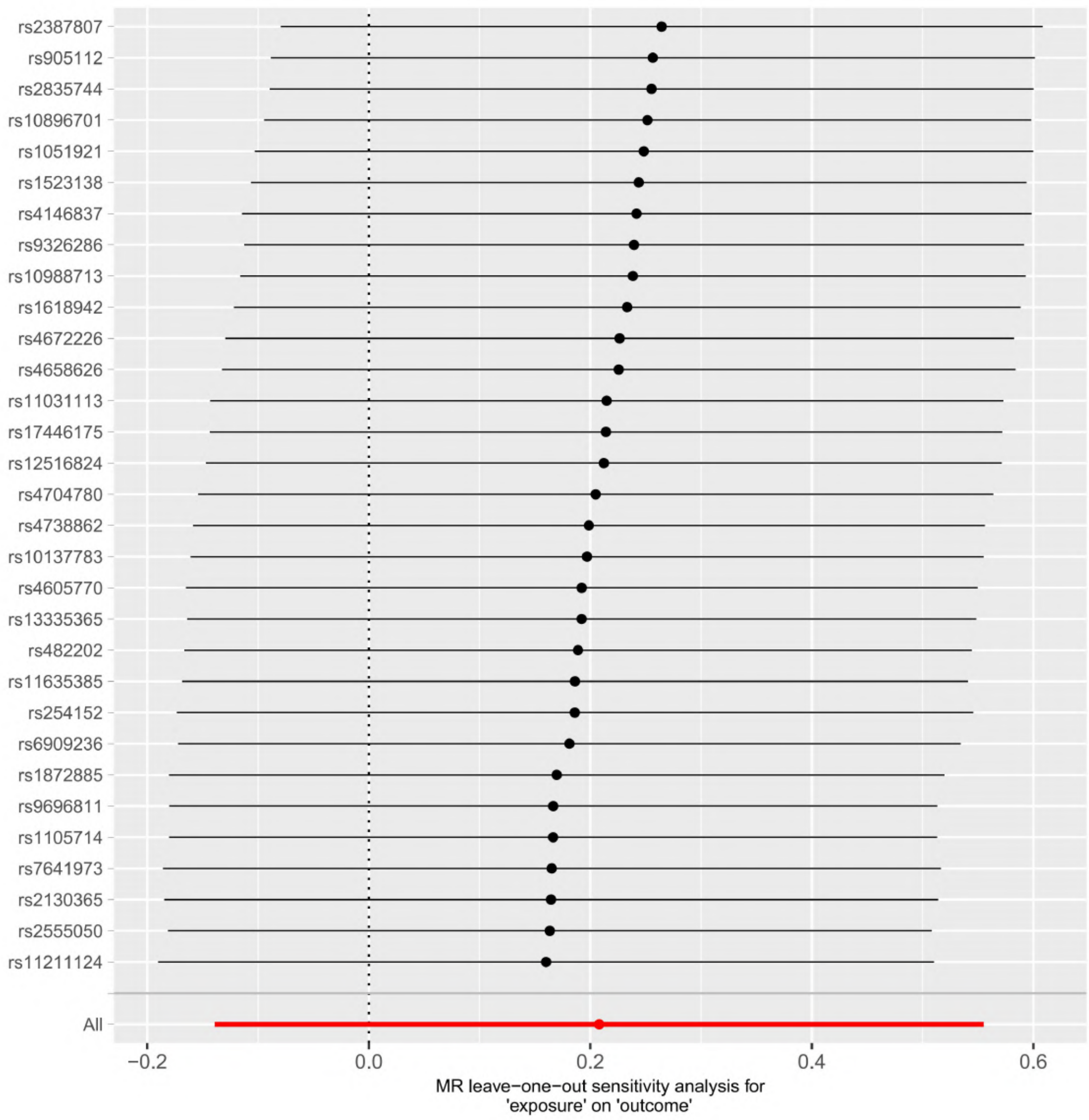

Supplementary Fig. 82 Leave-one-out inverse-variance weighted mendelian randomization analysis of pork intake on GERD

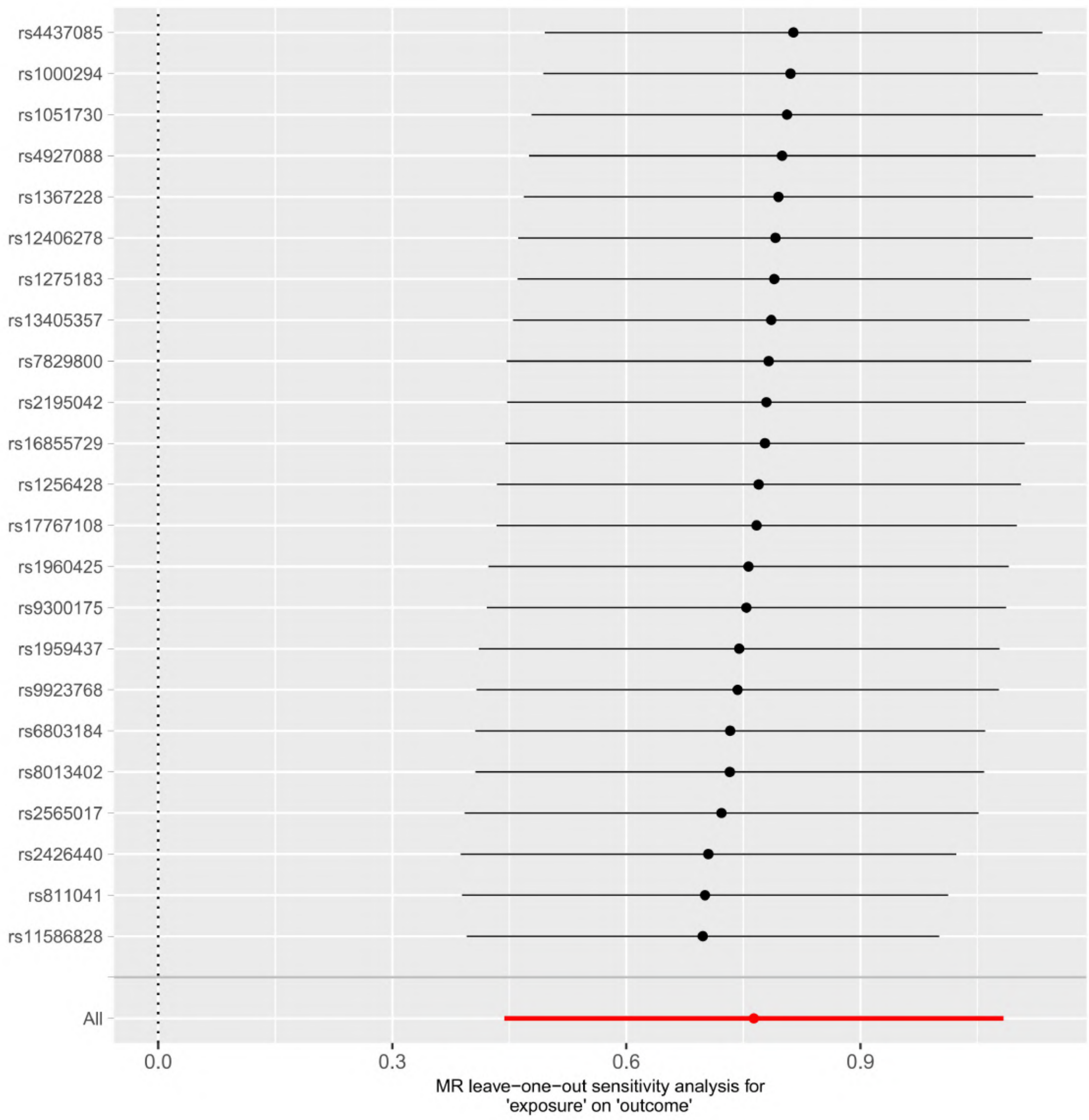

Supplementary Fig. 83 Leave-one-out inverse-variance weighted mendelian randomization analysis of pourtry intake on GERD

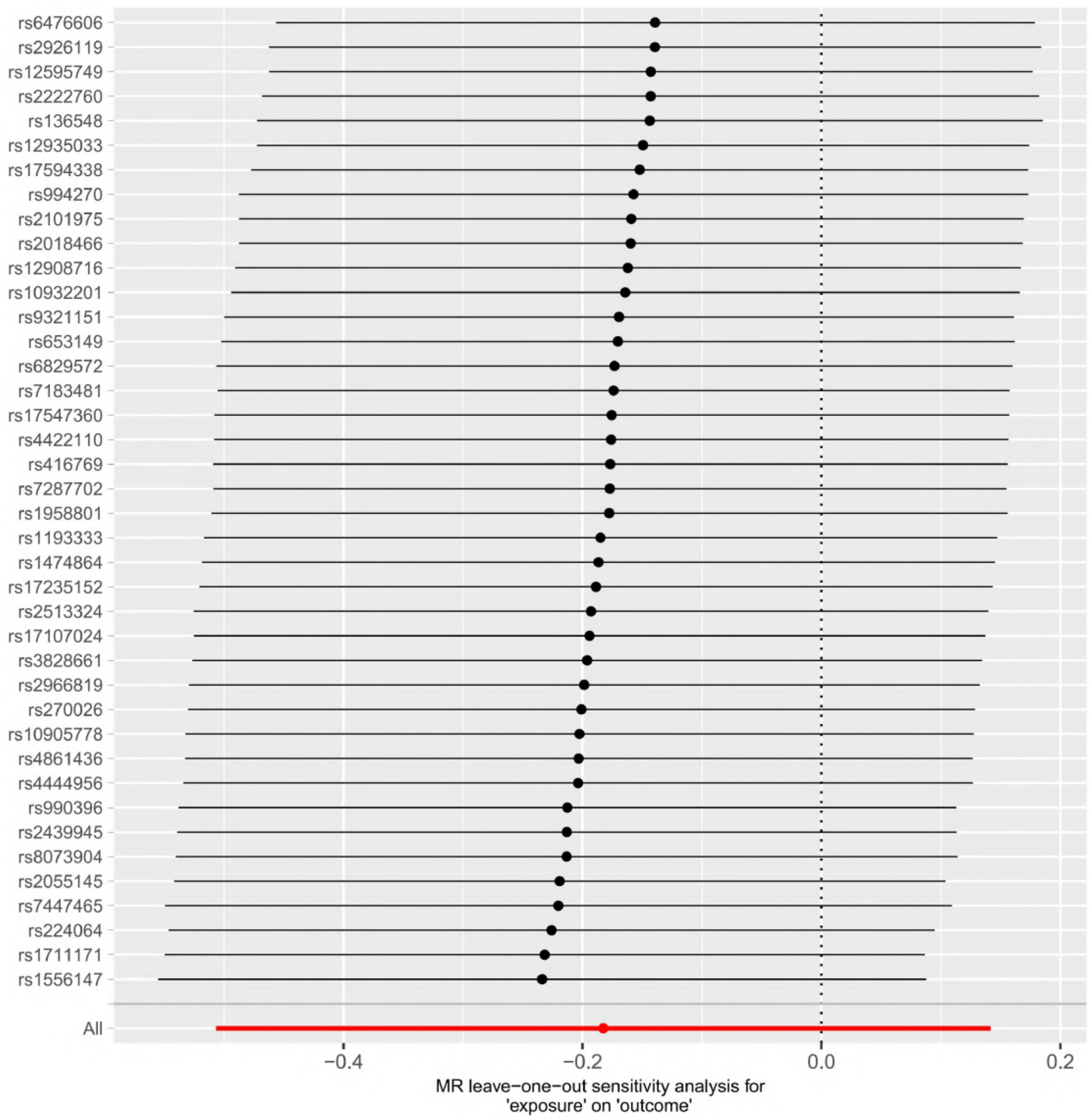

Supplementary Fig. 84 Leave-one-out inverse-variance weighted mendelian randomization analysis of Lamb/mutton intake on GERD

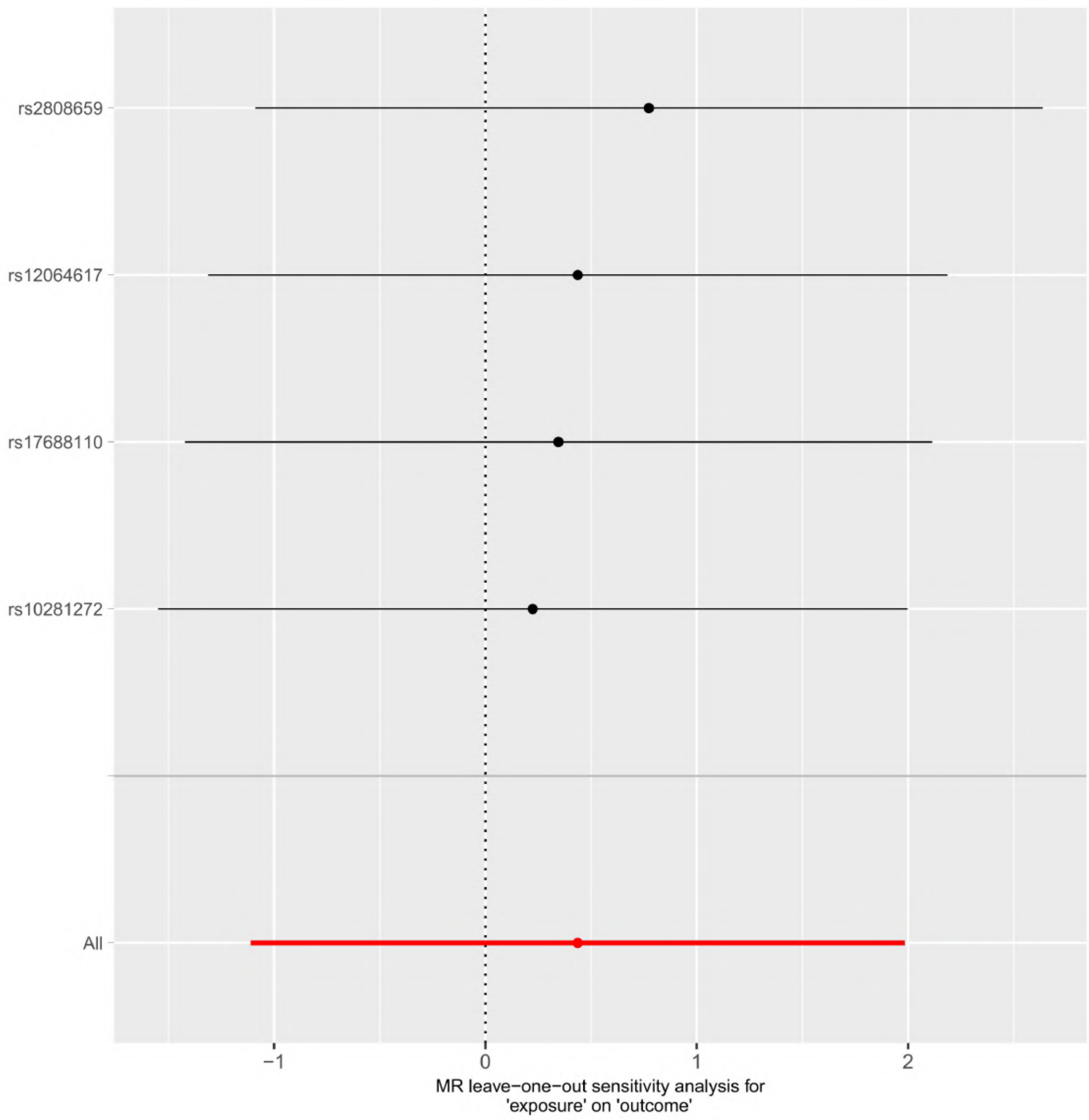

Supplementary Fig. 85 Leave-one-out inverse-variance weighted mendelian randomization analysis of Lobster/crab intake on GERD

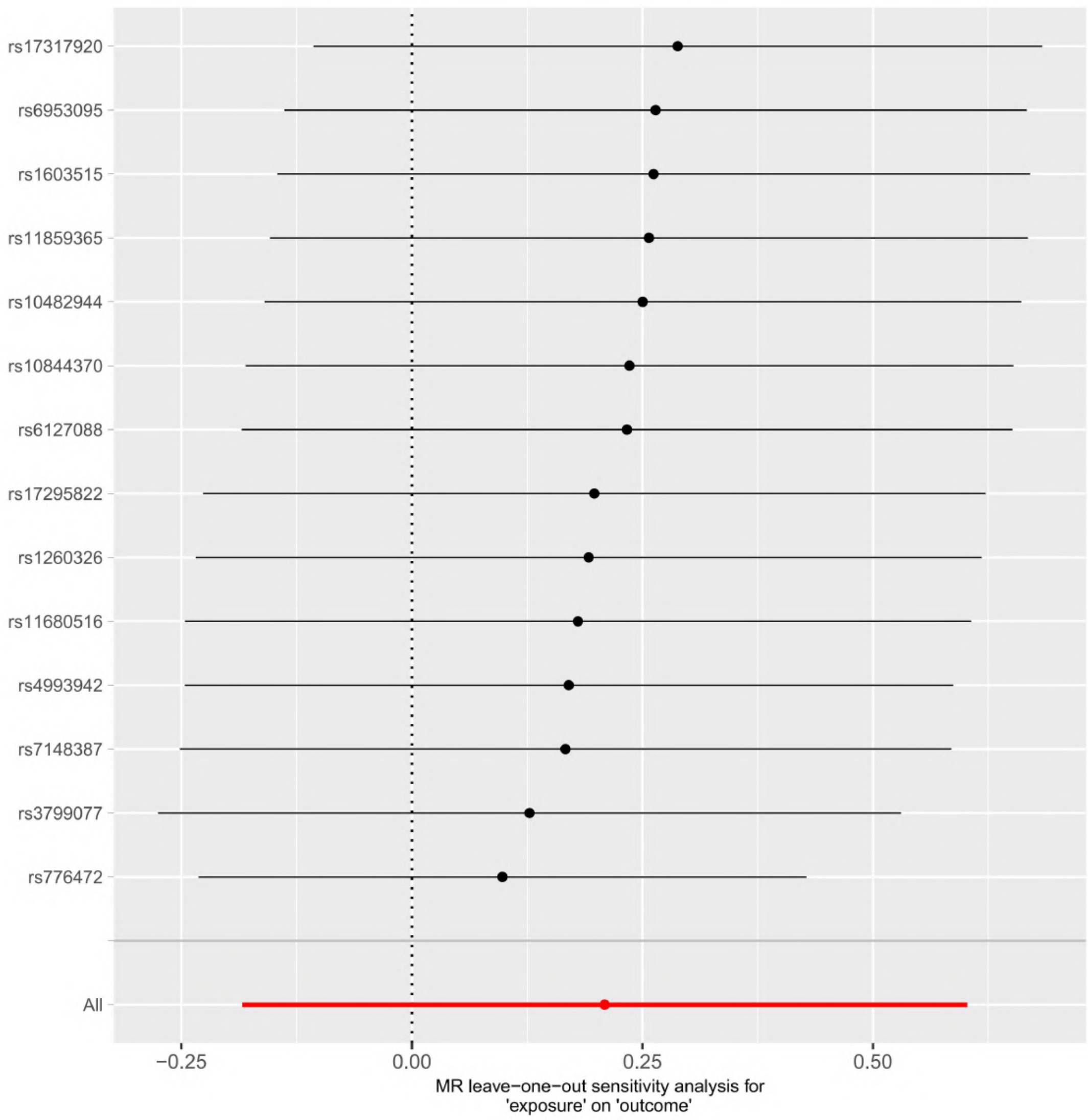

Supplementary Fig. 86 Leave-one-out inverse-variance weighted mendelian randomization analysis of Non-oily fish intake on GERD

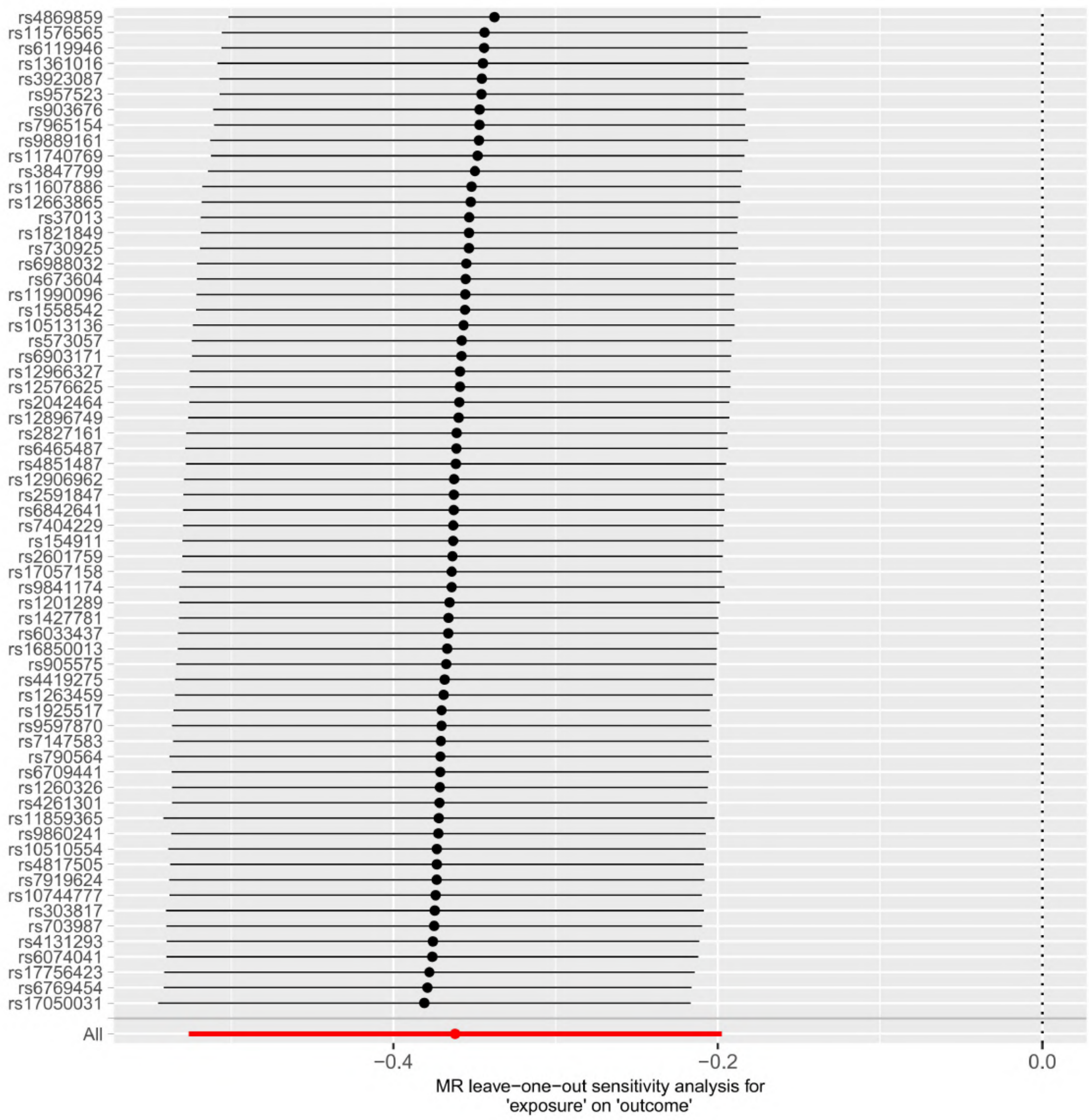

Supplementary Fig. 87 Leave-one-out inverse-variance weighted mendelian randomization analysis of oily fish intake on GERD

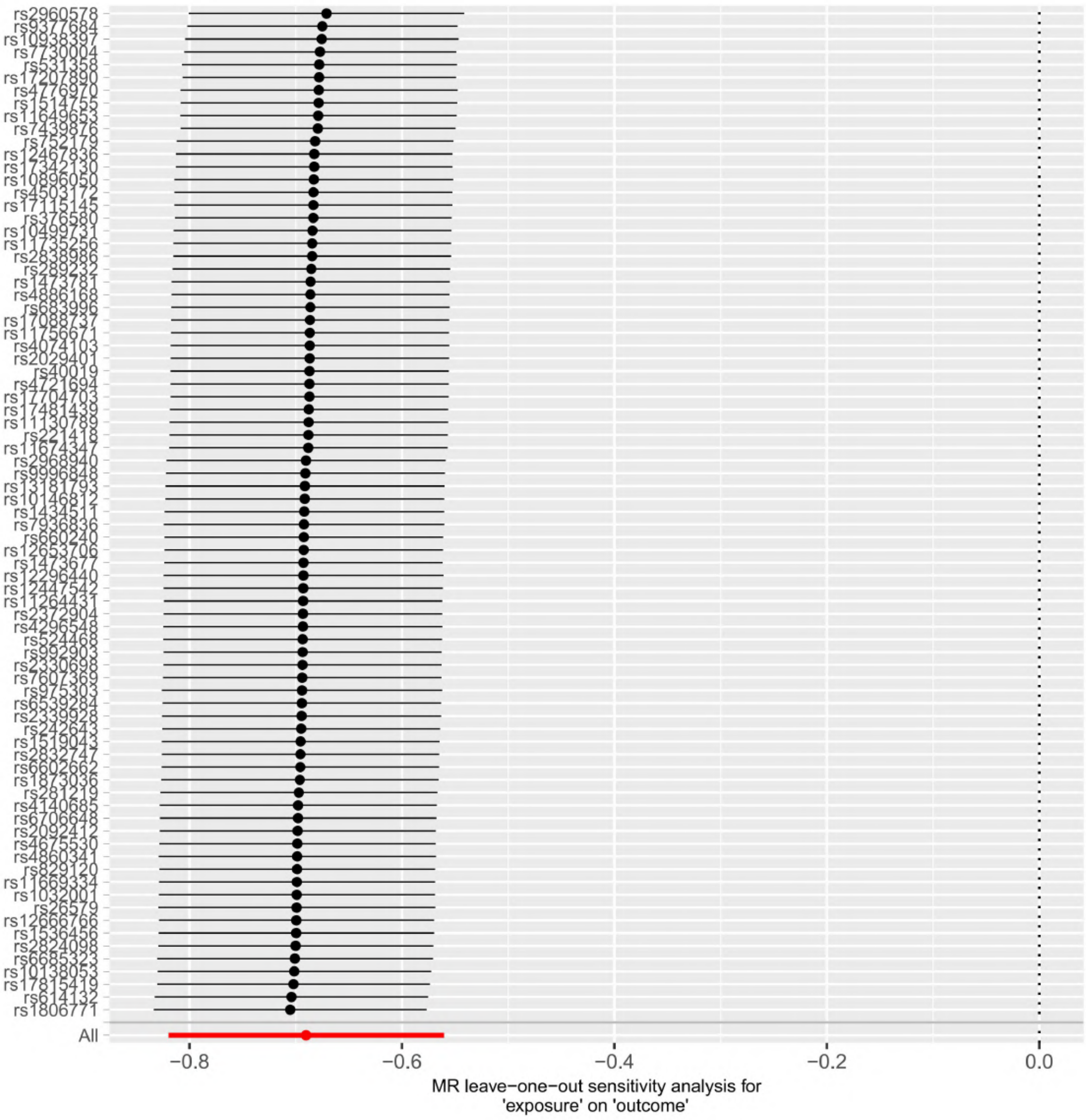

Supplementary Fig. 88 Leave-one-out inverse-variance weighted mendelian randomization analysis of cheese intake on GERD

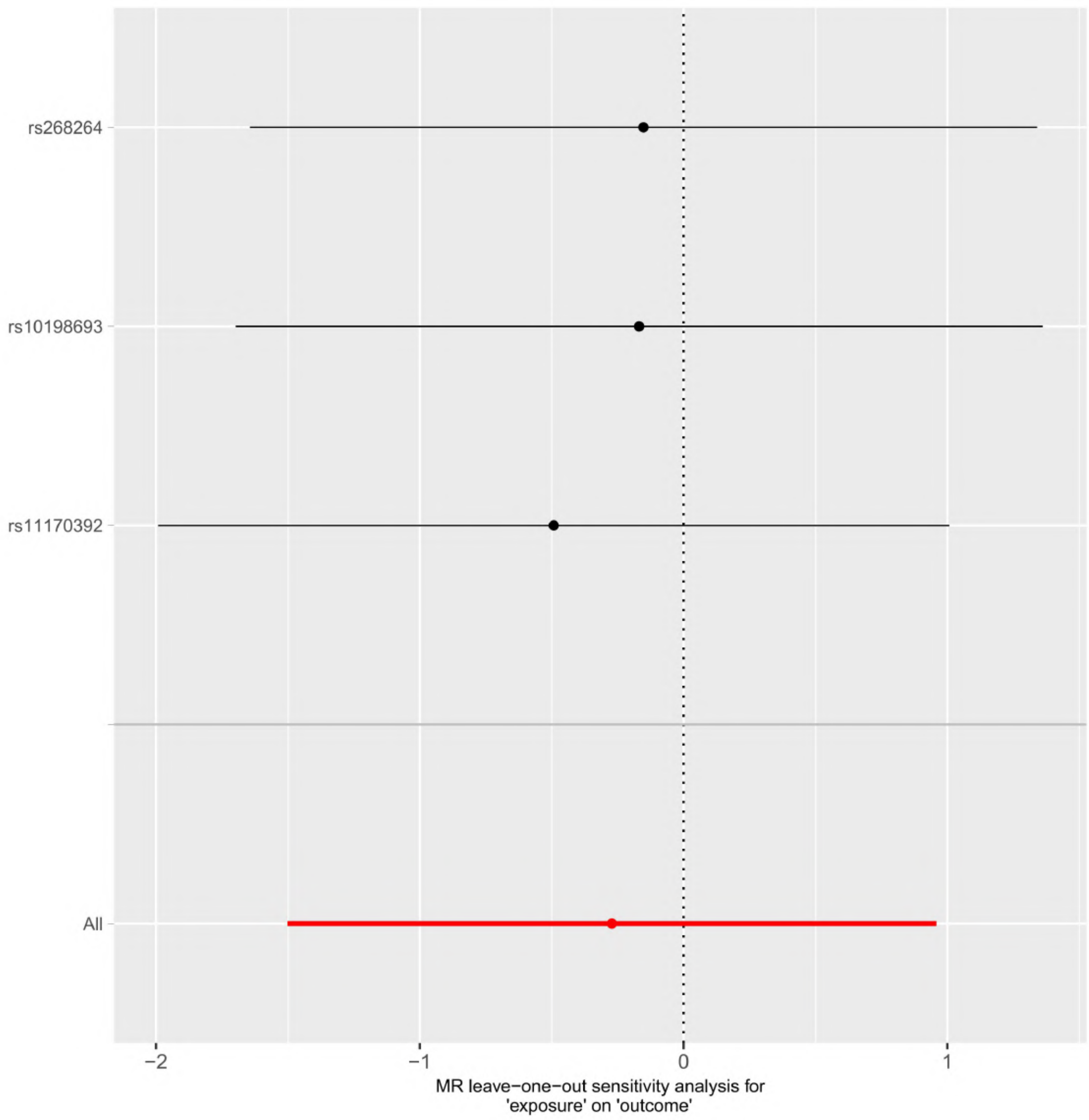

Supplementary Fig. 89 Leave-one-out inverse-variance weighted mendelian randomization analysis of feta intake on GERD

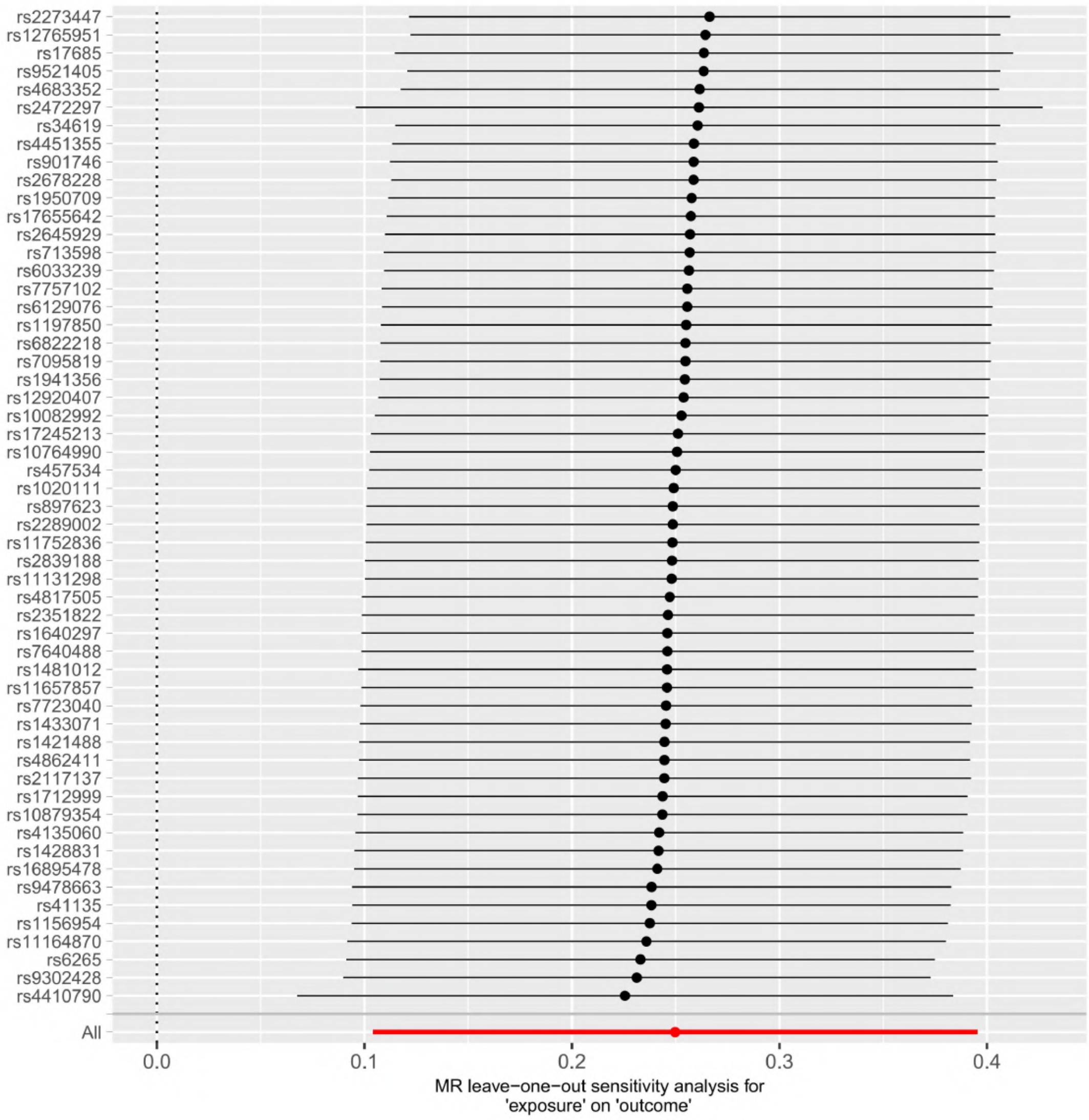

Supplementary Fig. 90 Leave-one-out inverse-variance weighted mendelian randomization analysis of tea intake on GERD

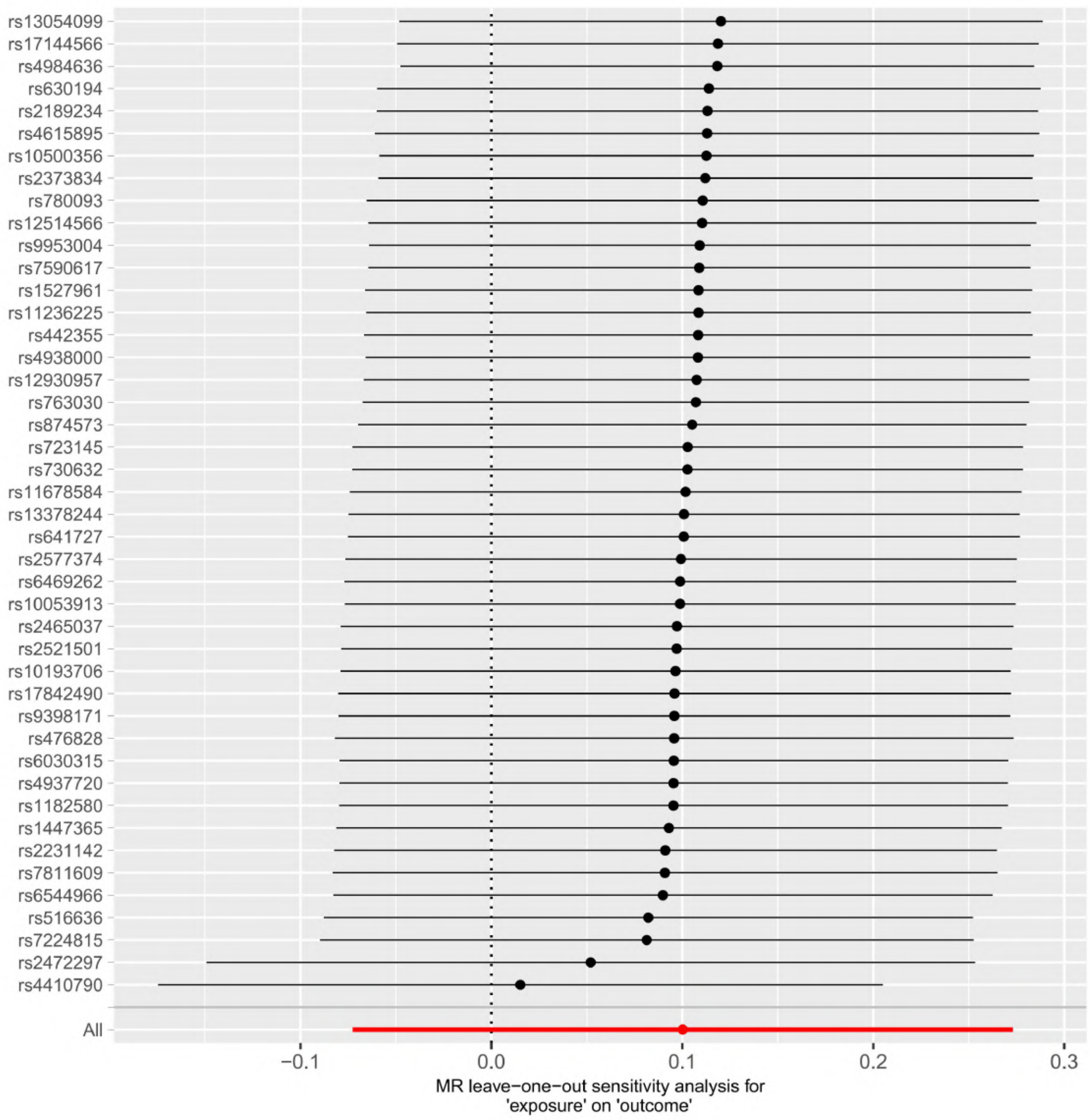

Supplementary Fig. 91 Leave-one-out inverse-variance weighted mendelian randomization analysis of coffee intake on GERD

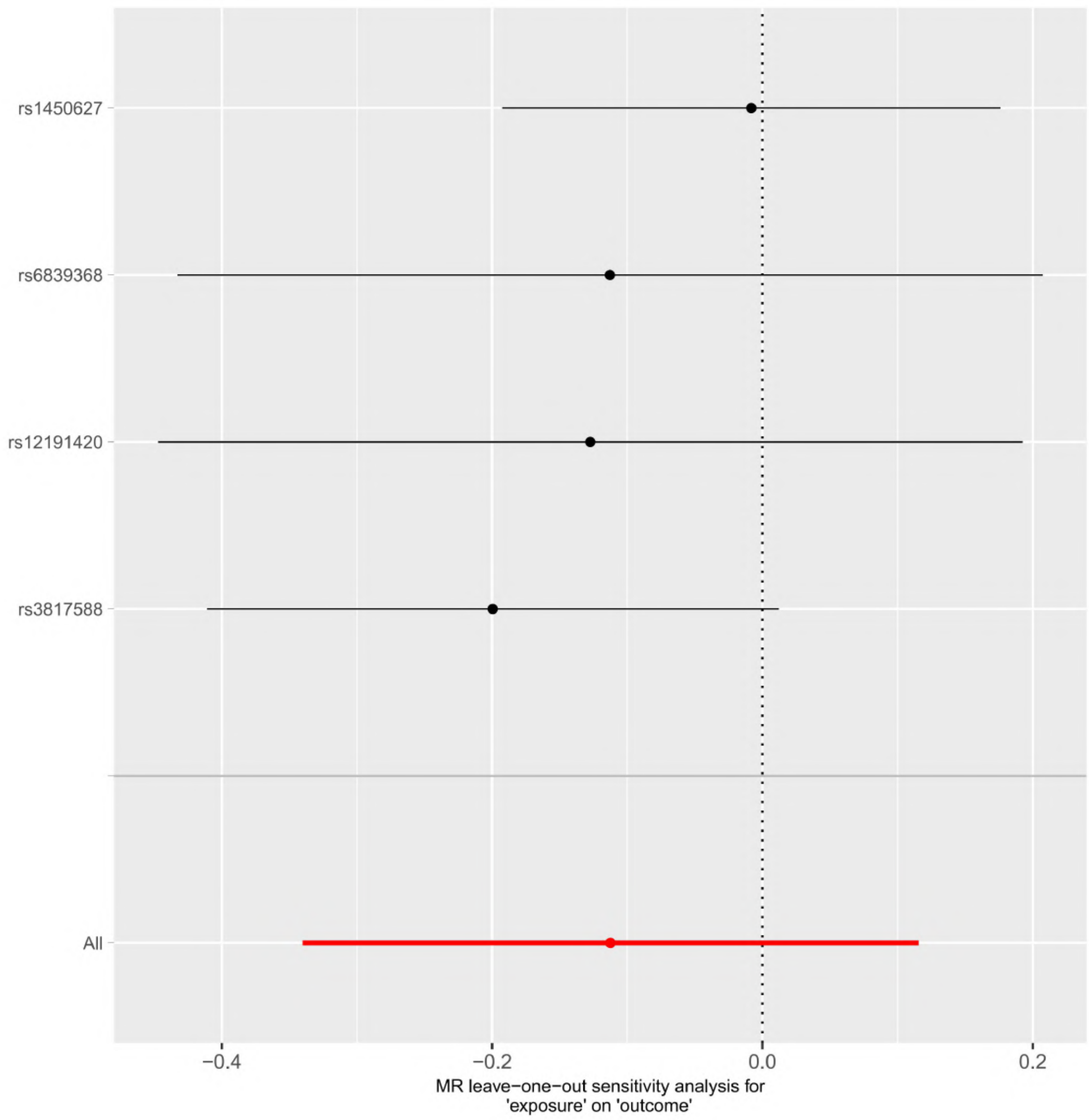

Supplementary Fig. 92 Leave-one-out inverse-variance weighted mendelian randomization analysis of beer/cider intake on GERD

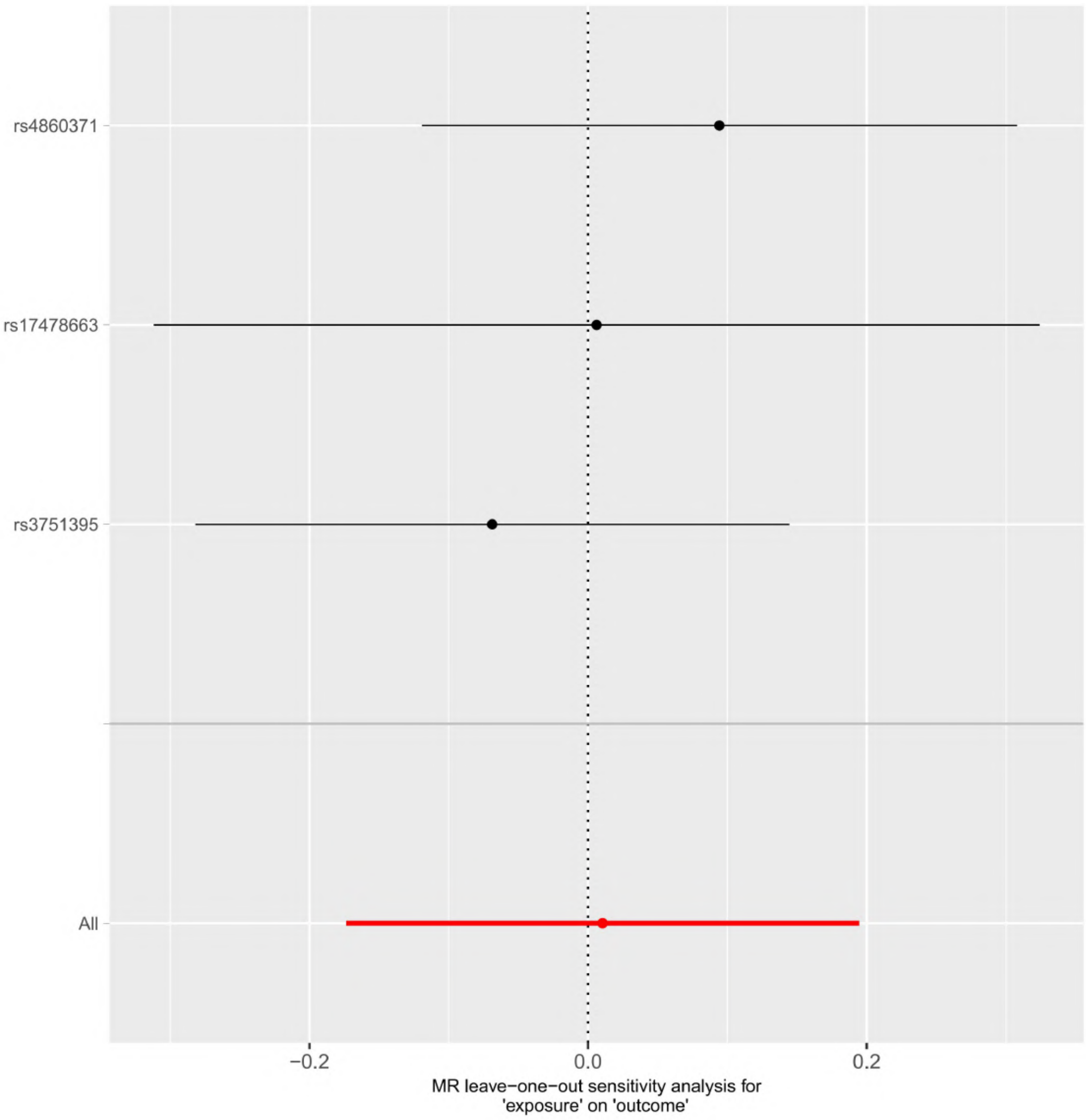

Supplementary Fig. 93 Leave-one-out inverse-variance weighted mendelian randomization analysis of red wine intake on GERD

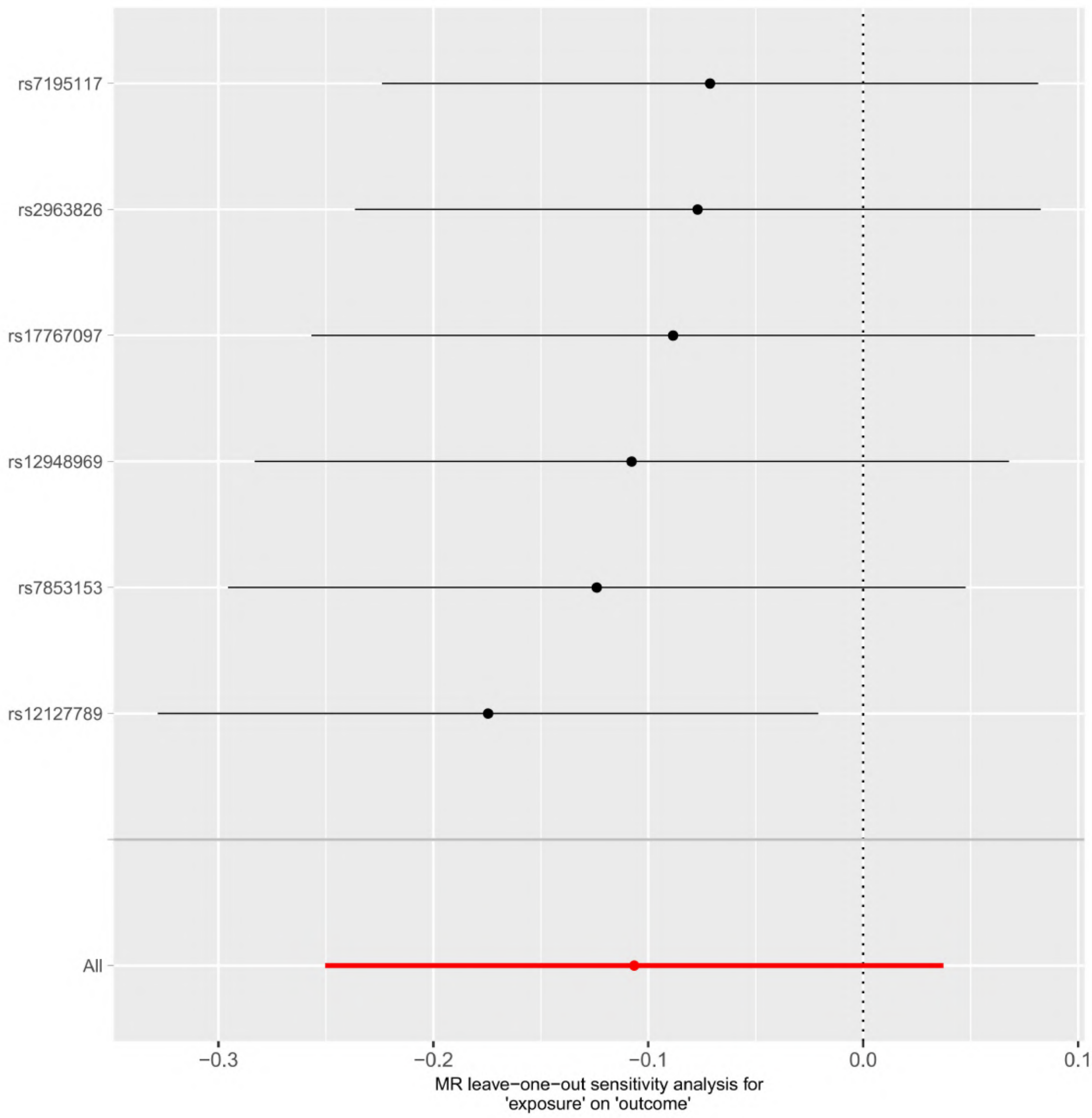

Supplementary Fig. 94 Leave-one-out inverse-variance weighted mendelian randomization analysis of apple intake on GERD

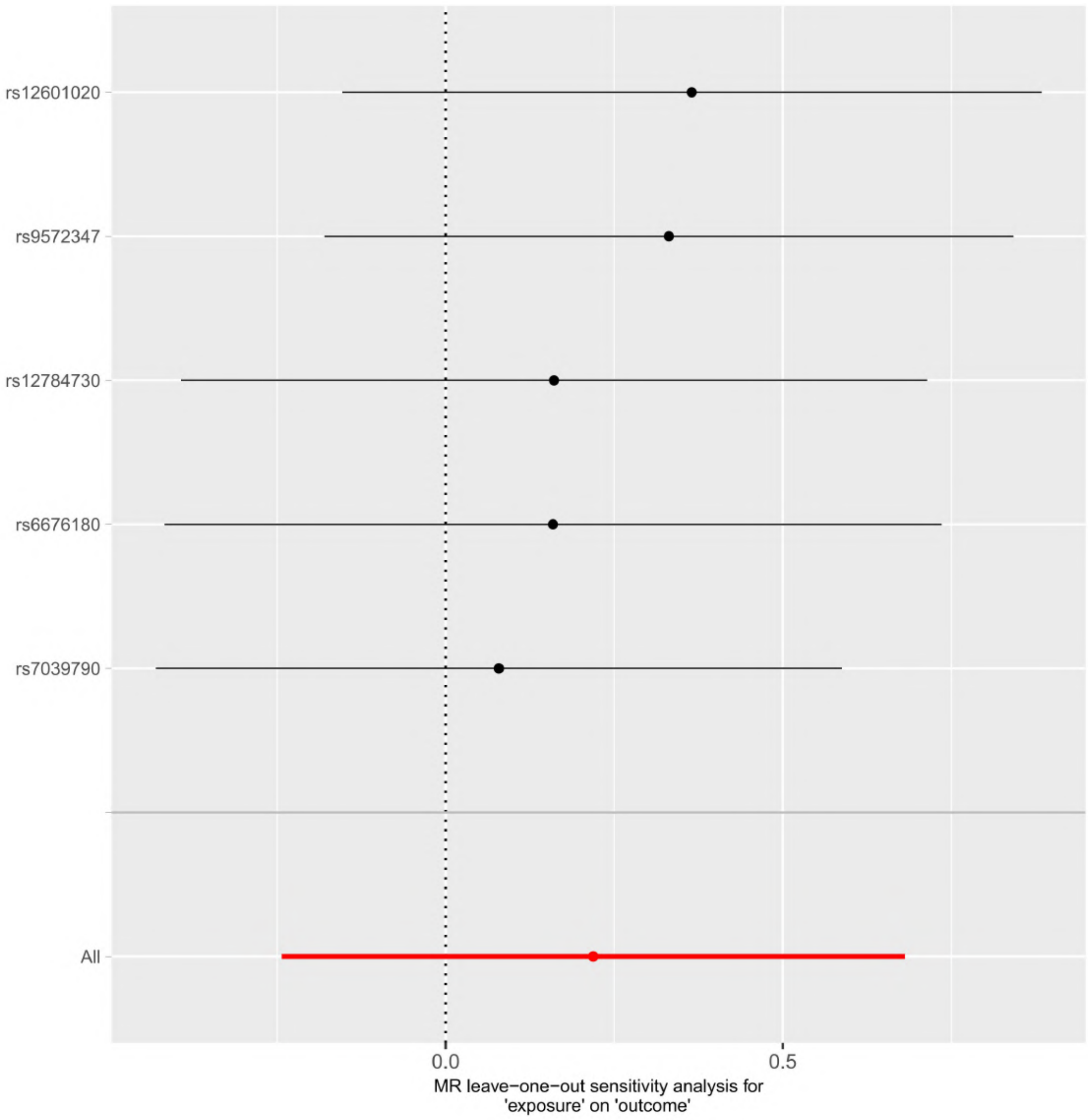

Supplementary Fig. 95 Leave-one-out inverse-variance weighted mendelian randomization analysis of cheery intake on GERD

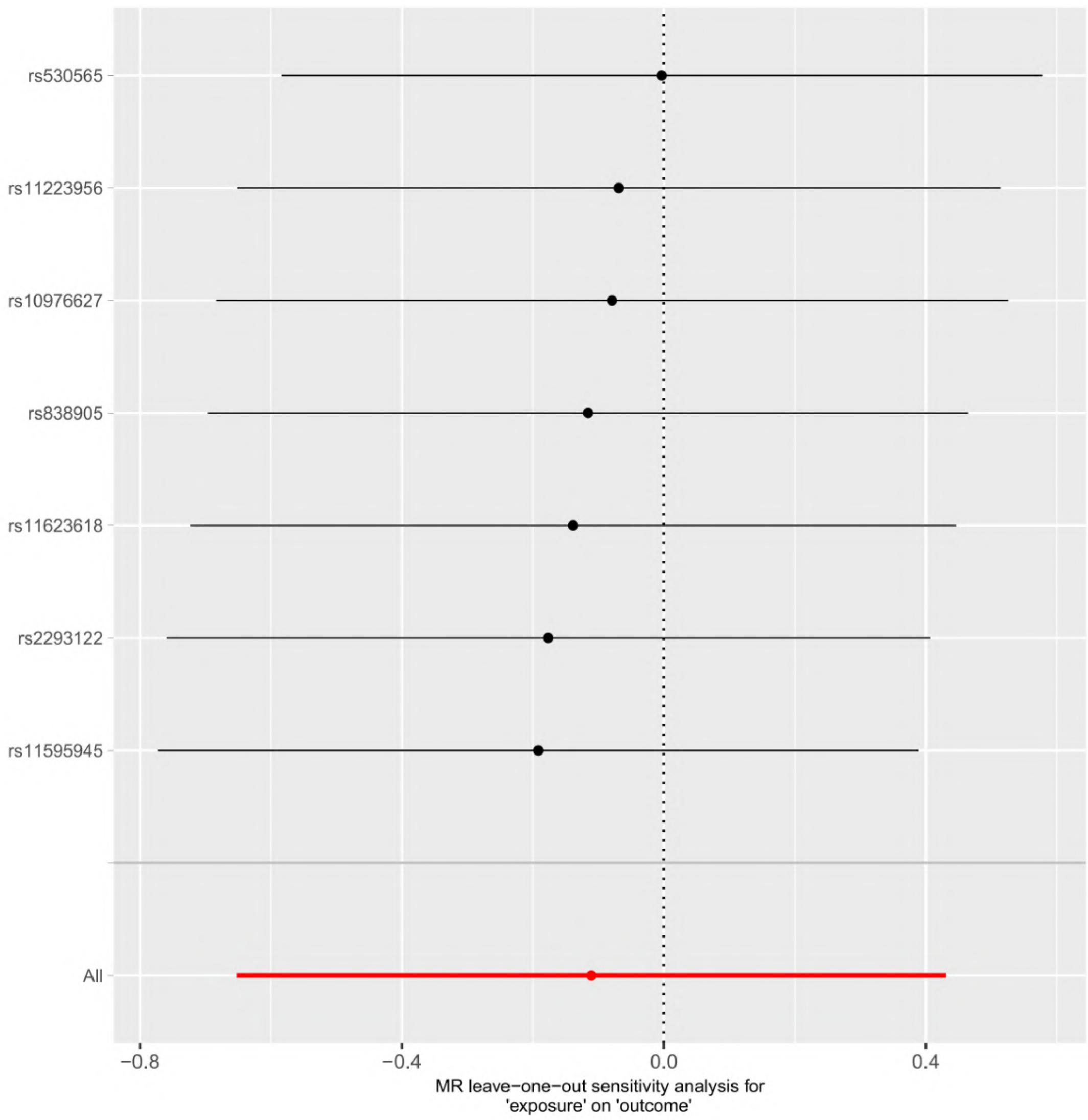

Supplementary Fig. 96 Leave-one-out inverse-variance weighted mendelian randomization analysis of mango intake on GERD

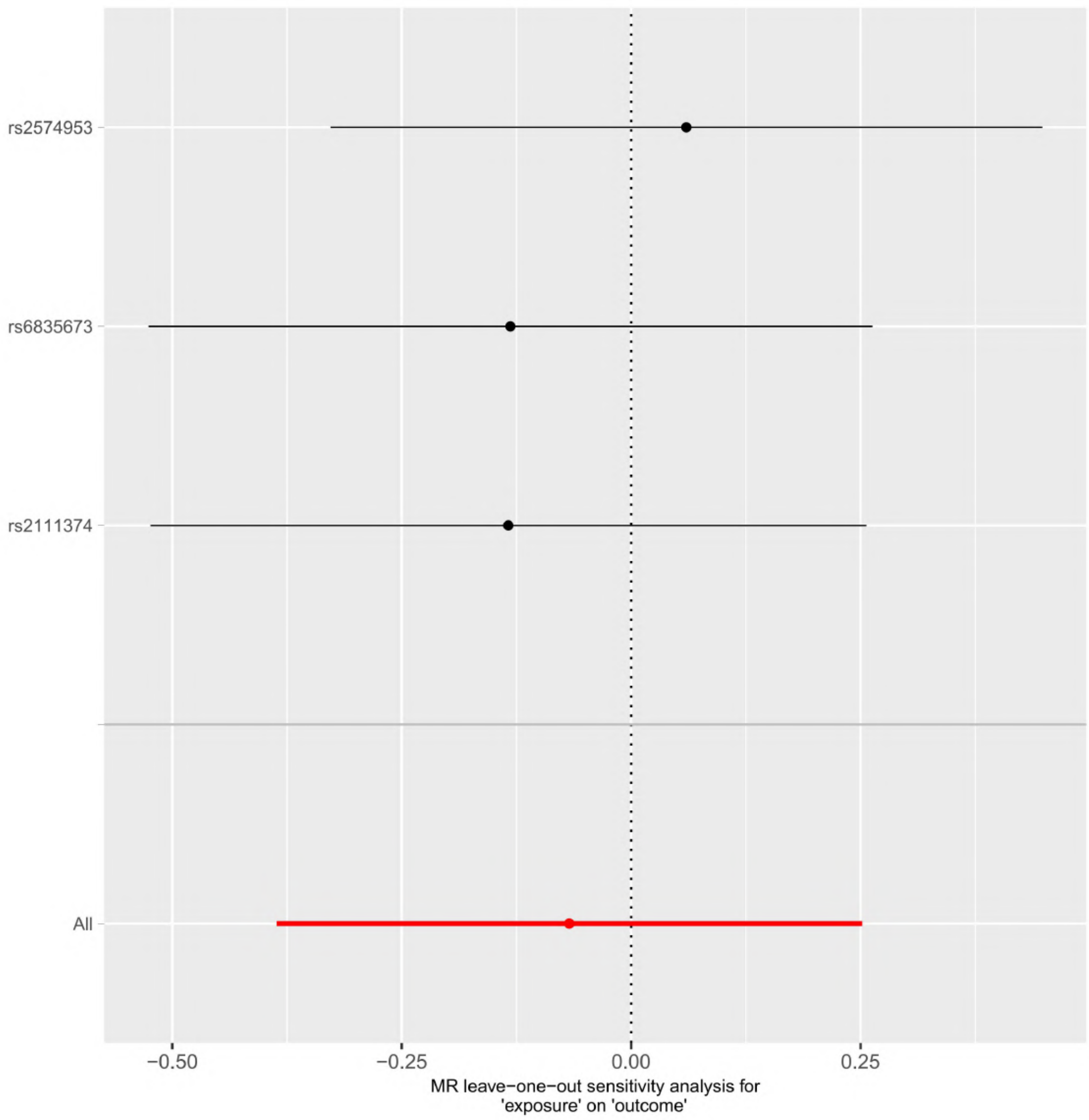

Supplementary Fig. 97 Leave-one-out inverse-variance weighted mendelian randomization analysis of garlic intake on GERD

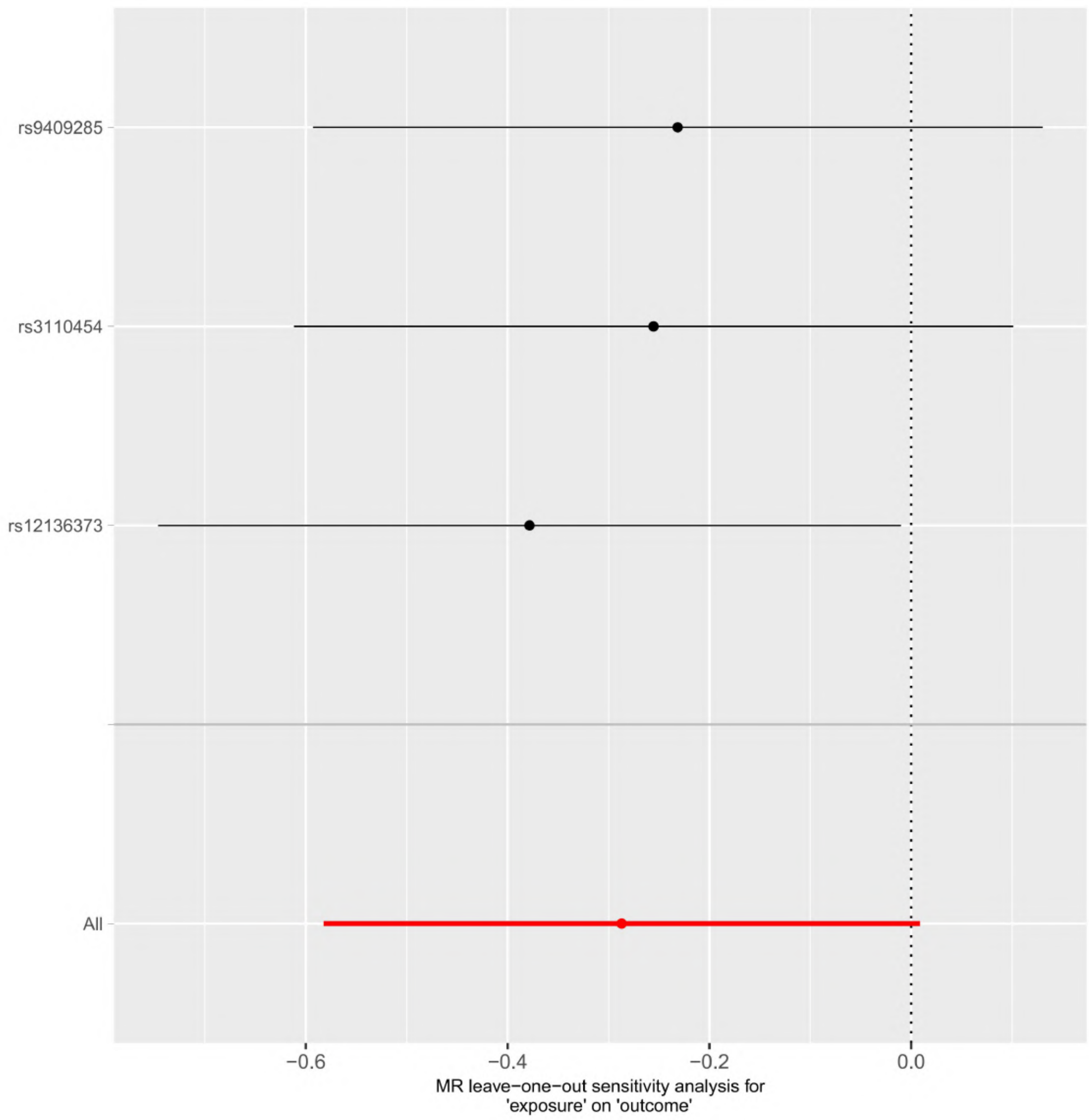

Supplementary Fig. 98 Leave-one-out inverse-variance weighted mendelian randomization analysis of celery intake on GERD

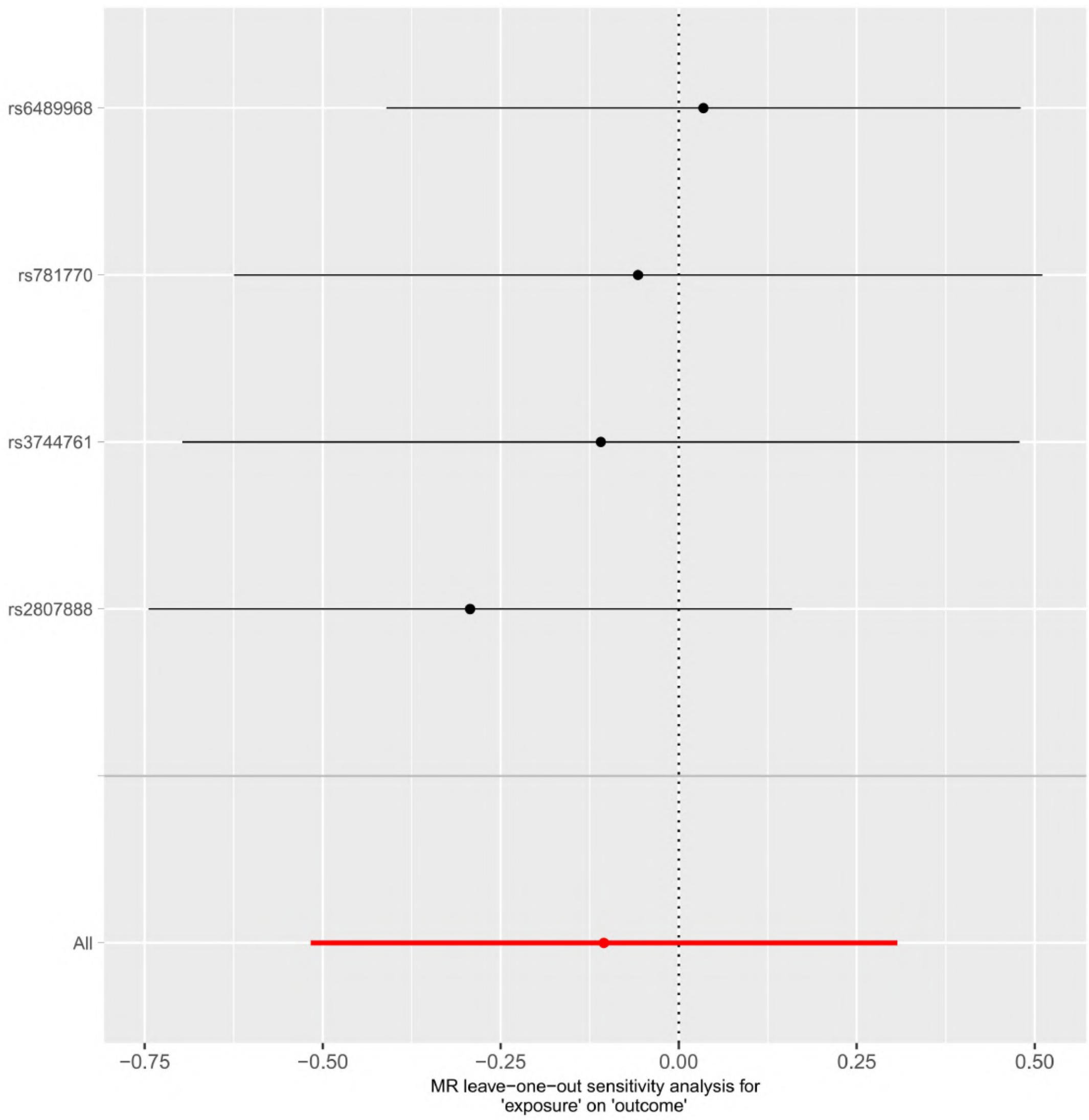

Supplementary Fig. 99 Leave-one-out inverse-variance weighted mendelian randomization analysis of milk intake on GERD

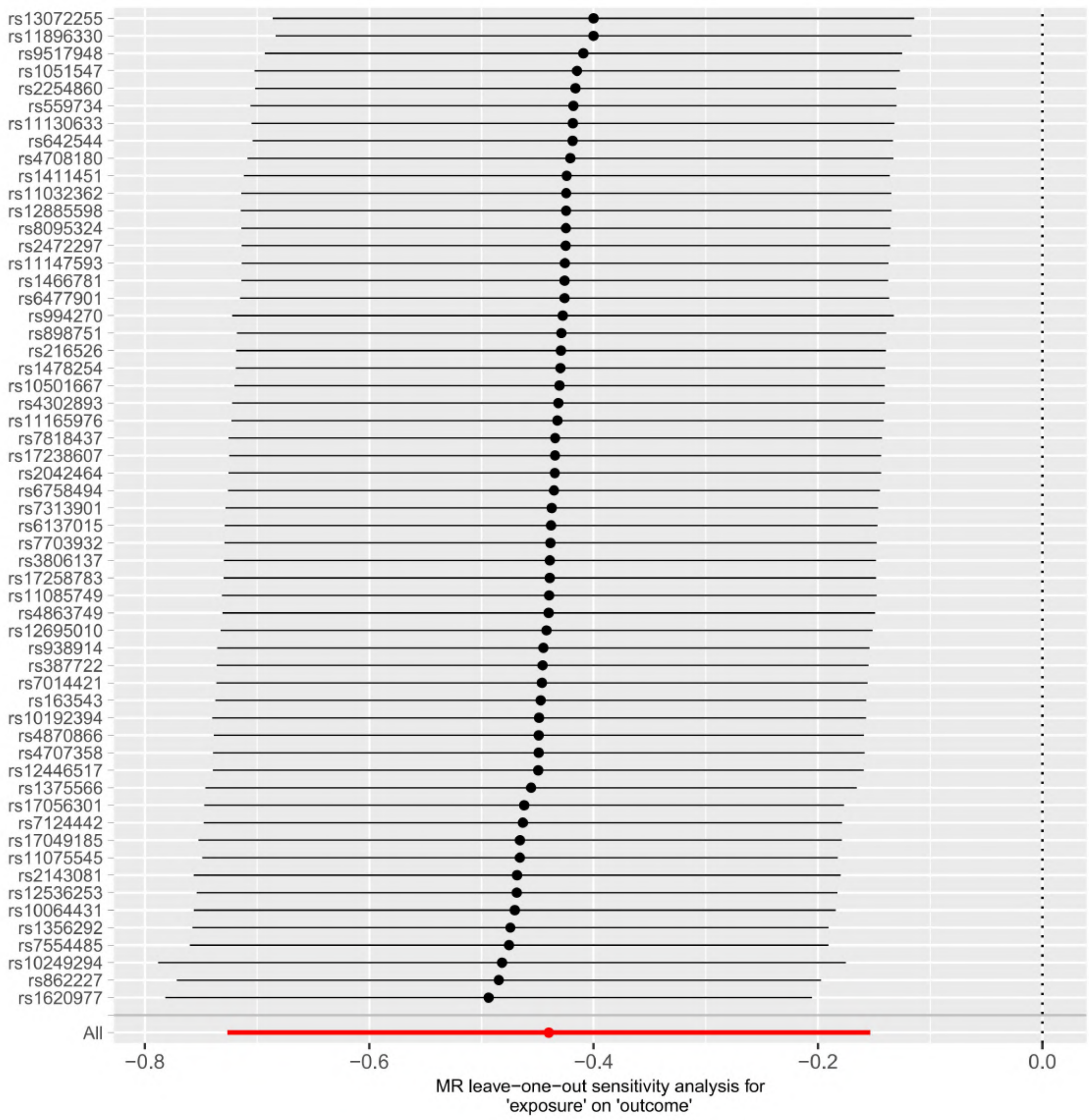

Supplementary Fig. 100 Leave-one-out inverse-variance weighted mendelian randomization analysis of fresh fruit intake on GERD

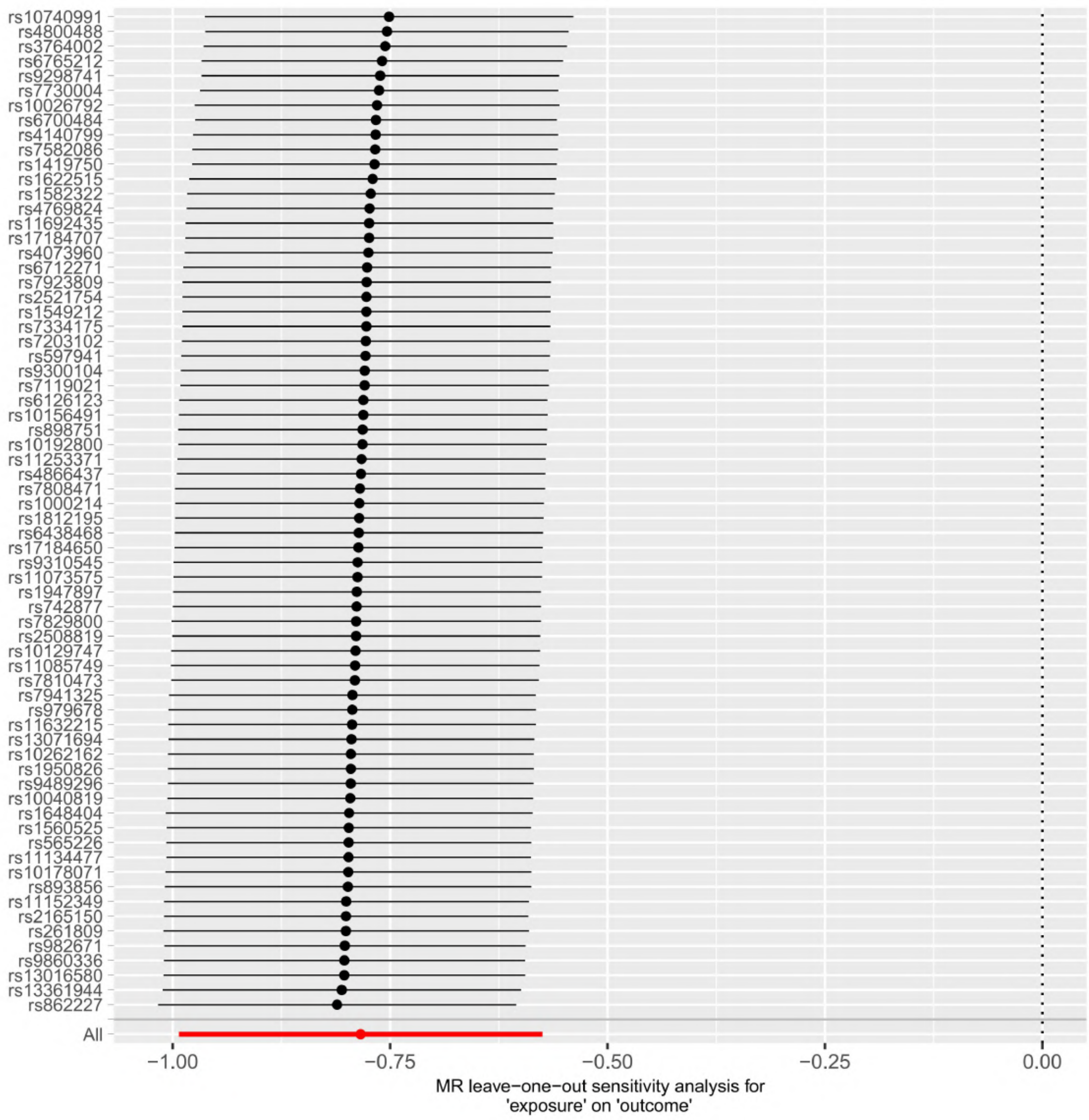

Supplementary Fig. 101 Leave-one-out inverse-variance weighted mendelian randomization analysis of dried fruit intake on GERD

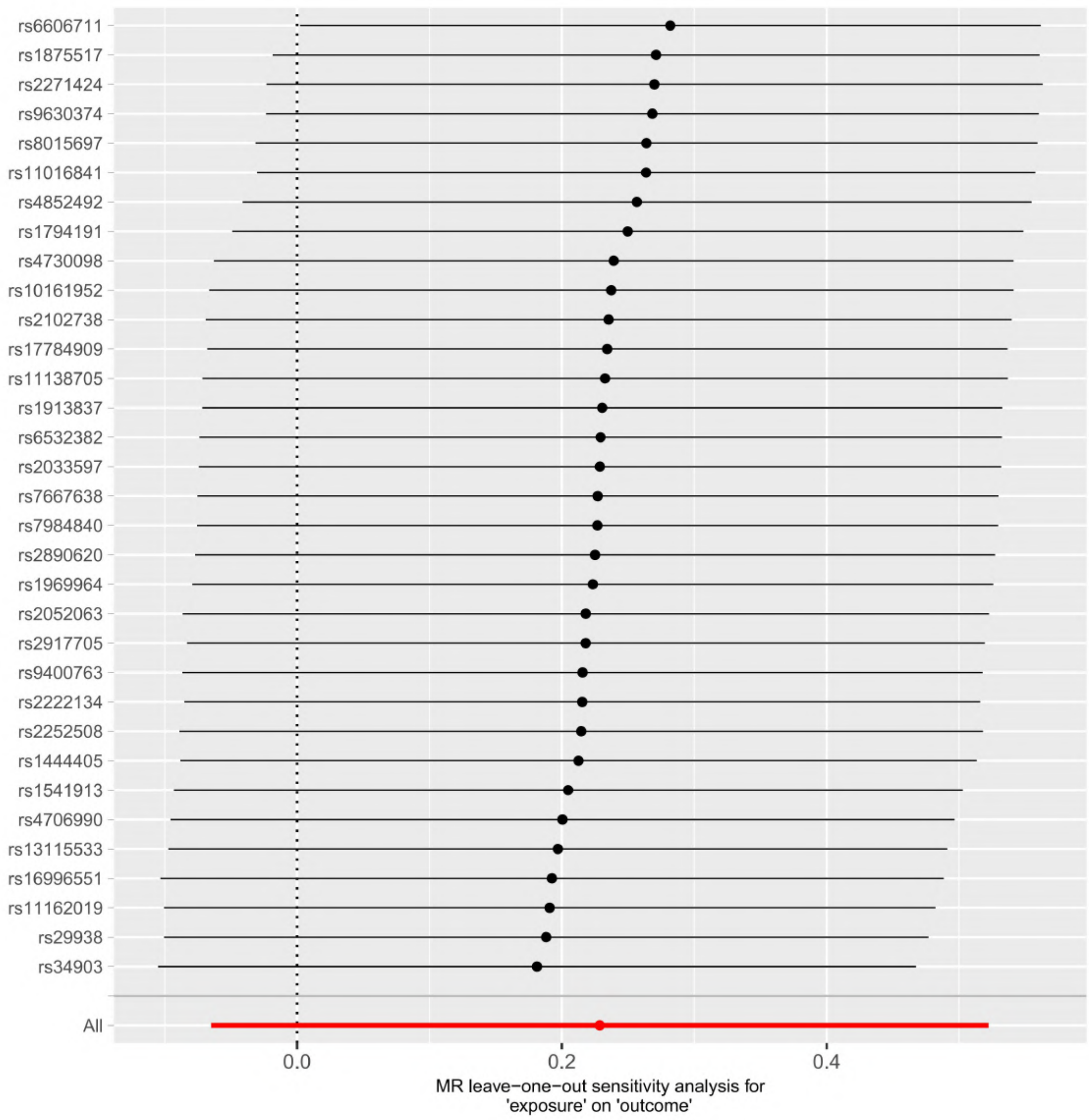

Supplementary Fig. 102 Leave-one-out inverse-variance weighted mendelian randomization analysis of cooked vegetable intake on GERD

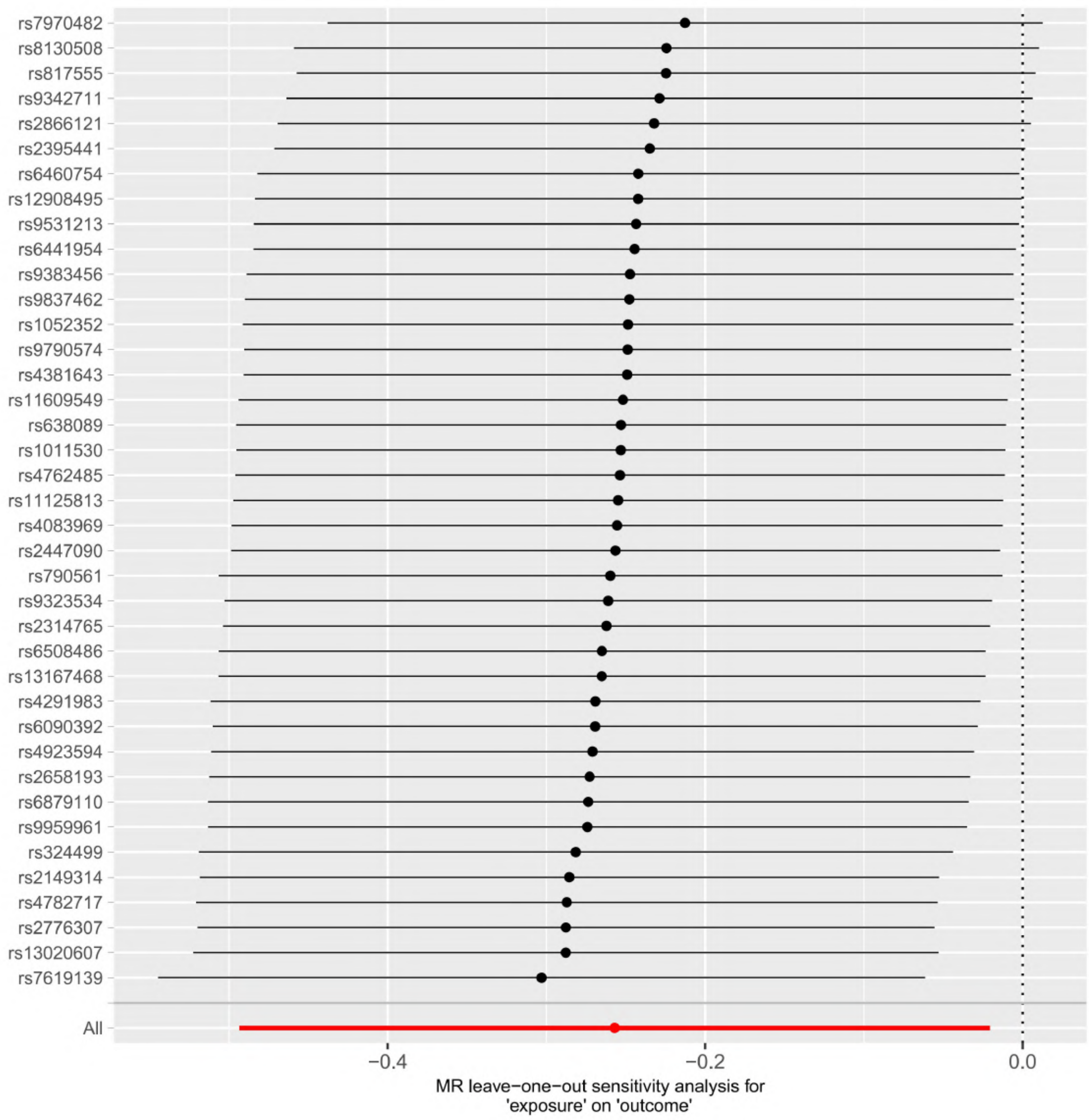

Supplementary Fig. 103 Leave-one-out inverse-variance weighted mendelian randomization analysis of salad/raw vegetable intake on GERD

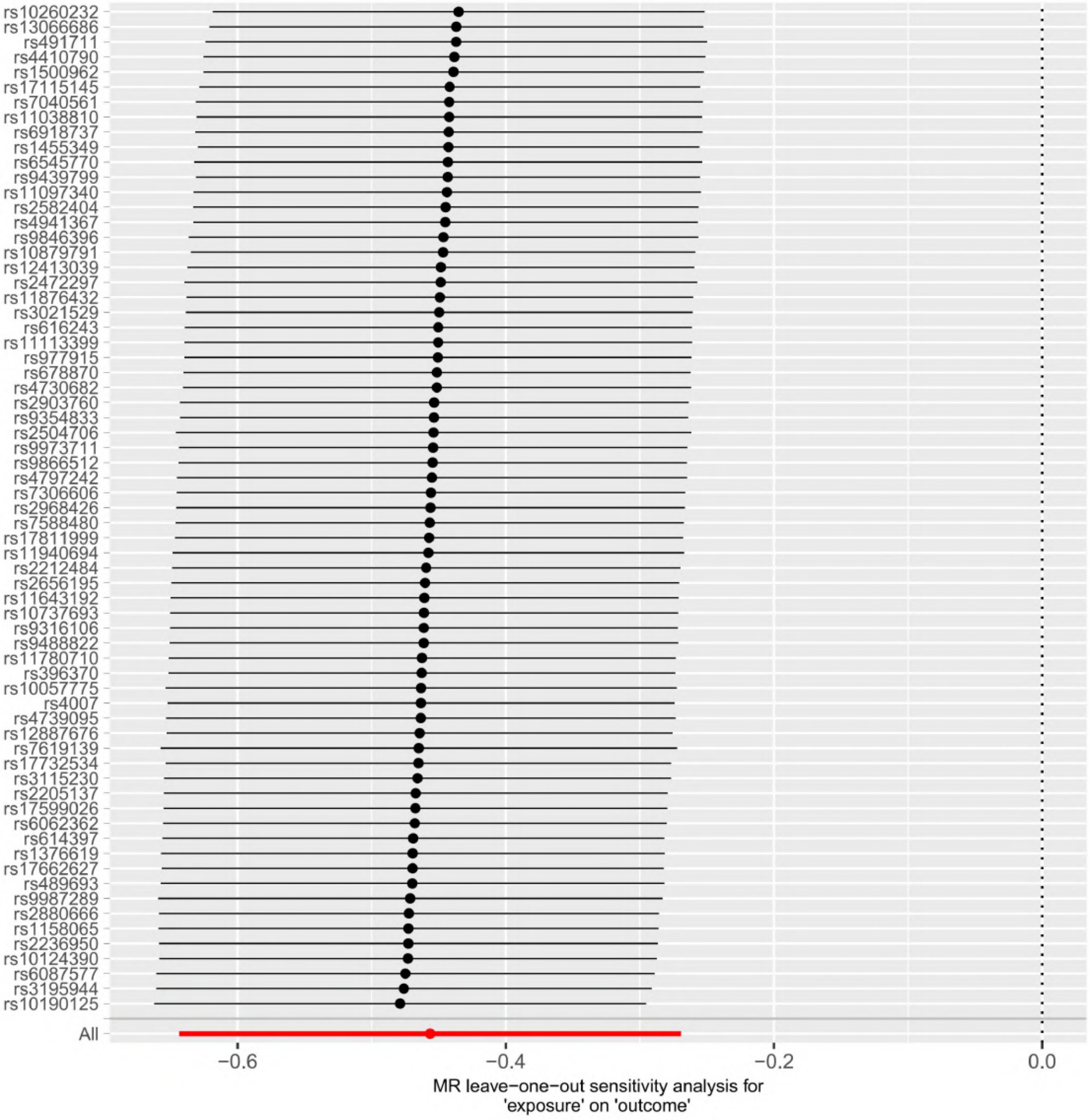

Supplementary Fig. 104 Leave-one-out inverse-variance weighted mendelian randomization analysis of cereal intake on GERD

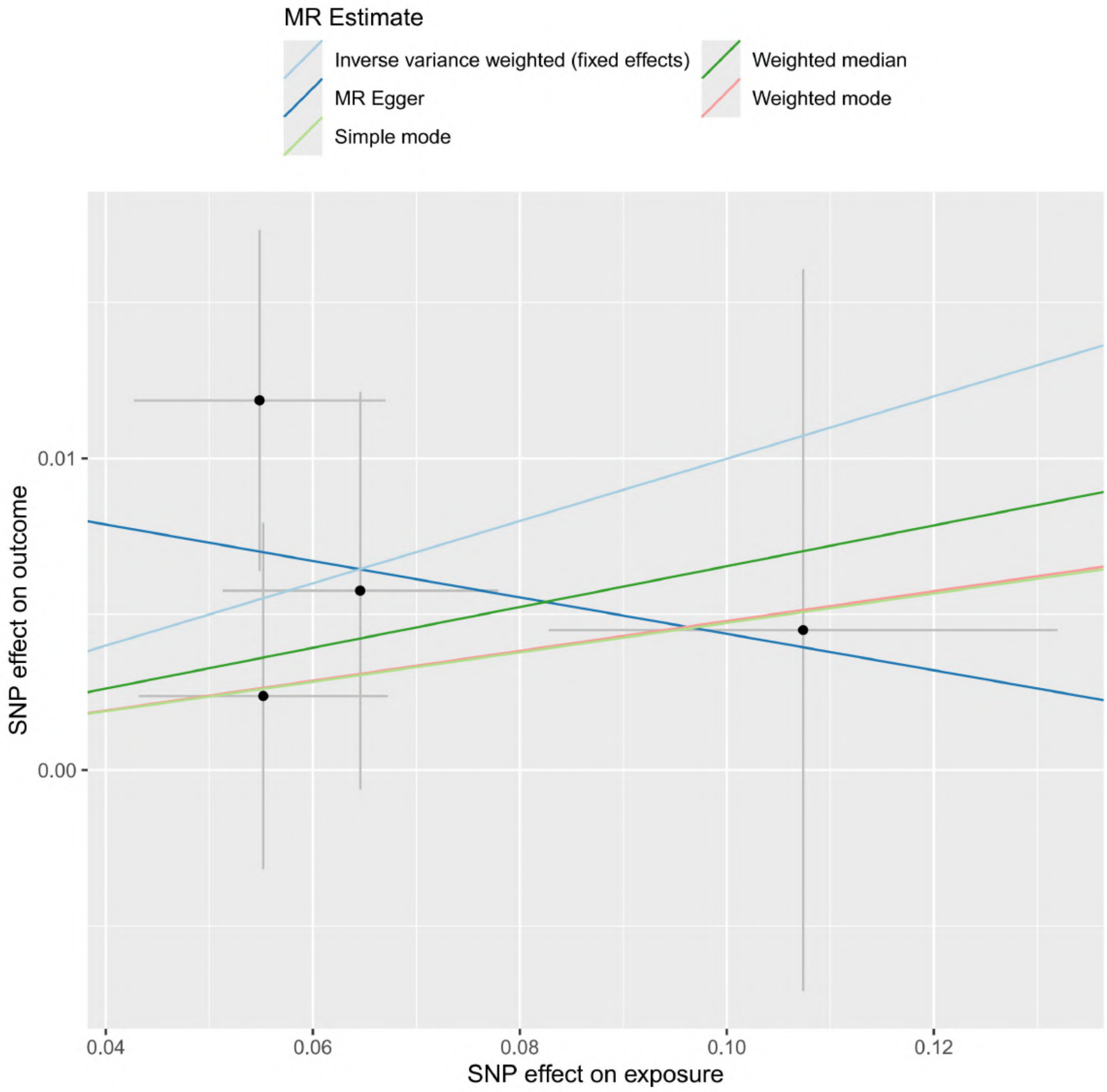

Supplementary Fig. 105 Scatter plot of the causal effect of class.Bacteroidia.id.912 on GERD

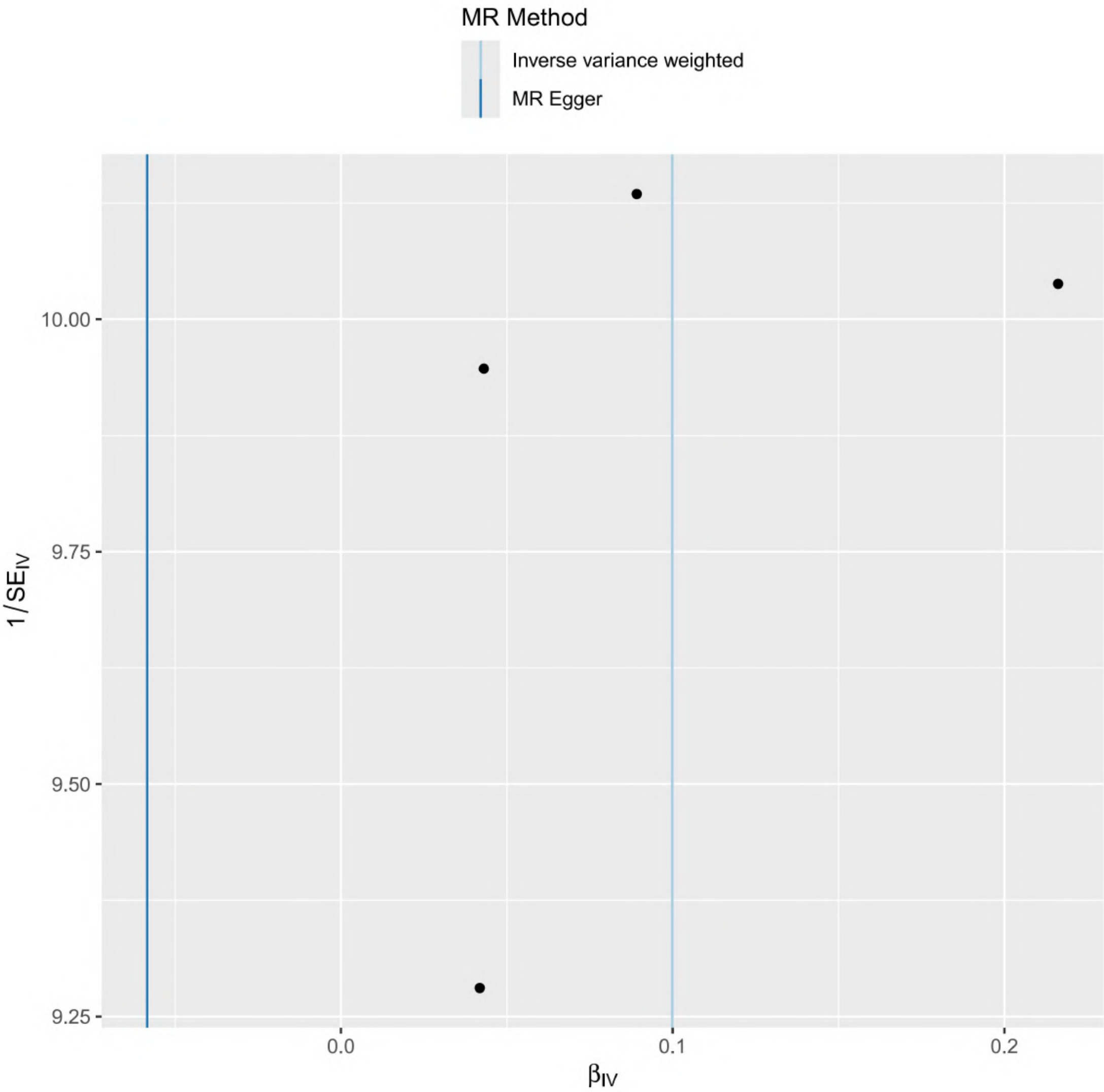

Supplementary Fig. 106 Funnel plot of the causal effect of class.Bacteroidia.id.912 on GERD

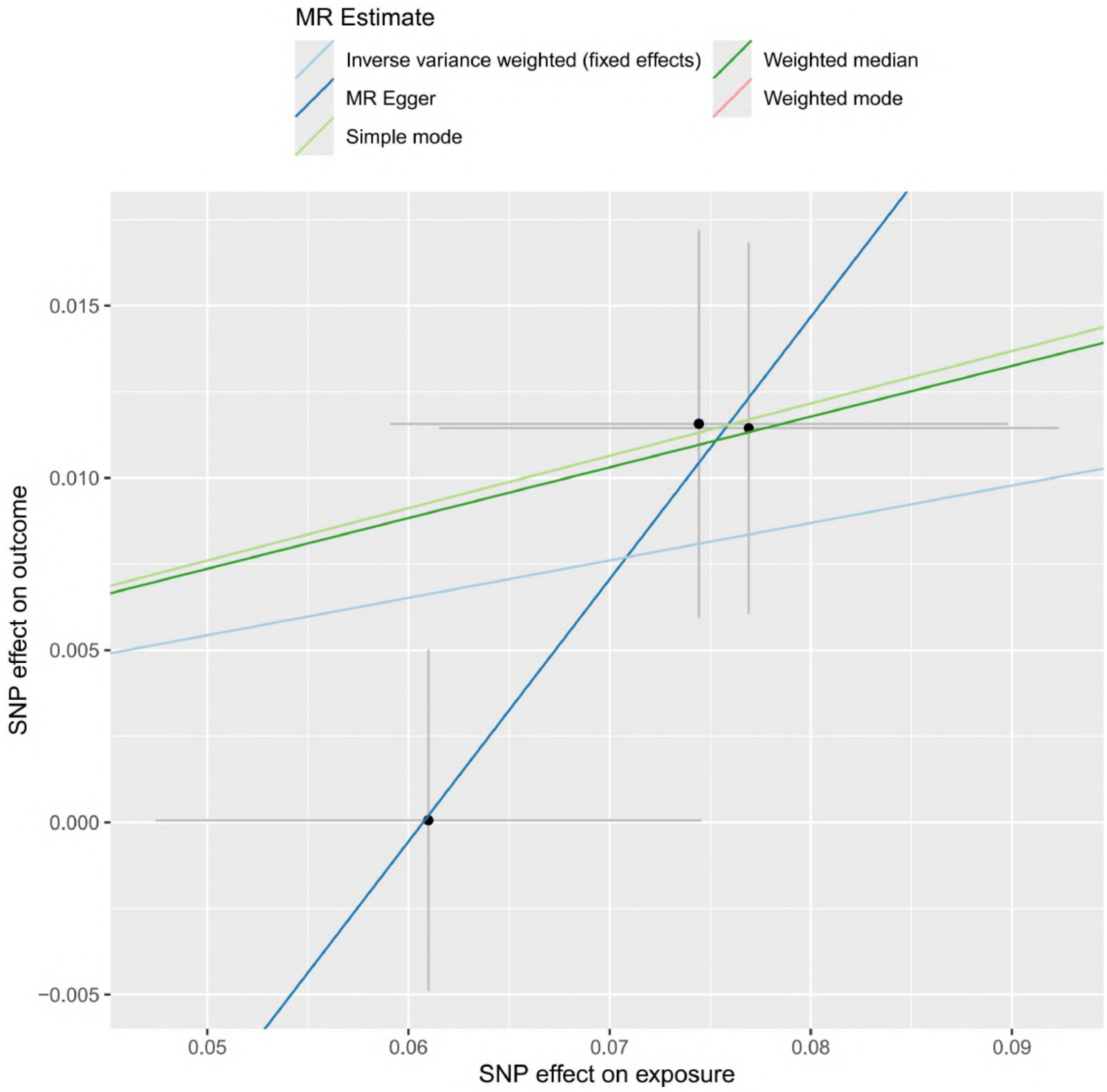

Supplementary Fig. 107 Scatter plot of the causal effect of class.Mollicutes.id.3920 on GERD

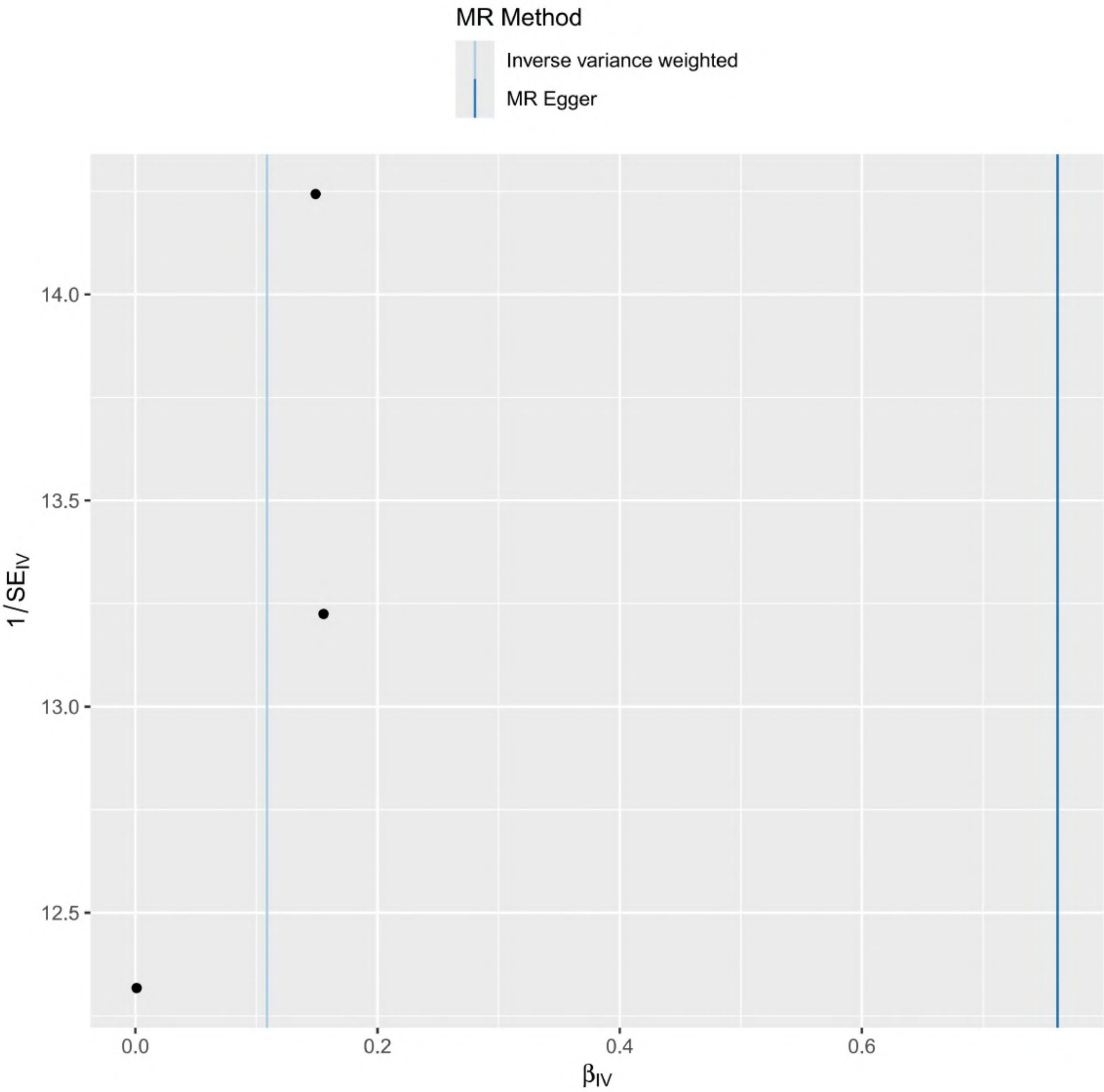

Supplementary Fig. 108 Funnel plot of the causal effect of class.Mollicutes.id.3920 on GERD

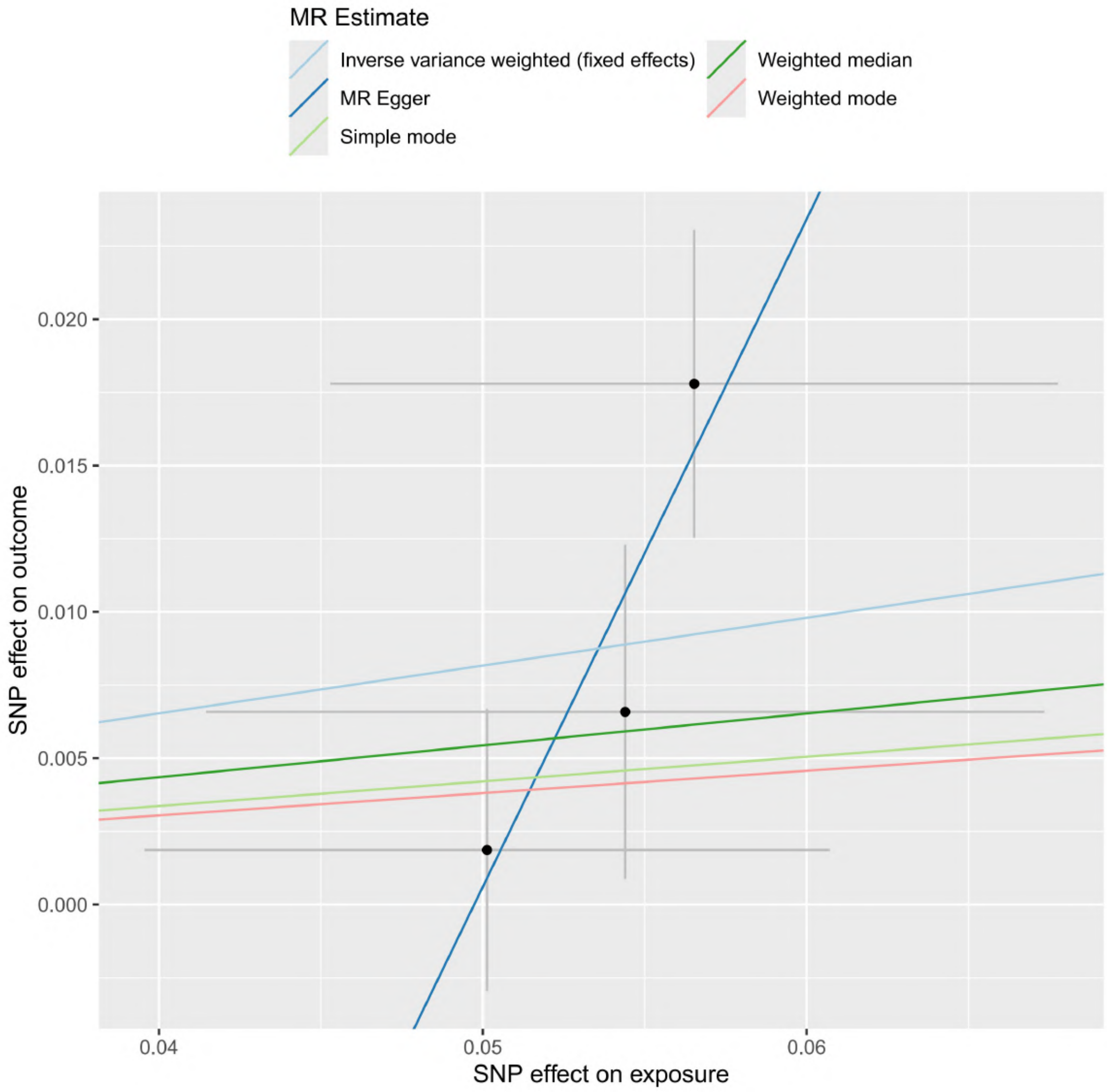

Supplementary Fig. 109 Scatter plot of the causal effect of class.Negativicutes.id.2164 on GERD

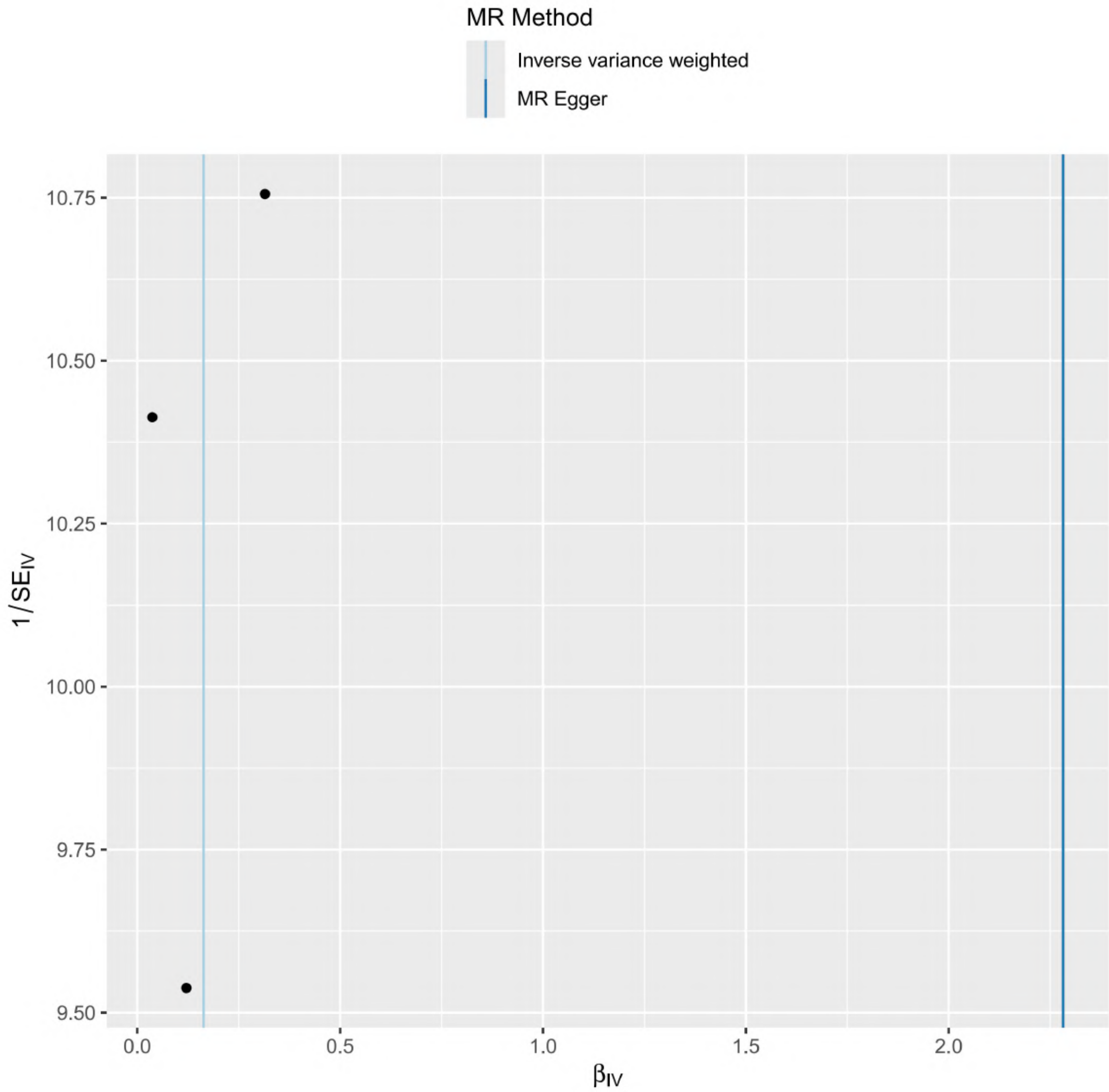

Supplementary Fig. 110 Funnel plot of the causal effect of class.Negativicutes.id.2164 on GERD

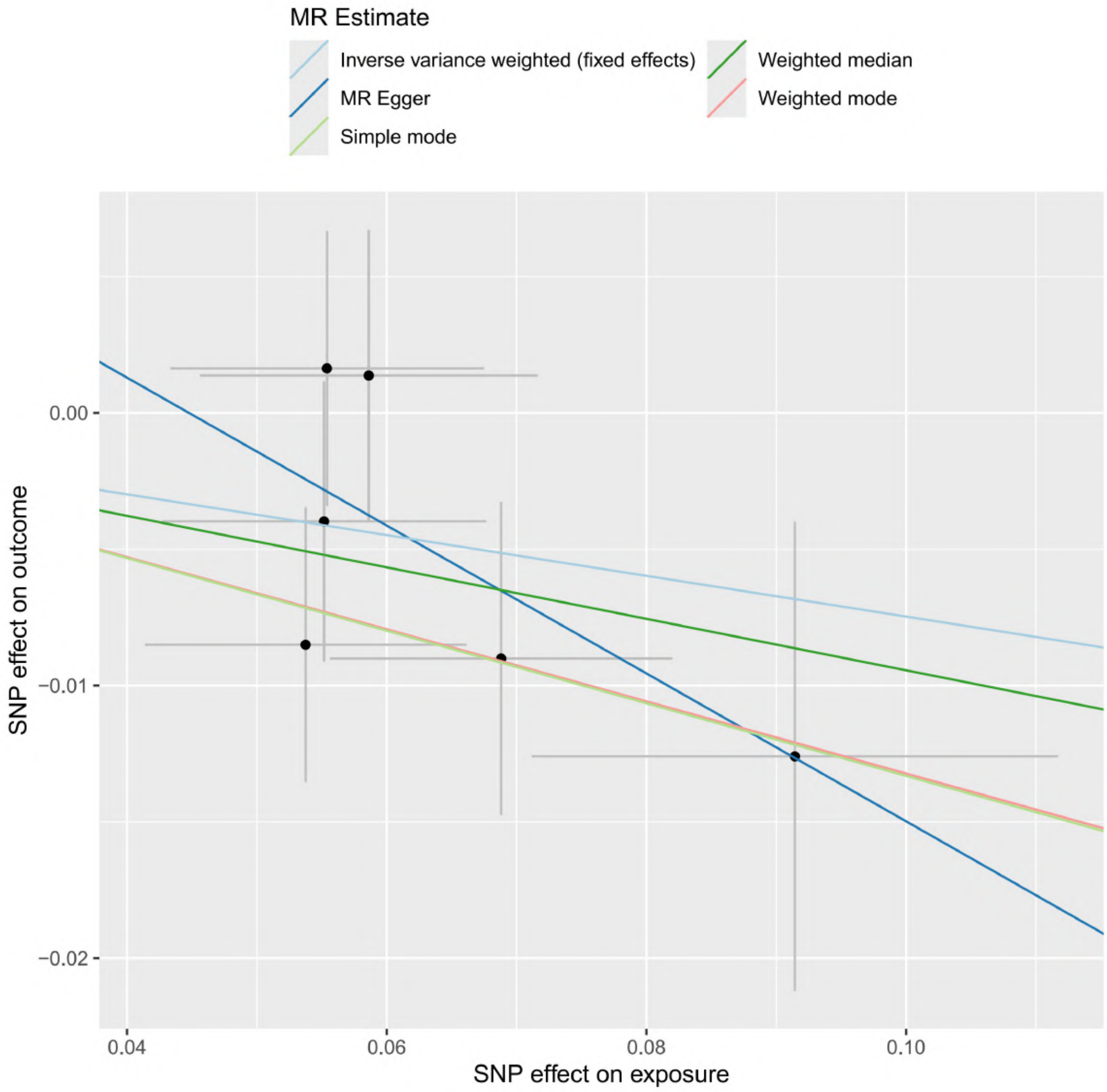

Supplementary Fig. 111 Scatter plot of the causal effect of family.Bifidobacteriaceae.id.433 on GERD

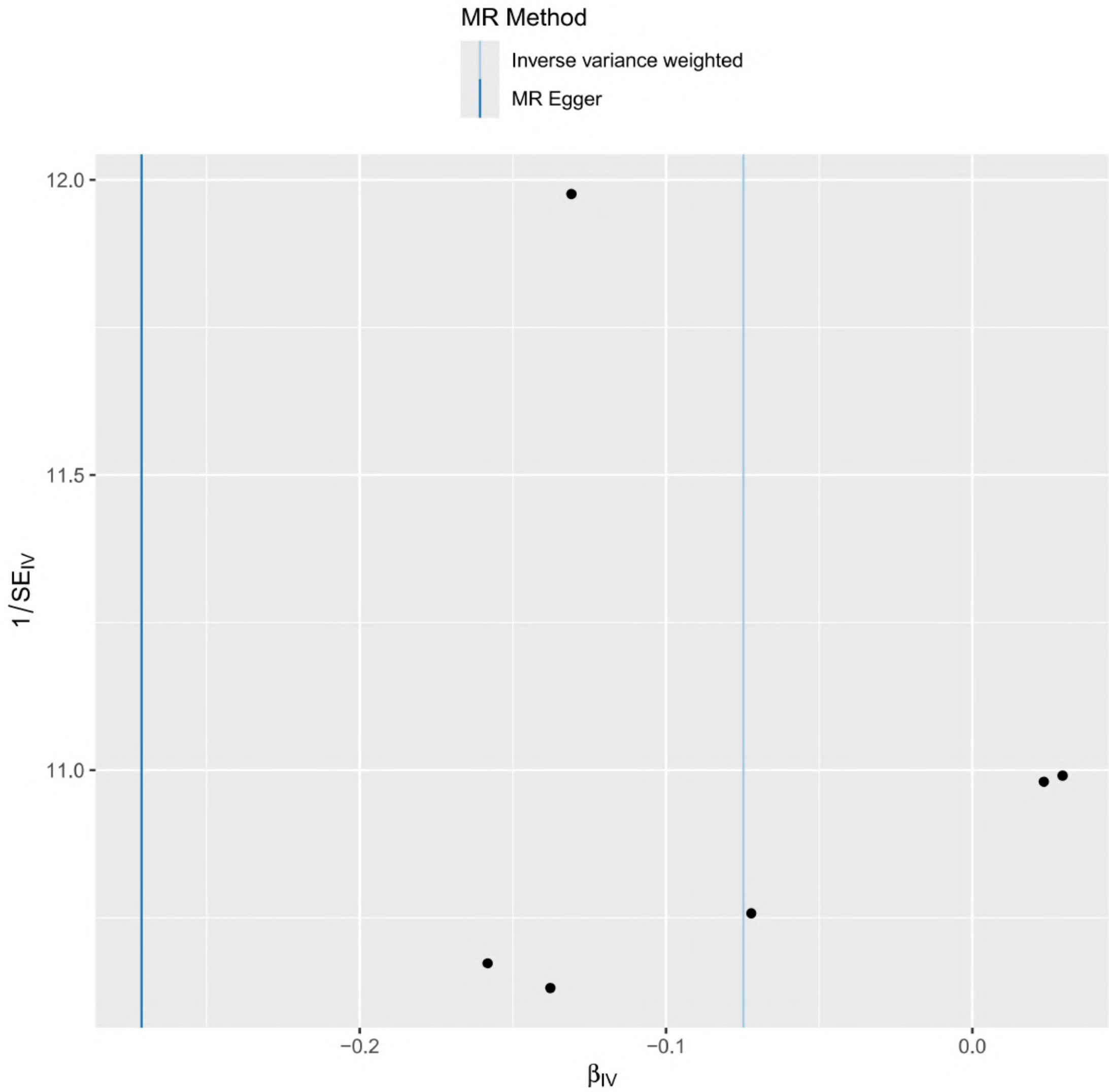

Supplementary Fig. 112 Funnel plot of the causal effect of family.Bifidobacteriaceae.id.433 on GERD

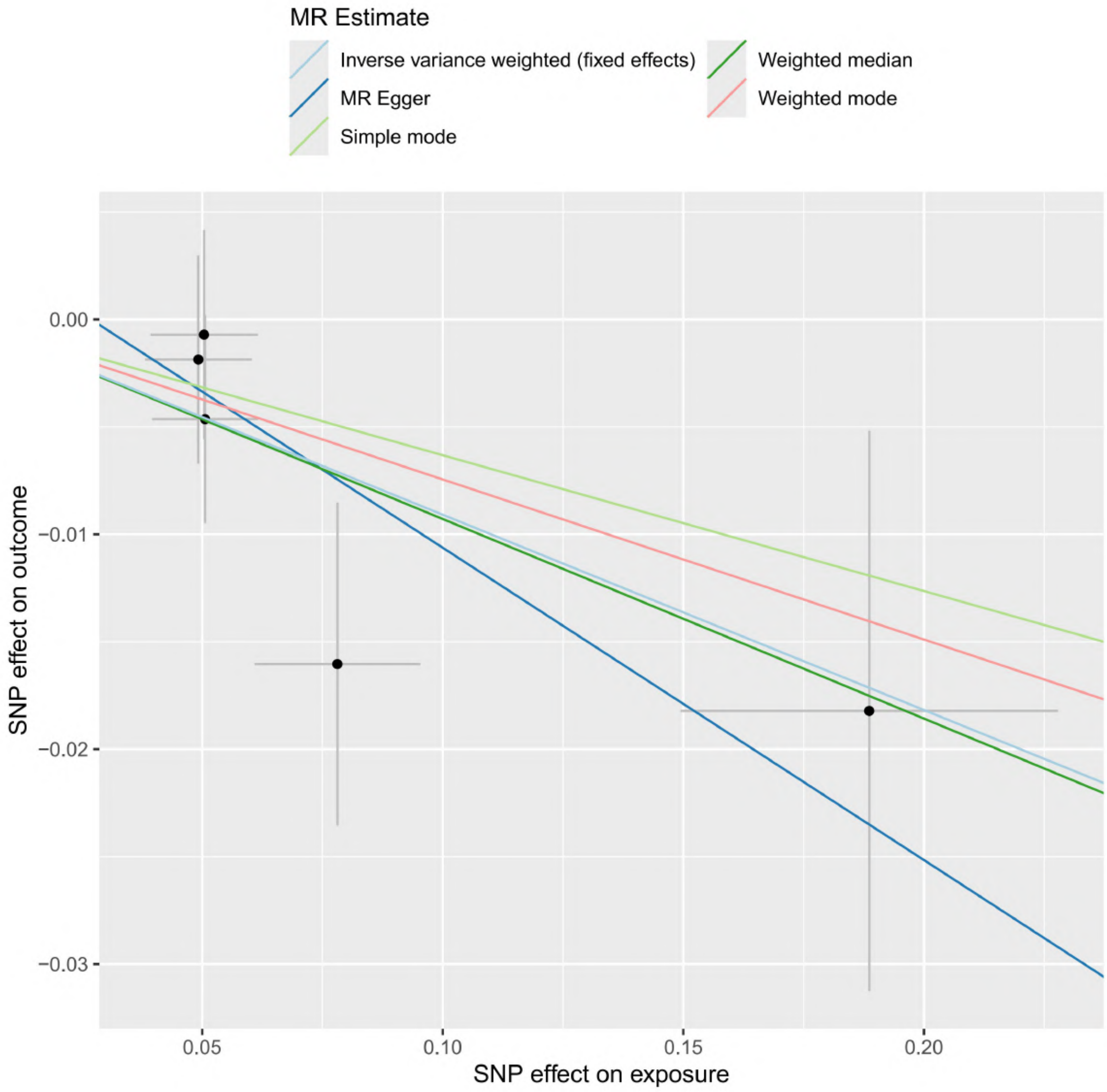

Supplementary Fig. 113 Scatter plot of the causal effect of family.Christensenellaceae.id.1866 on GERD

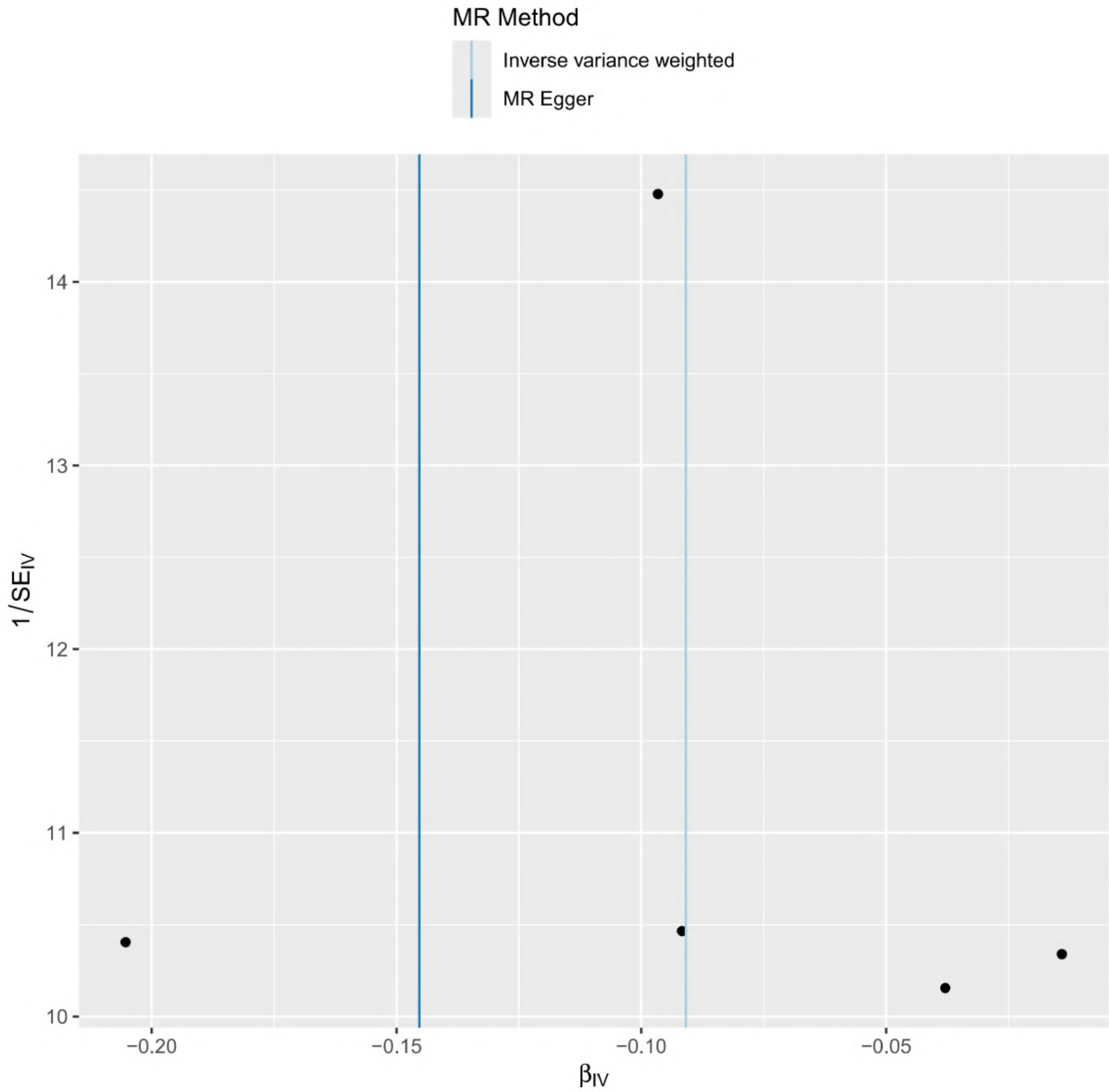

Supplementary Fig. 114 Funnel plot of the causal effect of family.Christensenellaceae.id.1866 on GERD

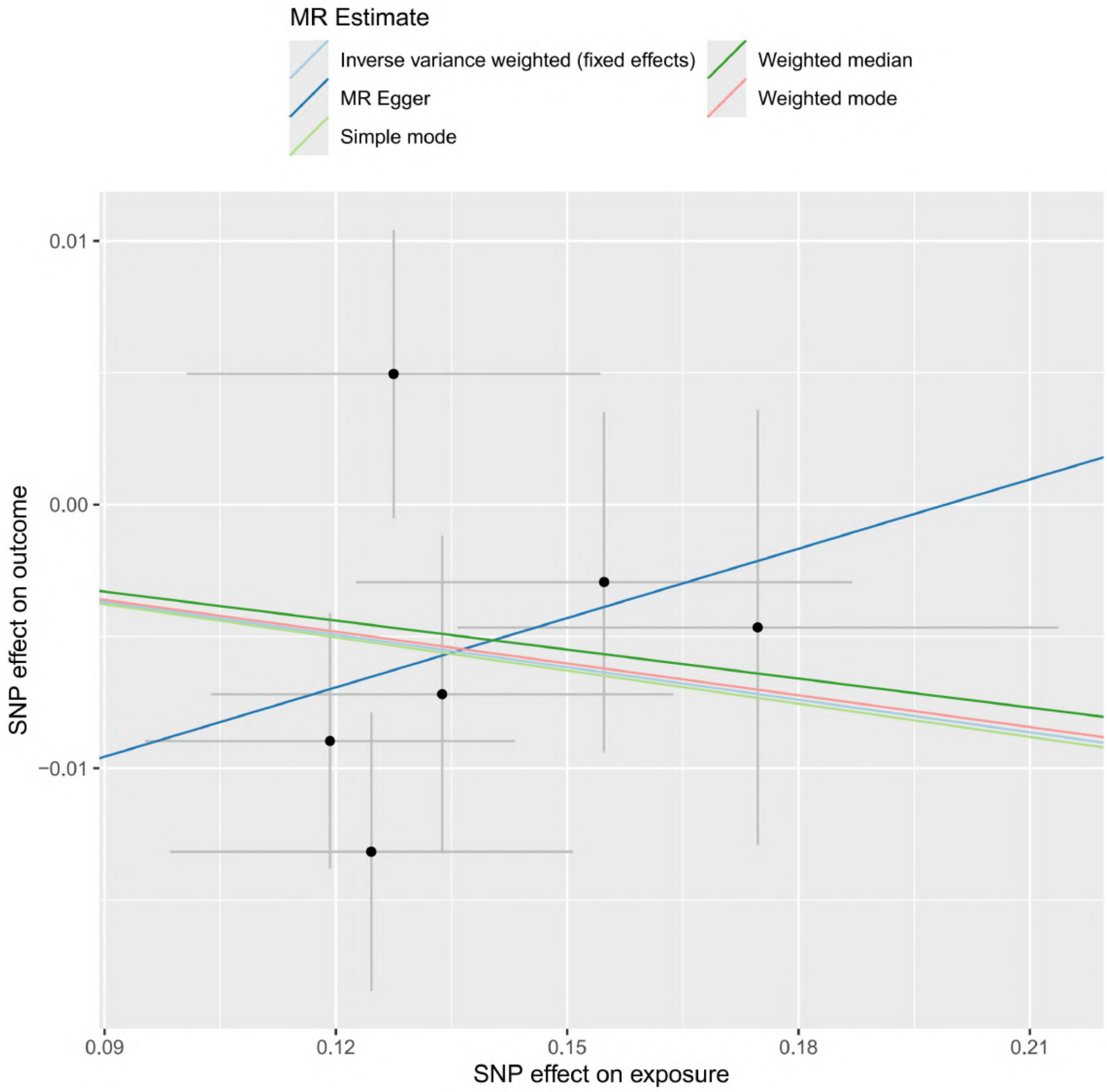

Supplementary Fig. 115 Scatter plot of the causal effect of genus.Butyrivibrio.id.1993 on GERD

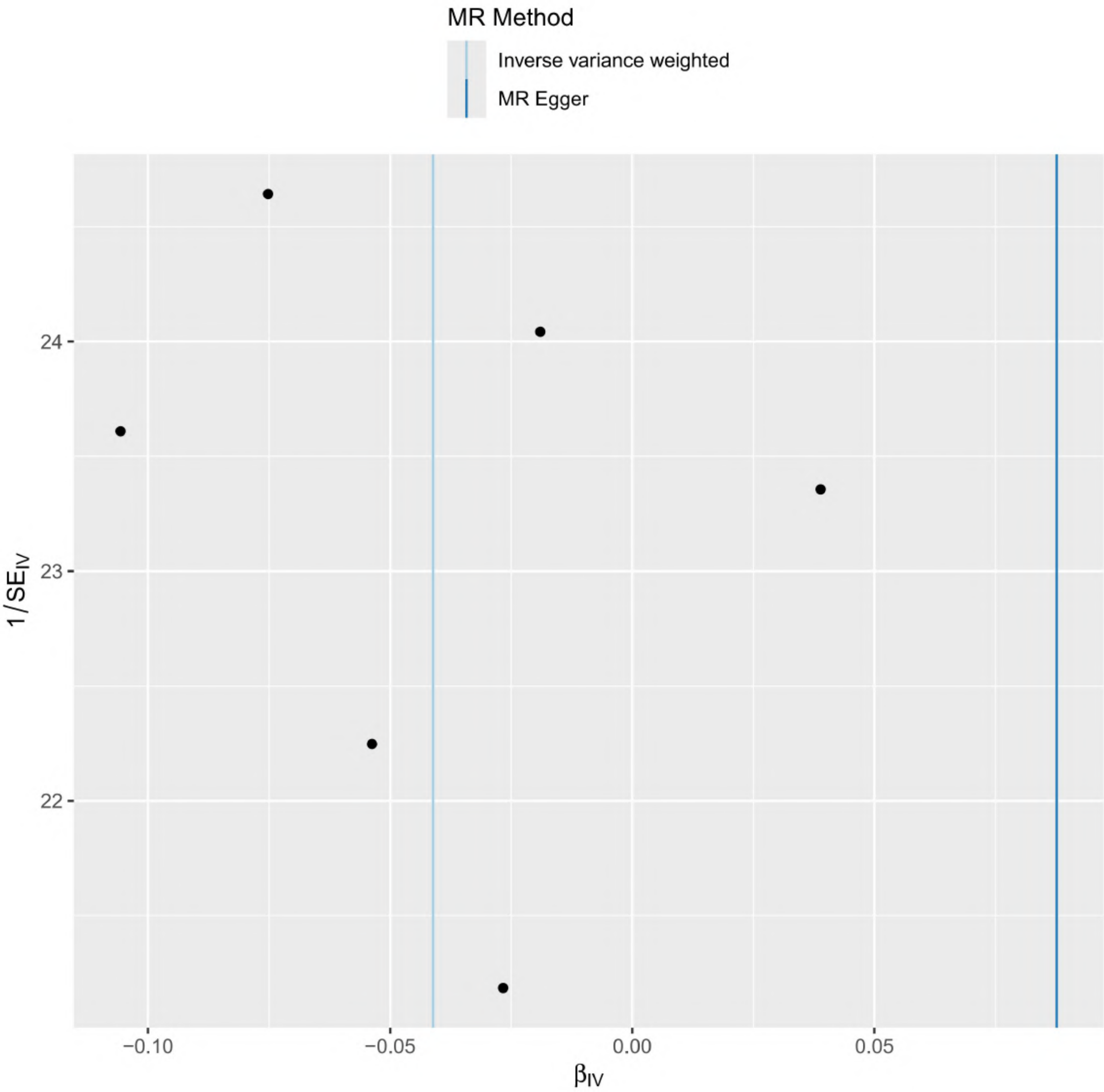

Supplementary Fig. 116 Funnel plot of the causal effect of genus.Butyrivibrio.id.1993 on GERD

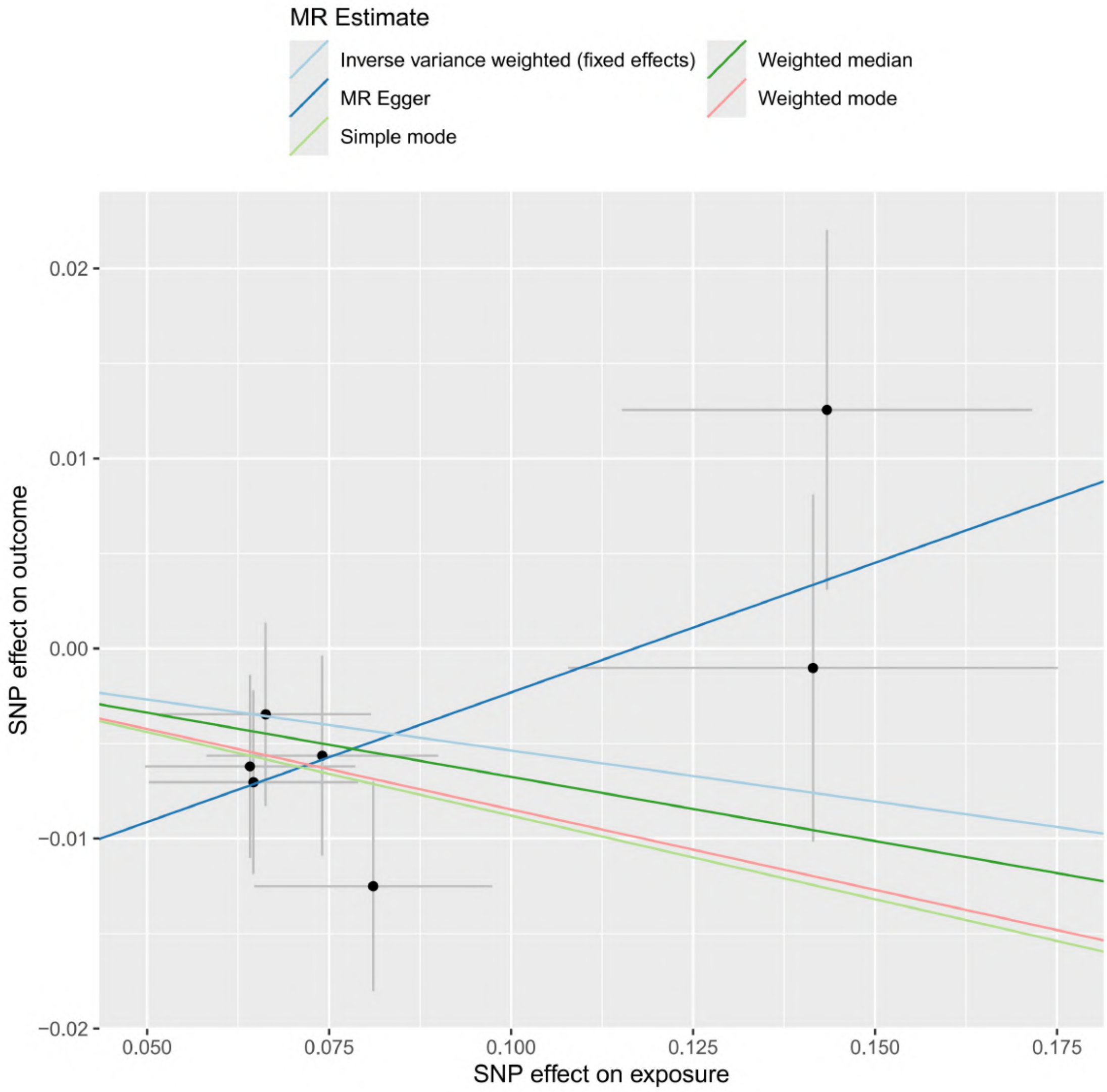

Supplementary Fig. 117 Scatter plot of the causal effect of genus.Erysipelatoclostridium.id.11381 on GERD

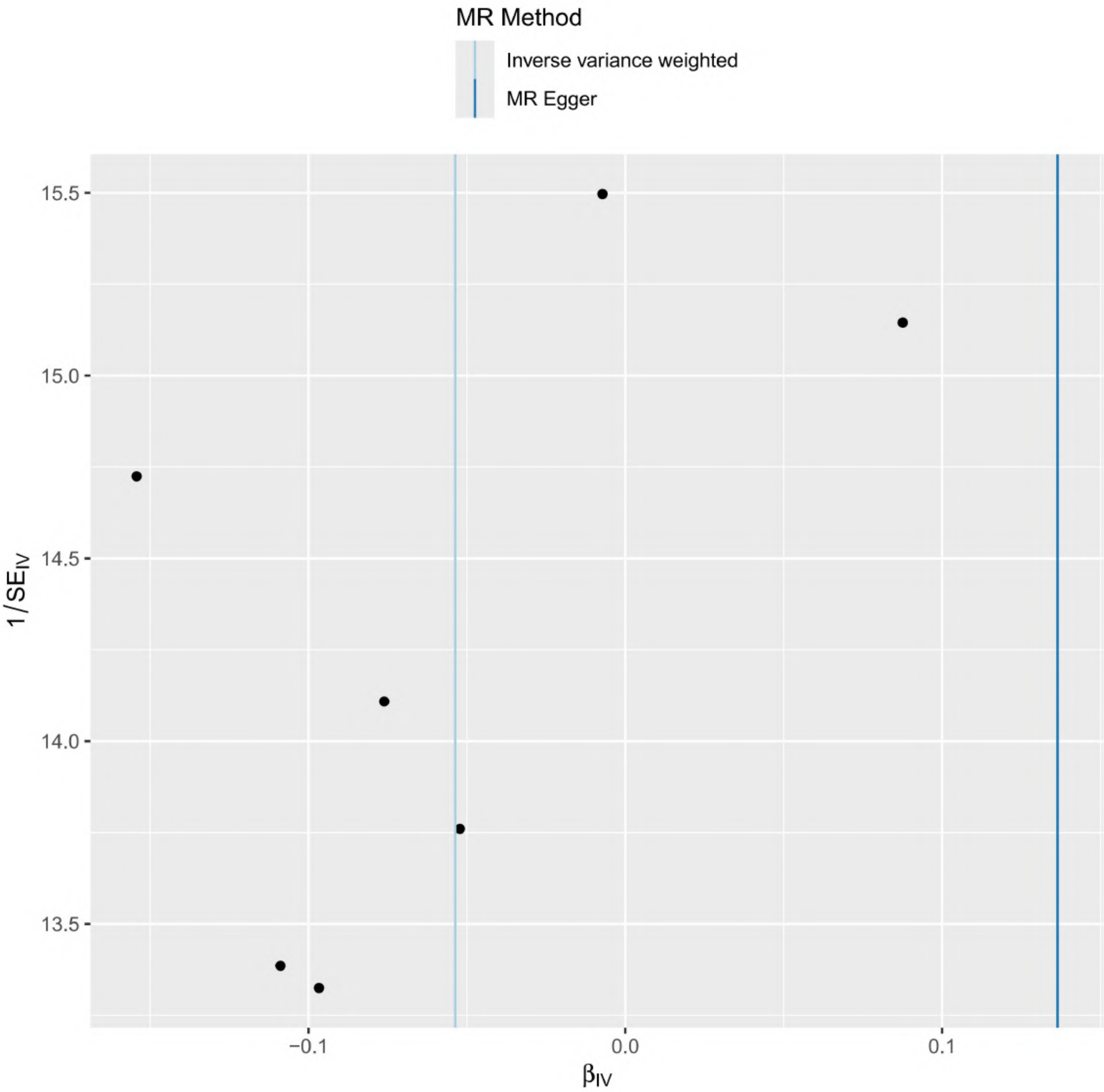

Supplementary Fig. 118 Funnel plot of the causal effect of genus.Erysipelatoclostridium.id.11381 on GERD

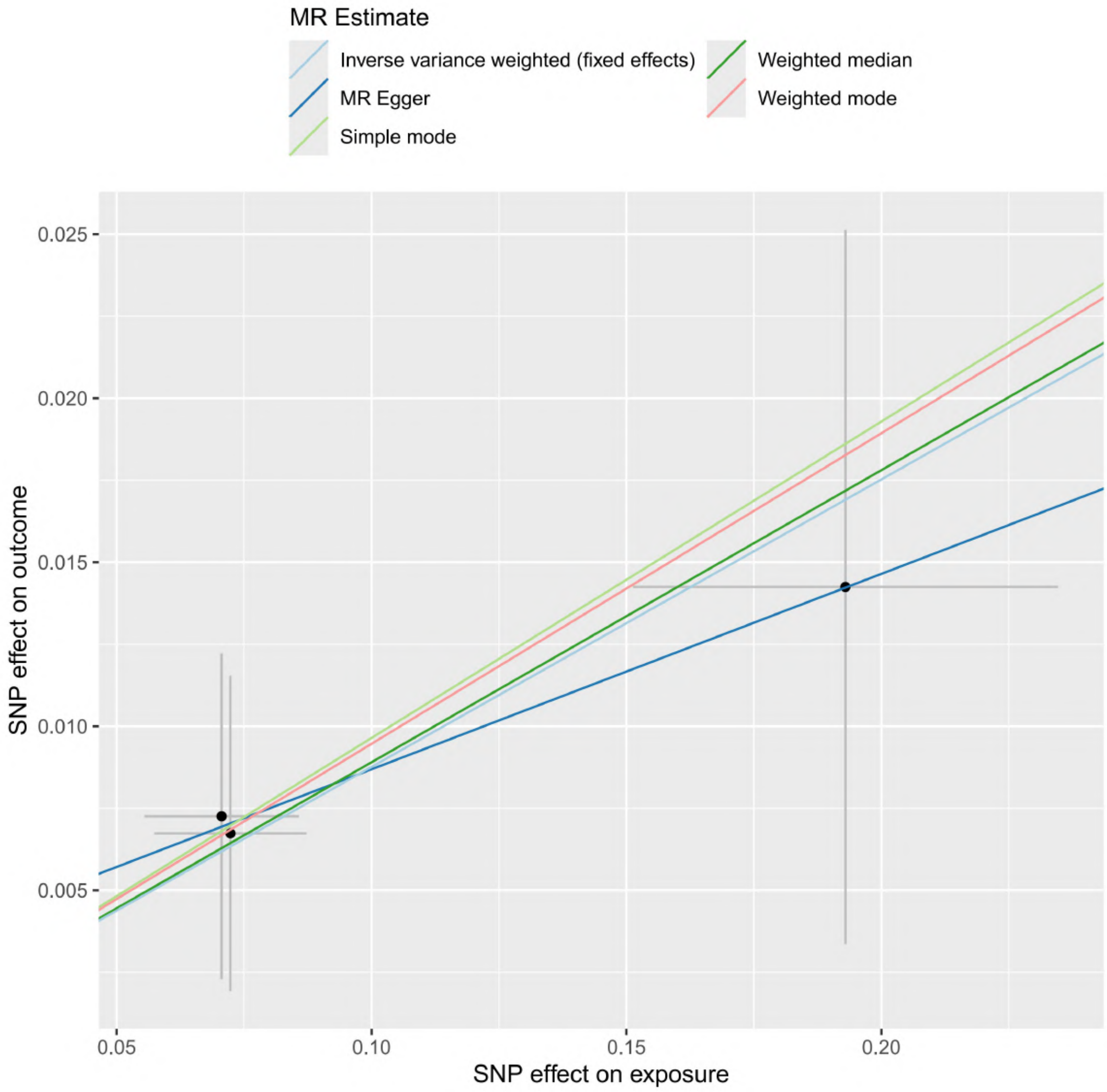

Supplementary Fig. 119 Scatter plot of the causal effect of genus.Haemophilus.id.3698 on GERD

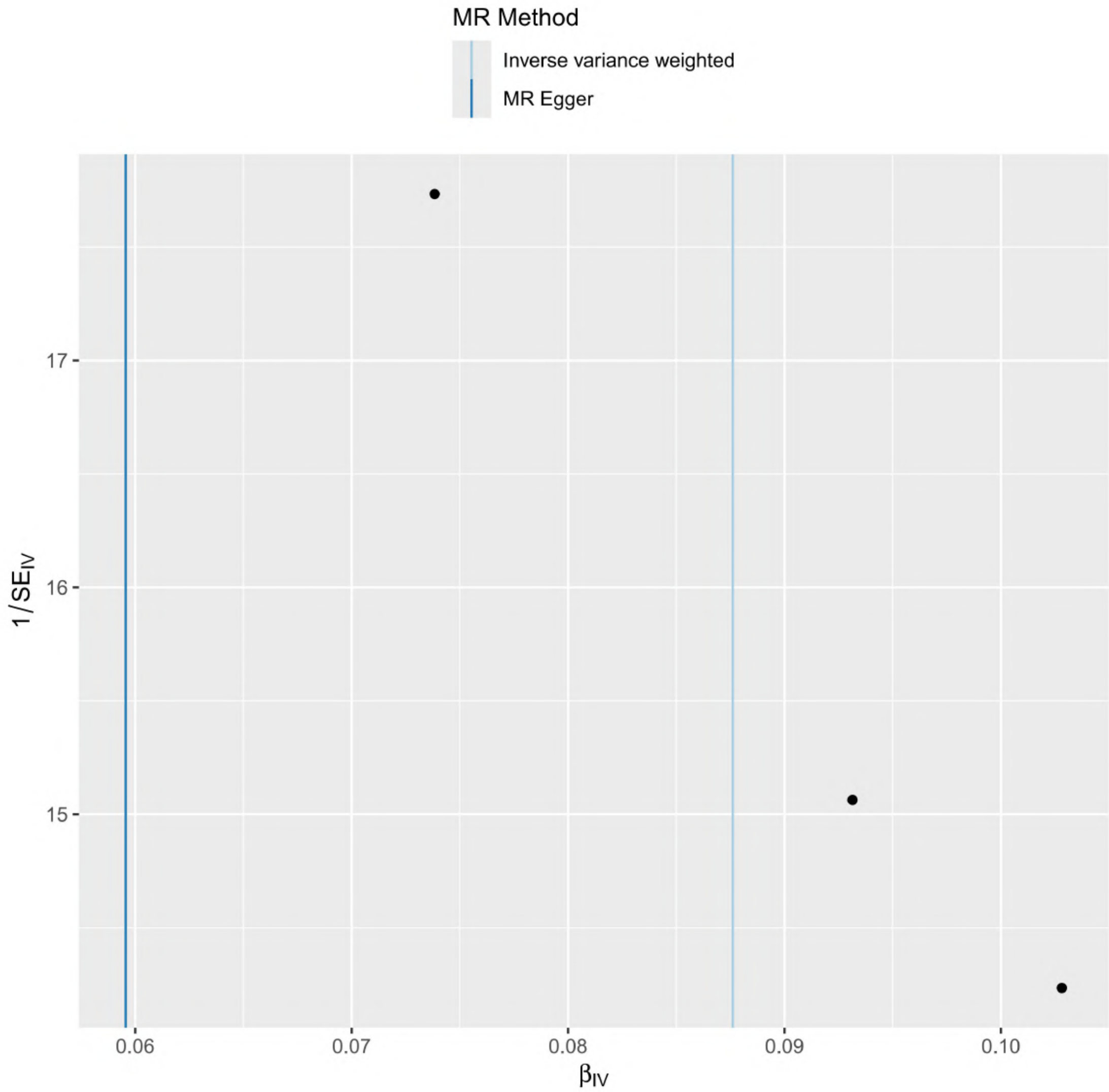

Supplementary Fig. 120 Funnel plot of the causal effect of genus.Haemophilus.id.3698 on GERD

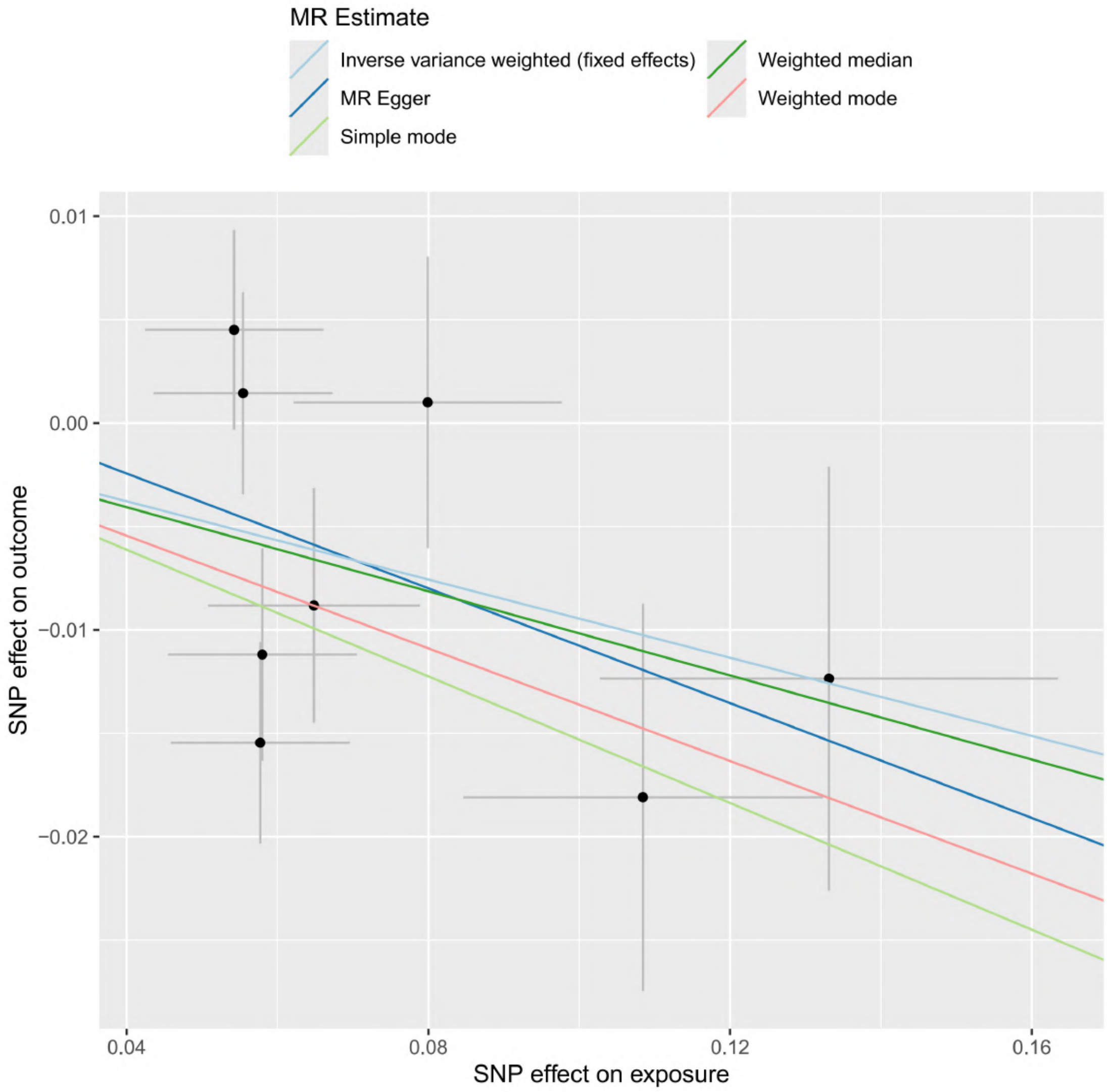

Supplementary Fig. 121 Scatter plot of the causal effect of genus.LachnospiraceaeUCG004.id.11324 on GERD

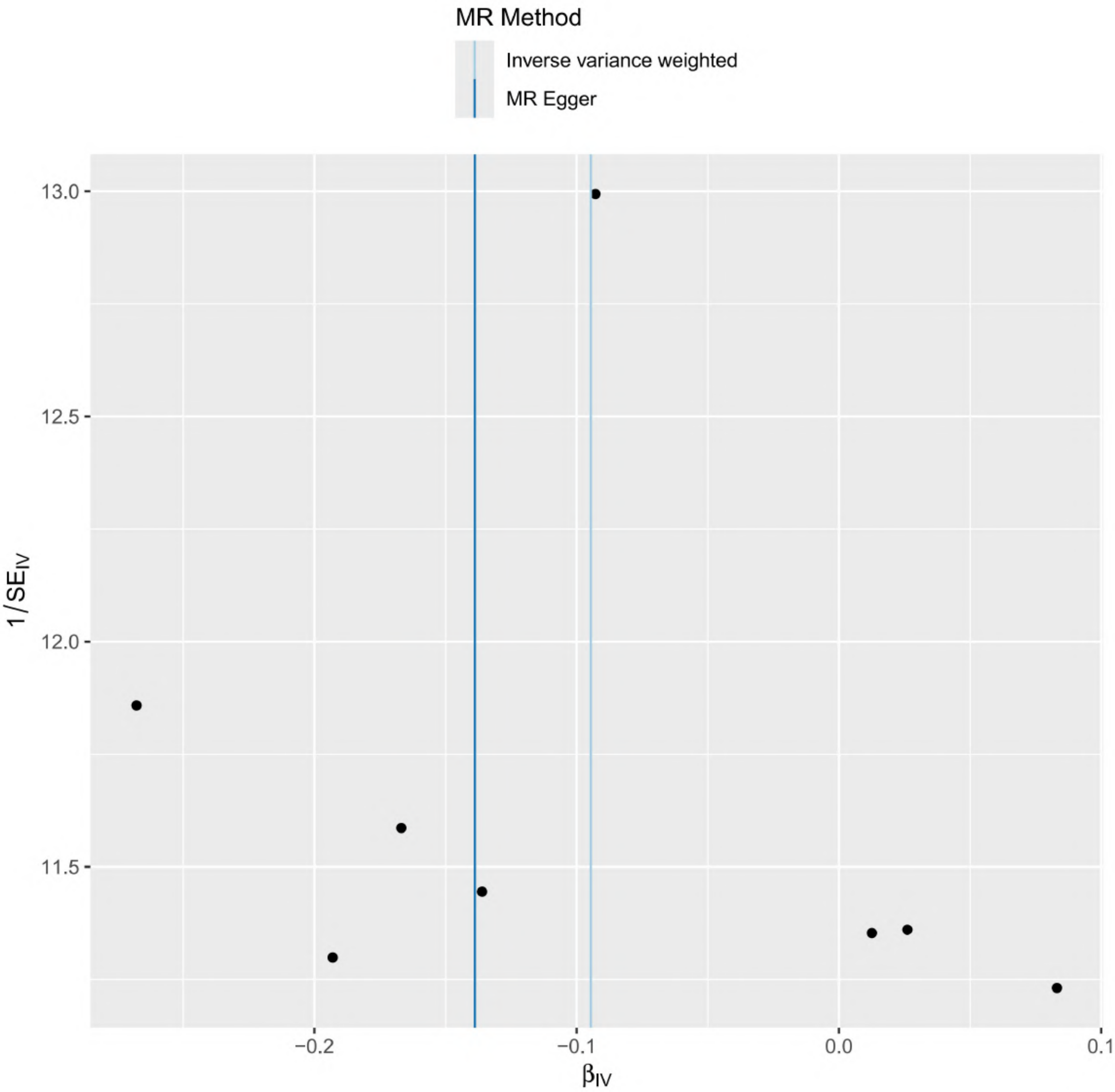

Supplementary Fig. 122 Funnel plot of the causal effect of genus.LachnospiraceaeUCG004.id.11324 on GERD

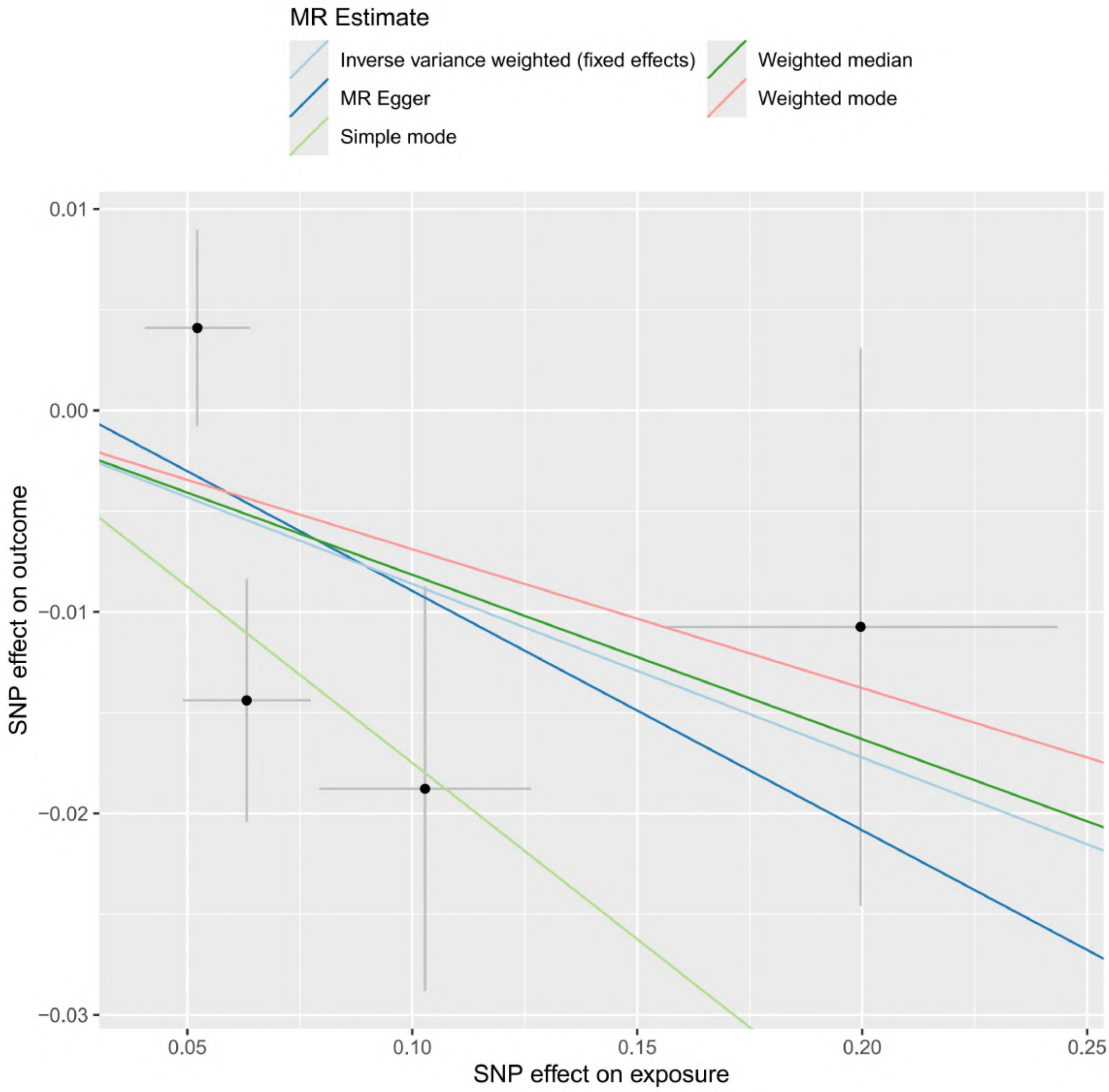

Supplementary Fig. 123 Scatter plot of the causal effect of genus.Ruminococcus2.id.11374 on GERD

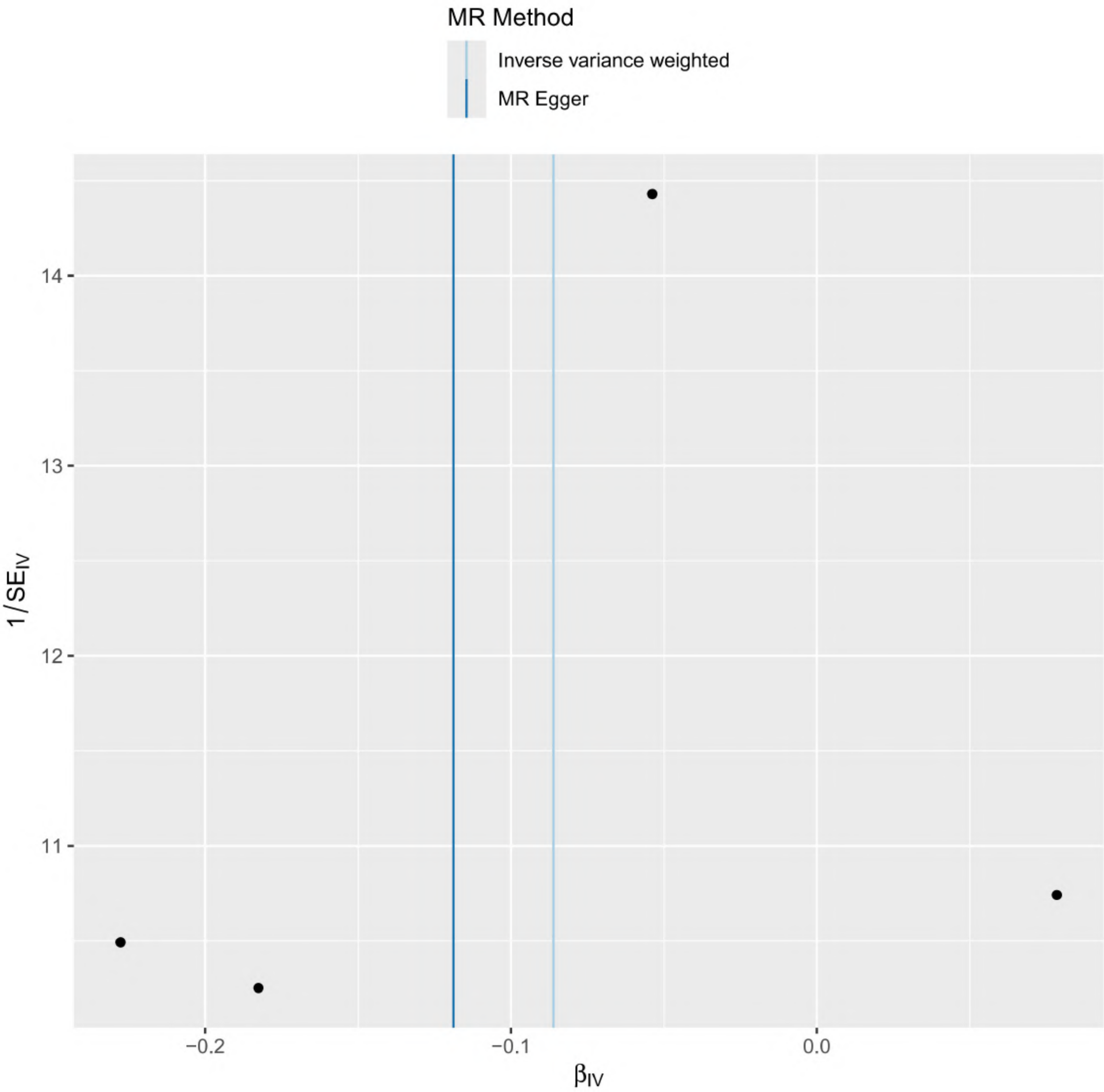

Supplementary Fig. 124 Funnel plot of the causal effect of genus.Ruminococcus2.id.11374 on GERD

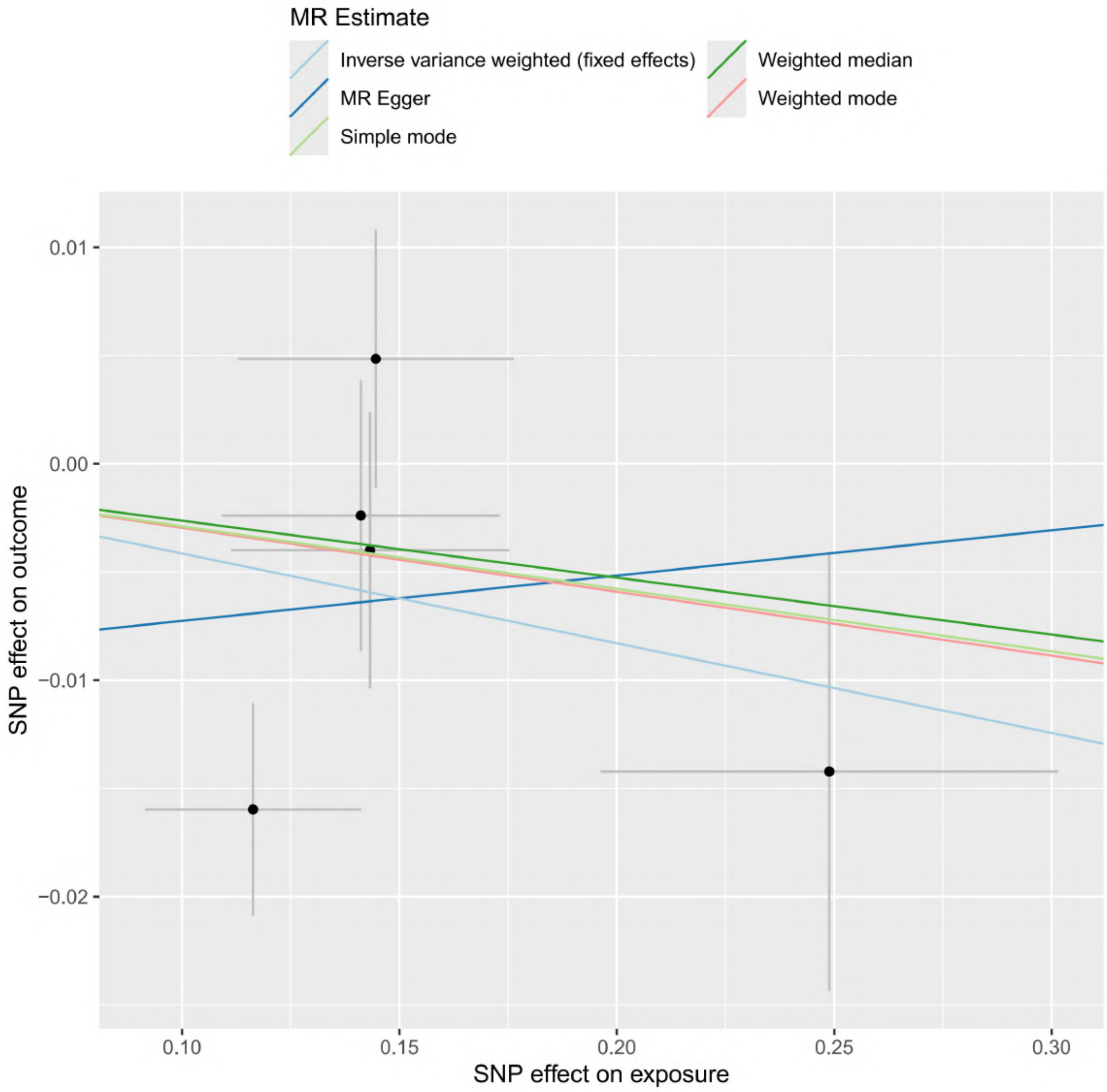

Supplementary Fig. 125 Scatter plot of the causal effect of order.Bacillales.id.1674 on GERD

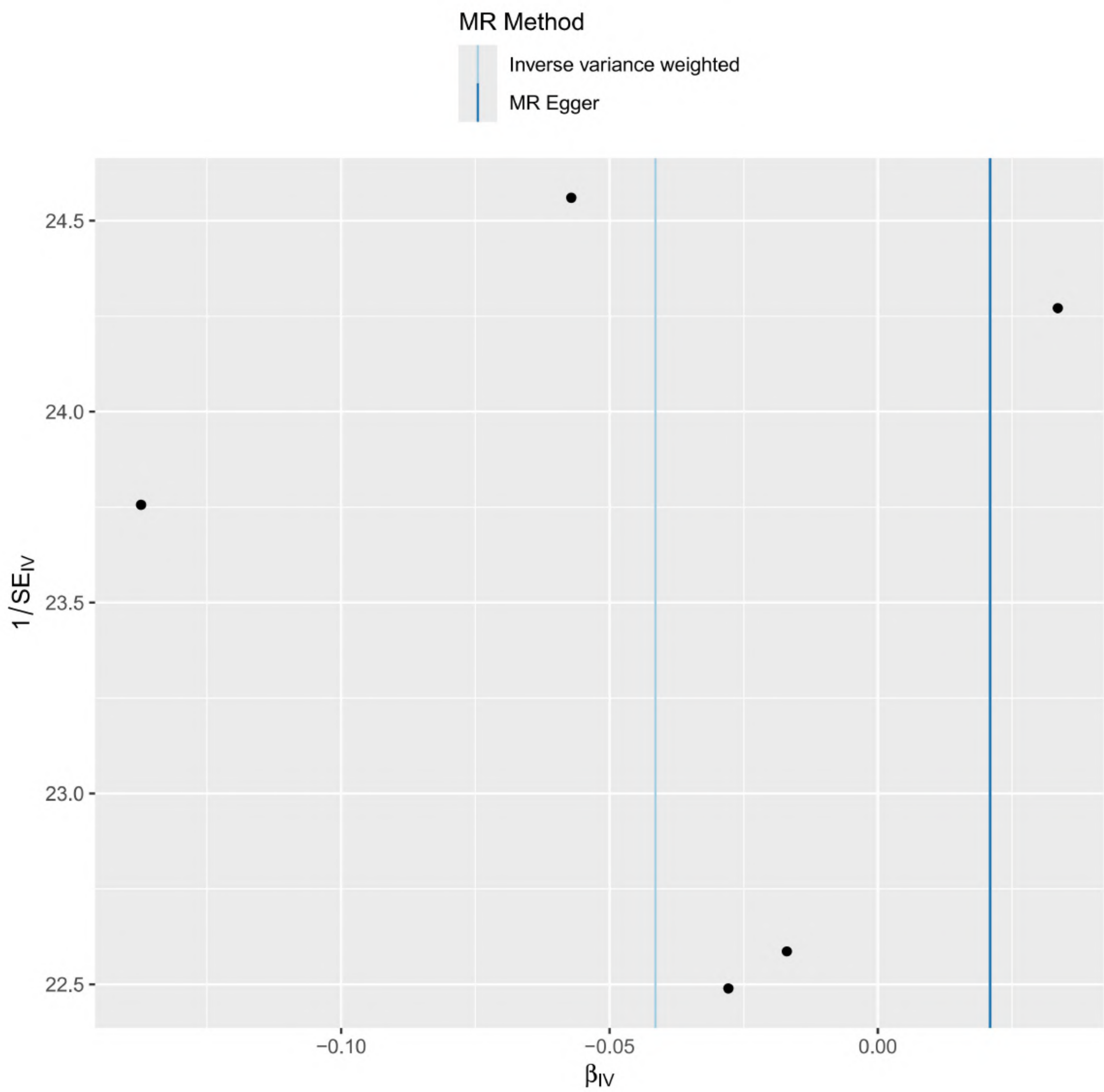

Supplementary Fig. 126 Funnel plot of the causal effect of order.Bacillales.id.1674 on GERD

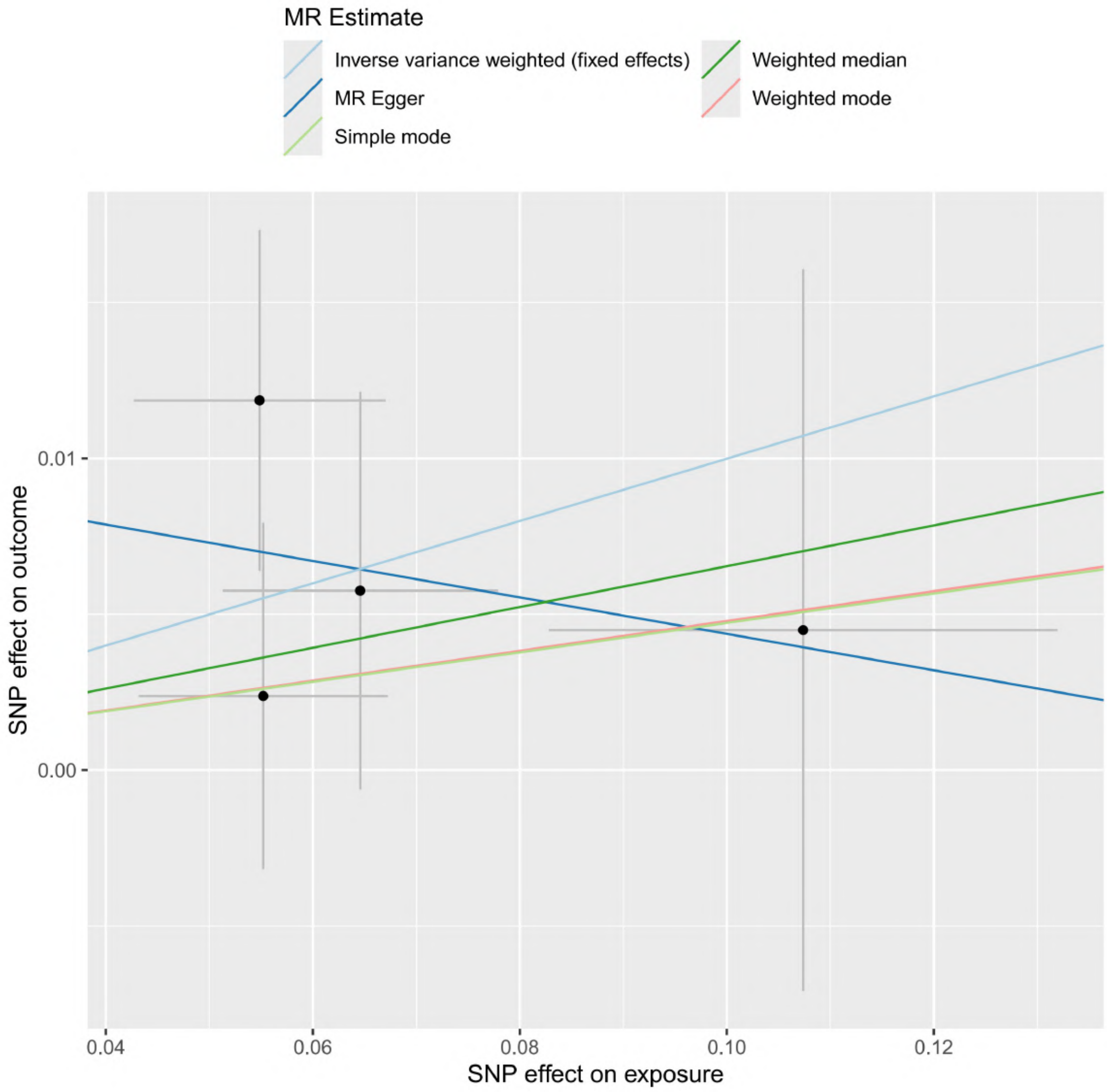

Supplementary Fig. 127 Scatter plot of the causal effect of order.Bacteroidales.id.913 on GERD

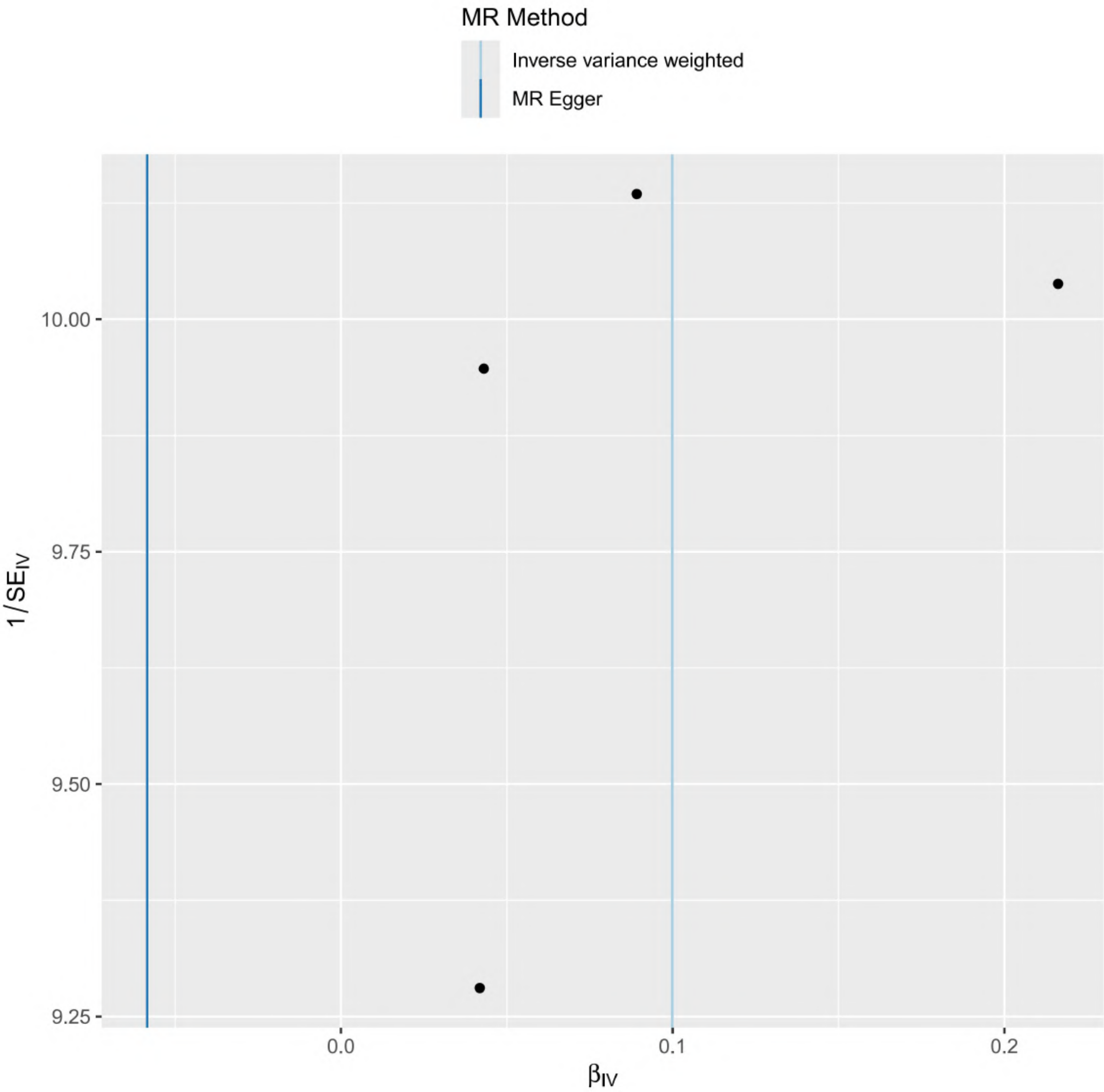

Supplementary Fig. 128 Funnel plot of the causal effect of order.Bacteroidales.id.913 on GERD

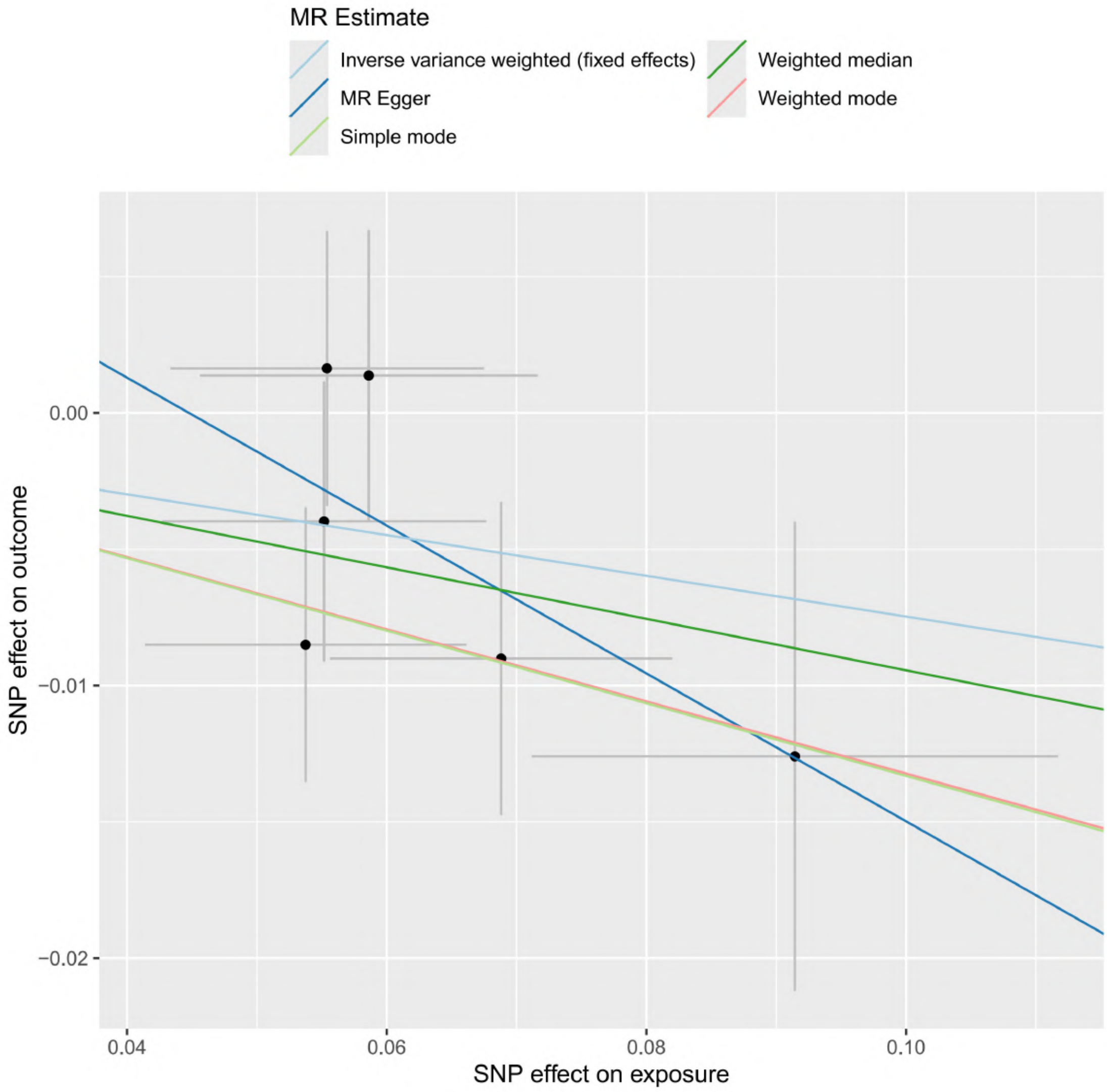

Supplementary Fig. 129 Scatter plot of the causal effect of order.Bifidobacteriales.id.432 on GERD

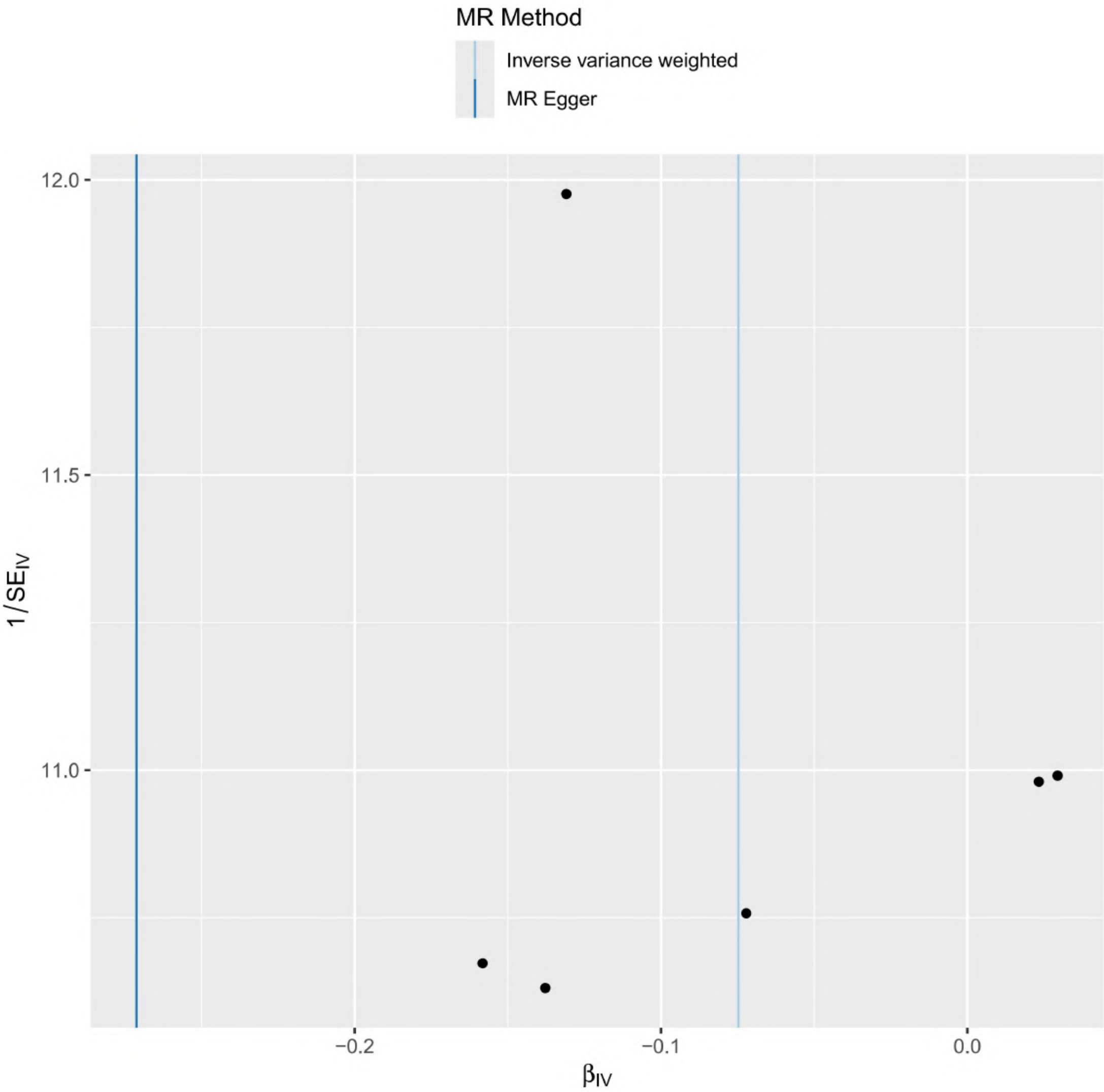

Supplementary Fig. 130 Funnel plot of the causal effect of order.Bifidobacteriales.id.432 on GERD

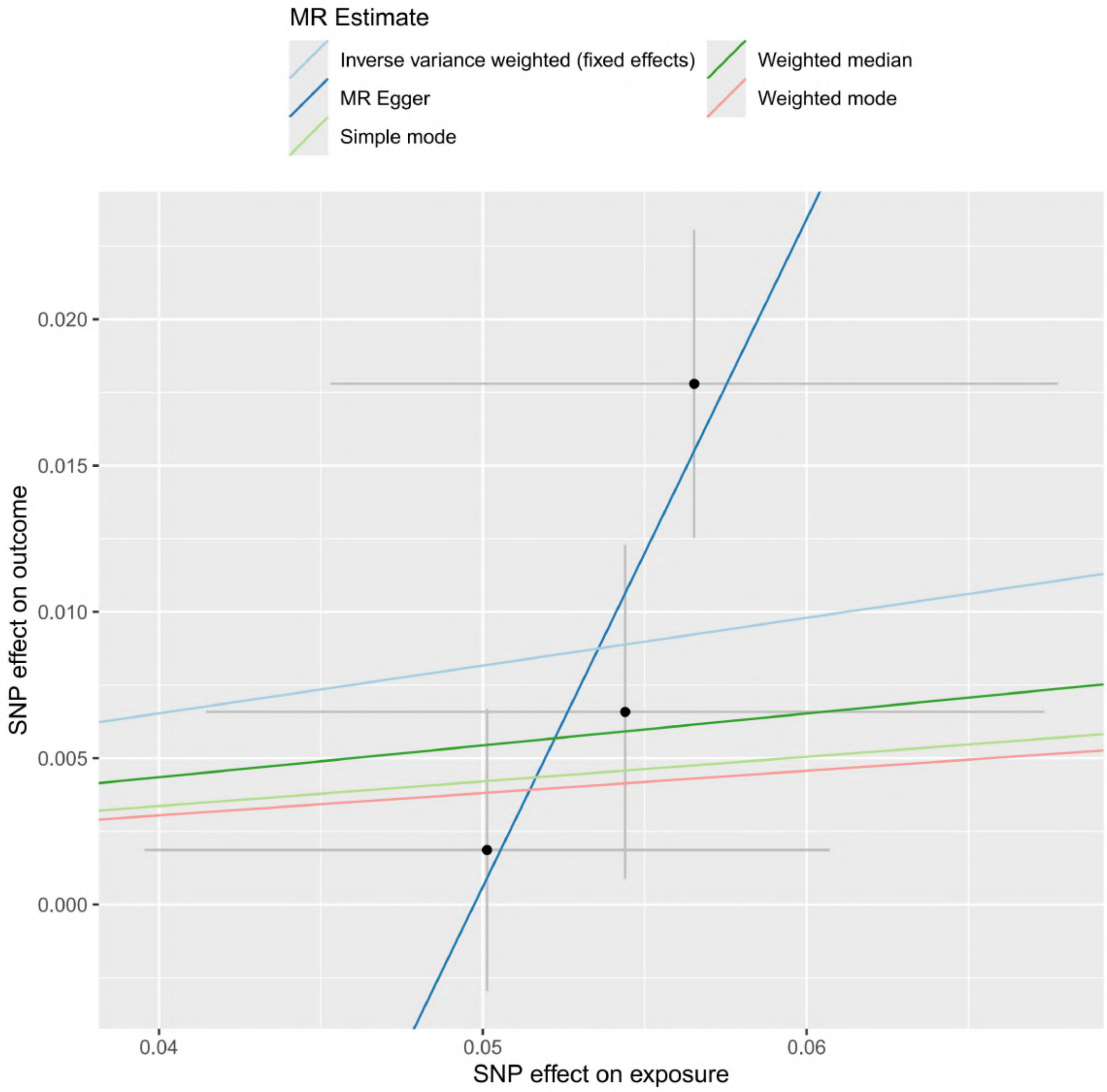

Supplementary Fig. 131 Scatter plot of the causal effect of order.Selenomonadales.id.2165 on GERD

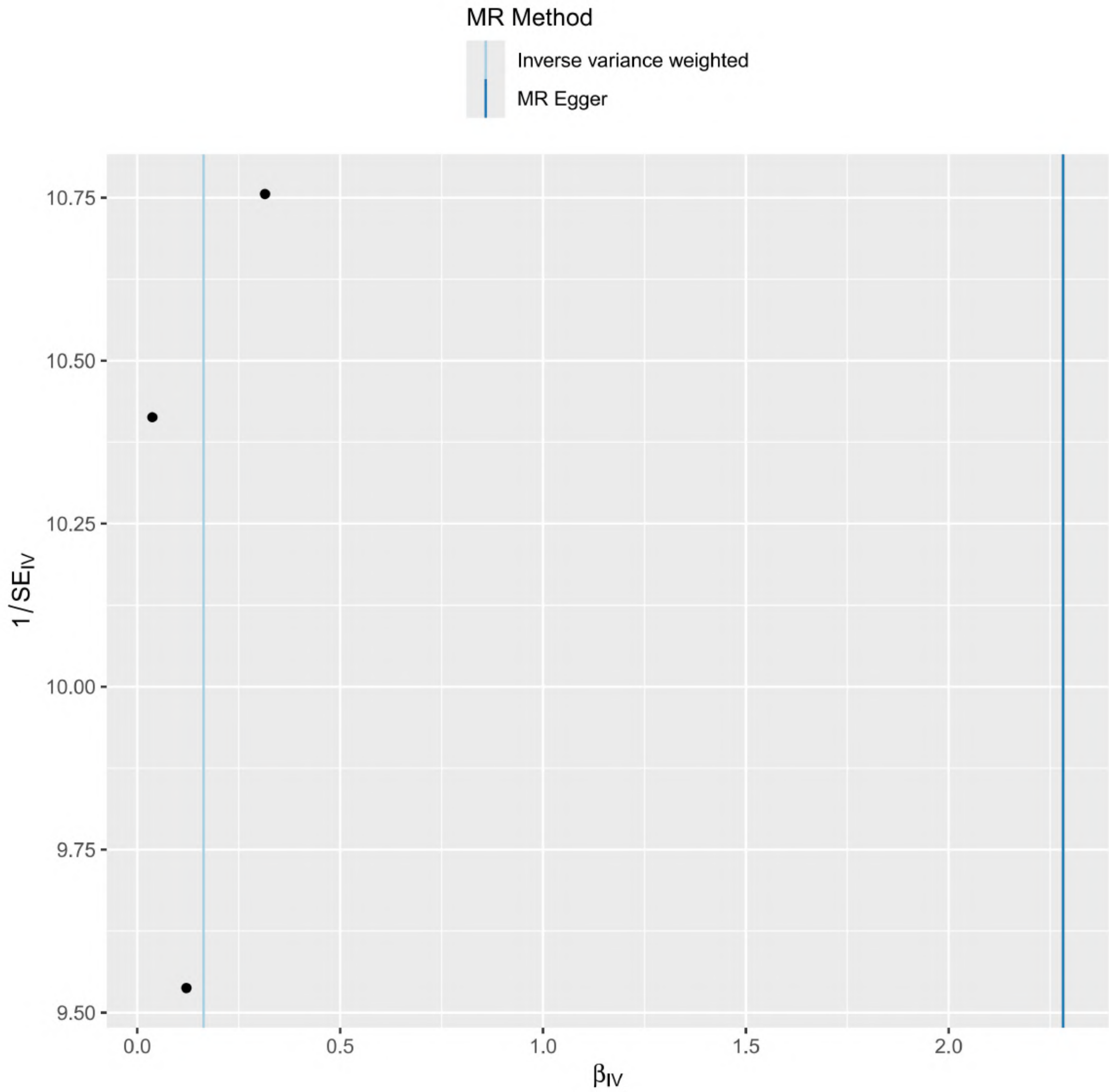

Supplementary Fig. 132 Funnel plot of the causal effect of order.Selenomonadales.id.2165 on GERD

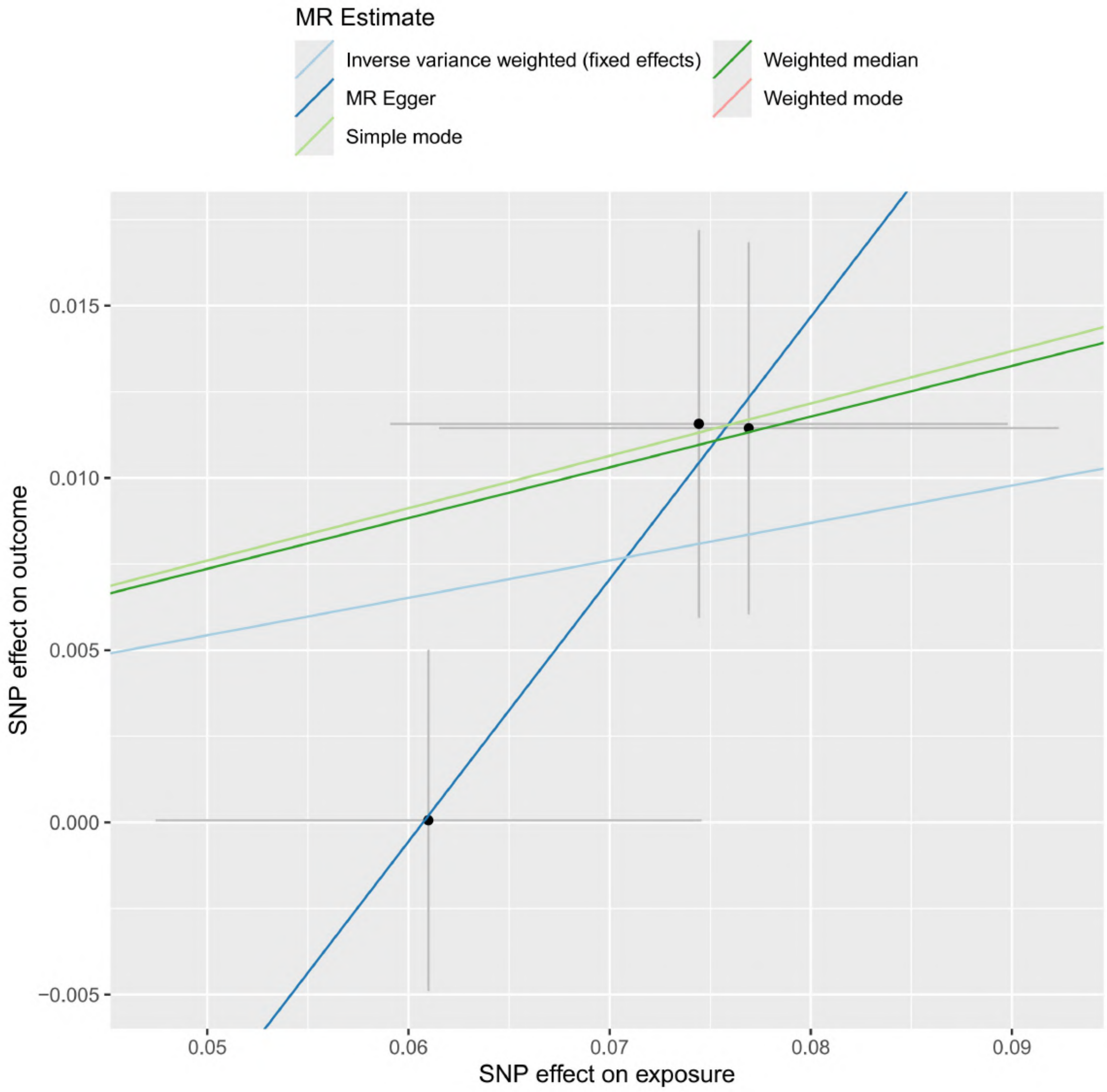

Supplementary Fig. 133 Scatter plot of the causal effect of phylum.Tenericutes.id.3919 on GERD

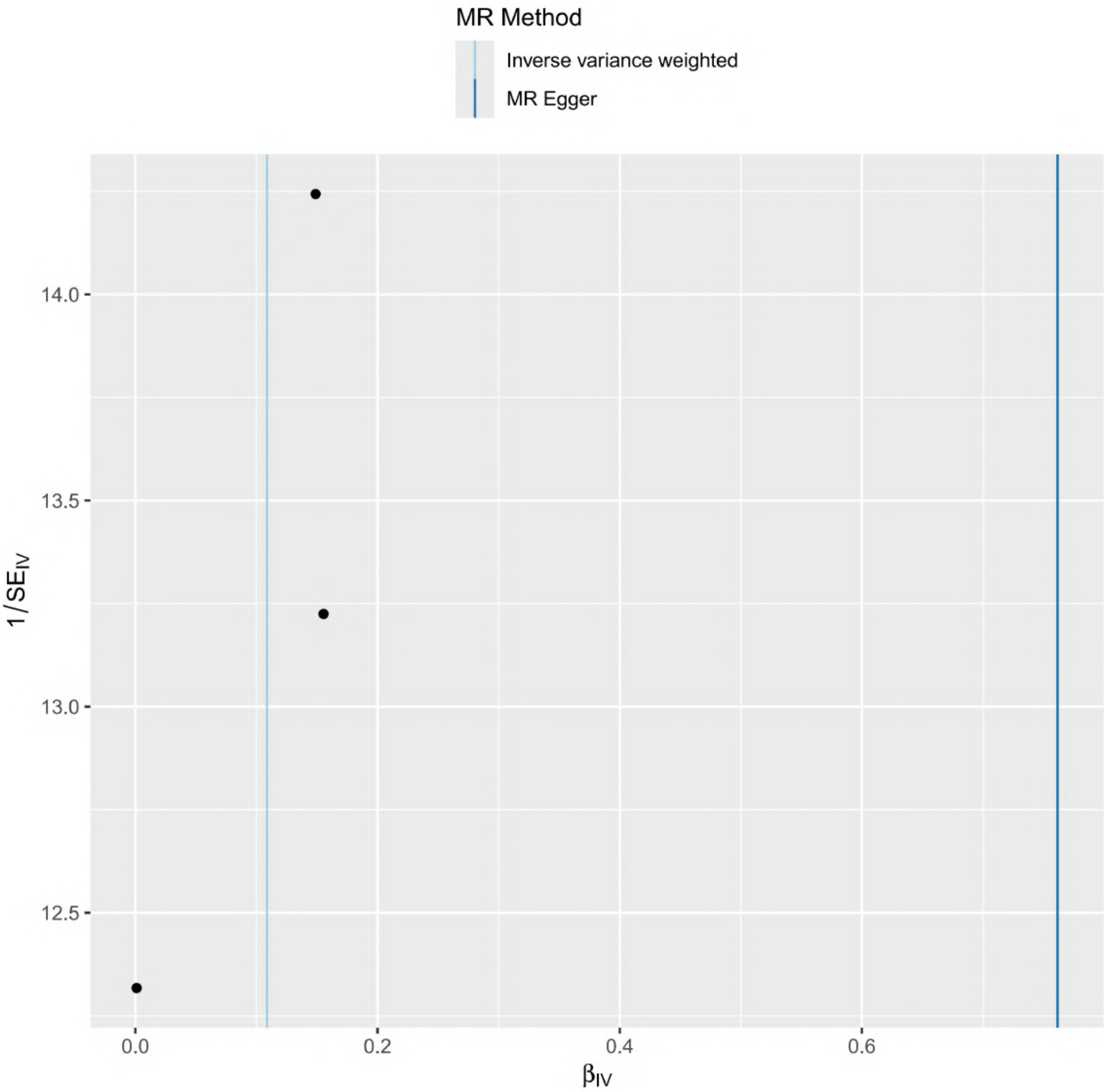

Supplementary Fig. 134 Funnel plot of the causal effect of phylum.Tenericutes.id.3919 on GERD

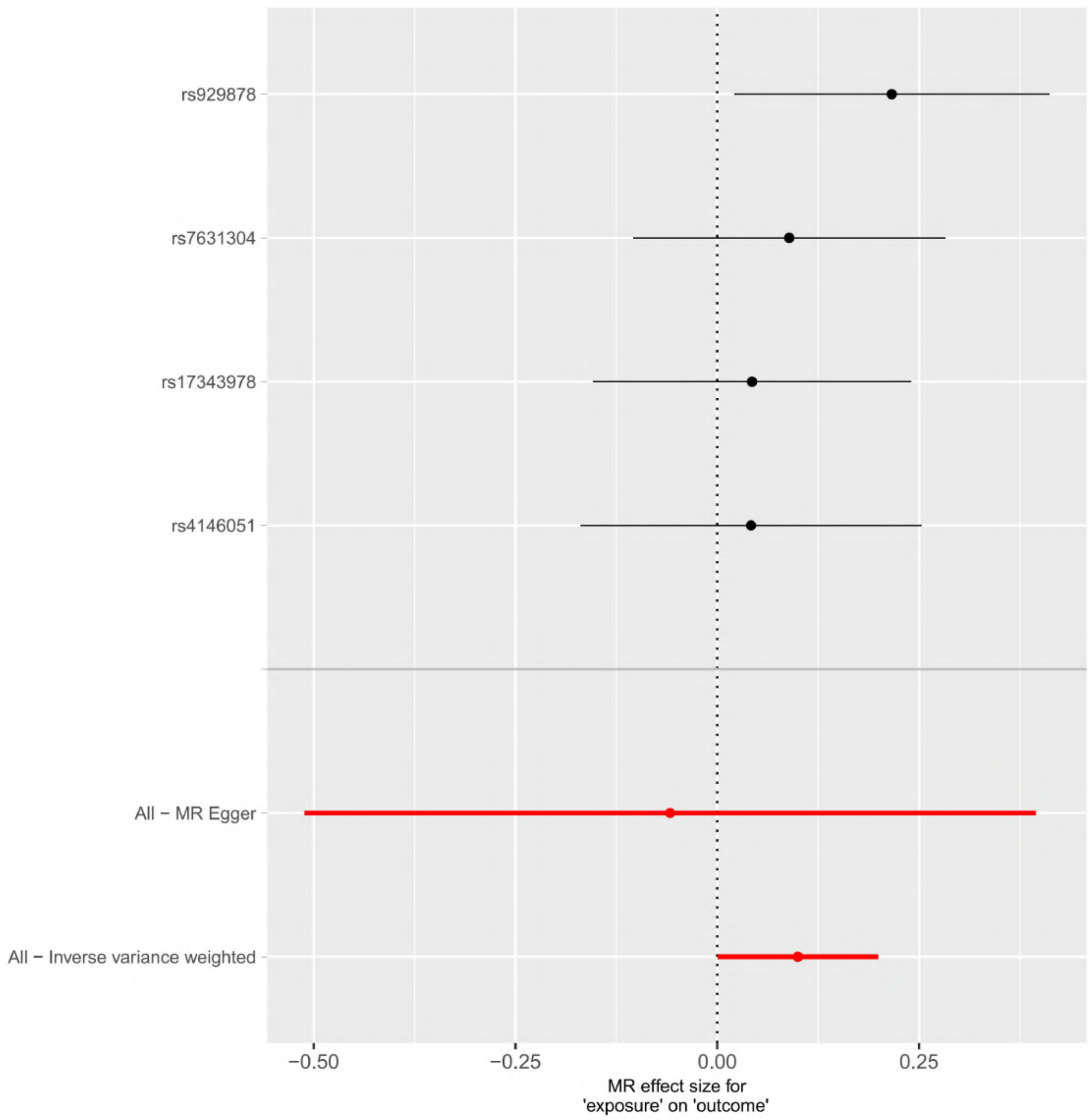

Supplementary Fig. 135 Forest plot of the causal effect of class.Bacteroidia.id.912 on GERD

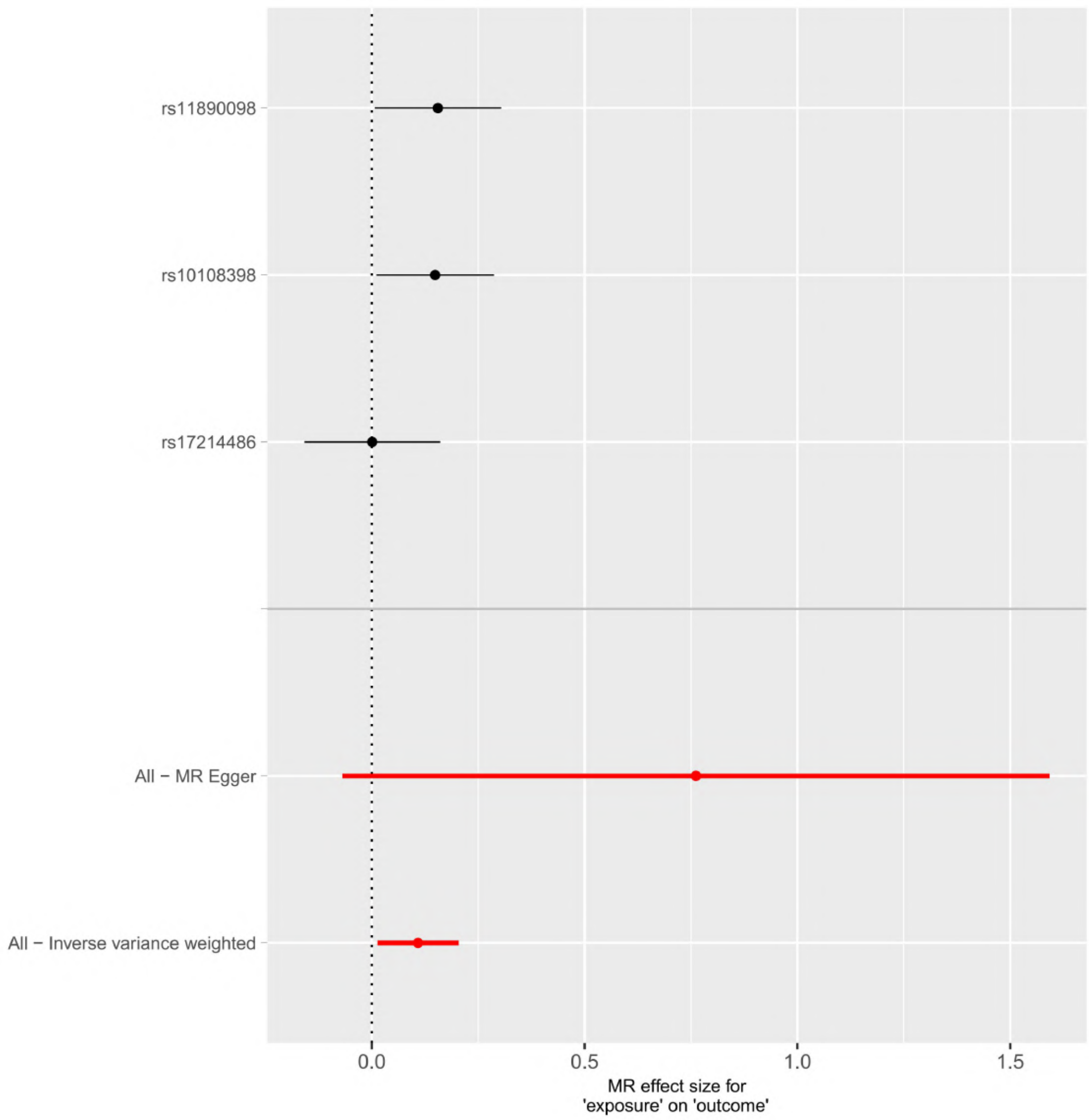

Supplementary Fig. 136 Forest plot of the causal effect of class.Mollicutes.id.3920 on GERD

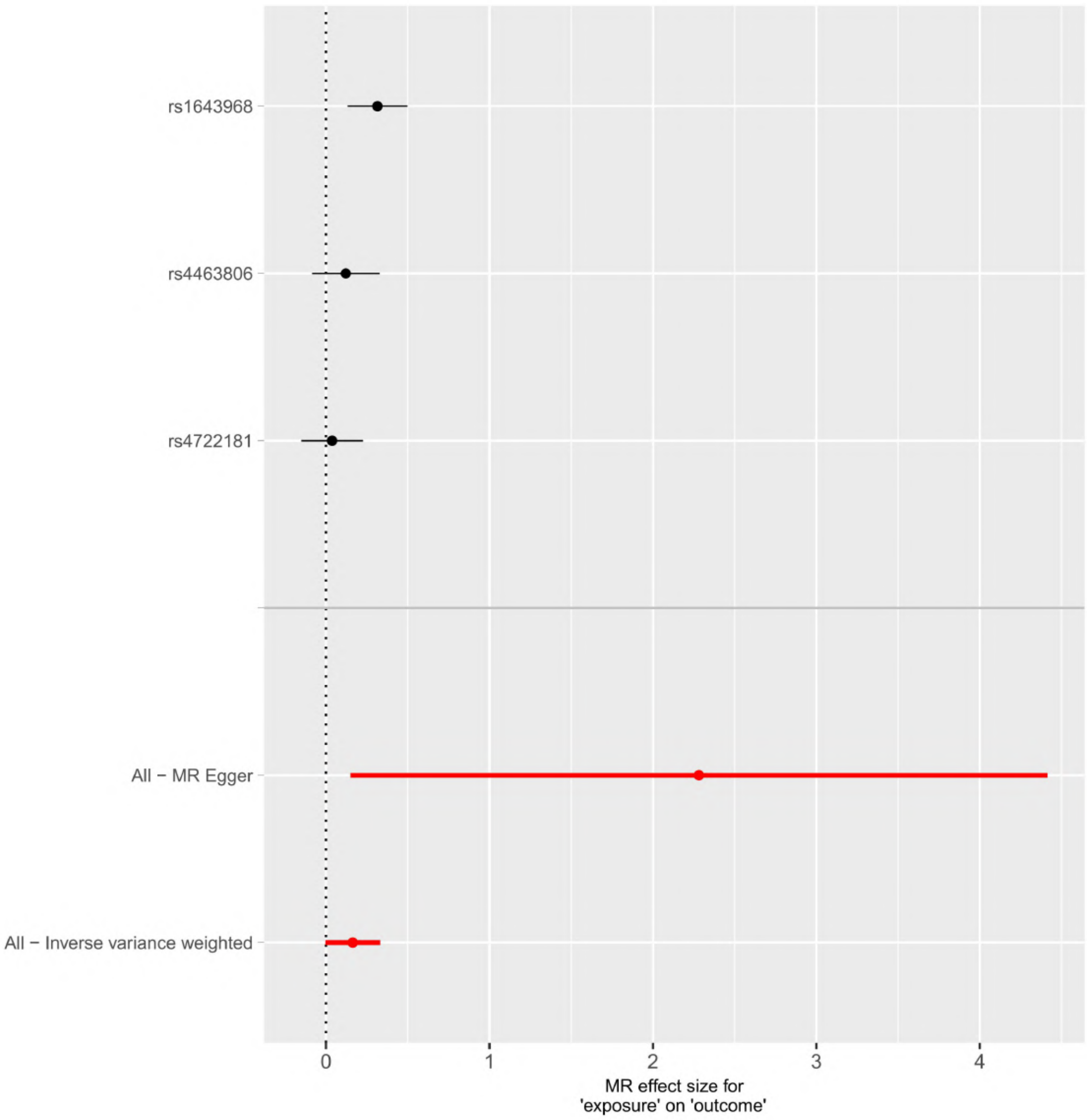

Supplementary Fig. 137 Forest plot of the causal effect of class.Negativicutes.id.2164 on GERD

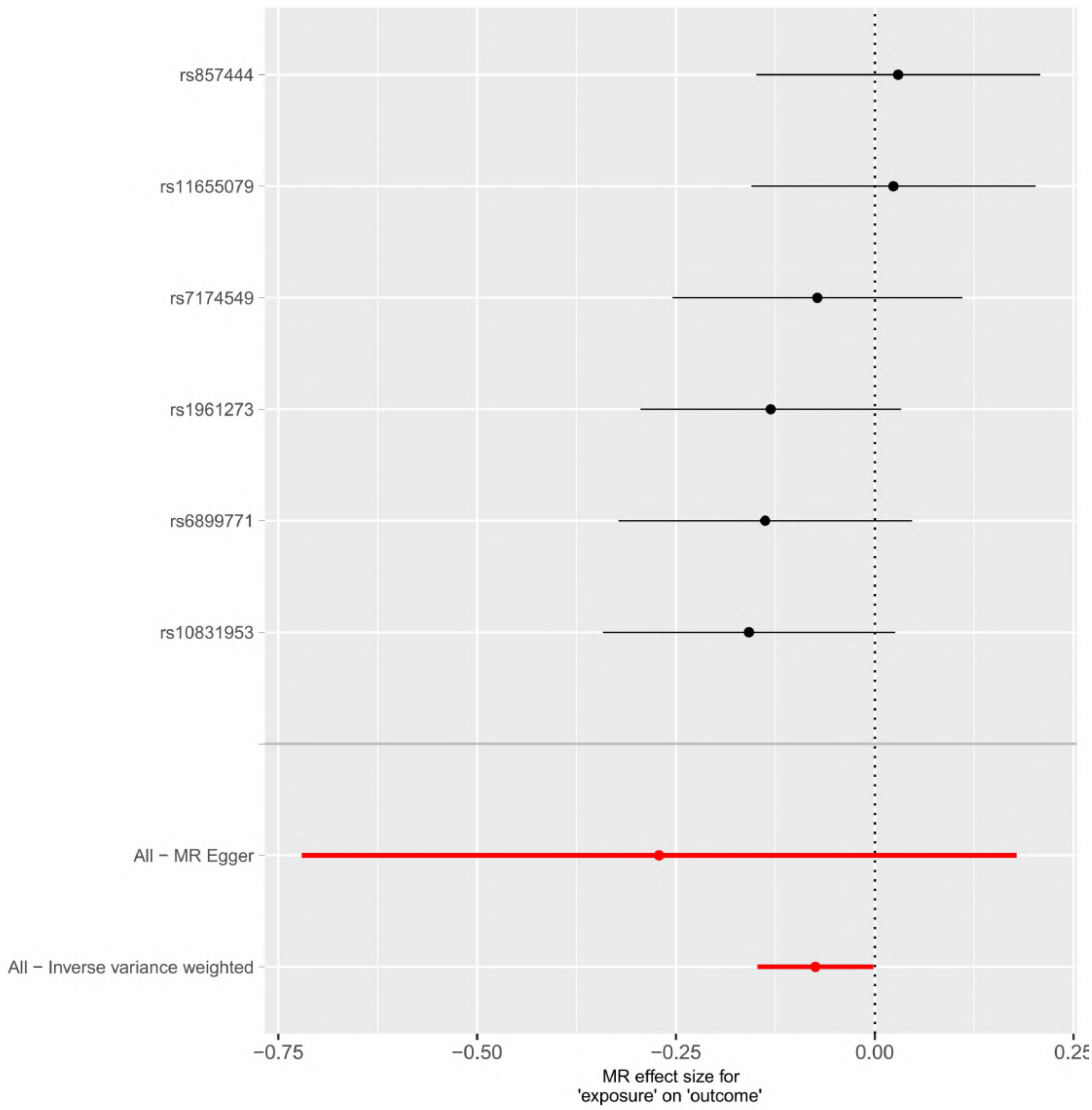

Supplementary Fig. 138 Forest plot of the causal effect of family.Bifidobacteriaceae.id.433 on GERD

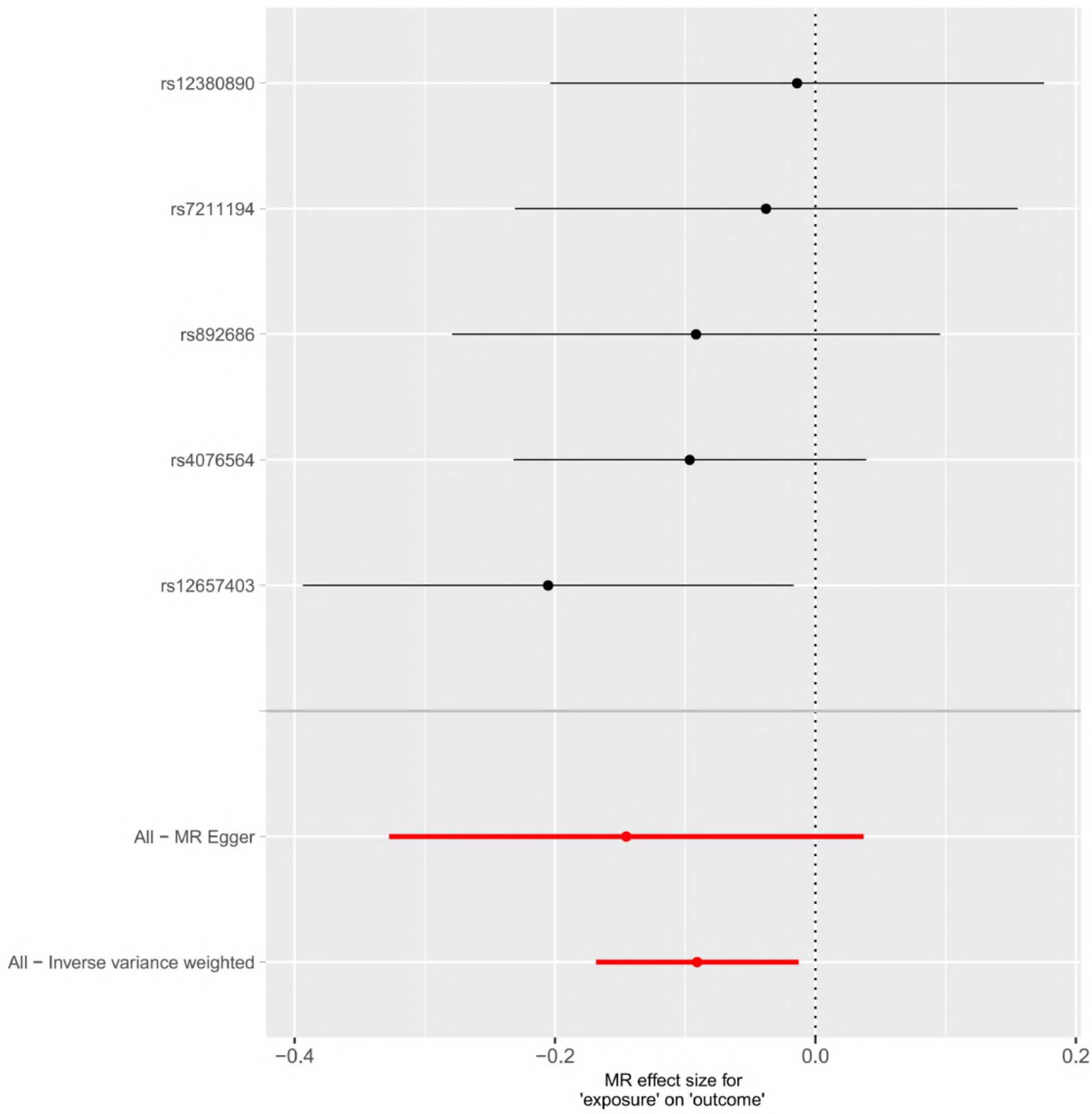

Supplementary Fig. 139 Forest plot of the causal effect of family.Christensenellaceae.id.1866 on GERD

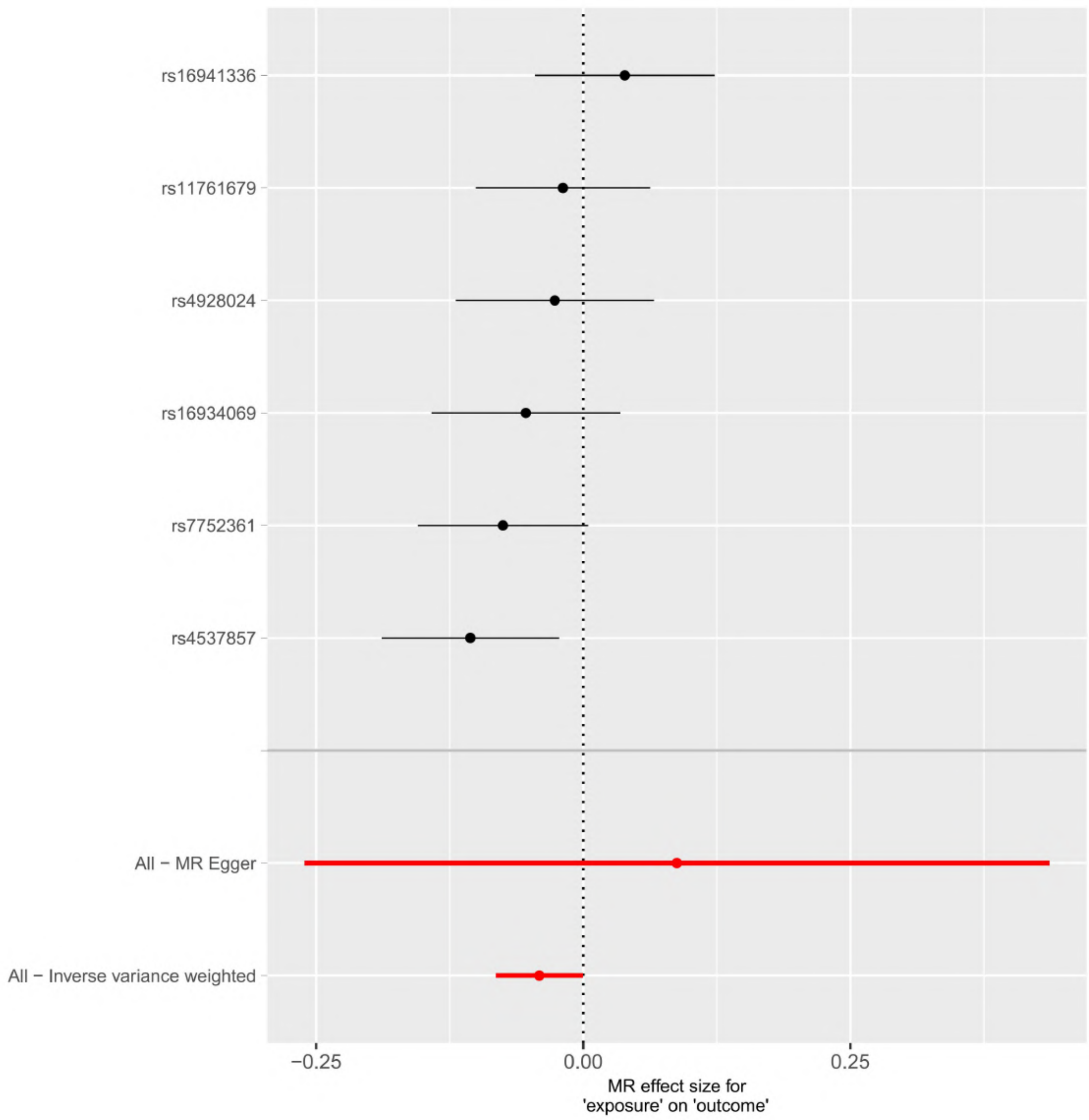

Supplementary Fig. 140 Forest plot of the causal effect of genus.Butyrivibrio.id.1993 on GERD

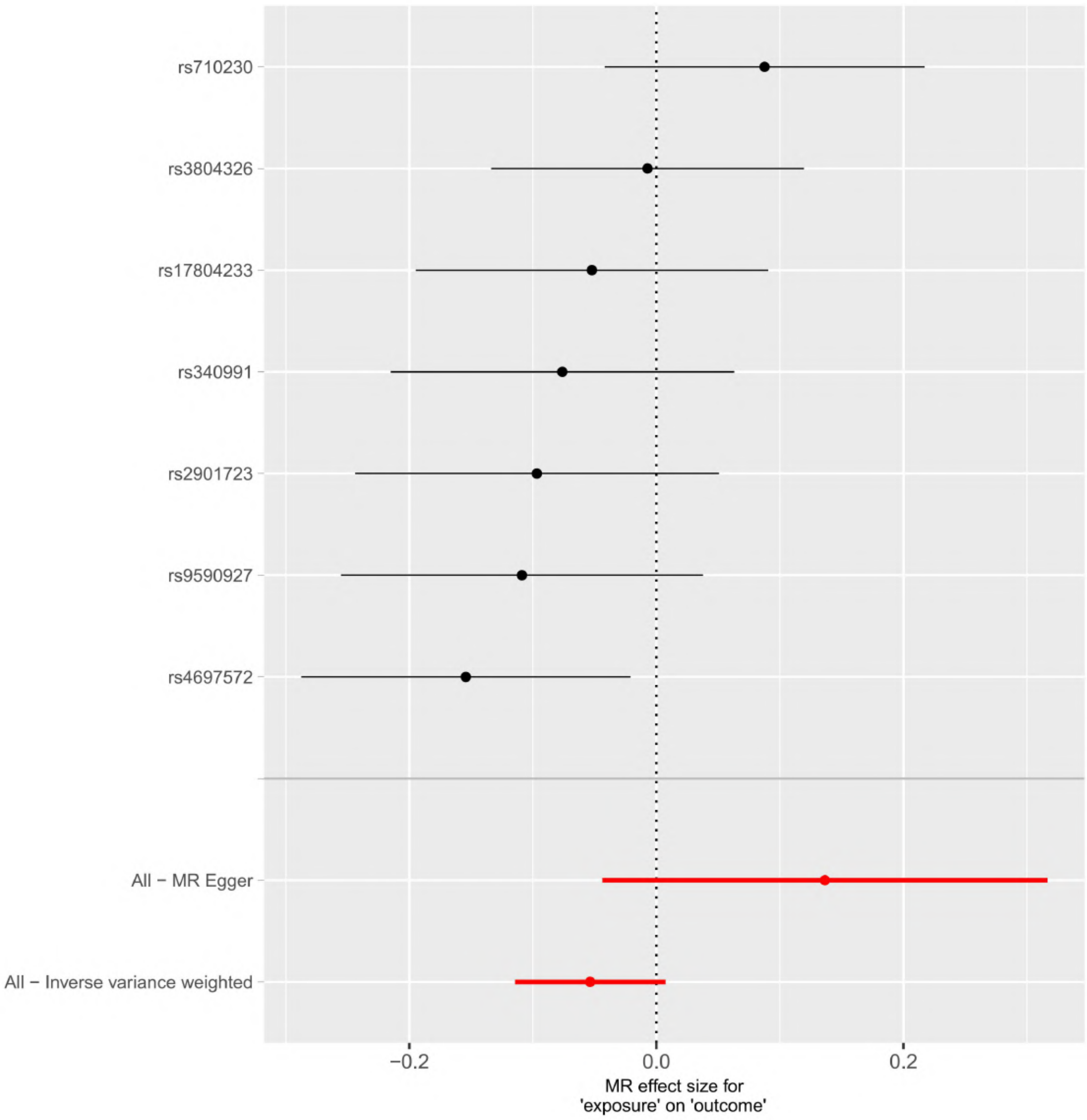

Supplementary Fig. 141 Forest plot of the causal effect of genus.Erysipelatoclostridium.id.11381 on GERD

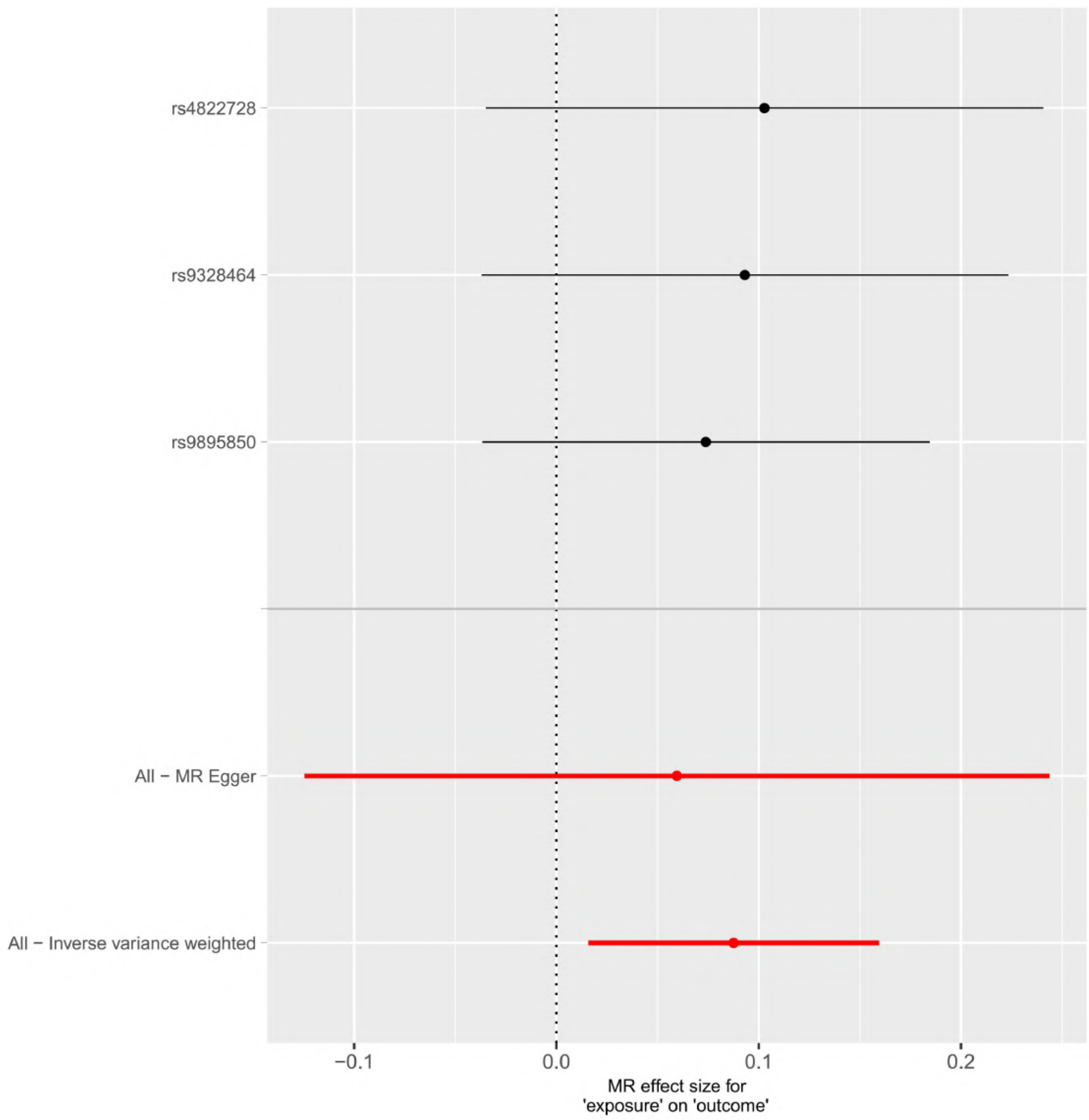

Supplementary Fig. 142 Forest plot of the causal effect of genus.Haemophilus.id.3698 on GERD

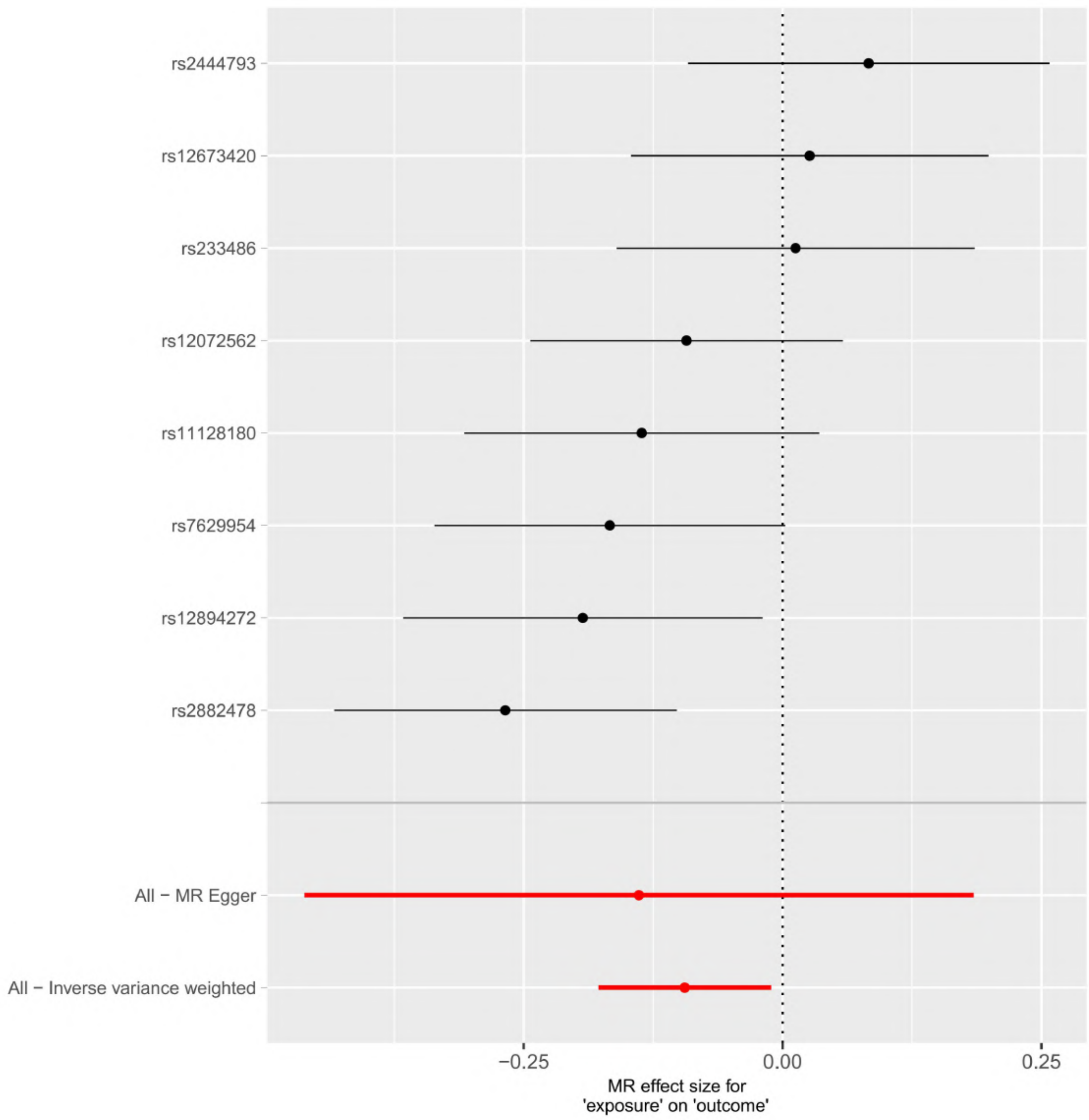

Supplementary Fig. 143 Forest plot of the causal effect of genus.LachnospiraceaeUCG004.id.11324 on GERD

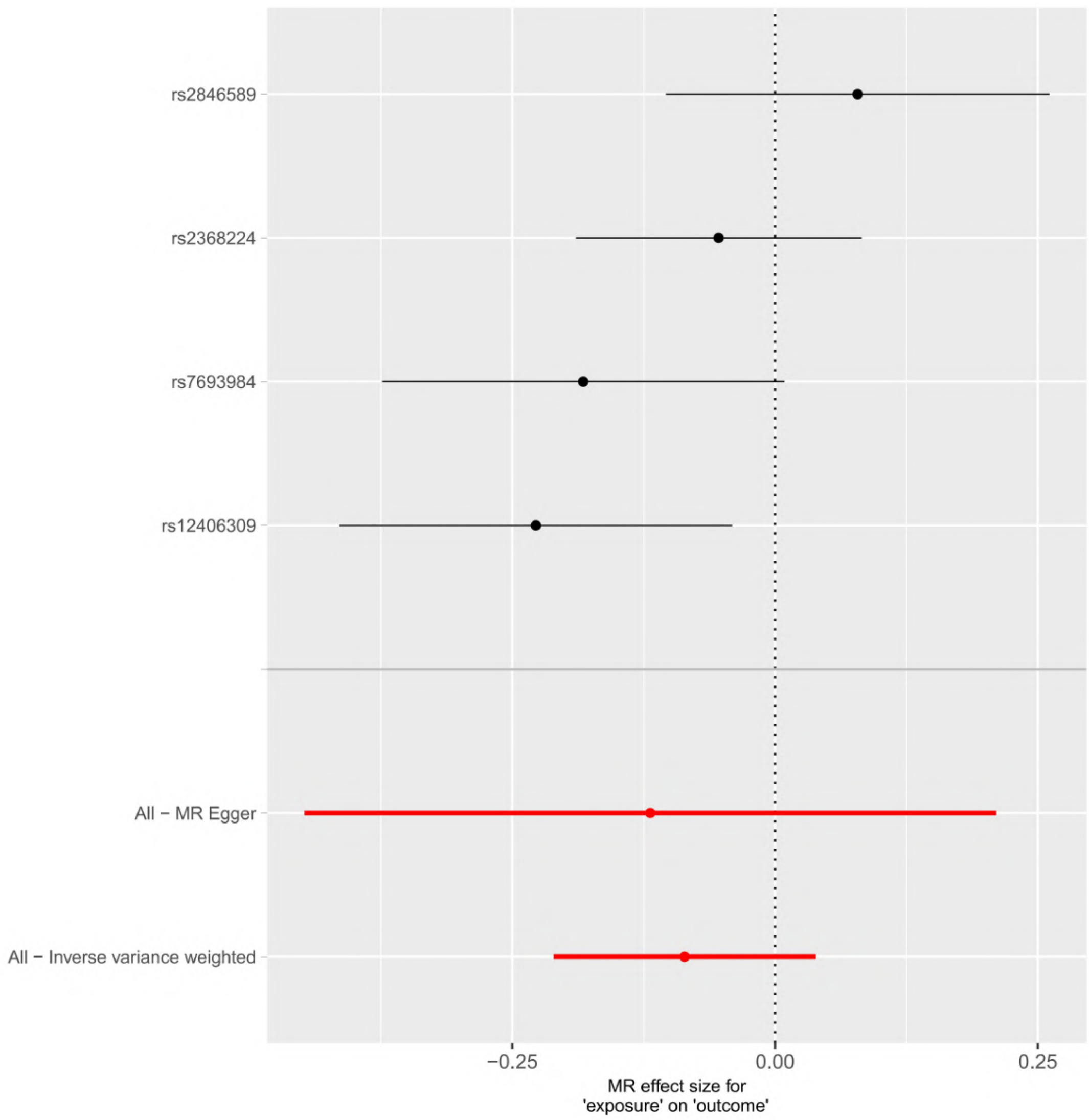

Supplementary Fig. 144 Forest plot of the causal effect of genus.Ruminococcus2.id.11374 on GERD

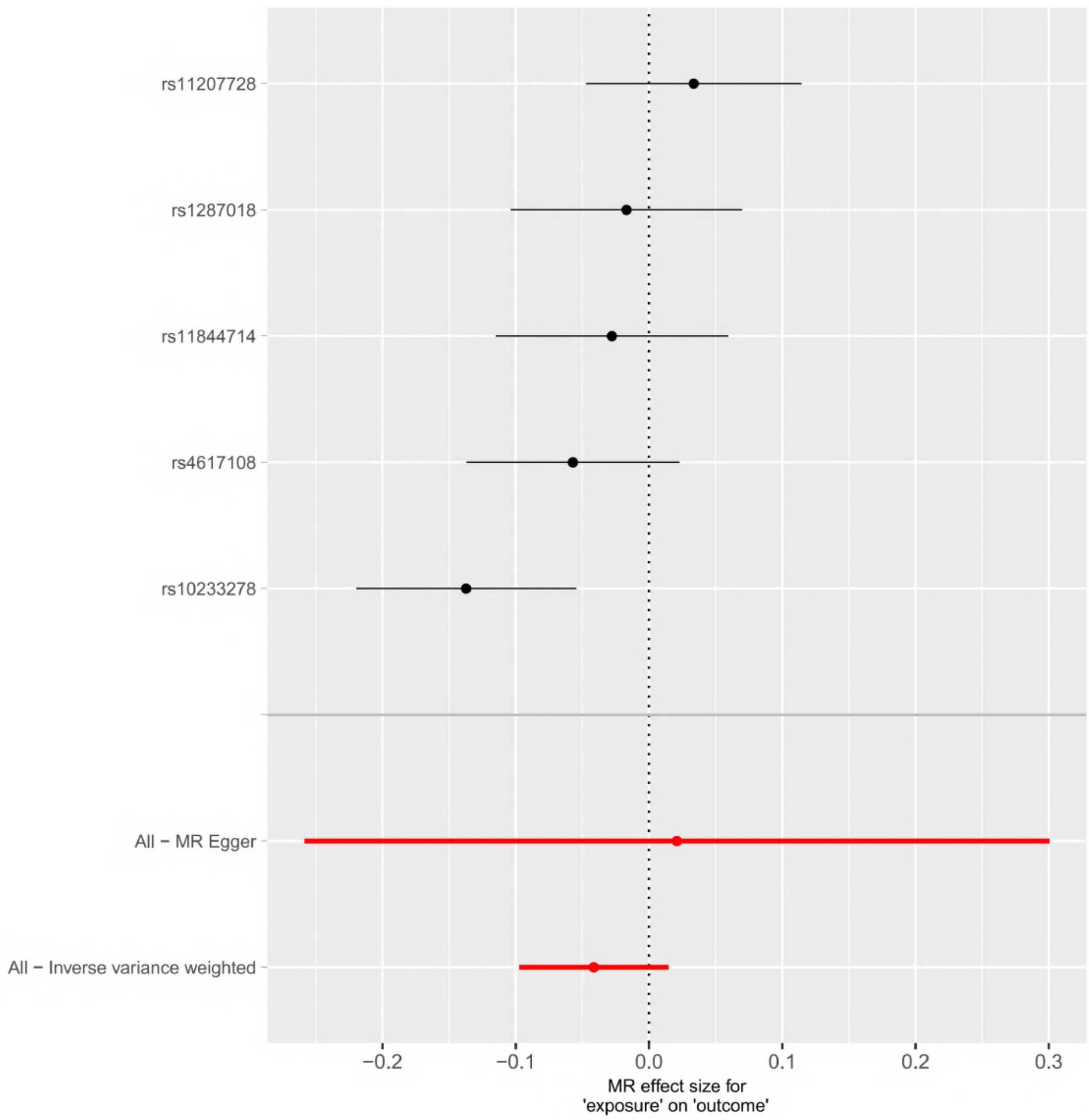

Supplementary Fig. 145 Forest plot of the causal effect of order.Bacillales.id.1674 on GERD

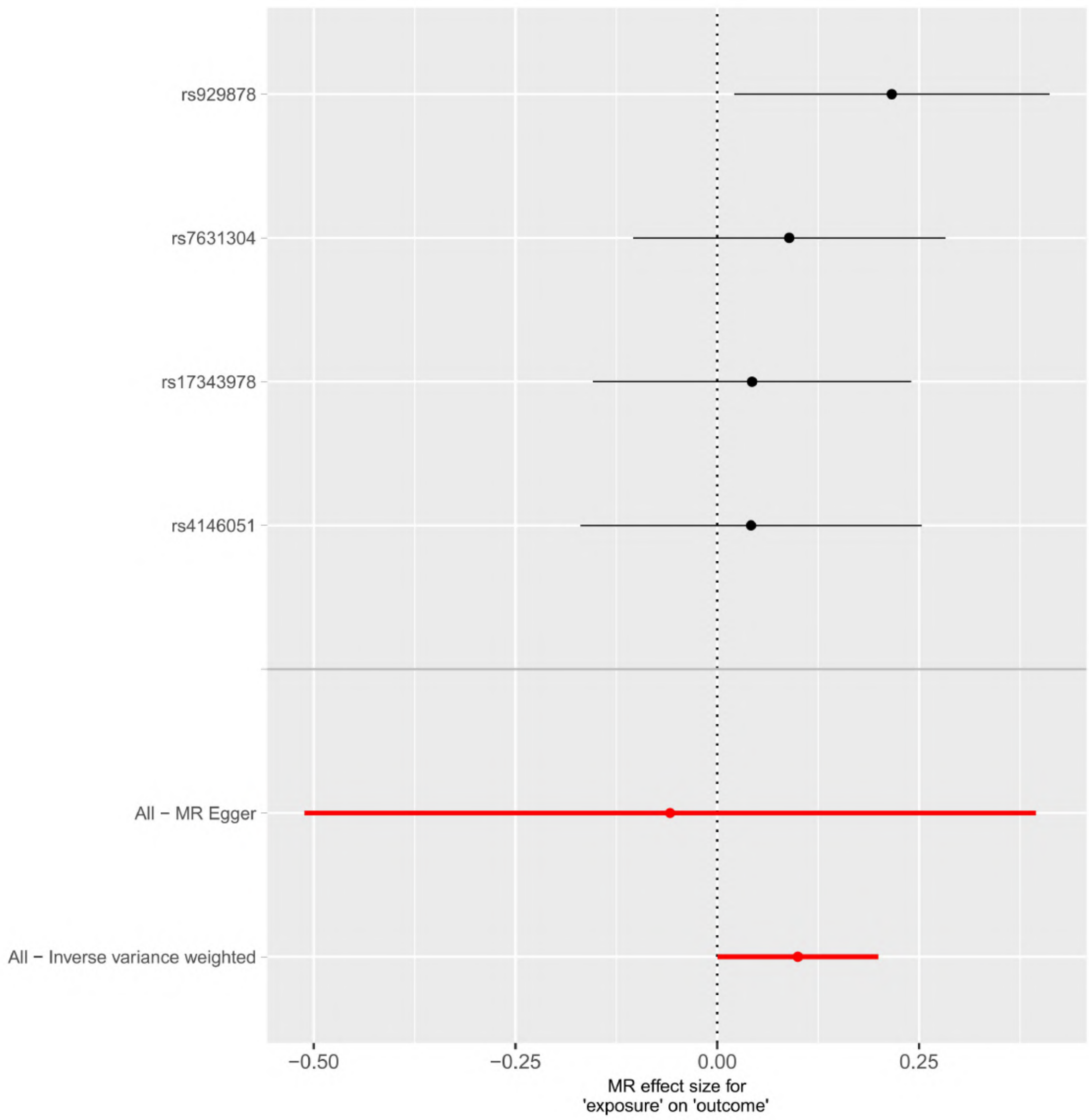

Supplementary Fig. 146 Forest plot of the causal effect of order.Bacteroidales.id.913 on GERD

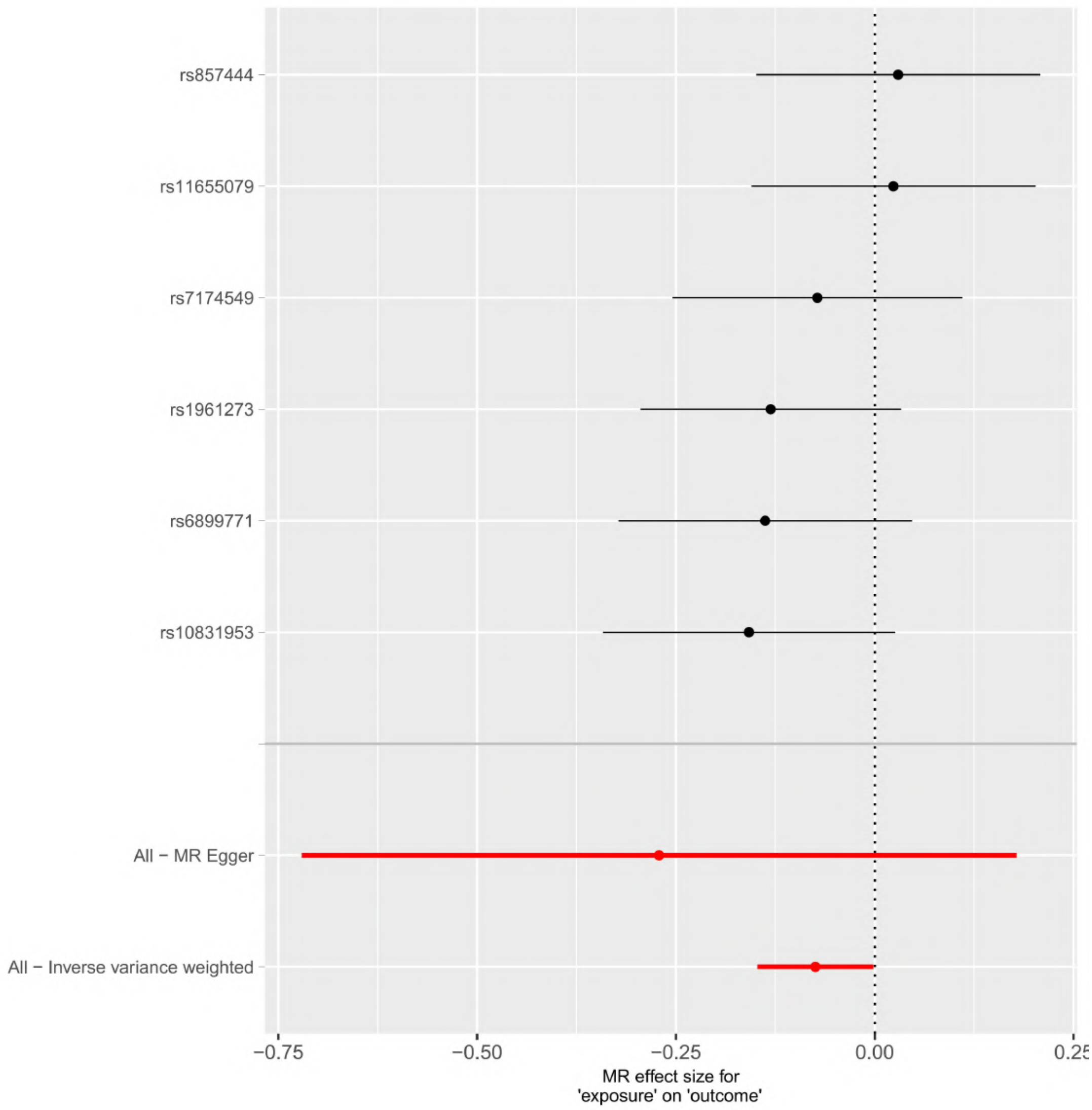

Supplementary Fig. 147 Forest plot of the causal effect of order.Bifidobacteriales.id.432 on GERD

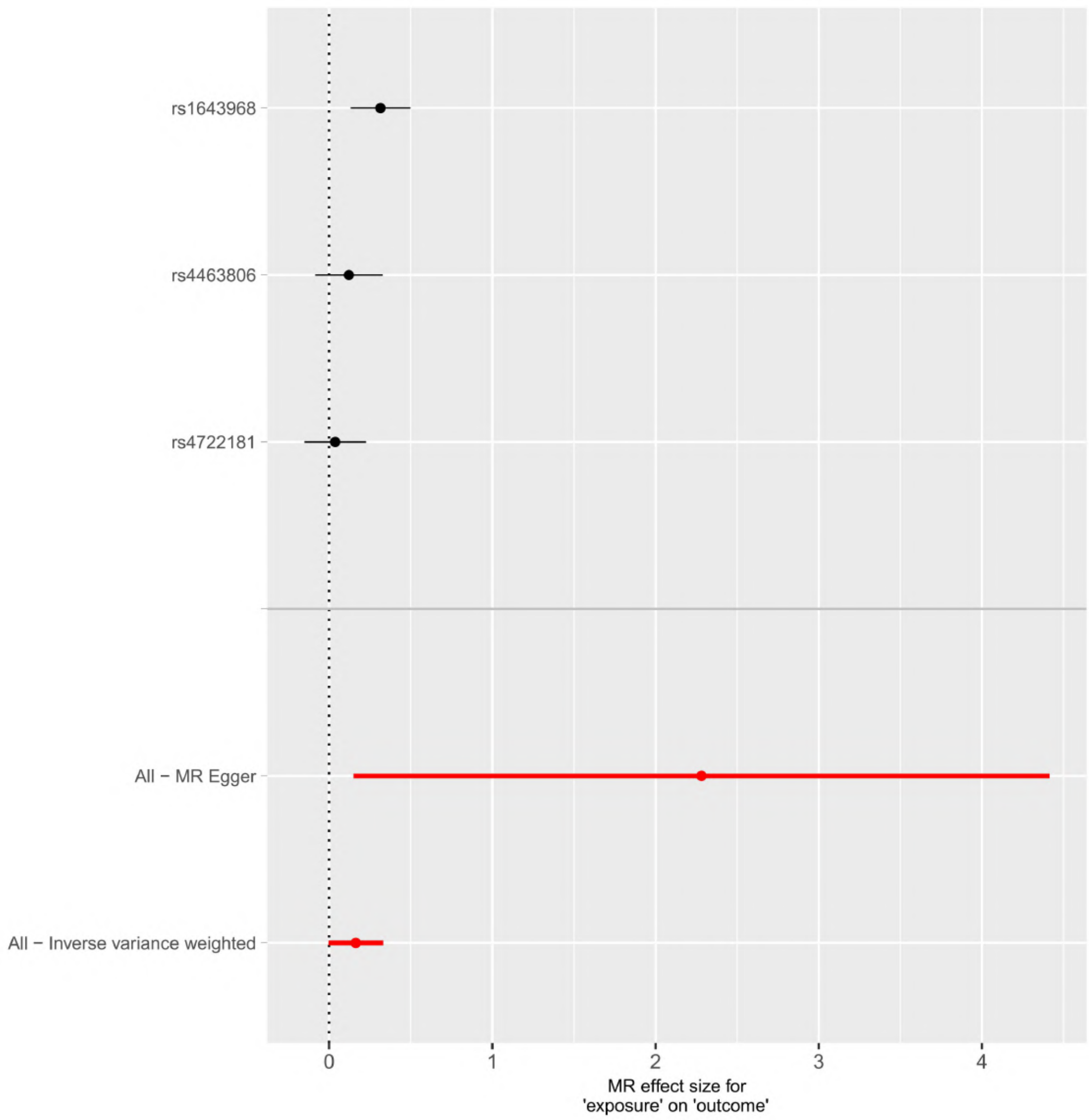

Supplementary Fig. 148 Forest plot of the causal effect of order.Selenomonadales.id.2165 on GERD

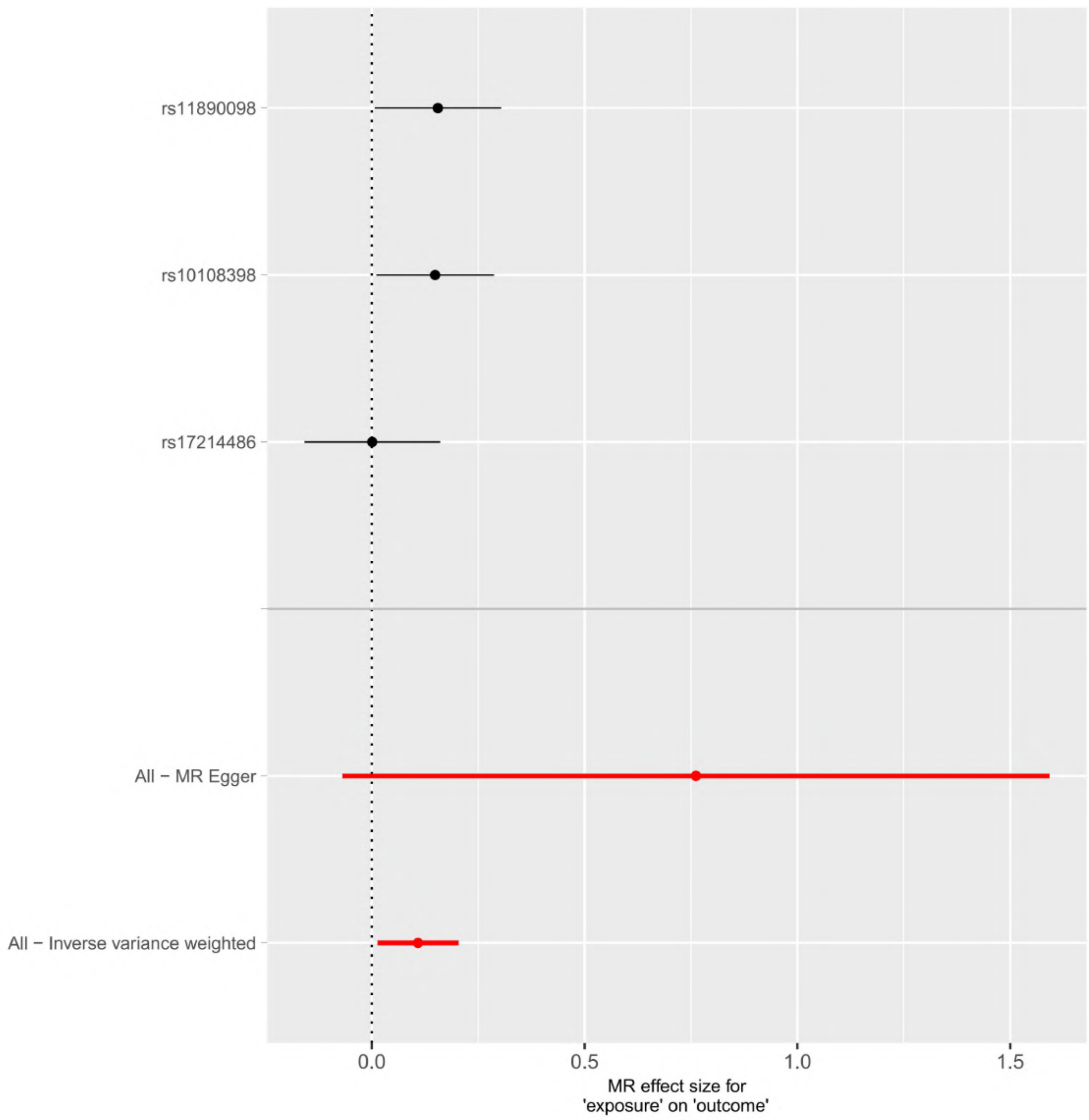

Supplementary Fig. 149 Forest plot of the causal effect of phylum.Tenericutes.id.3919 on GERD

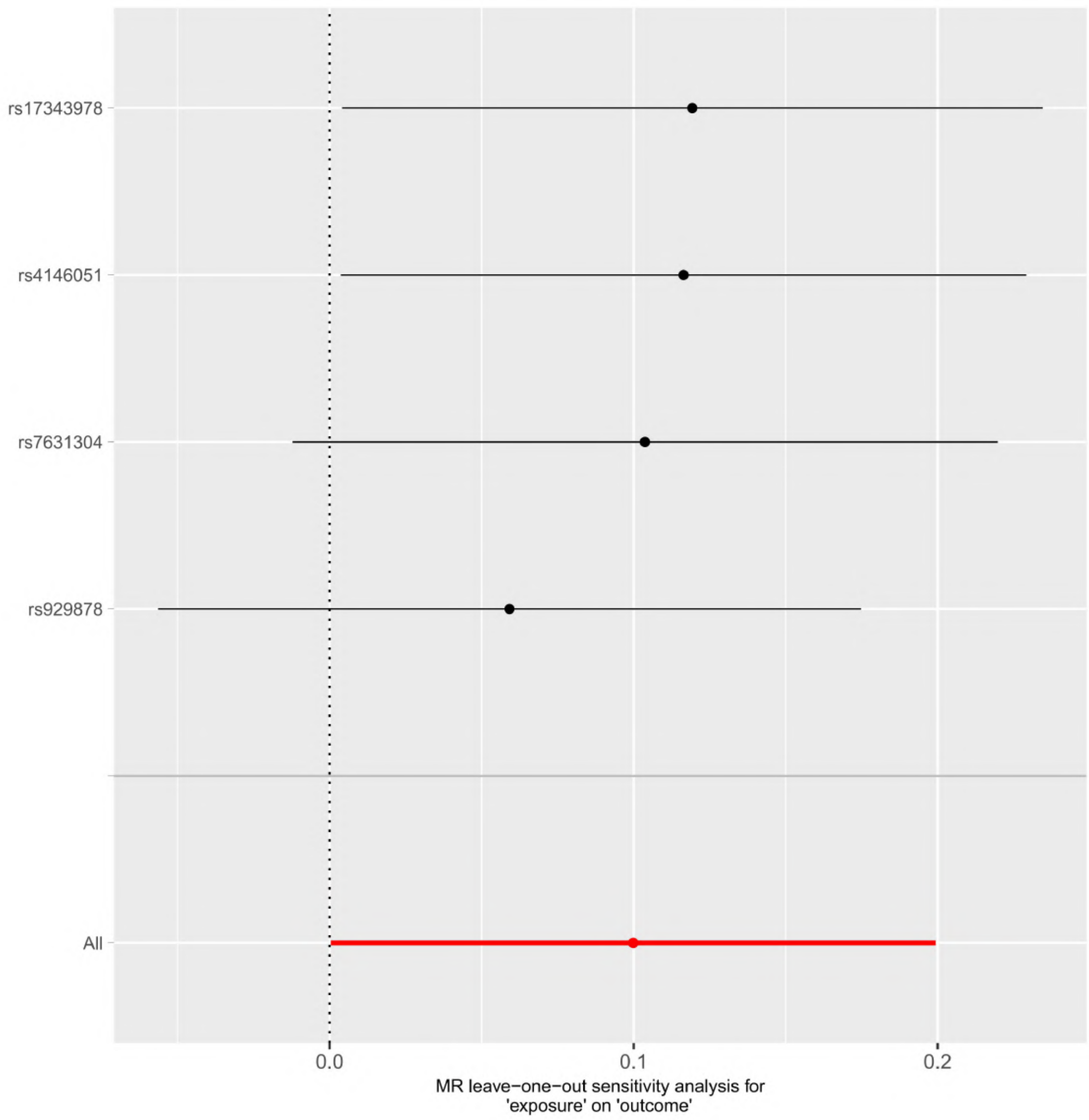

Supplementary Fig. 150 Leave-one-out inverse-variance weighted mendelian randomization analysis of class.Bacteroidia.id.912 on GERD

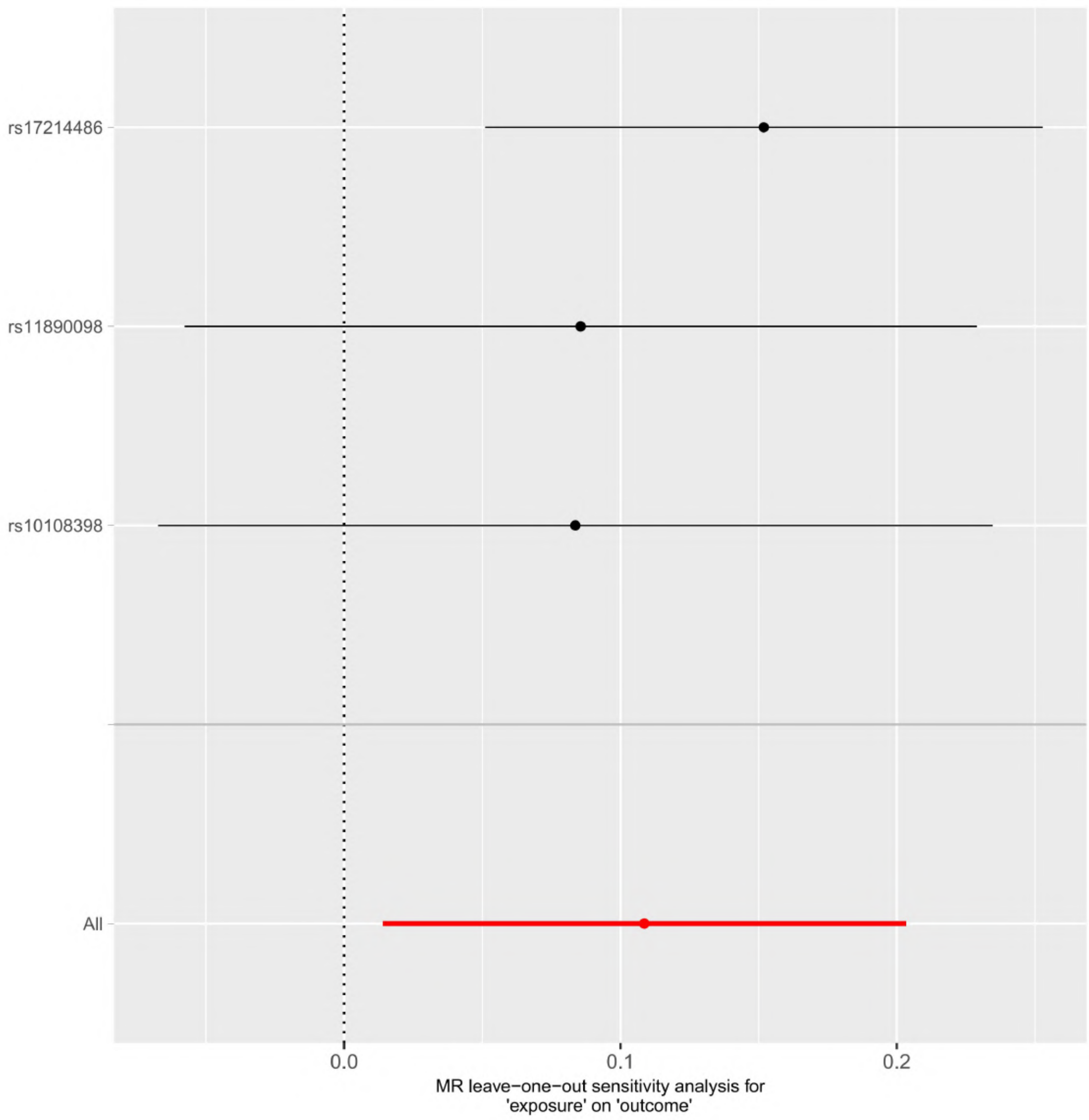

Supplementary Fig. 151 Leave-one-out inverse-variance weighted mendelian randomization analysis of class.Mollicutes.id.3920 on GERD

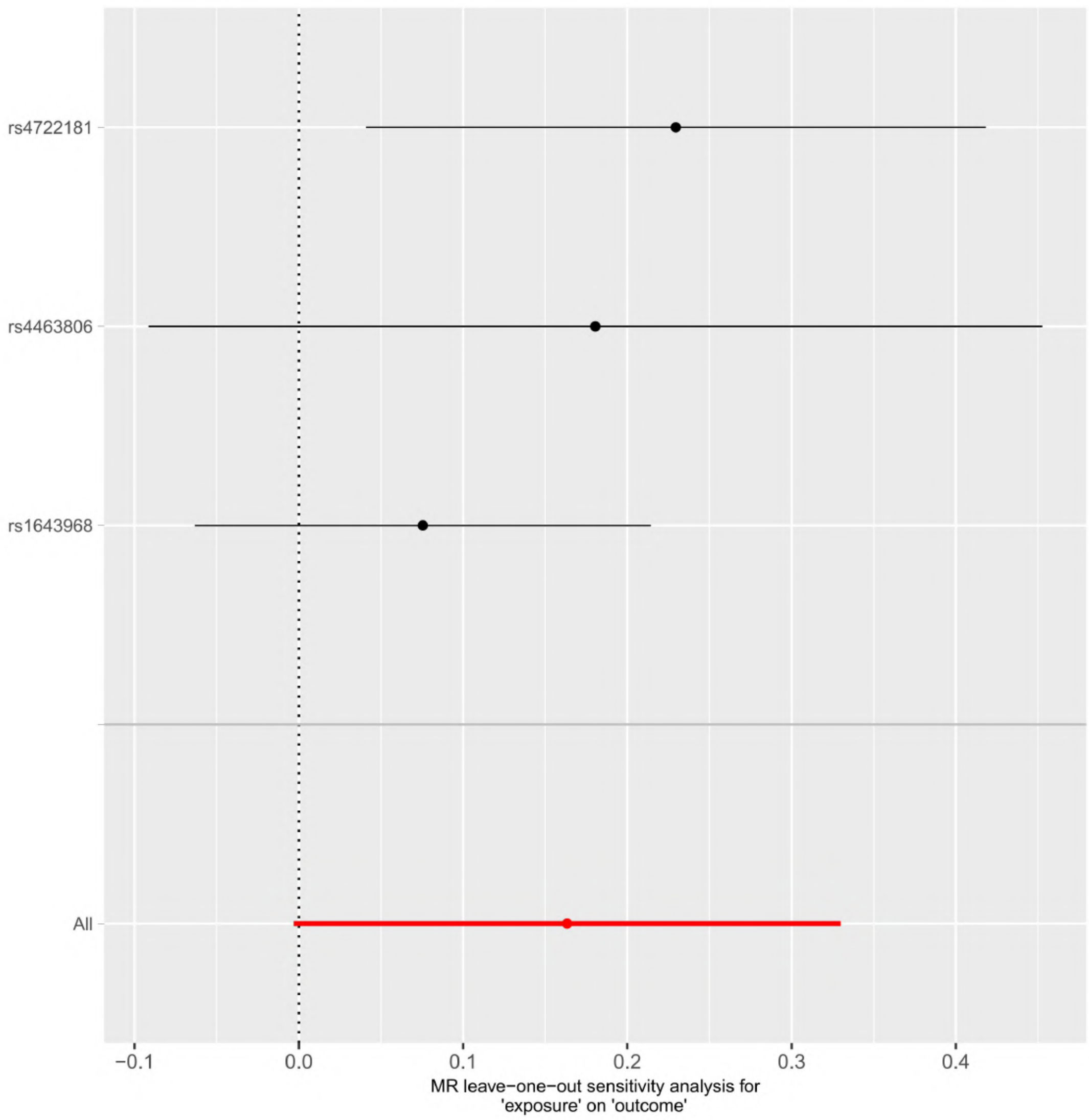

Supplementary Fig. 152 Leave-one-out inverse-variance weighted mendelian randomization analysis of class.Negativicutes.id.2164 on GERD

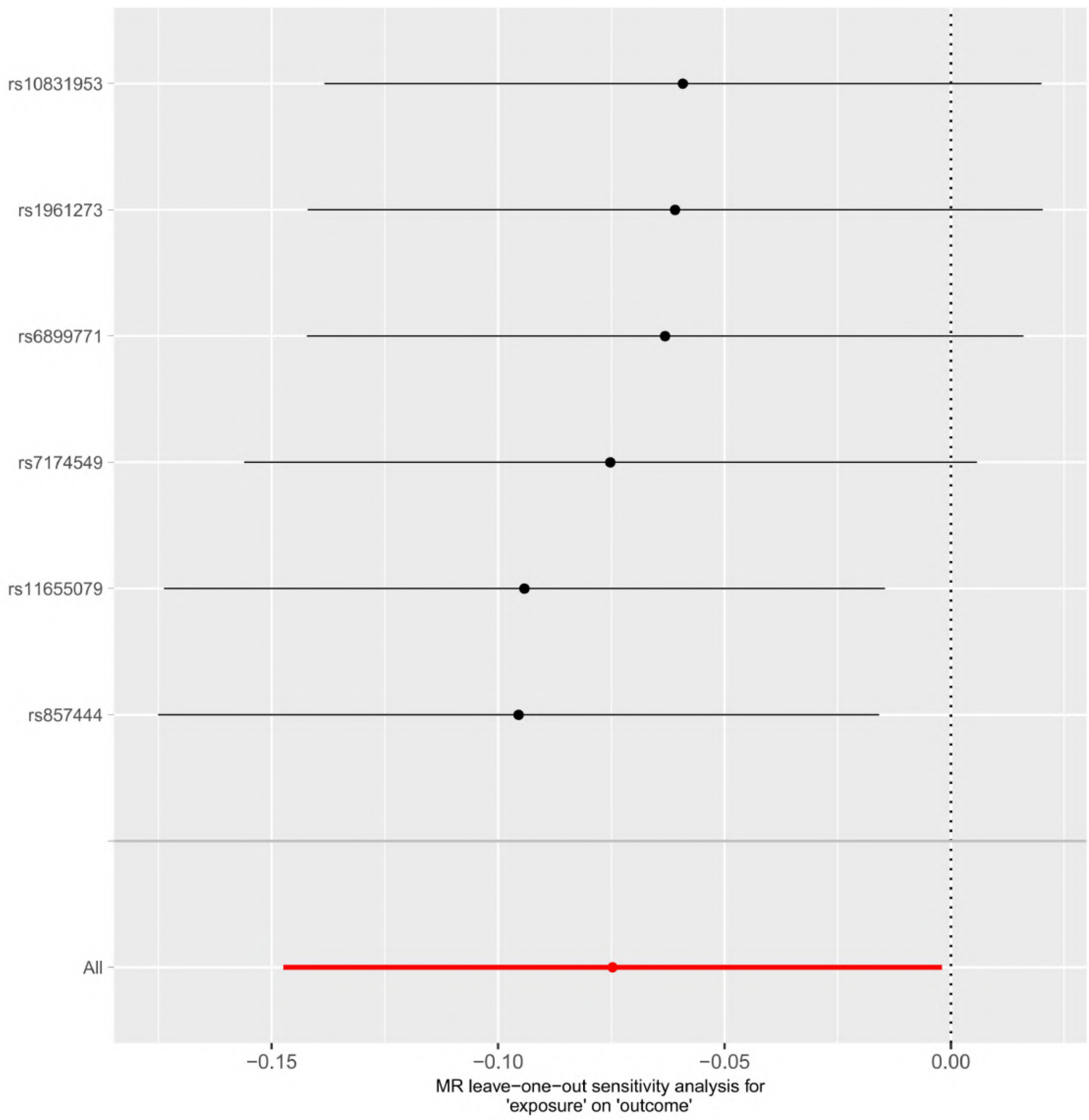

Supplementary Fig. 153 Leave-one-out inverse-variance weighted mendelian randomization analysis of family.Bifidobacteriaceae.id.433 on GERD

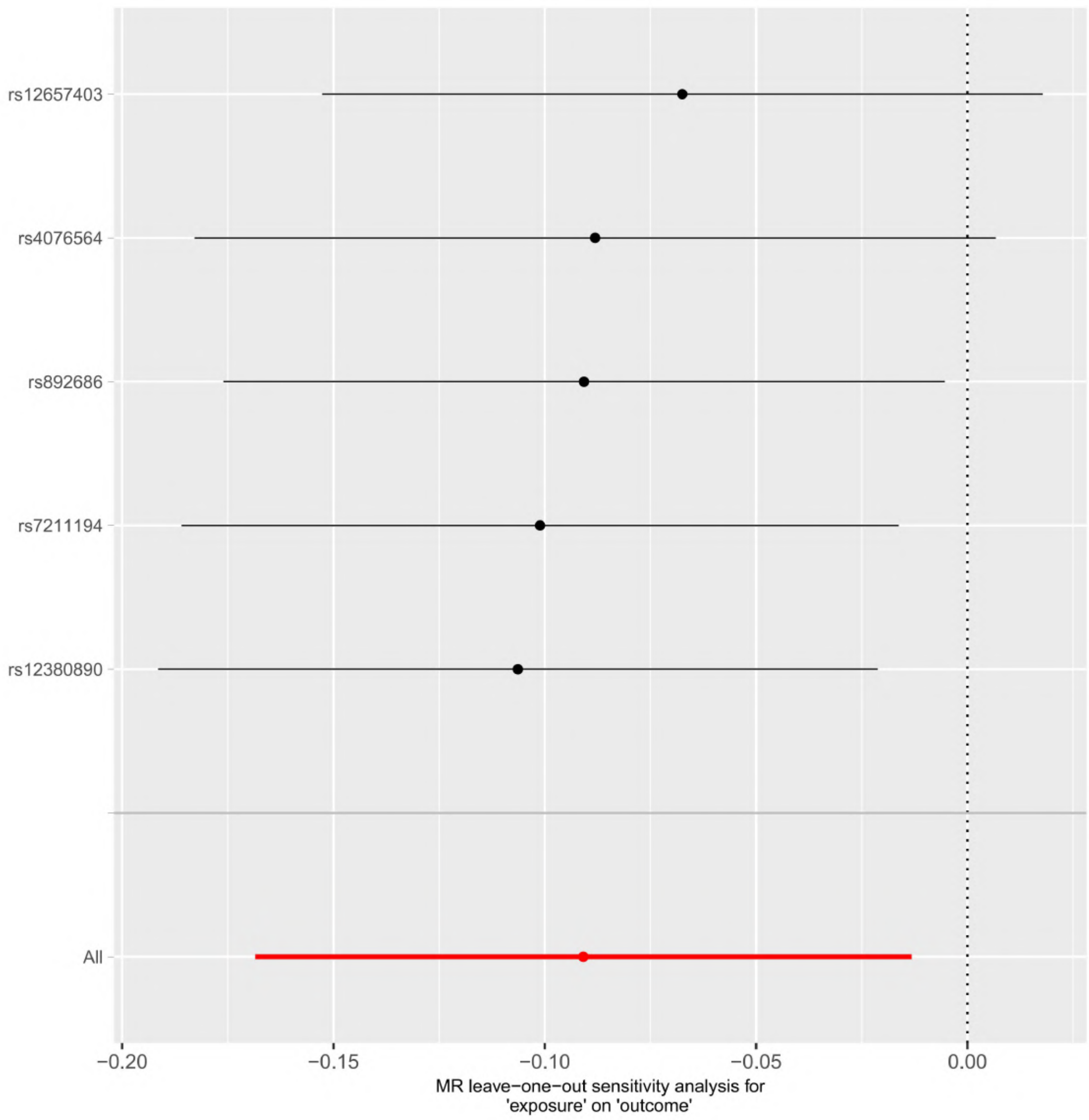

Supplementary Fig. 154 Leave-one-out inverse-variance weighted mendelian randomization analysis of family.Christensenellaceae.id.1866 on GERD

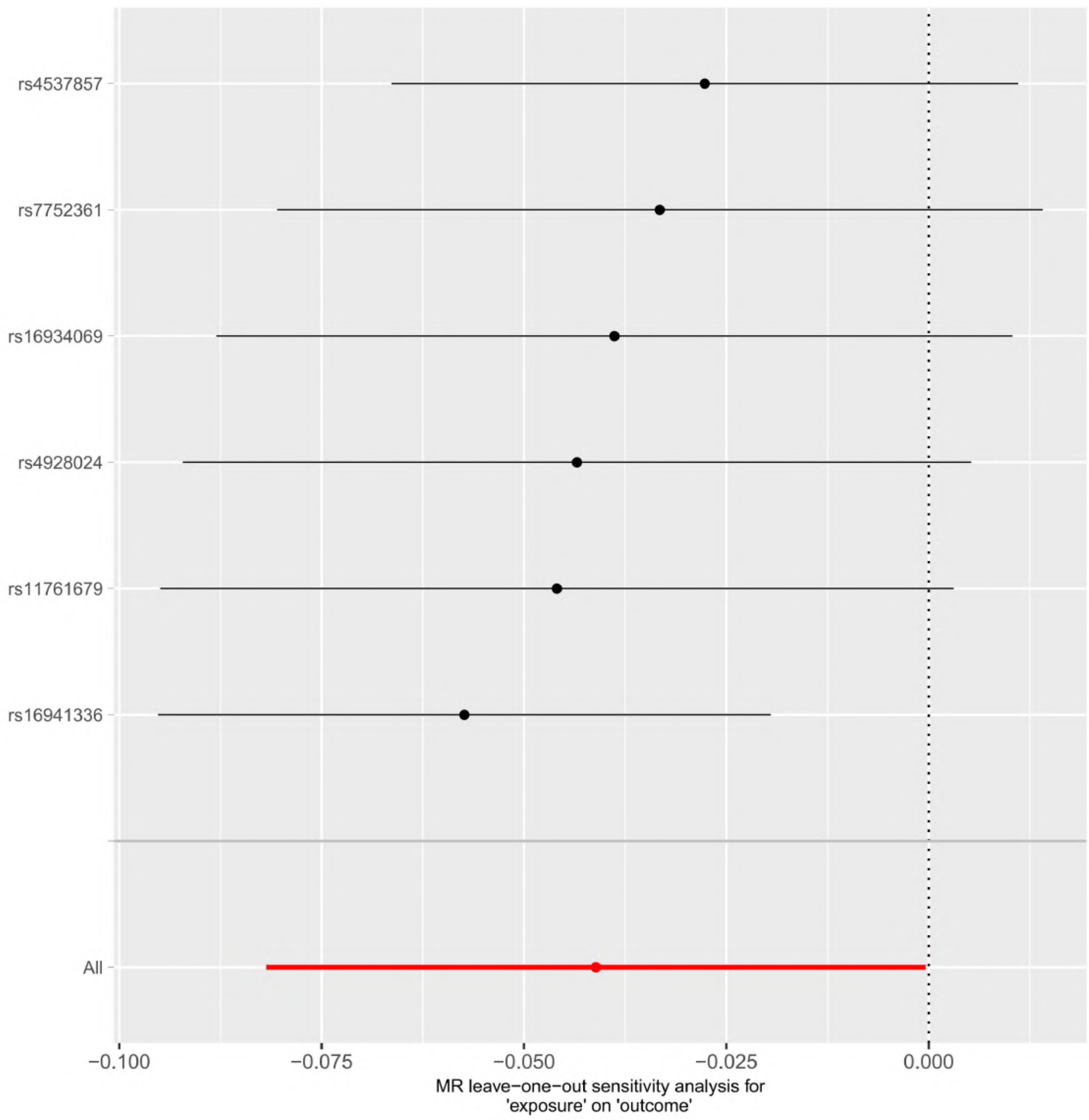

Supplementary Fig. 155 Leave-one-out inverse-variance weighted mendelian randomization analysis of genus.Butyrivibrio.id.1993 on GERD

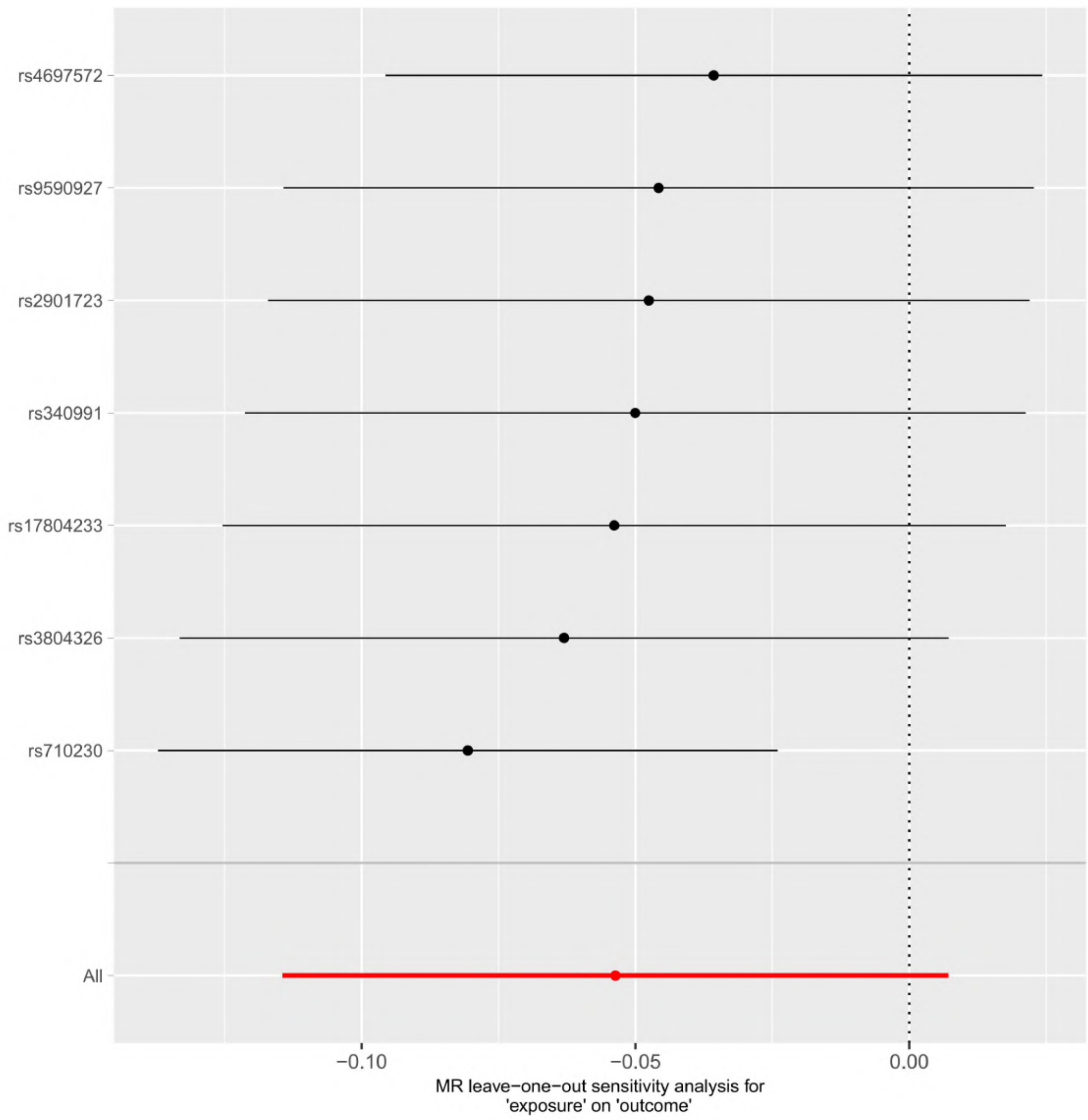

Supplementary Fig. 156 Leave-one-out inverse-variance weighted mendelian randomization analysis of genus.Erysipelatoclostridium.id.11381 on GERD

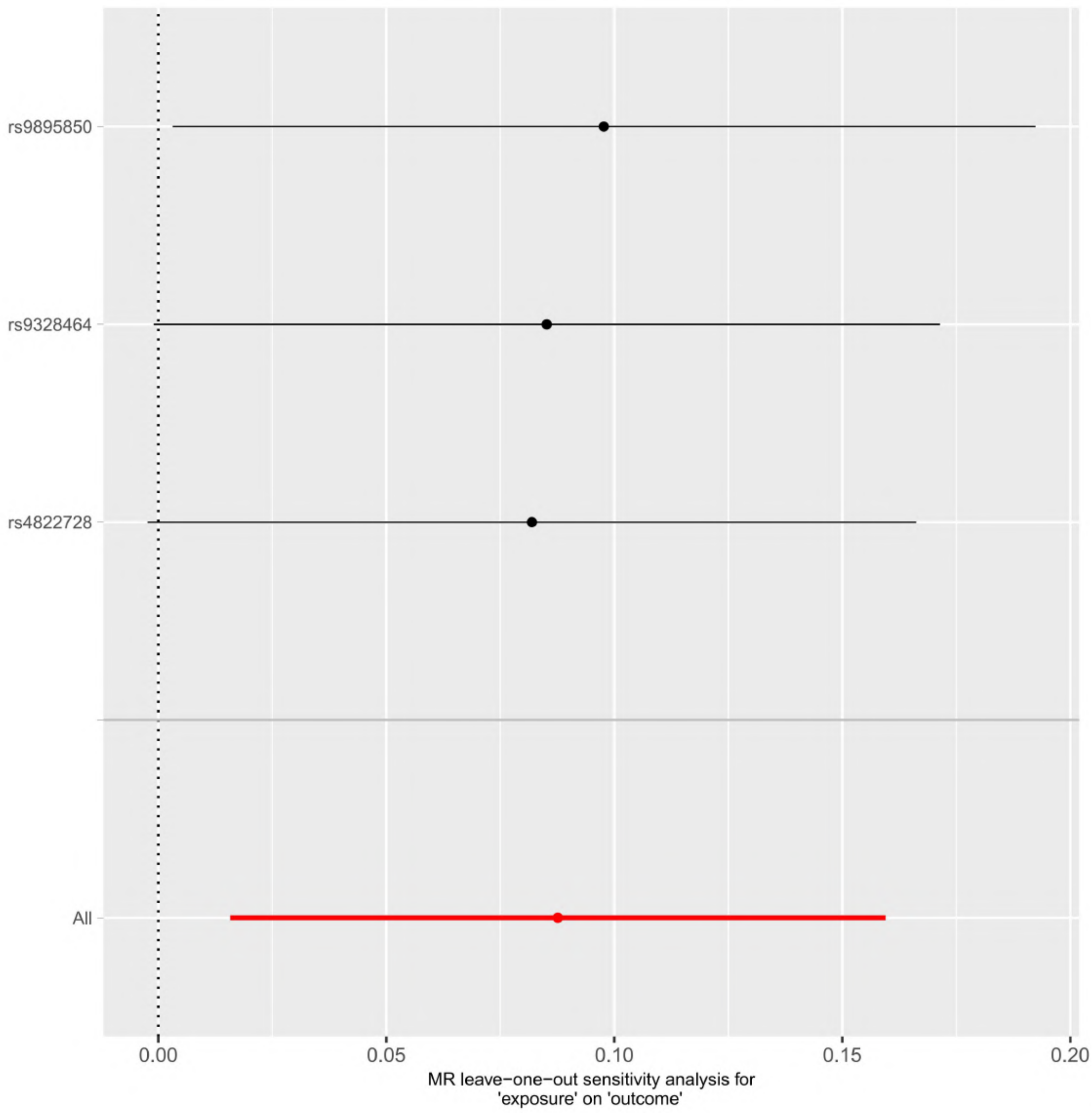

Supplementary Fig. 157 Leave-one-out inverse-variance weighted mendelian randomization analysis of genus.Haemophilus.id.3698 on GERD

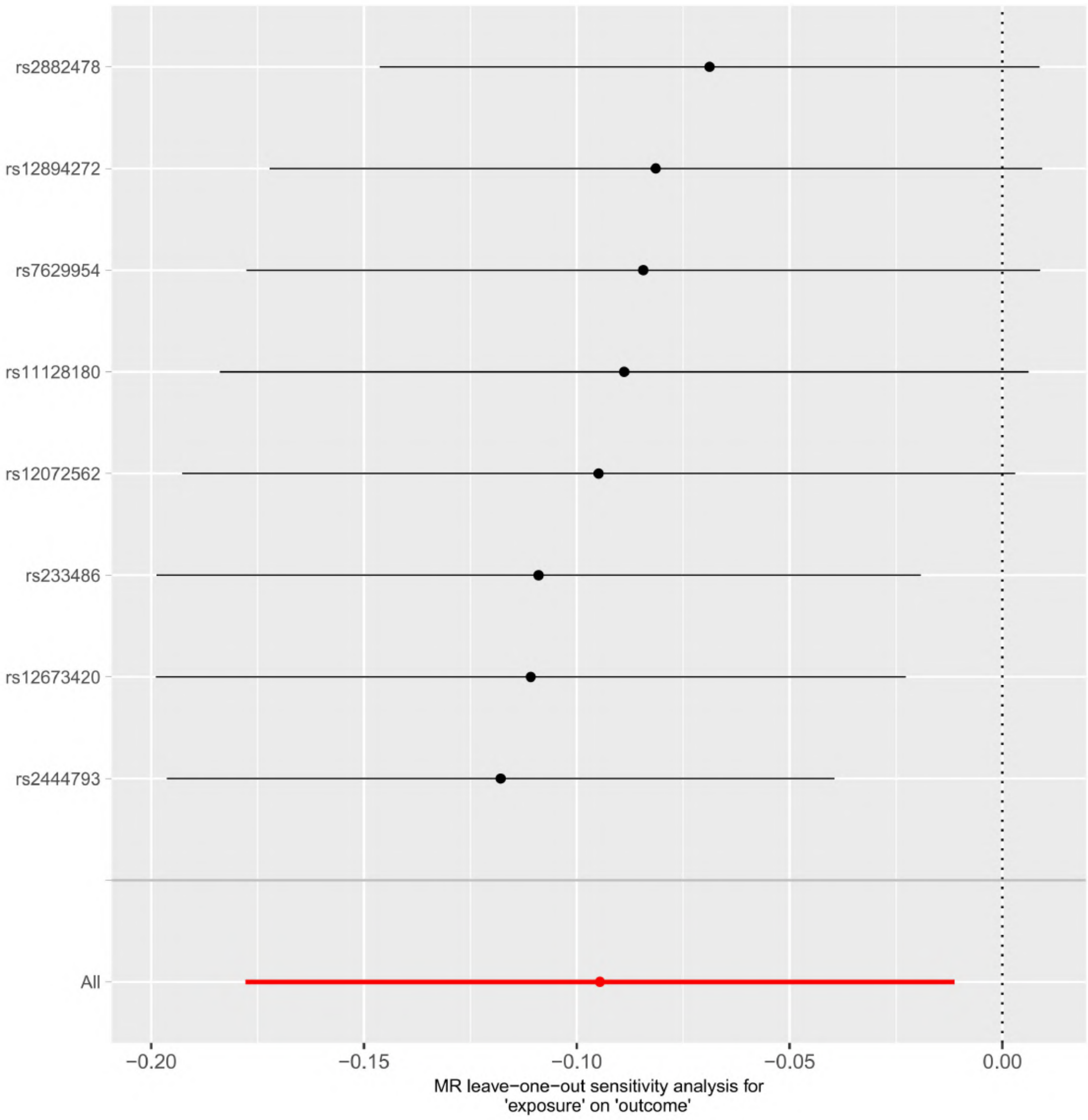

Supplementary Fig. 158 Leave-one-out inverse-variance weighted mendelian randomization analysis of genus.LachnospiraceaeUCG004.id.11324 on GERD

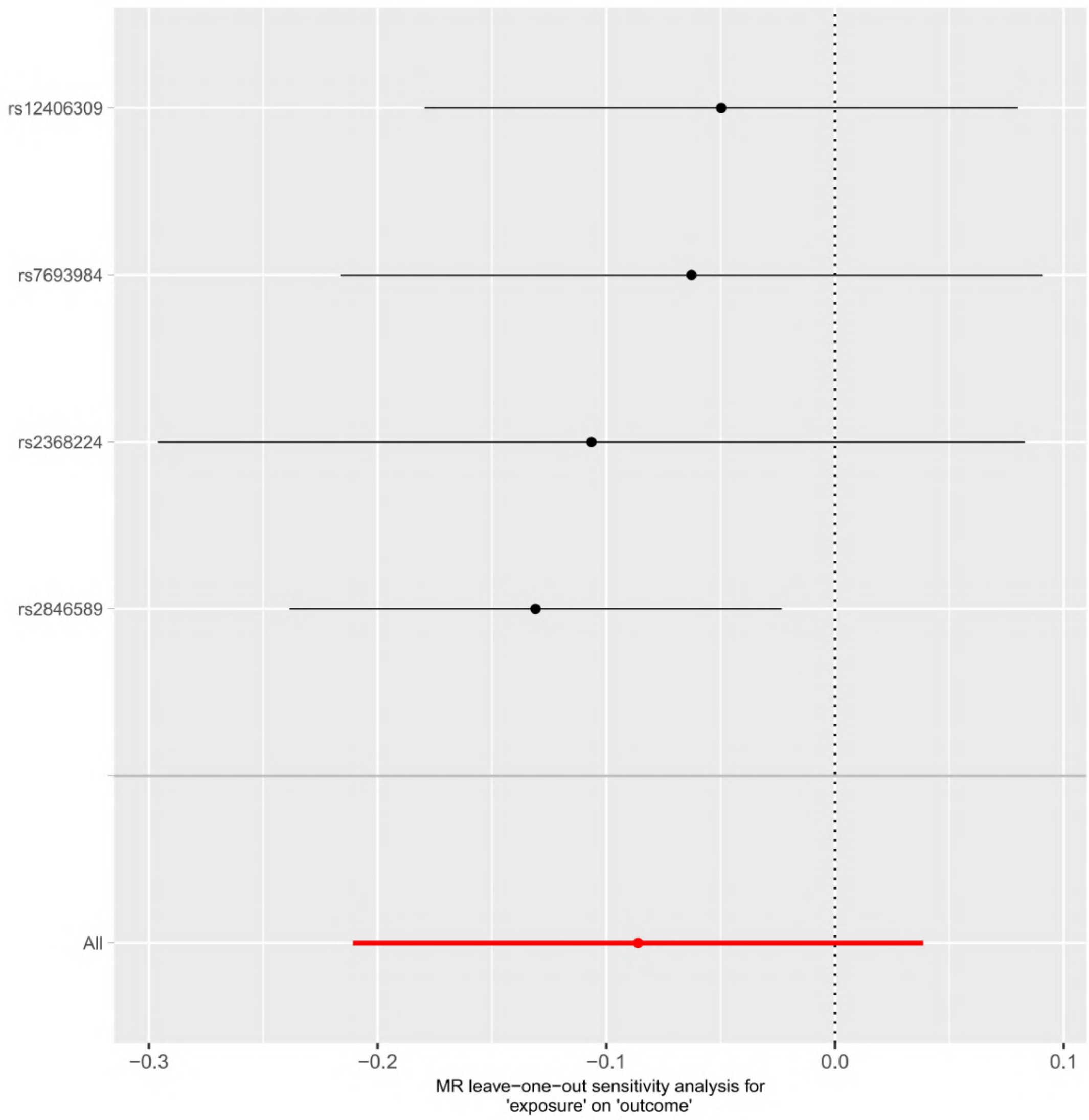

Supplementary Fig. 159 Leave-one-out inverse-variance weighted mendelian randomization analysis of genus.Ruminococcus2.id.11374 on GERD

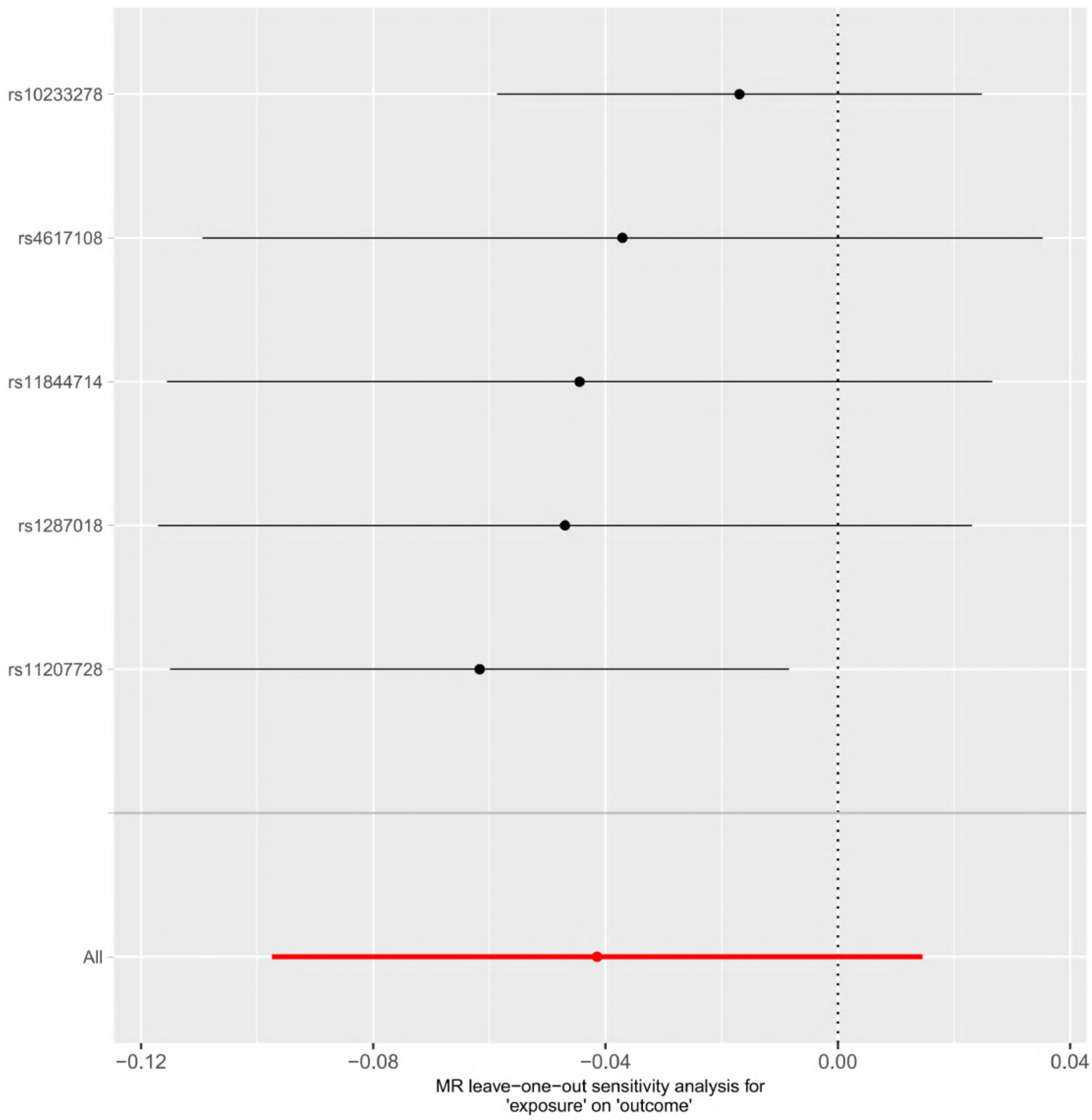

Supplementary Fig. 160 Leave-one-out inverse-variance weighted mendelian randomization analysis of order.Bacillales.id.1674 on GERD

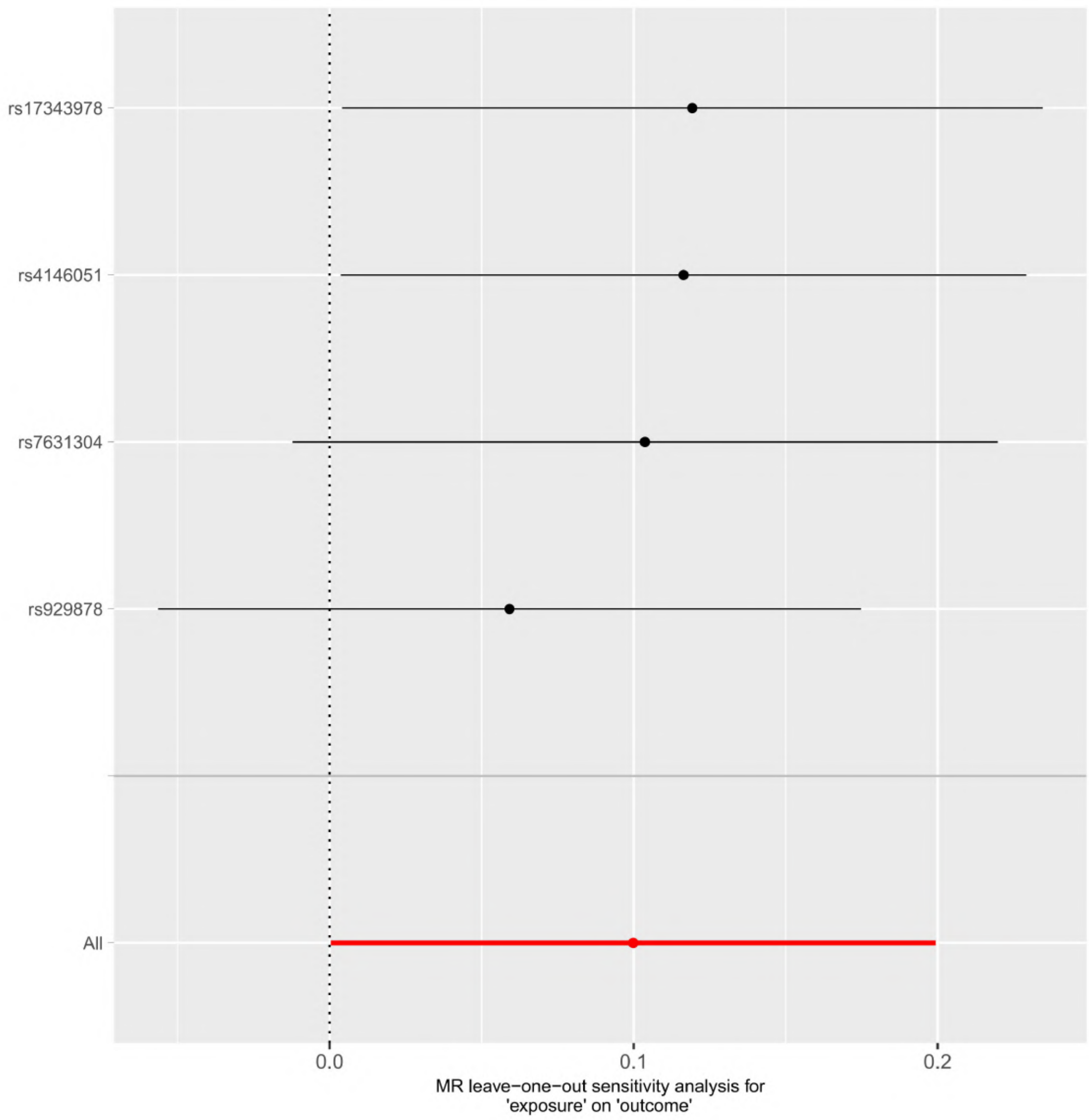

Supplementary Fig. 161 Leave-one-out inverse-variance weighted mendelian randomization analysis of order.Bacteroidales.id.913 on GERD

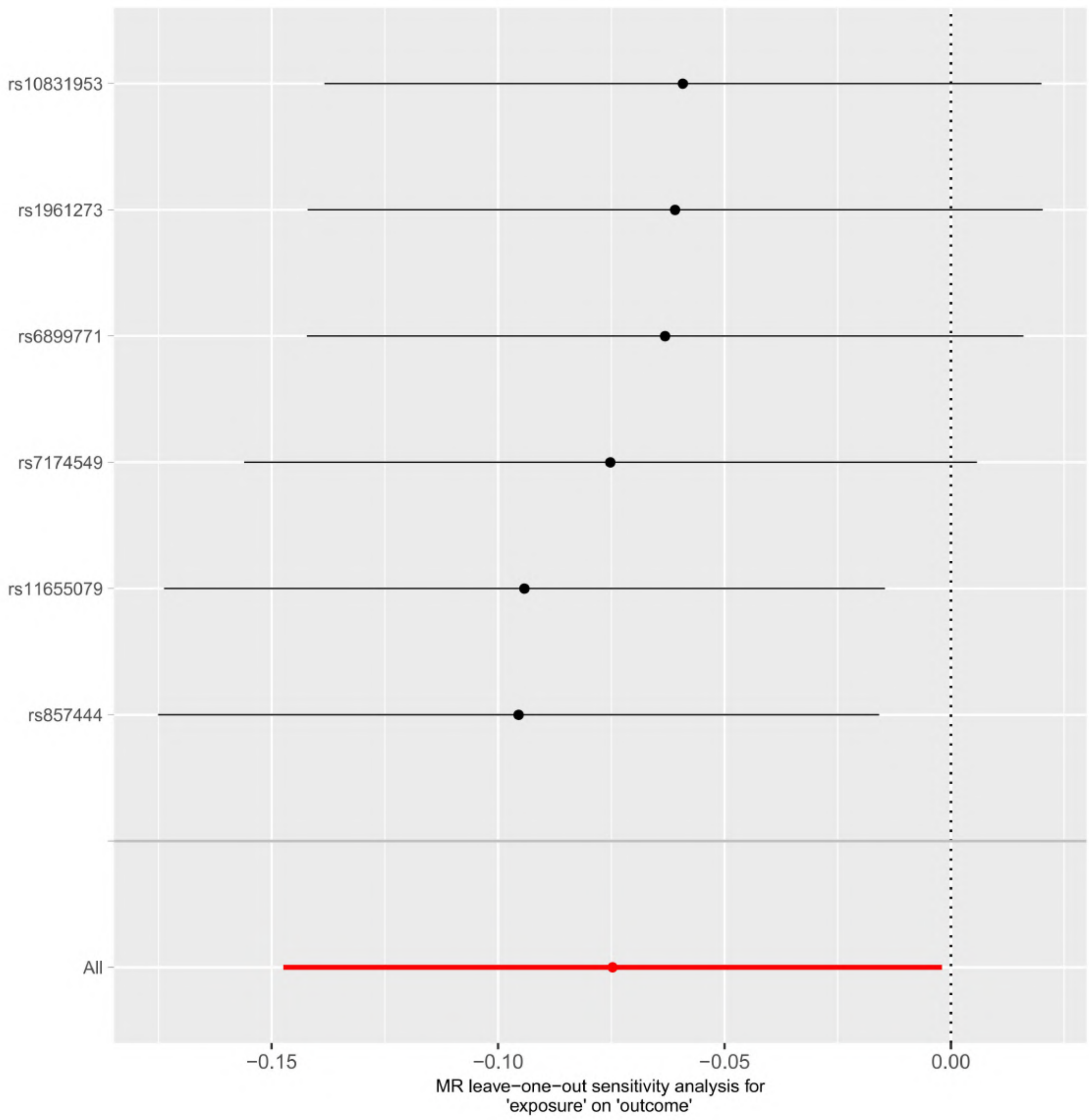

Supplementary Fig. 162 Leave-one-out inverse-variance weighted mendelian randomization analysis of order.Bifidobacteriales.id.432 on GERD

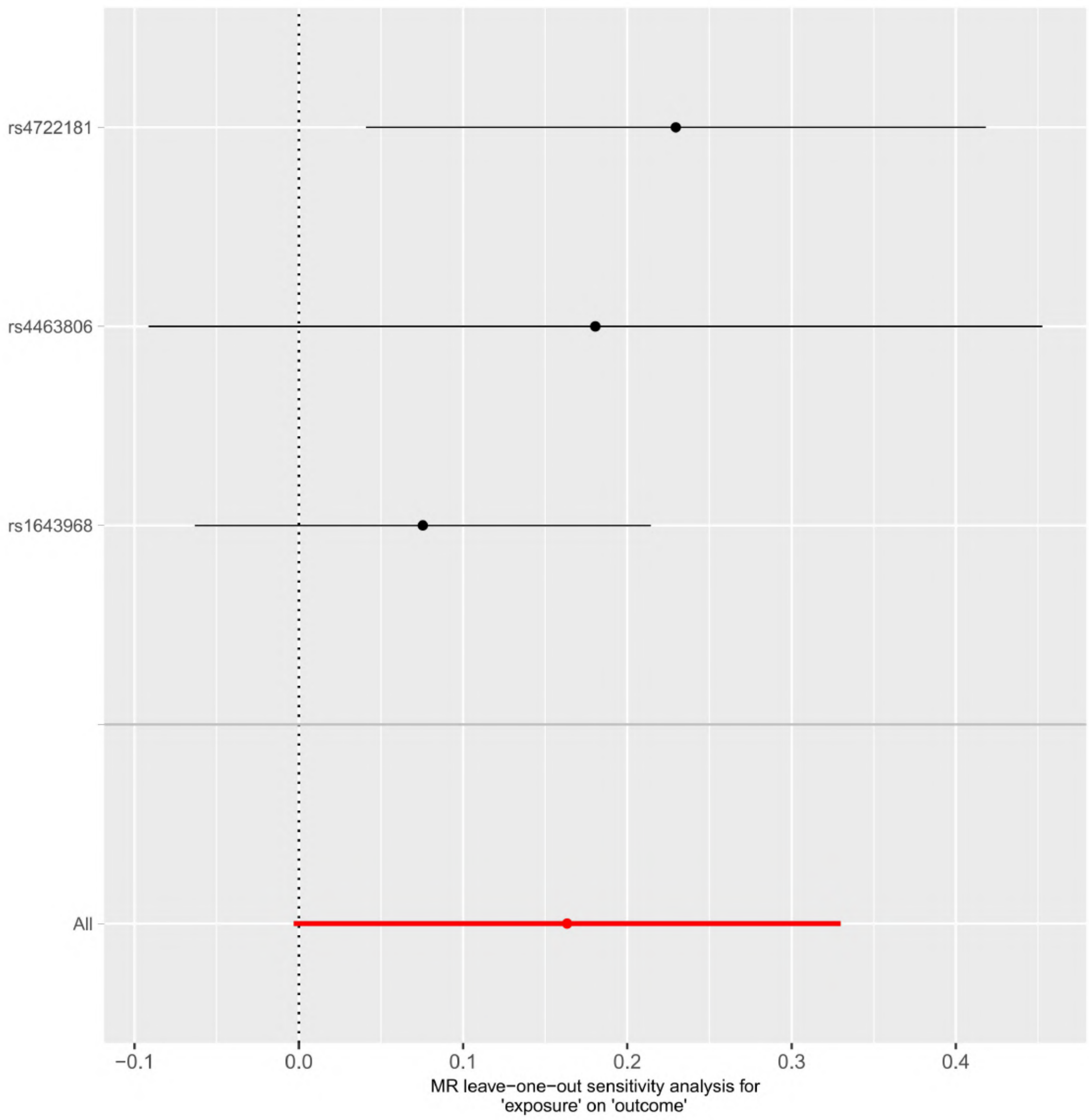

Supplementary Fig. 163 Leave-one-out inverse-variance weighted mendelian randomization analysis of order.Selenomonadales.id.2165 on GERD

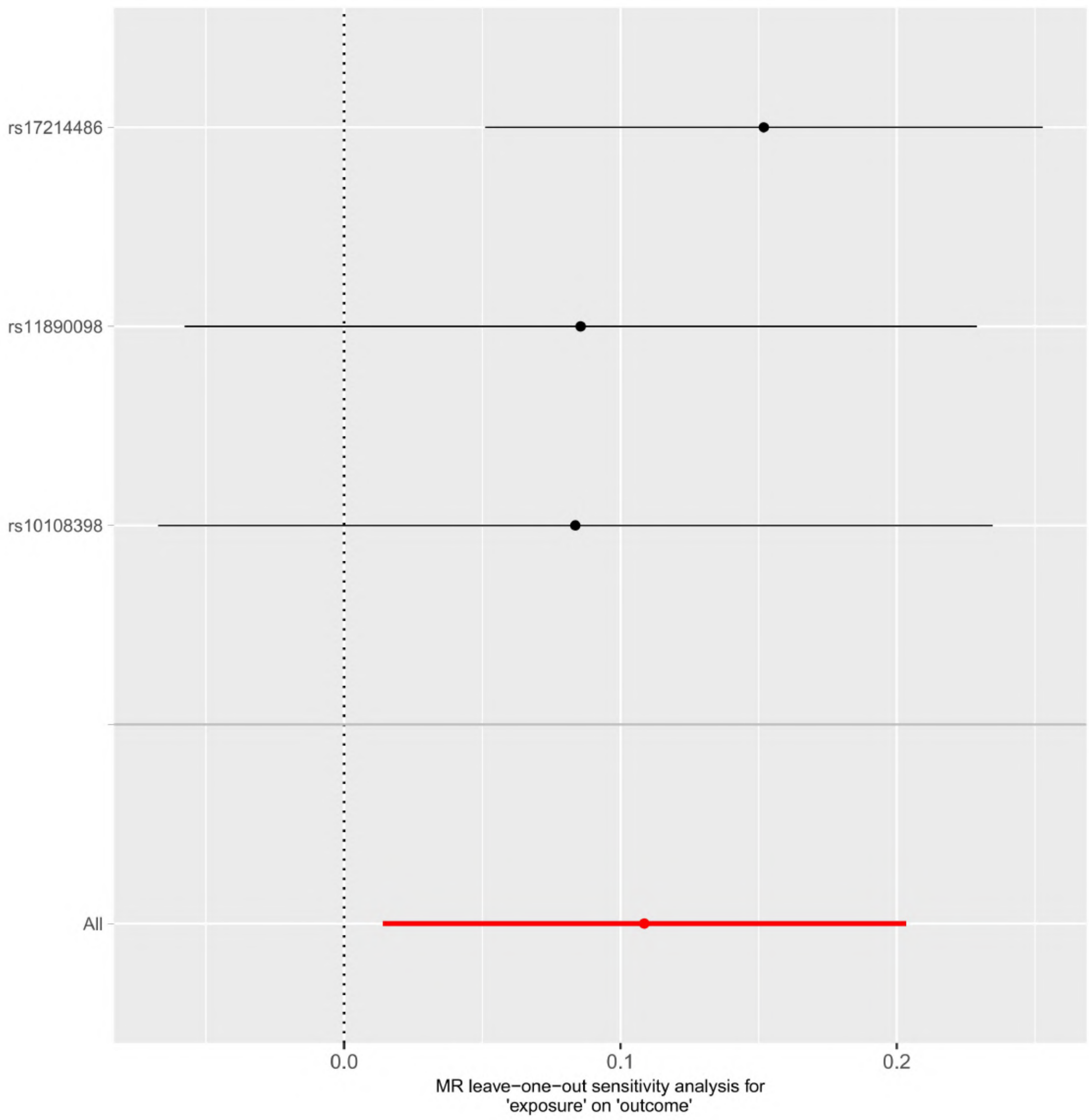

Supplementary Fig. 164 Leave-one-out inverse-variance weighted mendelian randomization analysis of phylum.Tenericutes.id.3919 on GERD

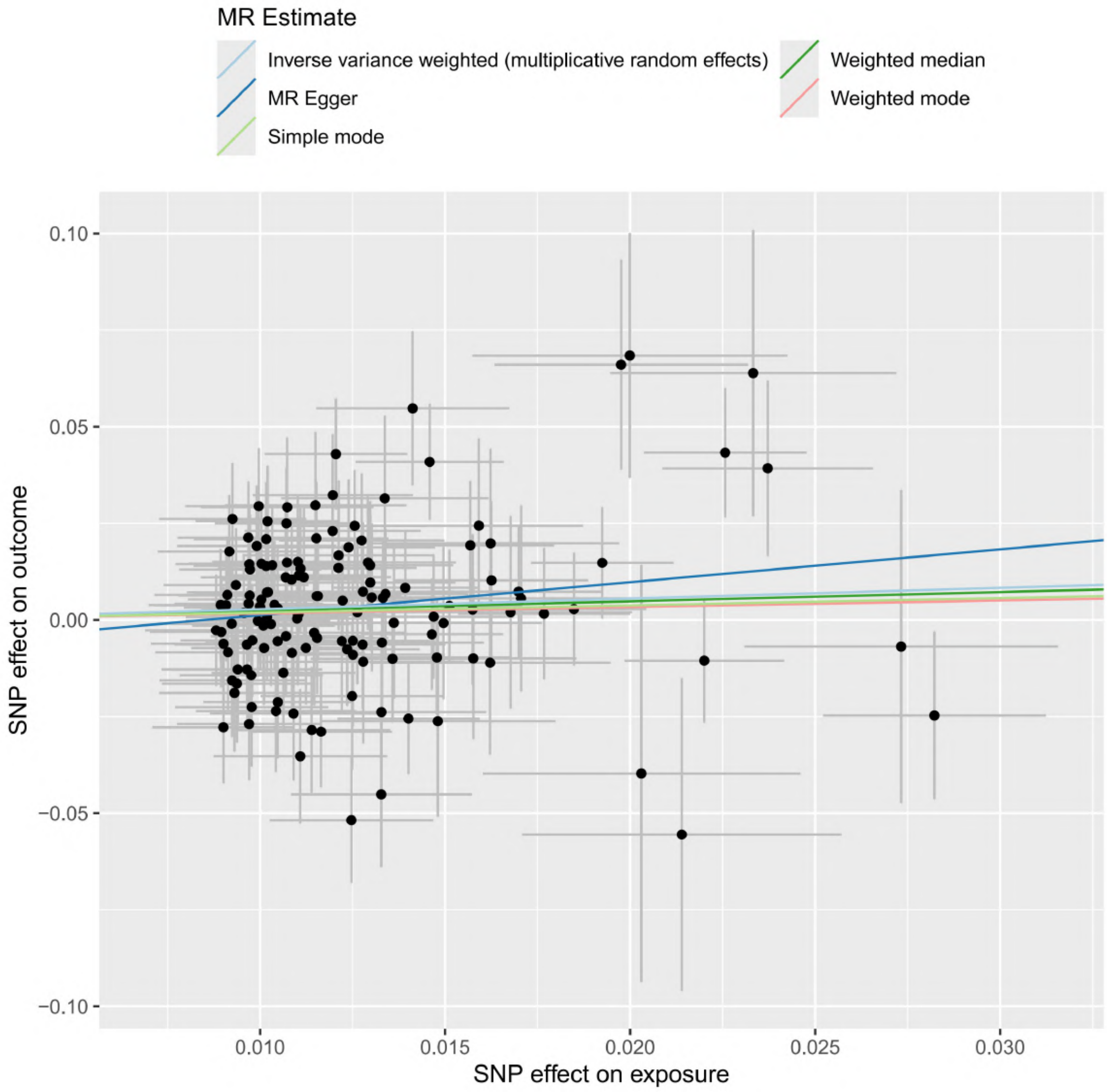

Supplementary Fig. 165 Scatter plot of the causal effect of oily fish on genus.Erysipelatoclostridium.id.11381

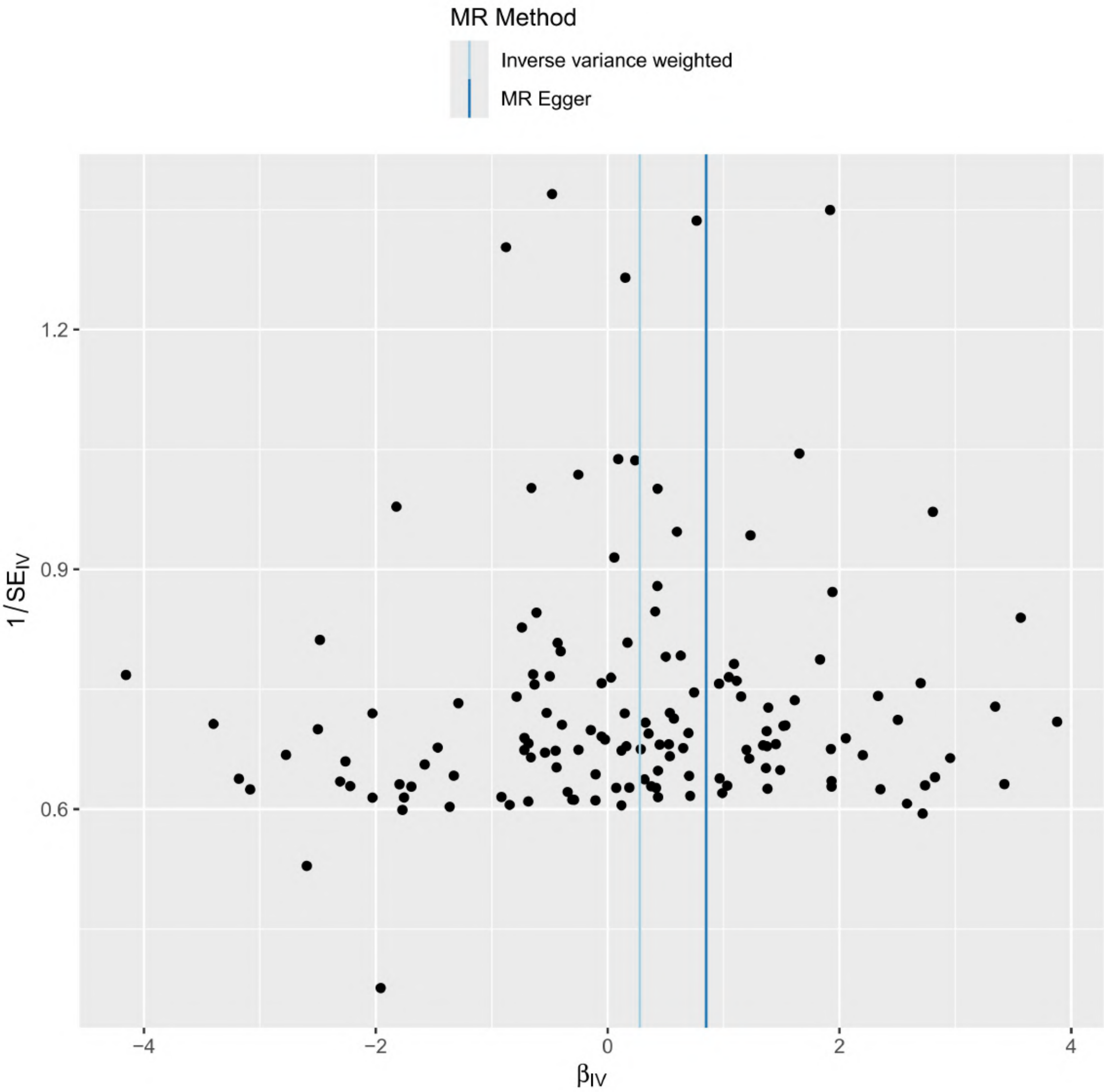

Supplementary Fig. 166 Funnel plot of the causal effect of oily fish on genus.Erysipelatoclostridium.id.11381

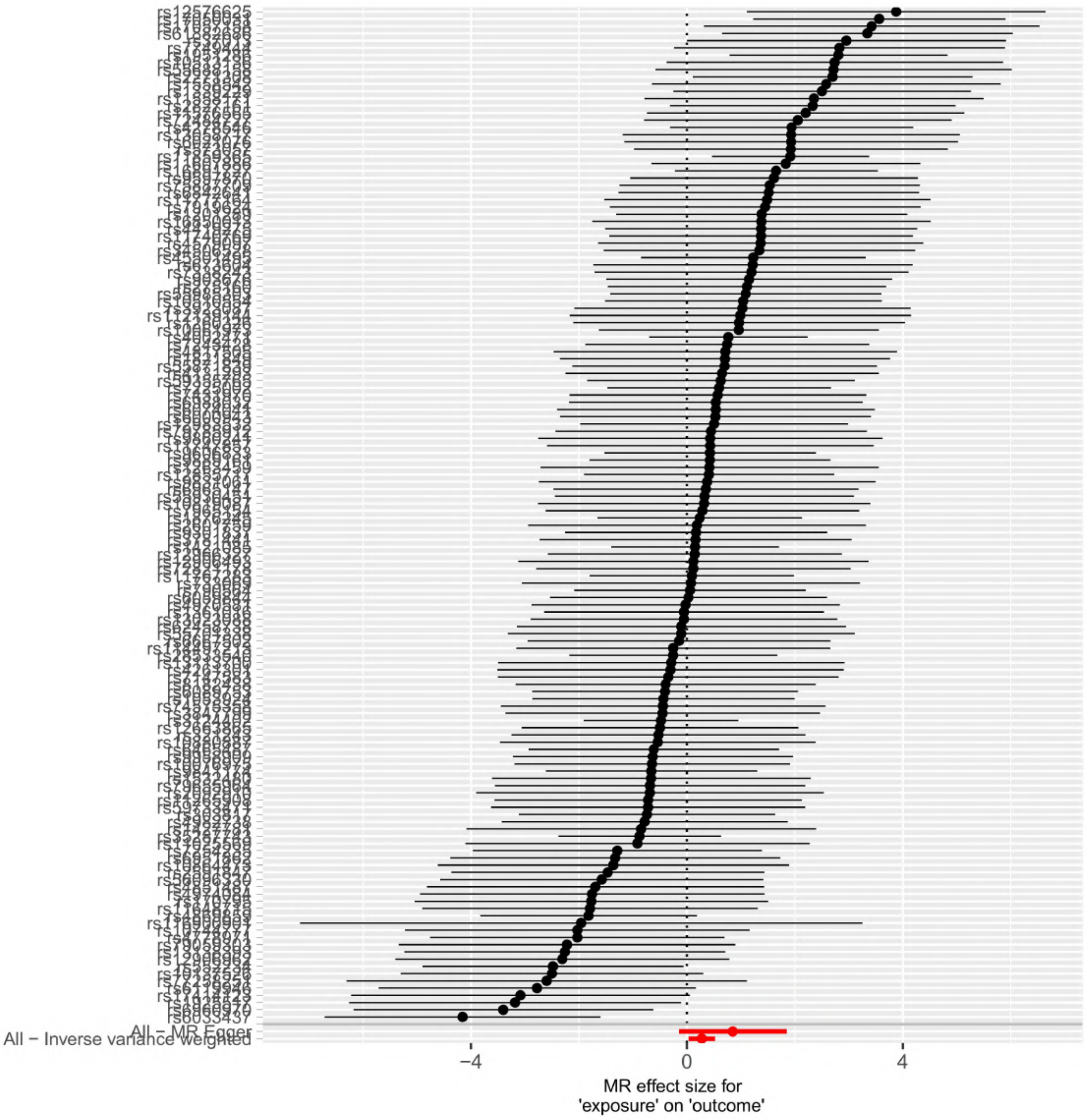

Supplementary Fig. 167 Forest plot of the causal effect of oily fish on genus.Erysipelatoclostridium.id.11381
